# Worldwide trends in diabetes prevalence and treatment from 1990 to 2022: a pooled analysis of 1108 population-representative studies with 141 million participants

**DOI:** 10.1016/S0140-6736(24)02317-1

**Published:** 2024-11

**Authors:** NCD Risk Factor Collaboration (NCD-RisC), Bin Zhou, Archie W Rayner, Edward W Gregg, Kate E Sheffer, Rodrigo M Carrillo-Larco, James E Bennett, Jonathan E Shaw, Christopher J Paciorek, Rosie K Singleton, Ana Barradas Pires, Gretchen A Stevens, Goodarz Danaei, Victor PF Lhoste, Nowell H Phelps, Rachel A Heap, Lakshya Jain, Ysé d’Ailhaud de Brisis, Agnese Galeazzi, Andre P Kengne, Anu Mishra, Nayu Ikeda, Hsien-Ho Lin, Carlos A Aguilar-Salinas, Ranjit Mohan Anjana, Habiba Ben Romdhane, Kairat Davletov, Shubash Ganapathy, Christin Heidemann, Yousef Saleh Khader, Young-Ho Khang, Avula Laxmaiah, Jean Claude N Mbanya, Viswanathan Mohan, KM Venkat Narayan, Meda E Pavkov, Eugène Sobngwi, Alisha N Wade, Novie O Younger-Coleman, Tomasz Zdrojewski, Majid Ezzati, Leandra Abarca-Gómez, Mohsen Abbasi-Kangevari, Hanan F Abdul Rahim, Niveen M Abu-Rmeileh, Shalkar Adambekov, Robert J Adams, Wichai Aekplakorn, Shoaib Afzal, Imelda A Agdeppa, Javad Aghazadeh-Attari, Carlos A Aguilar-Salinas, Charles Agyemang, Noor Ani Ahmad, Ali Ahmadi, Naser Ahmadi, Nastaran Ahmadi, Soheir H Ahmed, Wolfgang Ahrens, Kamel Ajlouni, Sarah F Al-Hamli, Halima Al-Hinai, Jawad A Al-Lawati, Deena Al Asfoor, Monira Alarouj, Fadia AlBuhairan, Shahla AlDhukair, Mohamed M Ali, Mohammed K Ali, Anna V Alieva, Farbod Alinezhad, Abdullah Alkandari, Ala’a Alkerwi, Eman Aly, Deepak N Amarapurkar, Lars Bo Andersen, Sigmund A Anderssen, Dolores S Andrade, Ranjit Mohan Anjana, Alireza Ansari-Moghaddam, Hajer Aounallah-Skhiri, Joana Araújo, Tahir Aris, Raphael E Arku, Nimmathota Arlappa, Krishna K Aryal, Thor Aspelund, Felix K Assah, Batyrbek Assembekov, Shiu Lun Au Yeung, Juha Auvinen, Mária Avdičová, Kishwar Azad, Ana Azevedo, Mohsen Azimi-Nezhad, Fereidoun Azizi, Flora Bacopoulou, Nagalla Balakrishna, Yulia Balanova, Mohamed Bamoshmoosh, Maciej Banach, Piotr Bandosz, José R Banegas, Carlo M Barbagallo, Alberto Barcelo, Maja Baretić, Lena Barrera, Marta Barreto, Abdul Basit, Anwar M Batieha, Aline P Batista, Louise A Baur, Antonisamy Belavendra, Habiba Ben Romdhane, Theodora Benedek, Mikhail Benet, Michaela Benzeval, Salim Berkinbayev, Antonio Bernabe-Ortiz, Ximena Berrios Carrasola, Heloísa Bettiol, Augustin F Beybey, Santosh K Bhargava, Yufang Bi, Elysée Claude Bika Lele, Mukharram M Bikbov, Bihungum Bista, Peter Bjerregaard, Espen Bjertness, Marius B Bjertness, Cecilia Björkelund, Katia V Bloch, Anneke Blokstra, Martin Bobak, Bernhard O Boehm, Jose G Boggia, Carlos P Boissonnet, Stig E Bojesen, Marialaura Bonaccio, Alice Bonilla-Vargas, Herman Borghs, Steve Botomba, Pascal Bovet, Imperia Brajkovich, Hermann Brenner, Lizzy M Brewster, Garry R Brian, Yajaira Briceño, Miguel Brito, Gloria Bueno, Anna Bugge, Frank Buntinx, Antonio Cabrera de León, Roberta B Caixeta, Günay Can, Ana Paula C Cândido, Mario V Capanzana, Naděžda Čapková, Eduardo Capuano, Rocco Capuano, Vincenzo Capuano, Viviane C Cardoso, Axel C Carlsson, Rodrigo M Carrillo-Larco, Felipe F Casanueva, Laura Censi, Marvin Cervantes–Loaiza, Charalambos A Chadjigeorgiou, Parinya Chamnan, Snehalatha Chamukuttan, Queenie Chan, Fadi J Charchar, Nish Chaturvedi, Chien-Jen Chen, Huashuai Chen, Long-Sheng Chen, Ching-Yu Cheng, Bahman Cheraghian, Angela Chetrit, Shu-Ti Chiou, María-Dolores Chirlaque, Jerzy Chudek, Renata Cifkova, Massimo Cirillo, Frank Claessens, Janine Clarke, Emmanuel Cohen, Hans Concin, Cyrus Cooper, Cojocaru R Cosmin, Simona Costanzo, Melanie J Cowan, Chris Cowell, Amelia C Crampin, Ana B Crujeiras, Juan J Cruz, Felipe V Cureau, Sarah Cuschieri, Graziella D’Arrigo, Eleonora d’Orsi, Haroldo da Silva-Ferreira, Jean Dallongeville, Albertino Damasceno, Rachel Dankner, Saeed Dastgiri, Luc Dauchet, Kairat Davletov, Amalia De Curtis, Giovanni de Gaetano, Stefaan De Henauw, David De Ridder, Mohan Deepa, Vincent DeGennaro, Stefaan Demarest, Elaine Dennison, Valérie Deschamps, Meghnath Dhimal, Zivka Dika, Shirin Djalalinia, Chiara Donfrancesco, Maria Dorobantu, Nico Dragano, Wojciech Drygas, Shufa Du, Yong Du, Charmaine A Duante, Priscilla Duboz, Rosemary B Duda, Anar Dushpanova, Vilnis Dzerve, Elzbieta Dziankowska-Zaborszczyk, Narges Ebrahimi, Ricky Eddie, Ebrahim Eftekhar, Vasiliki Efthymiou, Eruke E Egbagbe, Robert Eggertsen, Sareh Eghtesad, Clara Ladi Ejembi, Mohammad El-Khateeb, Jalila El Ati, Denise Eldemire-Shearer, Roberto Elosua, Ofem Enang, Rajiv T Erasmus, Cihangir Erem, Gul Ergor, Louise Eriksen, Johan G Eriksson, Ali Esmaeili, Roger G Evans, Guy Fagherazzi, Noushin Fahimfar, Ildar Fakhradiyev, Albina A Fakhretdinova, Caroline H Fall, Elnaz Faramarzi, Mojtaba Farjam, Farshad Farzadfar, Yosef Farzi, Mohammad Reza Fattahi, Asher Fawwad, Francisco J Felix-Redondo, Trevor S Ferguson, Daniel Fernández-Bergés, Desha R Fernando, Thomas Ferrao, Marika Ferrari, Marco M Ferrario, Catterina Ferreccio, Eldridge Ferrer, Edith JM Feskens, Günther Fink, David Flood, Maria Forsner, Sandrine Fosse-Edorh, Edward F Fottrell, Heba M Fouad, Damian K Francis, Guillermo Frontera, Isti I Fujiati, Matsuda Fumihiko, Takuro Furusawa, Zbigniew Gaciong, Fabio Galvano, Sarah P Garnett, Jean-Michel Gaspoz, Magda Gasull, Andrea Gazzinelli, Ulrike Gehring, Ebrahim Ghaderi, Seyyed-Hadi Ghamari, Ali Ghanbari, Erfan Ghasemi, Oana-Florentina Gheorghe-Fronea, Anup Ghimire, Alessandro Gialluisi, Simona Giampaoli, Francesco Gianfagna, Tiffany K Gill, Jonathan Giovannelli, Glen Gironella, Aleksander Giwercman, Marcel Goldberg, David Goltzman, Aleksandra Gomula, Helen Gonçalves, Mauer Gonçalves, David A Gonzalez-Chica, Marcela Gonzalez-Gross, Juan P González-Rivas, Angel R Gonzalez, Atsushi Goto, Frederic Gottrand, Dušan Grafnetter, Maria G Grammatikopoulou, Andriene Grant, Anne Sameline Grimsgaard, Tomasz Grodzicki, Anders Grøntved, Giuseppe Grosso, Dongfeng Gu, Vilmundur Gudnason, Ramiro Guerrero, Idris Guessous, Unjali P Gujral, Rajeev Gupta, Laura Gutierrez, Xinyi Gwee, Seongjun Ha, Rosa Haghshenas, Hamid Hakimi, Ian R Hambleton, Behrooz Hamzeh, Dominique Hange, Sari Hantunen, Jie Hao, Javad Harooni, Seyed Mohammad Hashemi-Shahri, Jun Hata, Alison J Hayes, Jiang He, Christin Heidemann, Rafael dos Santos Henrique, Ana Henriques, Sauli Herrala, Karl-Heinz Herzig, Ramin Heshmat, Allan G Hill, Sai Yin Ho, Michelle Holdsworth, Reza Homayounfar, Wilma M Hopman, Andrea RVR Horimoto, Claudia Hormiga, Bernardo L Horta, Leila Houti, Christina Howitt, Thein Thein Htay, Aung Soe Htet, Maung Maung Than Htike, José María Huerta, Ilpo Tapani Huhtaniemi, Laetitia Huiart, Martijn Huisman, Monica Hunsberger, Abdullatif Husseini, Inge Huybrechts, Licia Iacoviello, Ellina M Iakupova, Anna G Iannone, Norazizah Ibrahim Wong, Chinwuba Ijoma, Nayu Ikeda, Vilma E Irazola, Takafumi Ishida, Sheikh Mohammed Shariful Islam, Duygu Islek, Till Ittermann, Masanori Iwasaki, Tuija Jääskeläinen, Jeremy M Jacobs, Hashem Y Jaddou, Michel Jadoul, Bakary Jallow, Kenneth James, Kazi M Jamil, Edward Janus, Marjo-Riitta Jarvelin, Grazyna Jasienska, Ana Jelaković, Bojan Jelaković, Garry Jennings, Anjani Kumar Jha, AM Jibo, Ramon O Jimenez, Karl-Heinz Jöckel, Jari J Jokelainen, Jost B Jonas, Josipa Josipović, Farahnaz Joukar, Jacek Jóźwiak, Anthony Kafatos, Eero O Kajantie, Zhanna Kalmatayeva, Ofra Kalter-Leibovici, Argyro Karakosta, Khem B Karki, Marzieh Katibeh, Prasad Katulanda, Jussi Kauhanen, Gyulli M Kazakbaeva, François F Kaze, Calvin Ke, Sirkka Keinänen-Kiukaanniemi, Roya Kelishadi, Andre P Kengne, Maryam Keramati, Mathilde Kersting, Yousef Saleh Khader, Kazem Khalagi, Arsalan Khaledifar, Davood Khalili, Young-Ho Khang, Bahareh Kheiri, Motahareh Kheradmand, Alireza Khosravi Farsani, Ursula Kiechl-Kohlendorfer, Sophia J Kiechl, Stefan Kiechl, Hyeon Chang Kim, Andrew Kingston, Heidi Klakk, Jana Klanova, Michael Knoflach, Patrick Kolsteren, Jürgen König, Raija Korpelainen, Paul Korrovits, Jelena Kos, Seppo Koskinen, Sudhir Kowlessur, Slawomir Koziel, Wolfgang Kratzer, Susi Kriemler, Peter Lund Kristensen, Steinar Krokstad, Daan Kromhout, Ruzena Kubinova, Urho M Kujala, Mukhtar Kulimbet, Vaitheeswaran Kulothungan, Meena Kumari, Vladimir Kutsenko, Catherine Kyobutungi, Quang Ngoc La, Tiina Laatikainen, Demetre Labadarios, Carl Lachat, Youcef Laid, Lachmie Lall, Anne Langsted, Tiina Lankila, Vera Lanska, Georg Lappas, Bagher Larijani, Tint Swe Latt, Martino Laurenzi, Avula Laxmaiah, Gwenaëlle Le Coroller, Jeannette Lee, Terho Lehtimäki, Daniel Lemogoum, Gabriel M Leung, Charlie Lim, Wei-Yen Lim, M Fernanda Lima-Costa, Hsien-Ho Lin, Yi-Jing Lin, Lars Lind, Lauren Lissner, Liping Liu, Xiaotian Liu, Wei-Cheng Lo, Helle-Mai Loit, Esther Lopez-Garcia, Tania Lopez, José Eugenio Lozano, Dalia Luksiene, Annamari Lundqvist, Nuno Lunet, Thomas Lung, Michala Lustigová, Guansheng Ma, George LL Machado-Coelho, Aristides M Machado-Rodrigues, Enguerran Macia, Luisa M Macieira, Ahmed A Madar, Gladys E Maestre, Stefania Maggi, Dianna J Magliano, Emmanuella Magriplis, Gowri Mahasampath, Bernard Maire, Marcia Makdisse, Konstantinos Makrilakis, Mohammad-Reza Malekpour, Fatemeh Malekzadeh, Reza Malekzadeh, Sofia Malyutina, Lynell V Maniego, Yannis Manios, Fariborz Mansour-Ghanaei, Enzo Manzato, Mala Ali Mapatano, Anie Marcil, Francisco Mardones, Paula Margozzini, Pedro Marques-Vidal, Larissa Pruner Marques, Reynaldo Martorell, Luis P Mascarenhas, Mannix Masimango Imani, Masoud Masinaei, Shariq R Masoodi, Ellisiv B Mathiesen, Prashant Mathur, Tandi E Matsha, Jean Claude N Mbanya, Anselmo J Mc Donald Posso, Shelly R McFarlane, Stephen T McGarvey, Scott B McLean, Breige A McNulty, Sounnia Mediene Benchekor, Kirsten Mehlig, Amir Houshang Mehrparvar, Jesus D Melgarejo, Fabián Méndez, Ana Maria B Menezes, Alibek Mereke, Indrapal I Meshram, Diane T Meto, Nathalie Michels, Cláudia S Minderico, GK Mini, Juan Francisco Miquel, J Jaime Miranda, Mohammad Reza Mirjalili, Daphne Mirkopoulou, Pietro A Modesti, Sahar Saeedi Moghaddam, Kazem Mohammad, Mohammad Reza Mohammadi, Zahra Mohammadi, Noushin Mohammadifard, Reza Mohammadpourhodki, Viswanathan Mohan, Muhammad Fadhli Mohd Yusoff, Iraj Mohebbi, Niels C Møller, Dénes Molnár, Amirabbas Momenan, Roger A Montenegro Mendoza, Mahmood Moosazadeh, Farhad Moradpour, Alain Morejon, Luis A Moreno, Karen Morgan, Suzanne N Morin, George Moschonis, Alireza Moslem, Mildrey Mosquera, Malgorzata Mossakowska, Aya Mostafa, Seyed-Ali Mostafavi, Eugen Mota, Jorge Mota, Maria Mota, Mohammad Esmaeel Motlagh, Jorge Motta, Kelias P Msyamboza, Thet Thet Mu, Maria L Muiesan, Patricia B Munroe, Jaakko Mursu, Kamarul Imran Musa, Norlaila Mustafa, Muel Telo MC Muyer, Iraj Nabipour, Gabriele Nagel, Balkish M Naidu, Farid Najafi, Jana Námešná, Ei Ei K Nang, Vinay B Nangia, KM Venkat Narayan, Take Naseri, Ana J Navarro-Ramírez, Nareemarn Neelapaichit, Azim Nejatizadeh, Ilona Nenko, Flavio Nervi, Tze Pin Ng, Chung T Nguyen, Nguyen D Nguyen, Quang Ngoc Nguyen, Michael Y Ni, Peng Nie, Ramfis E Nieto-Martínez, Guang Ning, Toshiharu Ninomiya, Nobuo Nishi, Marianna Noale, Oscar A Noboa, Mitsuhiko Noda, Børge G Nordestgaard, Davide Noto, Mohannad Al Nsour, Irfan Nuhoğlu, Moffat Nyirenda, Terence W O’Neill, Kyungwon Oh, Ryutaro Ohtsuka, Mohd Azahadi Omar, Altan Onat, Sok King Ong, Obinna Onodugo, Pedro Ordunez, Rui Ornelas, Pedro J Ortiz, Clive Osmond, Afshin Ostovar, Johanna A Otero, Charlotte B Ottendahl, Akaninyene Otu, Ellis Owusu-Dabo, Elena Pahomova, Luigi Palmieri, Wen-Harn Pan, Demosthenes Panagiotakos, Songhomitra Panda-Jonas, Zengchang Pang, Francesco Panza, Mariela Paoli, Suyeon Park, Mahboubeh Parsaeian, Chona F Patalen, Nikhil D Patel, Raimund Pechlaner, Ivan Pećin, João M Pedro, Sergio Viana Peixoto, Markku Peltonen, Alexandre C Pereira, Thaliane Mayara Pessôa dos Prazeres, Niloofar Peykari, Modou Cheyassin Phall, Son Thai Pham, Rafael N Pichardo, Iris Pigeot, Hynek Pikhart, Aida Pilav, Pavel Piler, Freda Pitakaka, Aleksandra Piwonska, Andreia N Pizarro, Pedro Plans-Rubió, Silvia Plata, Barry M Popkin, Miquel Porta, Anil Poudyal, Farhad Pourfarzi, Akram Pourshams, Hossein Poustchi, Dorairaj Prabhakaran, Rajendra Pradeepa, Alison J Price, Jacqueline F Price, Rui Providencia, Jardena J Puder, Soile Puhakka, Margus Punab, Qing Qiao, Mostafa Qorbani, Hedley K Quintana, Tran Quoc Bao, Ricardas Radisauskas, Salar Rahimikazerooni, Olli Raitakari, Ambady Ramachandran, Manuel Ramirez-Zea, Jacqueline Ramke, Elisabete Ramos, Rafel Ramos, Lekhraj Rampal, Sanjay Rampal, Sheena E Ramsay, Daniel A Rangel Reina, Ravindra P Rannan-Eliya, Mohammad-Mahdi Rashidi, Josep Redon, Jane DP Renner, Cézane P Reuter, Luis Revilla, Negar Rezaei, Abbas Rezaianzadeh, Yeunsook Rho, Fernando Rigo, Leanne M Riley, Ulf Risérus, Reina G Roa, Louise Robinson, Wendy E Rodríguez-Anderson, Fernando Rodríguez-Artalejo, María del Cristo Rodriguez-Perez, Laura A Rodríguez-Villamizar, Andrea Y Rodríguez, Ulla Roggenbuck, Peter Rohloff, Rosalba Rojas-Martinez, Elisabetta L Romeo, Annika Rosengren, Joel GR Roy, Adolfo Rubinstein, Maria Ruiz-Castell, Paola Russo, Petra Rust, Marcin Rutkowski, Charumathi Sabanayagam, Hamideh Sabbaghi, Harshpal S Sachdev, Alireza Sadjadi, Ali Reza Safarpour, Sare Safi, Saeid Safiri, Mohammad Hossien Saghi, Olfa Saidi, Satoko Sakata, Nader Saki, Sanja Šalaj, Benoit Salanave, Jukka T Salonen, Massimo Salvetti, Jose Sánchez-Abanto, Diana A Santos, Lèlita C Santos, Maria Paula Santos, Rute Santos, Tamara R Santos, Jouko L Saramies, Luis B Sardinha, Nizal Sarrafzadegan, Yoko Sato, Kai-Uwe Saum, Stefan Savin, Norie Sawada, Mariana Sbaraini, Marcia Scazufca, Beatriz D Schaan, Herman Schargrodsky, Christa Scheidt-Nave, Sabine Schipf, Amand Floriaan Schmidt, Börge Schmidt, Carsten O Schmidt, Peter Schnohr, Catherine Mary Schooling, Ben Schöttker, Sara Schramm, Sylvain Sebert, Moslem Sedaghattalab, Aye Aye Sein, Abhijit Sen, Sadaf G Sepanlou, Jennifer Servais, Ronel Sewpaul, Svetlana Shalnova, Seyed Morteza Shamshirgaran, Coimbatore Subramaniam Shanthirani, Maryam Sharafkhah, Sanjib K Sharma, Almaz Sharman, Jonathan E Shaw, Amaneh Shayanrad, Ali Akbar Shayesteh, Kenji Shibuya, Hana Shimizu-Furusawa, Rahman Shiri, Marat Shoranov, Namuna Shrestha, Khairil Si-Ramlee, Alfonso Siani, Mark J Siedner, Diego Augusto Santos Silva, Xueling Sim, Mary Simon, Judith Simons, Leon A Simons, Michael Sjöström, Jolanta Slowikowska-Hilczer, Przemysław Slusarczyk, Liam Smeeth, Eugène Sobngwi, Stefan Söderberg, Agustinus Soemantri, Vincenzo Solfrizzi, Mohammad Hossein Somi, Aïcha Soumaré, Alfonso Sousa-Poza, Mafalda Sousa-Uva, Karen Sparrenberger, Jan A Staessen, Andreas Stang, Bill Stavreski, Jostein Steene-Johannessen, Peter Stehle, Aryeh D Stein, Jochanan Stessman, Jakub Stokwiszewski, Karien Stronks, Milton F Suarez-Ortegón, Phalakorn Suebsamran, Machi Suka, Chien-An Sun, Jianping Sun, Johan Sundström, Paibul Suriyawongpaisal, René Charles Sylva, E Shyong Tai, Furusawa Takuro, Abdonas Tamosiunas, Eng Joo Tan, Baimakhan Tanabayev, Nikhil Tandon, Mohammed Rasoul Tarawneh, Carolina B Tarqui-Mamani, Anne Taylor, Tania Tello, Yih Chung Tham, KR Thankappan, Holger Theobald, Xenophon Theodoridis, Nihal Thomas, Amanda G Thrift, Erik J Timmermans, Hanna K Tolonen, Janne S Tolstrup, Maciej Tomaszewski, Murat Topbas, Michael J Tornaritis, Maties Torrent, Laura Torres-Collado, Giota Touloumi, Pierre Traissac, Areti Triantafyllou, Oanh TH Trinh, Yu-Hsiang Tsao, Thomas Tsiampalis, Shoichiro Tsugane, John Tuitele, Azaliia M Tuliakova, Marshall K Tulloch-Reid, Tomi-Pekka Tuomainen, Maria L Turley, Evangelia Tzala, Christophe Tzourio, Peter Ueda, Eunice Ugel, Flora AM Ukoli, Hanno Ulmer, Hannu MT Uusitalo, Gonzalo Valdivia, Damaskini Valvi, Rob M van Dam, Bert-Jan van den Born, Johan Van der Heyden, Hoang Van Minh, Lenie van Rossem, Natasja M Van Schoor, Irene GM van Valkengoed, Dirk Vanderschueren, Diego Vanuzzo, Anette Varbo, Senthil K Vasan, Tomas Vega, Toomas Veidebaum, Gustavo Velasquez-Melendez, Charlotte Verdot, Giovanni Veronesi, Roosmarijn Verstraeten, Cesar G Victora, Lucie Viet, Luis Villarroel, Jesus Vioque, Jyrki K Virtanen, Bharathi Viswanathan, Peter Vollenweider, Ari Voutilainen, Martine Vrijheid, Alisha N Wade, Janette Walton, Wan Mohamad Wan Bebakar, Wan Nazaimoon Wan Mohamud, Chongjian Wang, Huijun Wang, Ningli Wang, Qian Wang, Weiqing Wang, Ya Xing Wang, Yi-Ren Wang, Ying-Wei Wang, S Goya Wannamethee, Karen Webster-Kerr, Niels Wedderkopp, Wenbin Wei, Leo D Westbury, Peter H Whincup, Kurt Widhalm, Indah S Widyahening, Andrzej Więcek, Nilmini Wijemunige, Rainford J Wilks, Karin Willeit, Peter Willeit, Tom Wilsgaard, Bogdan Wojtyniak, Roy A Wong-McClure, Andrew Wong, Emily B Wong, Mark Woodward, Chao-Chun Wu, Frederick C Wu, Haiquan Xu, Liang Xu, Yu Xu, Nor Azwany Yaacob, Li Yan, Weili Yan, Tabara Yasuharu, Chao-Yu Yeh, Moein Yoosefi, Akihiro Yoshihara, San-Lin You, Novie O Younger-Coleman, Yu-Ling Yu, Ahmad Faudzi Yusoff, Ahmad A Zainuddin, Farhad Zamani, Sabina Zambon, Antonis Zampelas, Abdul Hamid Zargar, Ko Ko Zaw, Tomasz Zdrojewski, Tajana Zeljkovic Vrkic, Yi Zeng, Bing Zhang, Lei Zhang, Luxia Zhang, Zhen-Yu Zhang, Ming-Hui Zhao, Wenhua Zhao, Bekbolat Zholdin, Paul Zimmet, Marie Zins, Emanuel Zitt, Nada Zoghlami, Julio Zuñiga Cisneros

## Abstract

**Background:**

Diabetes can be detected at the primary health-care level, and effective treatments lower the risk of complications. There are insufficient data on the coverage of treatment for diabetes and how it has changed. We estimated trends from 1990 to 2022 in diabetes prevalence and treatment for 200 countries and territories.

**Methods:**

We used data from 1108 population-representative studies with 141 million participants aged 18 years and older with measurements of fasting glucose and glycated haemoglobin (HbA_1c_), and information on diabetes treatment. We defined diabetes as having a fasting plasma glucose (FPG) of 7·0 mmol/L or higher, having an HbA_1c_ of 6·5% or higher, or taking medication for diabetes. We defined diabetes treatment as the proportion of people with diabetes who were taking medication for diabetes. We analysed the data in a Bayesian hierarchical meta-regression model to estimate diabetes prevalence and treatment.

**Findings:**

In 2022, an estimated 828 million (95% credible interval [CrI] 757–908) adults (those aged 18 years and older) had diabetes, an increase of 630 million (554–713) from 1990. From 1990 to 2022, the age-standardised prevalence of diabetes increased in 131 countries for women and in 155 countries for men with a posterior probability of more than 0·80. The largest increases were in low-income and middle-income countries in southeast Asia (eg, Malaysia), south Asia (eg, Pakistan), the Middle East and north Africa (eg, Egypt), and Latin America and the Caribbean (eg, Jamaica, Trinidad and Tobago, and Costa Rica). Age-standardised prevalence neither increased nor decreased with a posterior probability of more than 0·80 in some countries in western and central Europe, sub-Saharan Africa, east Asia and the Pacific, Canada, and some Pacific island nations where prevalence was already high in 1990; it decreased with a posterior probability of more than 0·80 in women in Japan, Spain, and France, and in men in Nauru. The lowest prevalence in the world in 2022 was in western Europe and east Africa for both sexes, and in Japan and Canada for women, and the highest prevalence in the world in 2022 was in countries in Polynesia and Micronesia, some countries in the Caribbean and the Middle East and north Africa, as well as Pakistan and Malaysia. In 2022, 445 million (95% CrI 401–496) adults aged 30 years or older with diabetes did not receive treatment (59% of adults aged 30 years or older with diabetes), 3·5 times the number in 1990. From 1990 to 2022, diabetes treatment coverage increased in 118 countries for women and 98 countries for men with a posterior probability of more than 0·80. The largest improvement in treatment coverage was in some countries from central and western Europe and Latin America (Mexico, Colombia, Chile, and Costa Rica), Canada, South Korea, Russia, Seychelles, and Jordan. There was no increase in treatment coverage in most countries in sub-Saharan Africa; the Caribbean; Pacific island nations; and south, southeast, and central Asia. In 2022, age-standardised treatment coverage was lowest in countries in sub-Saharan Africa and south Asia, and treatment coverage was less than 10% in some African countries. Treatment coverage was 55% or higher in South Korea, many high-income western countries, and some countries in central and eastern Europe (eg, Poland, Czechia, and Russia), Latin America (eg, Costa Rica, Chile, and Mexico), and the Middle East and north Africa (eg, Jordan, Qatar, and Kuwait).

**Interpretation:**

In most countries, especially in low-income and middle-income countries, diabetes treatment has not increased at all or has not increased sufficiently in comparison with the rise in prevalence. The burden of diabetes and untreated diabetes is increasingly borne by low-income and middle-income countries. The expansion of health insurance and primary health care should be accompanied with diabetes programmes that realign and resource health services to enhance the early detection and effective treatment of diabetes.

**Funding:**

UK Medical Research Council, UK Research and Innovation (Research England), and US Centers for Disease Control and Prevention.

## Introduction

Diabetes increases the risk of debilitating complications such as amputation, vision loss, and renal failure, and is associated with cardiovascular disease, dementia, some cancers, and infections such as tuberculosis and severe COVID-19. Diabetes can be detected at the primary health-care level, and treatment with oral hypoglycaemic drugs or insulin, as well as newer injectable medicines, reduces the risk of, and slows progression to, complications and sequelae.^1,2^ Failure to treat or delay in treatment increases the risk of complications and death. Therefore, diabetes prevalence and diabetes treatment coverage, and how they have changed, are important measures of population health and the performance of health systems.

Obesity, which is an important risk factor for diabetes, has increased in most countries in the past few decades, with the largest increases seen in low-income and middle-income countries.^3^ At the same time, several effective medicines for diabetes (eg, metformin) are now off patent and available at a relatively low cost in most countries,^4,5^ and pharmacological treatment is recommended alongside diet and lifestyle change to manage diabetes.^1,6–9^ Comparable data on diabetes prevalence and treatment coverage across countries can help identify good practices in the prevention and treatment of diabetes and guide health system priorities and programmes. Current global data on diabetes prevalence estimates have several limitations, and there are few data on the coverage of treatment for diabetes, and especially on how it has changed. We present global estimates of trends in diabetes prevalence and treatment from 1990 to 2022.

## Methods

### Overview

We pooled population-based studies with measurements of fasting glucose and glycated haemoglobin (HbA_1c_). Pooled data were analysed using a Bayesian hierarchical meta-regression model. Our primary outcomes were the prevalence of diabetes and the proportion of people with diabetes who were treated for diabetes. Diabetes was defined as taking medication for diabetes (treated diabetes), or having a fasting plasma glucose (FPG) of 7·0 mmol/L or more or an HbA_1c_ of 6·5% or more (untreated diabetes). Treatment was defined as the current use of oral hypoglycaemic drugs or insulin in people with diabetes.

We estimated trends from 1990 to 2022 in the primary outcomes for 200 countries and territories, which were divided into 20 regions and eight super-regions (appendix p 69). We estimated diabetes prevalence for women and men aged 18 years and older, and treatment for those aged 30 years and older. We used different age groups for prevalence and treatment because the relatively small number of participants with diabetes aged 18–29 years results in frequent zero or very small denominators and unstable estimates of treatment in these age ranges. All analyses were performed in R (version 4.3.1).

### Data

We pooled population-based studies with measurements of fasting glucose and HbA_1c_ from a database collated by the NCD Risk Factor Collaboration (NCD-RisC). Details of data sources, cleaning, and management are provided in the appendix (pp 27–32). We used 1108 studies conducted from 1980 to 2022 with 141 million participants aged 18 years or older (appendix pp 49–68). These studies measured fasting glucose, HbA_1c_, or both, as described in the appendix (pp 27*–*32). We had at least one study for 175 (88%) of the 200 countries for which estimates were made (appendix pp 74*–*75). Countries in the high-income western super-region (with an average of 12·3 studies per country) and the east and southeast Asia and the Pacific super-region (with an average of 11·5 studies per country) had the most data, and those in sub-Saharan Africa (1·8 studies per country) and Pacific island nations (2·8 studies per country) had the least data. Other super-regions had on average from 3·2 to 10·4 studies per country (appendix pp 76–77). 833 (75%) of these studies had data on treatment and the other 275 (25%) did not. The studies without treatment data were of two types: those that did not collect treatment data or did not separate pharmacological and lifestyle interventions, and the studies that were extracted from reports or obtained via a previous global data pooling study as detailed in the appendix (pp 27–32).

### Statistical methods for analysis of pooled data

We used a Bayesian hierarchical meta-regression model to estimate trends in diabetes prevalence and treatment by sex, age, country, and year. The statistical methods are detailed in previous publications^3,10^ and in the appendix (pp 33–47).

Age-standardised prevalence and treatment were calculated by weighting age-specific estimates using the WHO standard population. When calculating age-standardised treatment, we also accounted for the age pattern of diabetes prevalence by multiplying the WHO standard population weights with age-specific diabetes prevalence in each country and year, because the denominator of treatment was only people with diabetes.

All calculations were done at the posterior draw level. The reported 95% credible intervals (CrIs) represent the 2·5–97·5th percentiles of the posterior distributions. We obtained the posterior probability that an estimated change represented a true increase as the proportion of draws from the posterior distribution that had an increase. The results section presents the number of countries where prevalence or treatment changed with a posterior probability of more than 0·80, as an indication of probable changes in population health (prevalence) and health system response (treatment). The number of countries with an increase or decrease would be fewer if a stricter threshold (eg, 0·90) were used.

We decomposed the change over time in the number of people with untreated diabetes to calculate the contributions of three drivers: change in population size and age structure, change in prevalence, and change in treatment coverage. Decomposition was done using the basic algebraic relationship of these components (appendix p 48).

### Secondary analysis of diabetes diagnosis

In a secondary analysis, we used data from studies conducted from 2005 to 2022 that were representative of countries as a whole or one or more subnational regions. We used data from participants aged 30 years or older to calculate the proportion of those with untreated diabetes who did not receive a diagnosis, accounting for sample weights as stated in the appendix (pp 27–32). We combined data across studies through weighting by their respective sample sizes for eight super-regions, presented in the appendix (p 69). We did this secondary analysis to evaluate whether the extent of under-treatment of diabetes was due to under-diagnosis, or due to under-treatment among those with a diagnosis. Consistent with the previous NCD-RisC analysis of hypertension,^10^ we only considered participants who answered yes to the diagnosis question as having (diagnosed) diabetes if they used treatment or had elevated FPG or HbA_1c_ (ie, those who were not using treatment and had below-threshold levels of FPG and HbA_1c_ were not counted as having diabetes). We did this so that we did not overestimate the prevalence or extent of diagnosis, because in some studies the question used to elicit diagnosis information combined diabetes with prediabetes or past gestational diabetes.

### Role of the funding source

The funders of the study had no role in study design, data collection, data analysis, data interpretation, or writing of the report.

## Results

**Figure 1:**
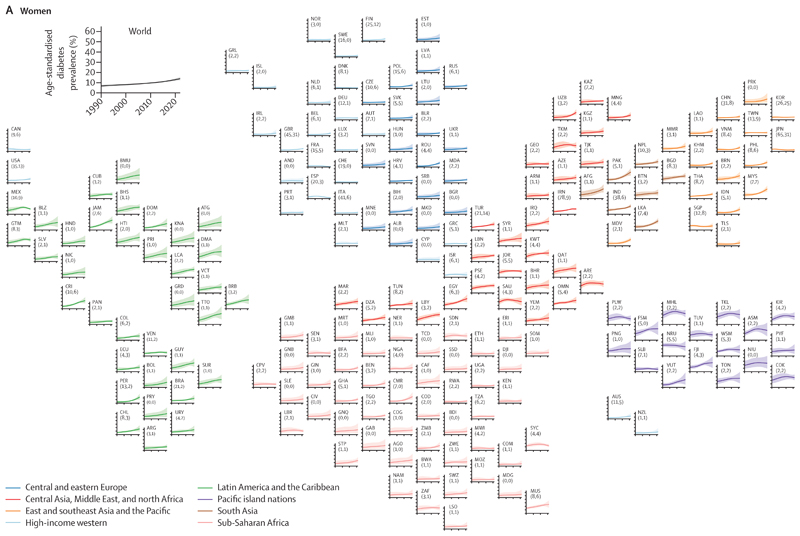

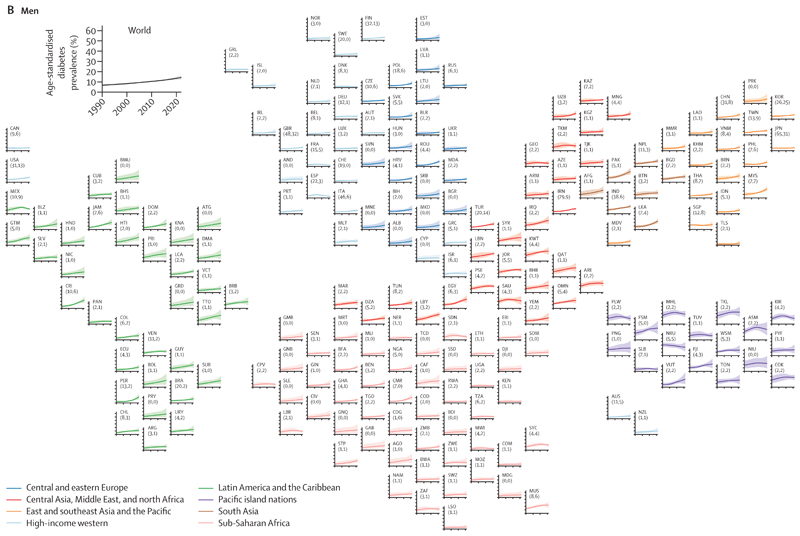
Age-standardised diabetes prevalence from 1990 to 2022 by country, for women and men aged 18 years or older The shaded areas around the lines show the 95% credible intervals of the estimates. Countries are labelled using their International Organization for Standardization 3166–1 α-3 codes. The numbers in brackets after each country’s label show the total number of data sources and the number of nationally representative data sources, respectively. See the appendix (pp 90–91) for the results for both sexes combined. AFG=Afghanistan. AGO=Angola. ALB=Albania. AND=Andorra. ARE=United Arab Emirates. ARG=Argentina. ARM=Armenia. ASM=American Samoa. ATG=Antigua and Barbuda. AUS=Australia. AUT=Austria. AZE=Azerbaijan. BDI=Burundi. BEL=Belgium. BEN=Benin. BFA=Burkina Faso. BGD=Bangladesh. BGR=Bulgaria. BHR=Bahrain. BHS=The Bahamas. BIH=Bosnia and Herzegovina. BLR=Belarus. BLZ=Belize. BMU=Bermuda. BOL=Bolivia. BRA=Brazil. BRB=Barbados. BRN=Brunei. BTN=Bhutan. BWA=Botswana. CAF=Central African Republic. CAN=Canada. CHE=Switzerland. CHL=Chile. CHN=China. CIV=Côte d’Ivoire. CMR=Cameroon. COD=DR Congo. COG=Congo (Brazzaville). COK=Cook Islands. COL=Colombia. COM=Comoros. CPV=Cabo Verde. CRI=Costa Rica. CUB=Cuba. CYP=Cyprus. CZE=Czechia. DEU=Germany. DJI=Djibouti. DMA=Dominica. DNK=Denmark. DOM=Dominican Republic. DZA=Algeria. ECU=Ecuador. EGY=Egypt. ERI=Eritrea. ESP=Spain. EST=Estonia. ETH=Ethiopia. FIN=Finland. FJI=Fiji. FRA=France. FSM=Federated States of Micronesia. GAB=Gabon. GBR=UK. GEO=Georgia. GHA=Ghana. GIN=Guinea. GMB=The Gambia. GNB=Guinea-Bissau. GNQ=Equatorial Guinea. GRC=Greece. GRD=Grenada. GRL=Greenland. GTM=Guatemala. GUY=Guyana. HND=Honduras. HRV=Croatia. HTI=Haiti. HUN=Hungary. IDN=Indonesia. IND=India. IRL=Ireland. IRN=Iran. IRQ=Iraq. ISL=Iceland. ISR=Israel. ITA=Italy. JAM=Jamaica. JOR=Jordan. JPN=Japan. KAZ=Kazakhstan. KEN=Kenya. KGZ=Kyrgyzstan. KHM=Cambodia. KIR=Kiribati. KNA=Saint Kitts and Nevis. KOR=South Korea. KWT=Kuwait. LAO=Laos. LBN=Lebanon. LBR=Liberia. LBY=Libya. LCA=Saint Lucia. LKA=Sri Lanka. LSO=Lesotho. LTU=Lithuania. LUX=Luxembourg. LVA=Latvia. MAR=Morocco. MDA=Moldova. MDG=Madagascar. MDV=Maldives. MEX=Mexico. MHL=Marshall Islands. MKD=North Macedonia. MLI=Mali. MLT=Malta. MMR=Myanmar. MNE=Montenegro. MNG=Mongolia. MOZ=Mozambique. MRT=Mauritania. MUS=Mauritius. MWI=Malawi. MYS=Malaysia. NAM=Namibia. NER=Niger. NGA=Nigeria. NIC=Nicaragua. NIU=Niue. NLD=Netherlands. NOR=Norway. NPL=Nepal. NRU=Nauru. NZL=New Zealand. OMN=Oman. PAK=Pakistan. PAN=Panama. PER=Peru. PHL=Philippines. PLW=Palau. PNG=Papua New Guinea. POL=Poland. PRI=Puerto Rico. PRK=North Korea. PRT=Portugal. PRY=Paraguay. PSE=Palestine. PYF=French Polynesia. QAT=Qatar. ROU=Romania. RUS=Russia. RWA=Rwanda. SAU=Saudi Arabia. SDN=Sudan. SEN=Senegal. SGP=Singapore. SLB=Solomon Islands. SLE=Sierra Leone. SLV=El Salvador. SOM=Somalia. SRB=Serbia. SSD=South Sudan. STP=São Tomé and Príncipe. SUR=Suriname. SVK=Slovakia. SVN=Slovenia. SWE=Sweden. SWZ=Eswatini. SYC=Seychelles. SYR=Syria. TCD=Chad. TGO=Togo. THA=Thailand. TJK=Tajikistan. TKL=Tokelau. TKM=Turkmenistan. TLS=Timor-Leste. TON=Tonga. TTO=Trinidad and Tobago. TUN=Tunisia. TUR=Türkiye. TUV=Tuvalu. TWN=Taiwan. TZA=Tanzania. UGA=Uganda. UKR=Ukraine. URY=Uruguay. USA=USA. UZB=Uzbekistan. VCT=Saint Vincent and the Grenadines. VEN=Venezuela. VNM=Viet Nam. VUT=Vanuatu. WSM=Samoa. YEM=Yemen. ZAF=South Africa. ZMB=Zambia. ZWE=Zimbabwe.

The results of this study can be explored using visualisations and downloaded from the NCD-RisC website. In 1990, diabetes prevalence was lowest throughout Europe compared with countries in other super-regions for both sexes and in sub-Saharan Africa for men (figure 1). The age-standardised prevalence of diabetes was 2–4% in 16 countries for women and in 22 for men, with Denmark having the lowest prevalence for both sexes, followed by Sweden for women and Rwanda for men. The age-standardised prevalence was 20–31% in some Pacific island nations (Nauru, Marshall Islands, Tokelau, and Cook Islands for both sexes, and Samoa for men) in 1990.

**Figure 2:**
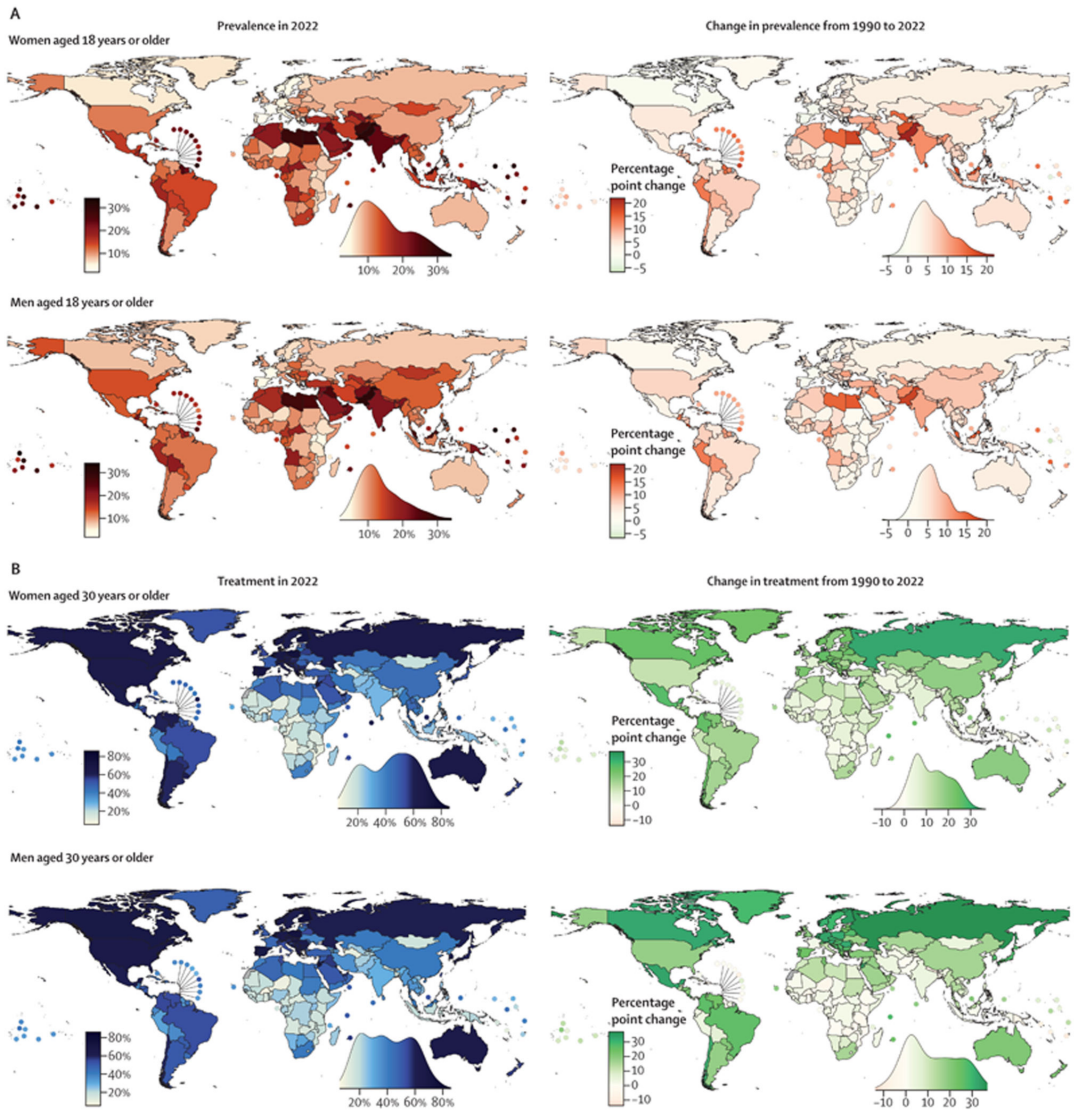
Age-standardised diabetes prevalence and treatment coverage in 2022 and change from 1990 to 2022 (A) Age-standardised diabetes prevalence in people aged 18 years or older. (B) Age-standardised treatment coverage in people aged 30 years or older. The density plot alongside each map shows the smoothed distribution of estimates across countries. The appendix (pp 78–81) shows the posterior probability of an estimated increase or decrease being a true increase or decrease. The appendix (pp 92–93) also shows the results for both sexes combined.

Age-standardised prevalence decreased from 1990 to 2022 with a posterior probability of more than 0·80 by 1–2 percentage points in women in Japan, Spain, and France,^11–13^ countries where women also had a declining or flat trend in obesity over this period,^3^ and by 7 percentage points in men in Nauru, albeit from a high 1990 prevalence of 30·7% (95% CrI 25·4–36·1; figures 1 and 2). Neither an increase nor a decrease in prevalence was detected at a posterior probability of 0·80 among women in 66 countries and men in 44 countries. Most of these countries were in western Europe (eg, Denmark and the Netherlands), sub-Saharan Africa (eg, Ethiopia and Malawi), east Asia and the Pacific (eg, Singapore), and Canada, as well as many island nations in Polynesia and Micronesia, where prevalence was already high in 1990. In the other 131 countries for women and 155 for men, age-standardised diabetes prevalence increased with a posterior probability of more than 0·80. The increase ranged 2–22 percentage points in women and 2–18 in men. The countries with the smallest increase were in Europe and sub-Saharan Africa (where many other countries did not have an increase with a posterior probability of more than 0·80) and Timor-Leste, as well as Japan (in men, with women having a decline as stated earlier). The largest increases, ranging 15–22 percentage points, was in a belt of countries north of the equator extending from southeast Asia (eg, Malaysia), to south Asia (eg, Pakistan), the Middle East and north Africa (eg, Egypt), and Latin America and the Caribbean (eg, Jamaica, Trinidad and Tobago, and Costa Rica). Prevalence also increased by a large amount in other Pacific islands such as Federated States of Micronesia and Vanuatu. In relative terms, age-standardised prevalence more than doubled in 66 countries for women and 79 for men from 1990 to 2022 (appendix pp 82–83).

Diabetes prevalence in 2022 was still lowest in western Europe and east Africa for both sexes, and in Japan and Canada for women. The age-standardised prevalence in 2022 was 2–4% for women in France, Denmark, Spain, Switzerland, and Sweden, and 3–5% for men in Denmark, France, Uganda, Kenya, Malawi, Spain, and Rwanda (figure 2). At the other end, the age-standardised prevalence among women in 21 countries and men in 14 countries surpassed 25% in 2022. These high-prevalence countries included most countries in Polynesia and Micronesia, some countries in the Caribbean (eg, Trinidad and Tobago, and Jamaica) and in the Middle East and north Africa (eg, Egypt), Pakistan, and Malaysia.^14,15^

In 2022, the crude prevalence was higher than age-standardised in high-income western countries, central and eastern Europe, east Asia and the Pacific, and parts of Latin America and the Caribbean (appendix pp 86–87). It was lower in sub-Saharan Africa, south Asia, and most countries in southeast Asia and central Asia, and the Middle East and north Africa. This difference occurs because of a combination of two factors: prevalence of diabetes increases with age, and the age of the actual population is older than that of the WHO standard population in high-income western countries, central and eastern Europe, east Asia and the Pacific, and parts of Latin America and the Caribbean, and younger in sub-Saharan Africa, south Asia, and most countries in southeast Asia and central Asia, and the Middle East and north Africa. The median age of people with diabetes was younger than 40 years in some countries in sub-Saharan Africa, and 40–45 years elsewhere in Africa and in some countries in central Asia, south Asia, the Middle East and north Africa, and the Caribbean; in most countries in the high-income western super-region and some countries in east Asia and the Pacific, the median age was older than 65 years (appendix pp 88–89). In 2022, diabetes prevalence was higher in men than women in most high-income western countries (appendix pp 84–85). It was the opposite in most countries in sub-Saharan Africa and Latin America and the Caribbean. In other super-regions, diabetes prevalence was similar or there was a mix of higher and lower female prevalence across countries.

**Figure 3:**
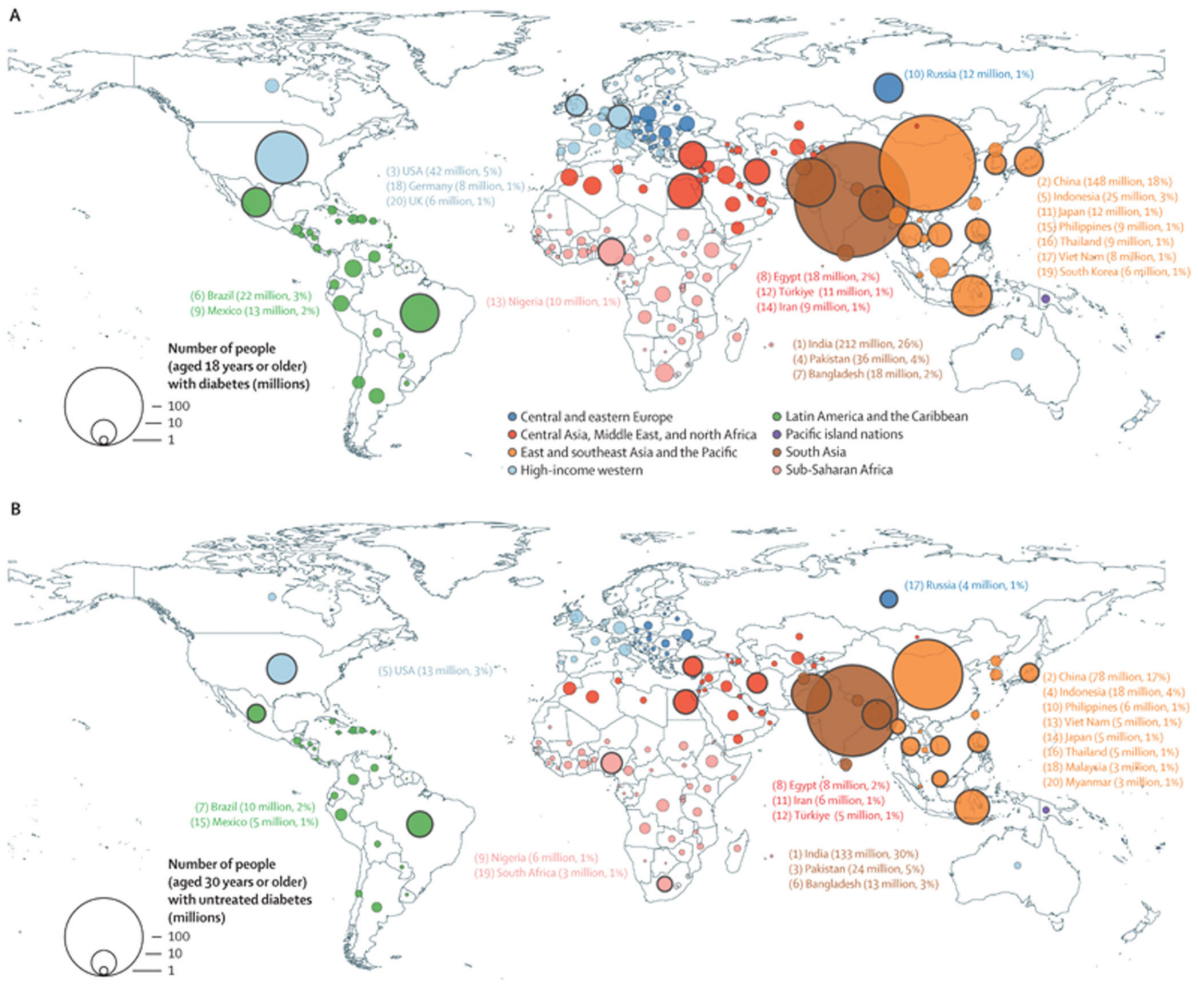
Number of people with (A) diabetes and (B) untreated diabetes in 2022 The number of people with diabetes is for people aged 18 years or older; the number of people with diabetes covering only those aged 30 years or older is shown in the appendix (p 70). The number of people with untreated diabetes is for people aged 30 years or older. The area of the circle is proportional to the number of people with diabetes or untreated diabetes in each country. Countries listed were the top 20 countries in 2022 in terms of number of people with diabetes or untreated diabetes, with their rankings shown by the numbers before their names. The numbers in brackets after each country name show the number of people with diabetes or untreated diabetes in that country, and its percentage of the global number (828 million for diabetes and 445 million for untreated diabetes).

In 2022, age-standardised diabetes prevalence in the world was 13·9% (95% CrI 12·3–15·8) for women and 14·3% (12·5–16·4) for men. An estimated 828 million (95% CrI 757–908) adults had diabetes, a substantial increase of 630 million (554–713) from 1990 (figure 3; appendix p 70). Of these, 420 million (372–473) were women and 408 million (356–467) were men. India (212 million, 175–250) and China (148 million, 103–202) accounted for the largest proportion of this number, followed by the USA, Pakistan, Indonesia, and Brazil.

In 1990, age-standardised diabetes treatment coverage was less than 50% in all except 11 countries for women and one for men. Age-standardised treatment coverage increased in 118 countries (59%) for women and 98 (49%) for men with a posterior probability of more than 0·80 from 1990 to 2022. Some of the largest improvements (25–37 percentage points) were observed in countries in Latin America (ie, Mexico, Colombia, Chile, Costa Rica, and Brazil), central and western Europe (eg, Netherlands, Finland, and Croatia), Canada, South Korea, Russia, Seychelles, and Jordan for one or both sexes (figure 2). Neither an increase nor a decrease was detected in treatment at a posterior probability of 0·80 in 82 countries for women and 101 for men, including most countries in sub-Saharan Africa; the Caribbean; south, southeast, and central Asia; and Pacific island nations. Age-standardised treatment coverage declined slightly in men in Jamaica with a posterior probability of more than 0·80.

**Figure 4:**
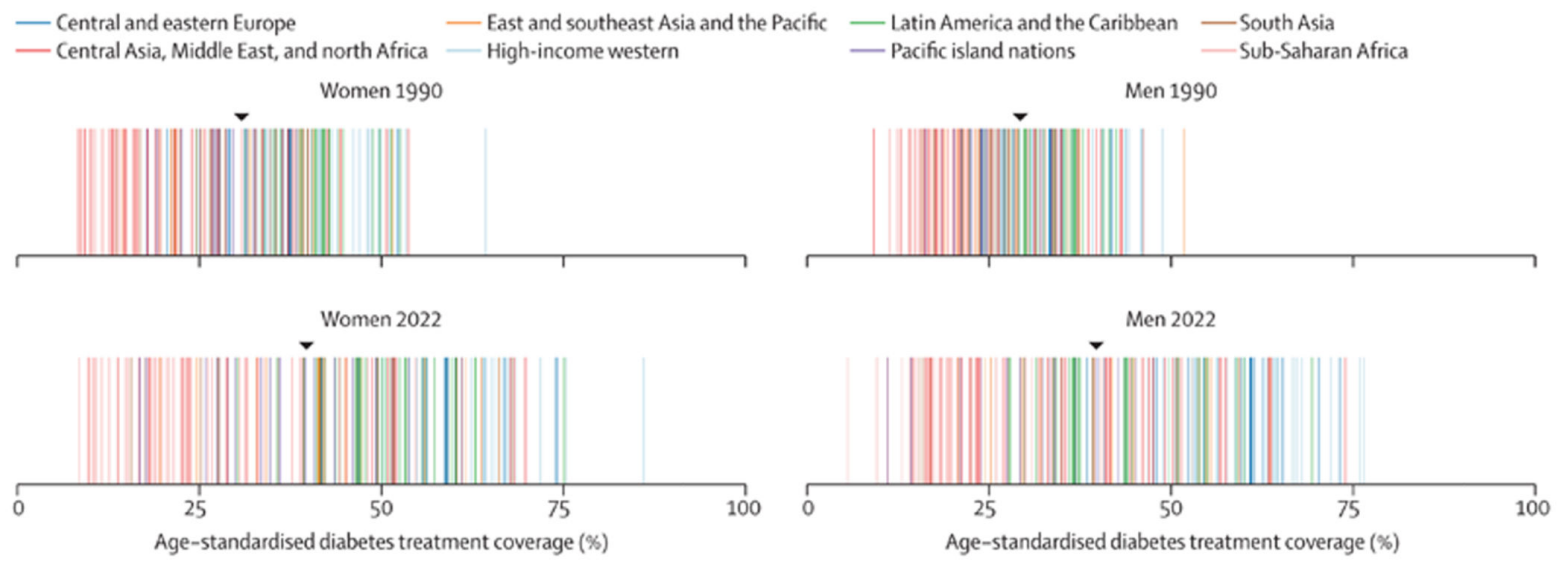
Age-standardised treatment coverage for women and men aged 30 years or older in 1990 and 2022 Each line represents a country, with countries coloured by the super-region in which they fall. The black triangle shows the age-standardised treatment coverage for the world. The appendix (p 69) shows the countries in each super-region. The appendix (pp 94–95) also shows the results for both sexes combined.

As a result of these trends, the gap between the countries with the highest and lowest treatment coverage widened from 56 and 43 percentage points in women and men, respectively, in 1990, to 78 and 71 percentage points in women and men, respectively, in 2022 (figure 4). The difference in treatment coverage also increased between the high-income western super-region, where treatment coverage was highest, and Pacific island nations, south Asia, and sub-Saharan Africa, where it was lowest. In 2022, age-standardised treatment coverage was 5–10% in a few countries in sub-Saharan Africa (eg, Burkina Faso, Angola, Niger, Liberia, and Benin) for one or both sexes. Treatment coverage was less than 25% in Haiti, most countries in sub-Saharan Africa, and some countries in Melanesia (ie, Vanuatu, Solomon Islands, Papua New Guinea, and Fiji), central Asia (ie, Turkmenistan, Mongolia, and Tajikistan), south Asia (eg, Bangladesh, Bhutan, and Nepal),^16^ and southeast Asia (ie, Timor-Leste, Indonesia, and the Philippines) for one or both sexes. Treatment was 55% or higher in South Korea, many high-income western countries, and some countries in central and eastern Europe (eg, Poland, Czechia, and Russia), Latin America (eg, Costa Rica,^17^ Chile, and Mexico), and the Middle East and north Africa (eg, Jordan,^18^ Qatar, and Kuwait). The highest age-standardised treatment coverage was 86% (95% CrI 74–95) for women and 77% (60–89) for men in Belgium (figure 2). Crude and age-standardised treatment estimates were similar (appendix pp 86–87). In 2022, diabetes treatment coverage was higher in women than men in most high-income western countries and countries in Latin America and the Caribbean (appendix pp 84–85). It was the opposite in most countries in sub-Saharan Africa. In other super-regions, treatment was similar between women and men, or there were some countries with higher treatment in women than men and vice versa.

**Figure 5:**
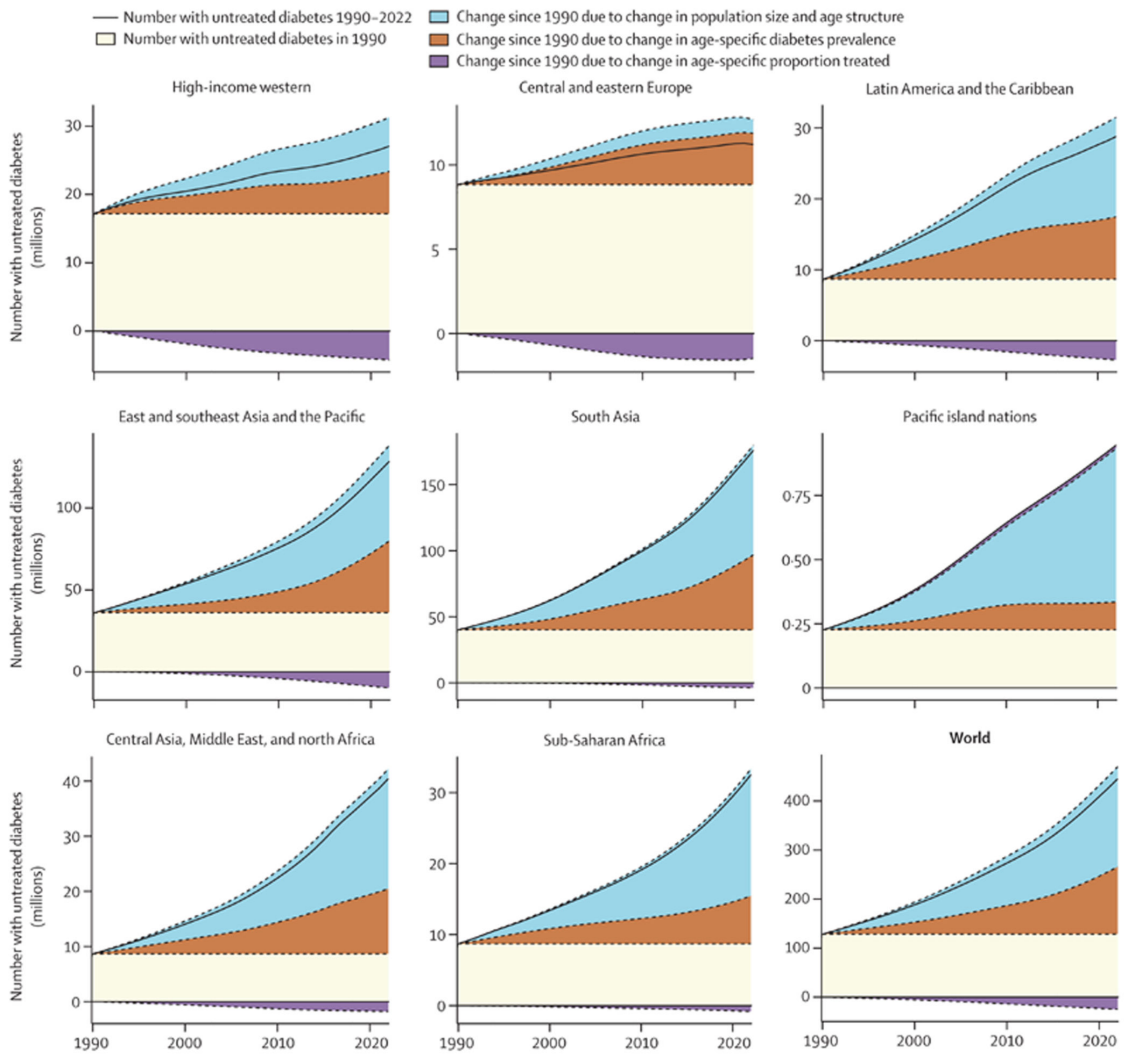
Contributions of population growth and ageing, rise in diabetes prevalence, and improvement in treatment coverage to the change in the number of people with untreated diabetes from 1990 to 2022 The sum of all sections in each graph is the total number of people with untreated diabetes over time, also shown by the solid black line. The area above the solid black line and top of stacked sections, where it is present, equals the contribution of improvement in treatment (ie, the purple section) towards reducing the number of people with untreated diabetes—namely, the top of the stacked sections shows how many people would have had untreated diabetes had treatment coverage not improved. The appendix (p 69) describes the countries in each super-region.

A total of 445 million (95% CrI 401–496) adults aged 30 years or older with diabetes were not treated with oral hypoglycaemic drugs nor insulin (59% of those with diabetes), 3·5 times the number in 1990 (129 million, 112–147; figure 5). 30% of those with untreated diabetes (133 million, 109–160) were in India, more than 50% greater than the next largest number of people with untreated diabetes, which was in China (78 million, 51–112; figure 3), because treatment coverage was higher in China than in India. Similarly, Pakistan (24 million, 16–31) and Indonesia (18 million, 12–25), the countries with the next two largest number of untreated diabetes, had a higher number of people with untreated diabetes than the USA (13 million, 9–18), which had higher treatment coverage.

The number of people with untreated diabetes increased in every super-region from 1990 to 2022 (figure 5). The largest contributor to this rise was the increase in the size and age of population, because diabetes is more prevalent in older age groups. The exception was central and eastern Europe, where the population size grew less than in other super-regions or decreased in some countries (eg, Romania and Bulgaria). In other super-regions, population change accounted for 61–83% of the increase in untreated diabetes cases, and rise in diabetes prevalence accounted for 15–42% of the increase in untreated diabetes cases (figure 5). In high-income western countries, followed by central and eastern Europe, Latin America, and east Asia and the Pacific, the rise in the number of people with diabetes was countered to some extent by the improvement in treatment (figure 5).

In every super-region, most people (84–97%) with untreated diabetes had not received a diagnosis (table). In sub-Saharan Africa and south Asia, more than 94% of untreated diabetes cases were undiagnosed.

## Discussion

Our results show an expanding inequity of diabetes in the world: the largest increases in prevalence have occurred in low-income and middle-income countries, whereas the improvements in treatment were largest in high-income and industrialised nations in Europe, north America, Australasia, and the Pacific, and some well performing middle-income nations and emerging economies, especially those in Latin America. These trends have widened the global gap in diabetes prevalence and treatment, with an increasing share of people with diabetes, especially with untreated diabetes, living in low-income and middle-income countries.

The estimated prevalence of diabetes in our work is higher than other recent global studies^19,20^ (appendix p 71). Beyond methodological differences in how data were pooled and analysed, there are three key reasons for this difference: first, we included people whose FPG or HbA_1c_ were elevated whereas previous studies mostly relied on elevated FPG or other single-biomarker data.^19,20^ Our approach to defining diabetes is consistent with current clinical guidelines and practice,^6–9,21–24^ and avoids leaving out people whose HbA_1c_ is elevated but have an FPG of less than 7·0 mmol/L. This inclusiveness has a larger effect on prevalence in south Asia, because compared with using both FPG and HbA_1c_, FPG alone misses more cases of diabetes in south Asia than other regions.^25–27^ Second, this difference might arise from whether and how self-report or registry data were used: such data were not used in our analyses because some people do not have a previous diagnosis, and hence are neither aware of their diabetes nor appear in a registry, and used in previous studies as described in those studies^19,20^ and summarised earlier in this paper. Third, data used in our analysis were more recent than those in other global analyses (appendix pp 72–73). With rising prevalence, newer data better captures contemporary prevalence. For example, both Sun and colleagues^19^ and the GBD Diabetes Collaborators^20^ used data from early (before 2015) rounds of the ICMR-INDIAB surveys from India, which only covered a small number of states, mostly in low-prevalence regions of the country, whereas we used all six rounds up to 2020, which covered the entire nation and a more recent time period,^26^ as well as an additional national survey from 2018.

Our prevalence estimates are consistent with country studies that used both fasting glucose and HbA_1c_.^26,28–32^ For example, our estimated prevalence for India is similar to that of the ICMR-INDIAB surveys^26^ when both blood glucose and HbA_1c_ are used, which is higher than prevalence based on glucose alone. National surveys from other countries in south Asia, for example Pakistan and Sri Lanka,^14,33^ also show high prevalence when using both FPG and HbA_1c_. This occurs because studies from south Asian countries have consistently found many people with diabetes present with isolated elevated HbA_1c_.^25–27,34^ Our results that diabetes prevalence did not increase, or increased only by a small amount, in some high-income countries, are consistent with reports from Spain, France, Switzerland, Germany, Sweden, Japan, and Taiwan, which showed flat or decreasing trends in prevalence in one or both sexes.^11–13,35–38^ These results are also consistent with evidence on the decline in diabetes incidence in high-income countries,^39^ because prevalence depends on the combination of incidence and survival of those with diabetes.

Our study presented comprehensive, consistent, and comparable global estimates of diabetes prevalence and treatment over a period in which risk factors such as obesity changed substantially, and the benefits of treatment were established and incorporated in clinical guidelines. A key strength of this work is the scale and quality of data, which were checked and harmonised in a rigorous and systematic process. We used data from more than 1100 studies in 175 countries, covering 98% of the world’s population. We used only data from studies that had measured glycaemic markers to avoid bias and other errors in self-reported and registry data due to undiagnosed cases. We reanalysed data according to a standardised protocol, and the characteristics and quality of data in relation to sampling and measurement methods were verified through repeated checks. We defined diabetes on the basis of both FPG and HbA_1c_, bringing global estimates of diabetes into harmony with contemporary clinical guidelines.^6–9,21–24^ This remedies the limitation of previous global estimates in terms of excluding people who have isolated elevated HbA_1c_, who make up a larger proportion of total diabetes in low-income and middle-income countries than high-income countries.^25^ We used a statistical model that accounted for important features of diabetes prevalence and treatment data, including non-linear time trends and age associations and difference in trends between younger and older adults, and gave more weight to national data than to non-national sources. The model fitted the data well, with median difference (which measures bias) between the model fit and national data that covered entire countries being 0·1 percentage points for diabetes prevalence, and –0·8 and –0·6 percentage points for treatment for women and men, respectively. Median absolute difference (which measures non-systematic or random deviation) was 1·5 and 1·7 percentage points for diabetes prevalence, and 6·1 and 5·9 percentage points for treatment for women and men, respectively.

Our study has limitations that affect global analyses that pool data collected in different countries and time periods. Some countries had fewer data and 25 had no data; the estimates for countries with fewer or no data were informed to a stronger degree by data from other countries through geographical hierarchy, which increased the uncertainty of their estimates. Nonetheless, the estimated global prevalence and treatment coverage are virtually identical between using countries with data versus using all countries. Health survey participation rate varies across countries and can change over time, and has become particularly low in Europe. Survey sample weights adjust for non-response but this adjustment might be imperfect, especially as response rates decrease. This issue shows the need to improve the public trust and motivation for participation in health surveys, and to consider alternative surveillance platforms such as routine primary care data, where access and use is high. Although we accounted for whether a study had measured fasting glucose in plasma or whole blood, other unobserved differences might persist due to differing methods—for example, in assays used for measuring FPG and HbA_1c_. The other differences are captured by study-specific random-effect terms in our model that adjust for unobserved differences across studies.

We did not include the 2-h postprandial glucose from an oral glucose tolerance test (2hOGTT) in our definition of diabetes. This is because its use in contemporary clinical practice and population surveillance is rare, due to the inconvenience related to the glucose load, 2-h time frame, and the two blood draws required for the test. If some people with an elevated 2hOGTT are not captured by the combination of FPG and HbA_1c_, the actual prevalence of diabetes would be higher than reported here. HbA_1c_ can be affected by some non-glycaemic factors, including anaemia due to iron deficiency or malaria, specific haemoglobin variants (eg, HbS and HbF), other haemoglobinopathies, polycythaemia due to living in high altitude, liver and kidney diseases, HIV, and specific drugs such as antiretroviral therapy for HIV. Some of these factors, including malaria-induced and iron deficiency anaemia, haemoglobinopathies such as sickle cell disease and thalassemia, and antiretroviral therapy, are more prevalent in parts of Asia and Africa than other parts of the world, and might have affected HbA_1c_ or its measurement. In clinical practice, diabetes diagnosis for those with these conditions might need to be accompanied by a glucose test before decisions about treatment or further testing are made.^6–9,21–24^ Furthermore, in studies that did not measure HbA_1c_, the prevalence of isolated elevated HbA_1c_ was predicted with equations that used the relationship between HbA_1c_, FPG, and other predictors in studies that had measured both (and vice versa for FPG when it was not measured).^25^ Although these equations performed well in cross-validation,^25^ their use increases the uncertainty of our estimates. Most survey data did not separate type 1 and type 2 diabetes in adults because distinguishing between these disorders can be challenging in adults. However, most (85–95%) cases of diabetes in adults are type 2.^40^ Most health surveys are based on one visit to participants, in which a glycaemic marker is measured. Many clinical guidelines recommend using a second confirmatory test for diabetes diagnosis and initiating treatment,^6,21–23^ although there is variation in this guidance. A key reason for a confirmatory test is to minimise risks of erroneous results—for example, due to the misrecording of laboratory results—potentially leading to a lifelong misdiagnosis for an individual patient. This is not a relevant issue in prevalence studies in a population, and hence population surveillance uses a single measurement. Nonetheless, diabetes prevalence based on data collected in multiple visits might be lower, and treatment coverage might be higher, than that based on one measurement. The COVID-19 pandemic might have impeded the rolling out of some health surveys or affected participation. The pandemic might also have affected diabetes prevalence and especially treatment coverage. We used 45 studies from 2020 and later, but additional data are needed to evaluate the population-level effects. We had insufficient comparable data on treatment details such as the use of insulin and specific medicines because these data are not consistently collected in population-representative surveys. Complementing survey data with data from health facilities or prescriptions could provide such clinically relevant details, which is especially important as new effective diabetes medicines become available. Future studies should complement our work on adults with estimates of diabetes prevalence and treatment in children and adolescents, noting that blood-based measurements in health surveys are less common in children and adolescents than in adults.

An important driver of the rise in diabetes, and its variation across countries, is obesity.^3^ Diabetes was either already high or increased more in some of the regions where obesity was or became prevalent, such as Pacific and Caribbean island nations and the Middle East and north Africa, compared with high-income countries in Europe and east Asia and the Pacific, where obesity and diabetes did not rise or rose by a relatively small amount.^3^ In addition to obesity, the consumption of specific foods might influence the risk of diabetes.^41^ For example, yogurt and possibly some other forms of dairy, whole grains, and green leafy vegetables reduce the risk of diabetes,^42–44^ whereas refined carbohydrates, including in sugar-sweetened beverages, increase this risk.^41,45–47^ In countries with universal health insurance and good access to primary care, people at a high risk of diabetes might also be identified early and advised to use a combination of diet and lifestyle modifications and medicines to prevent or delay diabetes onset.^48–55^ This approach is less widely used in low-resourced health systems with limited attention to, or resources for, diabetes screening. Genetic and phenotypic differences due to foetal and childhood nutrition and growth also influence worldwide variations, especially when accompanied with rapid weight gain—for example the high diabetes prevalence in south Asia.^56,57^

Given the disabling and fatal consequences of diabetes, preventing its onset and delaying its complications through improving diet and treatment are essential for better population health throughout the world. Curbing and reversing the rise in obesity, and improving the quality of diet, require both regulations and taxes to reduce the intake of foods such as refined carbohydrates that lead to weight gain, and improving financial and physical access to healthier foods, such as fresh fruits, vegetables, legumes, dairy and fish, and to sports and active leisure.^3^ Improving affordability and accessibility of healthy foods and sports is particularly important for poorer families and marginalised communities, and requires measures such as targeted cash transfers, subsidies or vouchers for healthy foods and sports facilities, and free healthy school meals.^3^ Alongside these population-based measures, diet and lifestyle modification and medicines in people at a high risk of diabetes (including those with prediabetes) can prevent or delay diabetes onset and reduce the risk of some complications.^48–55^ High-risk prevention requires identifying such people, and supporting them to achieve sustained changes in diet and exercise and to use effective medicines. This in turn requires health systems that facilitate regular contact with primary care providers, screening, access to healthy foods and pharmacological interventions, and monitoring and follow-ups to ensure compliance and to further adjust interventions.

For people who already have diabetes, lifestyle modification and pharmacological treatment, as well as blood pressure and cholesterol control, can delay progression to complications and reduce mortality, or even help with remission, especially if started early.^1,2,58–62^ Our results show that treatment coverage varies substantially globally, and is low in most low-income and middle-income countries. We also found that the current variations in treatment were largely related to the extent of diabetes under-diagnosis, which means that improving case detection is a prerequisite to increasing treatment coverage. Improving the detection of diabetes requires facilitating regular contact with health services and care providers as well as programmes that enhance screening. Universal insurance is essential for higher care access and use but, to improve diagnosis of diabetes, it should be accompanied with expanding as well as redesigning primary care towards diabetes and other long-term conditions that are initially asymptomatic.^63^ Such actions are especially important in regions with younger populations and lower average age of diabetes, because health system contact tends to be lower for young adults. Adaptation of primary care for more screening requires approaches such as extended or flexible hours for people in employment, workplace and community screening programmes and campaigns, integration with screening and care for diseases such as HIV and AIDS and tuberculosis, which have well established programmes, and the use of non-physician health providers.^17,61,63–71^ Increasing diagnosis also requires guidelines, training, and decision support tools to encourage diabetes screening, and the availability of laboratory facilities or point-of-care monitors. To improve treatment coverage, diagnosed diabetes cases need financial and physical access to a regular supply of medicines, information and support to adhere to treatment, and follow-up visits to evaluate control and screen for complications. Universal insurance is also important for treatment but should be accompanied with the inclusion of diabetes medicines as essential medicines covered through insurance; national or multi-country strategies for accessing medicines at lower costs, as done for HIV and AIDS; an efficient procurement and distribution system that ensures an uninterrupted supply of medicine, guidelines, training, and decision support to encourage and facilitate prescription; and information and support systems via digital technologies or community health workers to improve patient adherence.^61,64,69,72,73^ These factors show the need to go beyond universal insurance alone, and to have national diabetes programmes that leverage and adapt universal insurance and primary care expansion to address the burden of diabetes.^17,63,64,74^ With new diabetes medicines and technologies such as continuous glucose monitoring now available in high-income countries, such actions are particularly essential to avoid widening the global inequalities in diabetes burden and treatment.

## Supplementary Material

Appendix

## Figures and Tables

**Table:** Proportion of people with untreated diabetes who had not received a diagnosis, using data from studies conducted in 2005–22

	Number of studies	Median year of studies (range)	Proportion of people with untreated diabetes who were undiagnosed	
			Women	Men
Central and eastern Europe	21	2014 (2008–21)	84·4%	86·0%
Central Asia, the Middle East, and north Africa	62	2013 (2005–22)	89·1%	89·9%
East and southeast Asia and the Pacific	54	2014 (2005–22)	90·4%	90·3%
High-income western	92	2012 (2005–21)	84·0%	84·5%
Latin America and the Caribbean	53	2014 (2005–22)	88·8%	88·5%
Pacific island nations	22	2011 (2005–22)	87·3%	86·3%
South Asia	27	2015 (2006–21)	97·0%	95·1%
Sub-Saharan Africa	50	2014 (2007–22)	94·2%	94·2%

Data from 2005–22 were used because there were more data available in this time period and the data were more recent. There is no credible interval for the proportion of people with untreated diabetes who were undiagnosed, because these numbers were obtained using a weighted average across studies as stated in the methods. The countries in each super-region are described in the appendix (p 69).

## Research in context

### Evidence before this study

We searched MEDLINE (via PubMed) for articles published from database inception up to Aug 14, 2024, with no language restrictions, using the following search terms: (“Diabetes Mellitus”[MAJR:NoExp] OR “Diabetes Mellitus, Type 2”[MAJR:NoExp] OR “Diabetes Mellitus, Type 1”[MAJR:NoExp] OR “Blood Glucose”[MAJR] OR “Glycated Hemoglobin”[MAJR]) AND (“Health Surveys”[mh] OR “Epidemiological Monitoring”[mh] OR “Cross-Sectional Studies”[mh] OR “Prevalence”[mh]) AND “Humans”[mh] NOT “patient*”[Title] NOT Comment[ptyp] NOT Case Reports[ptyp]. Articles were screened to include measured data on blood glucose or glycated haemoglobin, collected from samples of national, subnational, or community populations aged 18 years and older.

Many studies reported diabetes prevalence and treatment for adults in a single country or a single region. Many of these found an increase in diabetes prevalence. However, studies in Spain, France, Switzerland, Germany, Sweden, Japan, and Taiwan found flat or decreasing trends in prevalence in one or both sexes. We found four studies that reported diabetes prevalence or treatment in multiple low-income and middle-income countries directly from surveys or other community-based samples. These studies did not report trends over time, nor did they account for differences in age groups included in surveys.

A few studies reported diabetes prevalence for multiple world regions or globally for different time periods, mostly before 2015. We found two global studies that reported diabetes prevalence beyond 2015. One study used population-based data that had measured a glycaemic biomarker such as FPG and HbA_1c_ as well as data sources that had no measurement and relied only on self-reported diabetes diagnosis (eg, the Canadian Community Health Survey and European Health Interview Surveys) or registry data (eg, a diabetes registry in Russia). The self-reported data were adjusted to approximate total diabetes prevalence on the basis of the average diagnosis rate in the country, if available, or region where the data came from. The other global study used population-based data that had measured glycaemic biomarkers such as FPG and HbA_1c_ as well as registry data (eg, New Zealand Virtual Diabetes Register and Australia National Diabetes Register); it did not make any adjustments to registry data to account for the underestimation of prevalence due to undiagnosed diabetes. These studies either reported diabetes prevalence on the basis of a single glycaemic marker (most commonly FPG), and hence did not include people with isolated elevated levels of other glycaemic markers such as HbA_1c_, or they did not standardise the definition of diabetes across studies. No study reported diabetes treatment coverage for all countries in the world.

### Added value of this study

To our knowledge, this study is the first global analysis of trends in both diabetes prevalence and treatment coverage that covers all countries. We reanalysed and pooled hundreds of population-representative studies with measurements of glycaemic biomarkers and data on diabetes treatment. We used a diabetes definition that included both FPG and HbA_1c_, thus closing a major gap between global health statistics and clinical practice and guidelines.

### Implications of all the available evidence

Since 1990, the largest increase in diabetes prevalence has occurred in low-income and middle-income countries, whereas the improvements in treatment were largest in high-income and industrialised nations and some emerging economies, especially in Latin America. These trends have widened the global gap in diabetes prevalence and treatment, with an increasing share of people with diabetes, especially with untreated diabetes, living in low-income and middle-income countries.

## Data sharing

The input data are available at https://www.ncdrisc.org and Zenodo (https://doi.org/10.5281/zenodo.13987398), where permitted by data governance and sharing arrangements; contact information are provided for other data sources in the same file. The computer code is also available from the same sites. The results of this study can be downloaded from https://www.ncdrisc.org.
