# Supplementary material for: Worldwide trends in diabetes prevalence and treatment from 1990 to 2022: a pooled analysis of 1108 population-representative studies with 141 million participants
Source: Lancet. Author manuscript; Available in PMC 2024 Nov 21. (PMC7616842; doi:10.1016/S0140-6736(24)02317-1)
Supplement: Appendix [file EMS200082-supplement-Appendix.pdf]

THE LANCET

Supplementary appendix

This appendix formed part of the original submission and has been peer reviewed. We post it as supplied by the authors.

Supplement to: NCD Risk Factor Collaboration (NCD-RisC). Worldwide trends in diabetes prevalence and treatment from 1990 to 2022: a pooled analysis of 1108 population-representative studies with 141 million participants. *Lancet* 2024; published online Nov 13. https://doi.org/10.1016/S0140-6736(24)02317-1.

Table of Contents

Appendix Text 1. NCD Risk Factor Collaboration (NCD-RisC)... 2

Appendix Text 2. Data sources.. 27

Appendix Text 3. Statistical methods... 33

Appendix Text 4. Decomposition of change in the number of people with untreated diabetes. 48

Appendix Table 1. Data sources used in the analysis. 49

Appendix Table 2. List of analysis regions and super-regions, and countries in each region. 69

Appendix Table 3. Global age-standardised diabetes prevalence and number of people with diabetes in 1990 and 2022 for women and men aged 18+ years and 30+ years. 70

Appendix Table 4. Comparison of age-standardised diabetes prevalence estimates in this work with recent global studies. 71

Appendix Table 5. Comparison of data sources and estimates of diabetes prevalence in this work with recent global studies, for the 30 countries with the largest adult population in 2022. 72

Appendix Figure 1. Number of data sources used in this analysis, by country.................... 74

Appendix Figure 2. Number of data sources used in this paper, by region and year. 76

Appendix Figure 3. Posterior probability that age-standardised diabetes prevalence for people aged 18+ years increased from 1990 to 2022. 78

Appendix Figure 4. Posterior probability that age-standardised diabetes treatment coverage for people aged 30+ years increased from 1990 to 2022. 80

Appendix Figure 5. Relationship between levels in 1990 and 2022 for age-standardised diabetes prevalence for people aged 18+ years and age-standardised treatment coverage for people aged 30+ years. 82

Appendix Figure 6. Relationship of age-standardised diabetes prevalence and treatment coverage between women and men in 2022. 84

Appendix Figure 7. Relationship between age-standardised and crude prevalence of diabetes among women and men aged 18+ years in 2022.. 86

Appendix Figure 8. Median age of women and men with diabetes in 2022 by country. 88

Appendix Figure 9. Age-standardised diabetes prevalence for people aged 18+ years from 1990 to 2022 by country, for both sexes combined. 90

Appendix Figure 10. Levels in 2022 and change from 1990 to 2022 by country of (A) age-standardised diabetes prevalence for people aged 18+ years, and (B) age-standardised treatment coverage for people aged 30+ years, for both sexes combined. 92

Appendix Figure 11. Age-standardised treatment coverage for people aged 30+ years in 1990 and 2022, for both sexes combined. 94

References.. 96

Appendix Text 1. NCD Risk Factor Collaboration (NCD-RisC)

Pooled Analysis and Writing (* equal contribution; joint first authors)

Bin Zhou (PhD; Imperial College London, UK)*; Archie W Rayner (MSci; Imperial College London, UK)*; Prof Edward W Gregg (PhD; RCSI University of Medicine and Health Sciences, Ireland; Imperial College London, UK); Kate E Sheffer (MSc; Imperial College London, UK); Rodrigo M Carrillo-Larco (MD; Emory University, USA); James E Bennett (PhD; Imperial College London, UK); Prof Jonathan E Shaw (MD; Baker Heart and Diabetes Institute, Australia); Prof Christopher J Paciorek (PhD; University of California, Berkeley, USA); Rosie K Singleton (MSc; Imperial College London, UK); Ana Barradas Pires (MD; Imperial College London, UK); Gretchen A Stevens (ScD; World Health Organization, Switzerland); Prof Goodarz Danaei (ScD; Harvard TH Chan School of Public Health, USA); Victor PF Lhoste (MSc; Imperial College London, UK); Nowell H Phelps (MMath; Johns Hopkins Bloomberg School of Public Health, USA); Rachel A Heap (MPH; Imperial College London, UK); Lakshya Jain (MSc; Imperial College London, UK); Ysé d'Ailhaud de Brisis (MSc; Imperial College London, UK); Agnese Galeazzi (MSci; Imperial College London, UK); Prof Andre P Kengne (PhD; South African Medical Research Council, South Africa); Anu Mishra (PhD; Bill & Melinda Gates Foundation, USA); Nayu Ikeda (PhD; National Institutes of Biomedical Innovation, Health and Nutrition, Japan); Prof Hsien-Ho Lin (MD; National Taiwan University, Taiwan); Prof Carlos A Aguilar-Salinas (DSc; Instituto Nacional de Ciencias Médicas y Nutrición, Mexico); Ranjit Mohan Anjana (PhD; Madras Diabetes Research Foundation, India); Prof Habiba Ben Romdhane (MD; University Tunis El Manar, Tunisia); Prof Kairat Davletov (PhD; Asfendiyarov Kazakh National Medical University, Kazakhstan); Shubash Ganapathy (MPH; Ministry of Health, Malaysia); Christin Heidemann (DrPH; Robert Koch Institute, Germany); Prof Yousef Saleh Khader (ScD; Jordan University of Science and Technology, Jordan); Prof Young-Ho Khang (MD; Seoul National University College of Medicine, Republic of Korea); Prof Avula Laxmaiah (PhD; ICMR - National Institute of Nutrition, India); Prof Jean Claude N Mbanya (PhD; University of Yaoundé 1, Cameroon); Viswanathan Mohan (MD; Madras Diabetes Research Foundation, India); Prof KM Venkat Narayan (MD; Emory University, USA);

Meda E Pavkov (PhD; Centres for Disease Control and Prevention, USA); Prof Eugène Sobngwi (MD; University of Yaoundé 1, Cameroon); Alisha N Wade (DPhil; University of the Witwatersrand, South Africa); Novie O Younger-Coleman (PhD; The University of the West Indies, Jamaica); Prof Tomasz Zdrojewski (MD; Medical University of Gdansk, Poland); Prof Majid Ezzati (PhD; Imperial College London, UK; University of Ghana, Ghana)

Country and Regional Data (* equal contribution; listed alphabetically)

Leandra Abarca-Gómez (Caja Costarricense de Seguro Social, Costa Rica)*; Mohsen Abbasi-Kangevari (Non-Communicable Diseases Research Center, Iran)*; Hanan F Abdul Rahim (Qatar University, Qatar)*; Niveen M Abu-Rmeileh (Birzeit University, State of Palestine)*; Shalkar Adambekov (Al-Farabi Kazakh National University, Kazakhstan; Academy of Preventive Medicine, Kazakhstan)*; Robert J Adams (Flinders University, Australia)*; Wichai Aekplakorn (Mahidol University, Thailand)*; Shoaib Afzal (Copenhagen University Hospital - Herlev and Gentofte, Denmark; University of Copenhagen, Denmark)*; Imelda A Agdeppa (Food and Nutrition Research Institute, Philippines)*; Javad Aghazadeh-Attari (Urmia University of Medical Sciences, Iran)*; Carlos A Aguilar-Salinas (Instituto Nacional de Ciencias Médicas y Nutrición, Mexico)*; Charles Agyemang (University of Amsterdam, The Netherlands)*; Noor Ani Ahmad (Ministry of Health, Malaysia)*; Ali Ahmadi (Shahrekord University of Medical Sciences, Iran)*; Naser Ahmadi (Non-Communicable Diseases Research Center, Iran)*; Nastaran Ahmadi (Shahid Sadoughi University of Medical Sciences, Iran)*; Soheir H Ahmed (University of Hargeisa, Somaliland)*; Wolfgang Ahrens (Leibniz Institute for Prevention Research and Epidemiology - BIPS, Germany)*; Kamel Ajlouni (National Center for Diabetes, Endocrinology and Genetics, Jordan)*; Sarah F Al-Hamli (Kuwait Institute for Scientific Research, Kuwait)*; Halima Al-Hinai (Ministry of Health, Oman)*; Jawad A Al-Lawati (Ministry of Health, Oman)*; Deena Al Asfoor (World Health Organization Regional Office for the Eastern Mediterranean, Egypt)*; Monira Alarouj (Dasman Diabetes Institute, Kuwait)*; Fadia AlBuhairan (Aldara Hospital and Medical Center, Saudi Arabia)*; Shahla AlDhukair (King Abdullah International Medical Research Center, Saudi Arabia)*;

Mohamed M Ali (World Health Organization, Switzerland)*; Mohammed K Ali (Emory University, USA)*; Anna V Alieva (Ministry of Health, Uzbekistan)*; Farbod Alinezhad (Northeastern University, USA)*; Abdullah Alkandari (Dasman Diabetes Institute, Kuwait)*; Ala'a Alkerwi (Directorate of Health, Luxembourg)*; Eman Aly (World Health Organization Regional Office for the Eastern Mediterranean, Egypt)*; Deepak N Amarapurkar (Bombay Hospital and Medical Research Centre, India)*; Lars Bo Andersen (Western Norway University of Applied Sciences, Norway)*; Sigmund A Anderssen (Norwegian School of Sport Sciences, Norway)*; Dolores S Andrade (Universidad de Cuenca, Ecuador)*; Ranjit Mohan Anjana (Madras Diabetes Research Foundation, India)*; Alireza Ansari-Moghaddam (Health Promotion Research Center, Iran)*; Hajer Aounallah-Skhiri (National Institute of Public Health, Tunisia)*; Joana Araújo (University of Porto, Portugal)*; Tahir Aris (Ministry of Health, Malaysia)*; Raphael E Arku (University of Massachusetts Amherst, USA)*; Nimmathota Arlappa (ICMR - National Institute of Nutrition, India)*; Krishna K Aryal (University of Bergen, Norway)*; Thor Aspelund (University of Iceland, Iceland)*; Felix K Assah (University of Yaoundé 1, Cameroon)*; Batyrbek Assembekov (Asfendiyarov Kazakh National Medical University, Kazakhstan)*; Shiu Lun Au Yeung (The University of Hong Kong, China)*; Juha Auvinen (Oulu University Hospital, Finland; University of Oulu, Finland)*; Mária Avdičová (Regional Authority of Public Health, Slovakia)*; Kishwar Azad (Diabetic Association of Bangladesh, Bangladesh)*; Ana Azevedo (University of Porto, Portugal)*; Mohsen Azimi-Nezhad (Neyshabur University of Medical Sciences, Iran)*; Fereidoun Azizi (Research Institute for Endocrine Sciences, Iran)*; Flora Bacopoulou (National and Kapodistrian University of Athens, Greece)*; Nagalla Balakrishna (ICMR - National Institute of Nutrition, India)*; Yulia Balanova (National Medical Research Centre for Therapy and Preventive Medicine, Russia)*; Mohamed Bamoshmoosh (University of Science and Technology, Yemen)*; Maciej Banach (The John Paul II Catholic University of Lublin, Poland)*; Piotr Bandosz (Medical University of Gdańsk, Poland)*; José R Banegas (CIBERESP, Spain; Universidad Autónoma de Madrid, Spain)*; Carlo M Barbagallo (University of Palermo, Italy)*; Alberto Barcelo (University of Miami, USA)*; Maja Baretić (University Hospital Centre Zagreb,

Croatia)*; Lena Barrera (Universidad del Valle, Colombia)*; Marta Barreto (National Institute of Health Doutor Ricardo Jorge, Portugal; NOVA University Lisbon, Portugal)*; Abdul Basit (Diabetic Association of Pakistan, Pakistan; Health Promotion Foundation, Pakistan)*; Anwar M Batieha (Jordan University of Science and Technology, Jordan)*; Aline P Batista (Universidade Federal de Ouro Preto, Brazil)*; Louise A Baur (University of Sydney, Australia)*; Antonisamy Belavendra (Christian Medical College Vellore, India)*; Habiba Ben Romdhane (University Tunis El Manar, Tunisia)*; Theodora Benedek (University of Medicine, Pharmacy, Sciences and Technology of Târgu-Mureș, Romania)*; Mikhail Benet (Cafam University Foundation, Colombia)*; Michaela Benzeval (University of Essex, UK)*; Salim Berkinbayev (Kazakh National Medical University, Kazakhstan)*; Antonio Bernabe-Ortiz (Universidad Científica del Sur, Peru)*; Ximena Berrios Carrasola (Pontificia Universidad Católica de Chile, Chile)*; Heloísa Bettiol (University of São Paulo, Brazil)*; Augustin F Beybey (University of Yaoundé 1, Cameroon)*; Santosh K Bhargava (Sunder Lal Jain Hospital, India)*; Yufang Bi (Shanghai Jiao-Tong University School of Medicine, China)*; Elysée Claude Bika Lele (Institute of Medical Research and Medicinal Plant Studies, Cameroon)*; Mukharram M Bikbov (Ufa Eye Research Institute of the Bashkir State Medical University, Russia)*; Bihungum Bista (Nepal Health Research Council, Nepal)*; Peter Bjerregaard (University of Southern Denmark, Denmark)*; Espen Bjertness (University of Oslo, Norway)*; Marius B Bjertness (University of Oslo, Norway)*; Cecilia Björkelund (University of Gothenburg, Sweden)*; Katia V Bloch (Universidade Federal do Rio de Janeiro, Brazil)*; Anneke Blokstra (National Institute for Public Health and the Environment, The Netherlands)*; Martin Bobak (University College London, UK)*; Bernhard O Boehm (Nanyang Technological University, Singapore)*; Jose G Boggia (Universidad de la República, Uruguay)*; Carlos P Boissonnet (Centro de Educación Médica e Investigaciones Clínicas, Argentina)*; Stig E Bojesen (Copenhagen University Hospital - Herlev and Gentofte, Denmark; University of Copenhagen, Denmark)*; Marialaura Bonaccio (IRCCS Neuromed, Italy)*; Alice Bonilla-Vargas (Caja Costarricense de Seguro Social, Costa Rica)*; Herman Borghs (KU Leuven, Belgium)*; Steve Botomba (University of Kinshasa, DR Congo)*; Pascal Bovet (Ministry of Health, Seychelles;

Unisanté, Switzerland)*; Imperia Brajkovich (Universidad Central de Venezuela, Venezuela)*; Hermann Brenner (German Cancer Research Center, Germany)*; Lizzy M Brewster (University of Amsterdam, The Netherlands)*; Garry R Brian (The Fred Hollows Foundation, New Zealand)*; Yajaira Briceño (University of the Andes, Venezuela)*; Miguel Brito (Instituto Politécnico de Lisboa, Portugal)*; Gloria Bueno (University of Zaragoza, Spain)*; Anna Bugge (University College Copenhagen, Denmark)*; Frank Buntinx (KU Leuven, Belgium)*; Antonio Cabrera de León (Universidad de La Laguna, Spain)*; Roberta B Caixeta (Pan American Health Organization, USA)*; Günay Can (Istanbul University, Türkiye)*; Ana Paula C Cândido (Universidade Federal de Juiz de Fora, Brazil)*; Mario V Capanzana (Food and Nutrition Research Institute, Philippines)*; Naděžda Čapková (National Institute of Public Health, Czechia)*; Eduardo Capuano (Canopo Study Center - Salerno, Italy)*; Rocco Capuano (Canopo Study Center - Salerno, Italy)*; Vincenzo Capuano (Canopo Study Center - Salerno, Italy)*; Viviane C Cardoso (University of São Paulo, Brazil)*; Axel C Carlsson (Karolinska Institutet, Sweden)*; Rodrigo M Carrillo-Larco (Emory University, USA)*; Felipe F Casanueva (Santiago de Compostela University, Spain)*; Laura Censi (Council for Agricultural Research and Economics, Italy)*; Marvin Cervantes-Loaiza (Caja Costarricense de Seguro Social, Costa Rica)*; Charalambos A Chadjigeorgiou (Research and Education Institute of Child Health, Cyprus)*; Parinya Chamnan (Sanpasitthiprasong Regional Hospital, Thailand)*; Snehalatha Chamukuttan (India Diabetes Research Foundation, India)*; Queenie Chan (Imperial College London, UK)*; Fadi J Charchar (Federation University Australia, Australia)*; Nish Chaturvedi (University College London, UK)*; Chien-Jen Chen (Academia Sinica, Taiwan)*; Huashuai Chen (Xiangtan University, China)*; Long-Sheng Chen (Ministry of Health and Welfare, Taiwan)*; Ching-Yu Cheng (National University of Singapore, Singapore)*; Bahman Cheraghian (Ahvaz Jundishapur University of Medical Sciences, Iran)*; Angela Chetrit (The Gertner Institute for Epidemiology and Health Policy Research, Israel)*; Shu-Ti Chiou (Ministry of Health and Welfare, Taiwan)*; María-Dolores Chirlaque (CIBERESP, Spain)*; Jerzy Chudek (Medical University of Silesia, Poland)*; Renata Cifkova (Charles University, Czechia; Thomayer University Hospital, Czechia)*; Massimo Cirillo (University of

Salerno, Italy)*; Frank Claessens (KU Leuven, Belgium)*; Janine Clarke (Statistics Canada, Canada)*; Emmanuel Cohen (UMR CNRS-MNHN 7206, France)*; Hans Concin (Agency for Preventive and Social Medicine, Austria)*; Cyrus Cooper (University of Southampton, UK)*; Cojocaru R Cosmin (Carol Davila University of Medicine and Pharmacy, Romania)*; Simona Costanzo (IRCCS Neuromed, Italy)*; Melanie J Cowan (World Health Organization, Switzerland)*; Chris Cowell (University of Sydney, Australia)*; Amelia C Crampin (Malawi Epidemiology and Intervention Research Unit, Malawi)*; Ana B Crujeiras (CIBEROBN, Spain)*; Juan J Cruz (CIBERESP, Spain; Universidad Autónoma de Madrid, Spain)*; Felipe V Cureau (Universidade Federal do Rio Grande do Norte, Brazil)*; Sarah Cuschieri (University of Malta, Malta)*; Graziella D'Arrigo (National Research Council, Italy)*; Eleonora d'Orsi (Universidade Federal de Santa Catarina, Brazil)*; Haroldo da Silva-Ferreira (Universidade Federal de Alagoas, Brazil)*; Jean Dallongeville (Institut Pasteur de Lille, France)*; Albertino Damasceno (Eduardo Mondlane University, Mozambique)*; Rachel Dankner (The Gertner Institute for Epidemiology and Health Policy Research, Israel)*; Saeed Dastgiri (Tabriz Health Services Management Research Center, Iran)*; Luc Dauchet (University of Lille, France; Lille University Hospital, France)*; Kairat Davletov (Asfendiyarov Kazakh National Medical University, Kazakhstan)*; Amalia De Curtis (IRCCS Neuromed, Italy)*; Giovanni de Gaetano (IRCCS Neuromed, Italy)*; Stefaan De Henauw (Ghent University, Belgium)*; David De Ridder (University of Geneva, Switzerland)*; Mohan Deepa (Madras Diabetes Research Foundation, India)*; Vincent Jr DeGennaro (Innovating Health International, Haiti)*; Stefaan Demarest (Sciensano, Belgium)*; Elaine Dennison (University of Southampton, UK)*; Valérie Deschamps (The National Public Health Agency, France)*; Meghnath Dhimal (Nepal Health Research Council, Nepal)*; Zivka Dika (University of Zagreb, Croatia)*; Shirin Djalalinia (Ministry of Health and Medical Education, Iran)*; Chiara Donfrancesco (Istituto Superiore di Sanità, Italy)*; Maria Dorobantu (Carol Davila University of Medicine and Pharmacy, Romania)*; Nico Dragano (University Hospital Düsseldorf, Germany)*; Wojciech Drygas (Medical University of Lodz, Poland; Calisia University, Poland)*; Shufa Du (University of North Carolina at Chapel Hill, USA)*; Yong Du (Robert Koch Institute, Germany)*; Charmaine

A Duante (Food and Nutrition Research Institute, Philippines)*; Priscilla Duboz (IRL 3189 ESS, France)*; Rosemary B Duda (Beth Israel Deaconess Medical Center, USA; Harvard Medical School, USA)*; Anar Dushpanova (Scuola Superiore Sant'Anna, Italy; Al-Farabi Kazakh National University, Kazakhstan)*; Vilnis Dzerve (University of Latvia, Latvia)*; Elzbieta Dziankowska-Zaborszczyk (Medical University of Lodz, Poland)*; Narges Ebrahimi (Non-Communicable Diseases Research Center, Iran)*; Ricky Eddie (Ministry of Health and Medical Services, Solomon Islands)*; Ebrahim Eftekhar (Hormozgan University of Medical Sciences, Iran)*; Vasiliki Efthymiou (National and Kapodistrian University of Athens, Greece)*; Eruke E Egbagbe (University of Benin, Nigeria)*; Robert Eggertsen (University of Gothenburg, Sweden)*; Sareh Eghtesad (Tehran University of Medical Sciences, Iran)*; Clara Ladi Ejembi (Ahmadu Bello University, Nigeria)*; Mohammad El-Khateeb (National Center for Diabetes, Endocrinology and Genetics, Jordan)*; Jalila El Ati (National Institute of Nutrition and Food Technology, Tunisia)*; Denise Eldemire-Shearer (The University of the West Indies, Jamaica)*; Roberto Elosua (Institut Hospital del Mar d'Investigacions Mèdiques, Spain; CIBERCV, Spain)*; Ofem Enang (University of Calabar, Nigeria)*; Rajiv T Erasmus (Stellenbosch University, South Africa)*; Cihangir Erem (Imperial Hospital, Türkiye)*; Gul Ergor (Dokuz Eylul University, Türkiye)*; Louise Eriksen (University of Southern Denmark, Denmark)*; Johan G Eriksson (University of Helsinki, Finland)*; Ali Esmaeili (Rafsanjan University of Medical Sciences, Iran)*; Roger G Evans (Monash University, Australia)*; Guy Fagherazzi (Luxembourg Institute of Health, Luxembourg)*; Noushin Fahimfar (Tehran University of Medical Sciences, Iran)*; Ildar Fakhradiyev (Kazakh National Medical University, Kazakhstan)*; Albina A Fakhretdinova (Ufa Eye Research Institute, Russia)*; Caroline H Fall (University of Southampton, UK)*; Elnaz Faramarzi (Tabriz University of Medical Sciences, Iran)*; Mojtaba Farjam (Fasa University of Medical Sciences, Iran)*; Farshad Farzadfar (World Health Organization, Switzerland)*; Yosef Farzi (Non-Communicable Diseases Research Center, Iran)*; Mohammad Reza Fattahi (Shiraz University of Medical Sciences, Iran)*; Asher Fawwad (Baqai Medical University, Pakistan)*; Francisco J Felix-Redondo (Centro de Salud Villanueva Norte, Spain)*; Trevor S Ferguson (The University of the West Indies, Jamaica)*;

Daniel Fernández-Bergés (Hospital Don Benito-Villanueva de la Serena, Spain)*; Desha R Fernando (University of Colombo, Sri Lanka)*; Thomas Ferrao (Statistics Canada, Canada)*; Marika Ferrari (Council for Agricultural Research and Economics, Italy)*; Marco M Ferrario (University of Insubria, Italy)*; Catterina Ferreccio (Pontificia Universidad Católica de Chile, Chile)*; Eldridge Ferrer (Food and Nutrition Research Institute, Philippines)*; Edith JM Feskens (Wageningen University, The Netherlands)*; Günther Fink (Swiss Tropical and Public Health Institute, Switzerland; University of Basel, Switzerland)*; David Flood (Wuqu' Kawoq, Guatemala)*; Maria Forsner (Umeå University, Sweden)*; Sandrine Fosse-Edorh (French Public Health Agency, France)*; Edward F Fottrell (University College London, UK)*; Heba M Fouad (World Health Organization Regional Office for the Eastern Mediterranean, Egypt)*; Damian K Francis (Georgia College and State University, USA)*; Guillermo Frontera (Hospital Universitario Son Espases, Spain)*; Isti I Fujiati (Universitas Sumatera Utara, Indonesia)*; Matsuda Fumihiko (Kyoto University, Japan)*; Takuro Furusawa (Kyoto University, Japan)*; Zbigniew Gaciong (Medical University of Warsaw, Poland)*; Fabio Galvano (University of Catania, Italy)*; Sarah P Garnett (University of Sydney, Australia)*; Jean-Michel Gaspoz (Geneva University Hospitals, Switzerland)*; Magda Gasull (CIBERESP, Spain; Hospital del Mar Research Institute, Spain)*; Andrea Gazzinelli (Universidade Federal de Minas Gerais, Brazil)*; Ulrike Gehring (Utrecht University, The Netherlands)*; Ebrahim Ghaderi (Kurdistan University of Medical Sciences, Iran)*; Seyyed-Hadi Ghamari (Non-Communicable Diseases Research Center, Iran)*; Ali Ghanbari (Non-Communicable Diseases Research Center, Iran)*; Erfan Ghasemi (Non-Communicable Diseases Research Center, Iran)*; Oana-Florentina Gheorghe-Fronea (Carol Davila University of Medicine and Pharmacy, Romania)*; Anup Ghimire (B P Koirala Institute of Health Sciences, Nepal)*; Alessandro Gialluisi (IRCCS Neuromed, Italy; "Giuseppe Degennaro" LUM University, Italy)*; Simona Giampaoli (Istituto Superiore di Sanità, Italy)*; Francesco Gianfagna (University of Insubria, Italy; Mediterranea Cardiocentro, Italy)*; Tiffany K Gill (University of Adelaide, Australia)*; Jonathan Giovannelli (University of Lille, France; Lille University Hospital, France)*; Glen Gironella (Food and Nutrition Research Institute, Philippines)*; Aleksander Giwercman (Lund University, Sweden)*;

Marcel Goldberg (Institut National de la Santé et de la Recherche Médicale, France; Paris Cité University, France)*; David Goltzman (McGill University, Canada)*; Aleksandra Gomula (PASs Hirszfeld Institute of Immunology and Experimental Therapy, Poland)*; Helen Gonçalves (Universidade Federal de Pelotas, Brazil)*; Mauer Gonçalves (University Agostinho Neto, Angola)*; David A Gonzalez-Chica (University of Adelaide, Australia)*; Marcela Gonzalez-Gross (Universidad Politécnica de Madrid, Spain)*; Juan P González-Rivas (St. Ann's University Hospital, Czechia)*; Angel R Gonzalez (Universidad Autónoma de Santo Domingo, Dominican Republic)*; Atsushi Goto (National Cancer Center, Japan)*; Frederic Gottrand (University of Lille, France)*; Dušan Grafnetter (Institute for Clinical and Experimental Medicine, Czechia)*; Maria G Grammatikopoulou (University of Thessaly, Greece)*; Andriene Grant (Ministry of Health and Wellness, Jamaica)*; Anne Sameline Grimsgaard (UiT the Arctic University of Tromsø, Norway)*; Tomasz Grodzicki (Jagiellonian University Medical College, Poland)*; Anders Grøntved (University of Southern Denmark, Denmark)*; Giuseppe Grosso (University of Catania, Italy)*; Dongfeng Gu (National Center of Cardiovascular Diseases, China)*; Vilmundur Gudnason (University of Iceland, Iceland)*; Ramiro Guerrero (Universidad Icesi, Colombia)*; Idris Guessous (Geneva University Hospitals, Switzerland)*; Unjali P Gujral (Emory University, USA)*; Rajeev Gupta (Eternal Heart Care Centre and Research Institute, India)*; Laura Gutierrez (Institute for Clinical Effectiveness and Health Policy, Argentina)*; Xinyi Gwee (National University of Singapore, Singapore)*; Seongjun Ha (National Health Insurance Service, Republic of Korea)*; Rosa Haghshenas (Non-Communicable Diseases Research Center, Iran)*; Hamid Hakimi (Rafsanjan University of Medical Sciences, Iran)*; Ian R Hambleton (The University of the West Indies, Barbados)*; Behrooz Hamzeh (Kermanshah University of Medical Sciences, Iran)*; Dominique Hange (University of Gothenburg, Sweden)*; Sari Hantunen (University of Eastern Finland, Finland)*; Jie Hao (Capital Medical University Beijing Tongren Hospital, China)*; Javad Harooni (Yasuj University of Medical Sciences, Iran)*; Seyed Mohammad Hashemi-Shahri (Zahedan University of Medical Sciences, Iran)*; Jun Hata (Kyushu University, Japan)*; Alison J Hayes (University of Sydney, Australia)*; Jiang He (Tulane

University, USA)*; Christin Heidemann (Robert Koch Institute, Germany)*; Rafael dos Santos Henrique (Universidade Federal de Pernambuco, Brazil)*; Ana Henriques (University of Porto, Portugal)*; Sauli Herrala (Oulu University Hospital, Finland)*; Karl-Heinz Herzig (University of Oulu, Finland; Oulu University Hospital, Finland)*; Ramin Heshmat (Chronic Diseases Research Center, Iran)*; Allan G Hill (University of Southampton, UK)*; Sai Yin Ho (The University of Hong Kong, China)*; Michelle Holdsworth (French National Research Institute for Sustainable Development, France)*; Reza Homayounfar (Shahid Beheshti University of Medical Sciences, Iran)*; Wilma M Hopman (Kingston Health Sciences Centre, Canada)*; Andrea RVR Horimoto (University of São Paulo, Brazil)*; Claudia Hormiga (Universidad Autónoma de Bucaramanga, Colombia)*; Bernardo L Horta (Universidade Federal de Pelotas, Brazil)*; Leila Houti (University Oran 1, Algeria)*; Christina Howitt (The University of the West Indies, Barbados)*; Thein Thein Htay (Independent Public Health Specialist, Myanmar)*; Aung Soe Htet (UiT The Arctic University of Norway, Norway)*; Maung Maung Than Htike (Ministry of Health and Sports, Myanmar)*; José María Huerta (CIBERESP, Spain)*; Ilpo Tapani Huhtaniemi (Imperial College London, UK)*; Laetitia Huiart (Santé publique France, France)*; Martijn Huisman (Vrije Universiteit Amsterdam, The Netherlands)*; Monica Hunsberger (University of Gothenburg, Sweden)*; Abdullatif Husseini (Birzeit University, State of Palestine)*; Inge Huybrechts (International Agency for Research on Cancer, France)*; Licia Iacoviello (IRCCS Neuromed, Italy; "Giuseppe Degennaro" LUM University, Italy)*; Ellina M Iakupova (Ufa Eye Research Institute, Russia)*; Anna G Iannone (Gaetano Fucito Hospital, Italy)*; Norazizah Ibrahim Wong (Ministry of Health, Malaysia)*; Chinwuba Ijoma (University of Nigeria, Nigeria)*; Nayu Ikeda (National Institutes of Biomedical Innovation, Health and Nutrition, Japan)*; Vilma E Irazola (Institute for Clinical Effectiveness and Health Policy, Argentina)*; Takafumi Ishida (The University of Tokyo, Japan)*; Sheikh Mohammed Shariful Islam (Deakin University, Australia)*; Duygu Islek (Emory University, USA)*; Till Ittermann (University Medicine Greifswald, Germany)*; Masanori Iwasaki (Hokkaido University, Japan)*; Tuija Jääskeläinen (Finnish Institute for Health and Welfare, Finland)*; Jeremy M Jacobs (Hadassah University Medical Center, Israel)*; Hashem Y Jaddou (Jordan University of

Science and Technology, Jordan)*; Michel Jadoul (Université Catholique de Louvain, Belgium)*; Bakary Jallow (Gambia National Nutrition Agency, The Gambia)*; Kenneth James (The University of the West Indies, Jamaica)*; Kazi M Jamil (Kuwait Institute for Scientific Research, Kuwait)*; Edward Janus (University of Melbourne, Australia)*; Marjo-Riitta Jarvelin (Imperial College London, UK; University of Oulu, Finland)*; Grazyna Jasienska (Jagiellonian University Medical College, Poland)*; Ana Jelaković (University Hospital Centre Zagreb, Croatia)*; Bojan Jelaković (University of Zagreb, Croatia)*; Garry Jennings (Heart Foundation, Australia)*; Anjani Kumar Jha (Nepal Health Research Council, Nepal)*; AM Jibo (University of Bisha, Saudi Arabia)*; Ramon O Jimenez (Universidad Eugenio Maria de Hostos, Dominican Republic)*; Karl-Heinz Jöckel (University of Duisburg-Essen, Germany)*; Jari J Jokelainen (Oulu University Hospital, Finland)*; Jost B Jonas (Rothschild Foundation Hospital, France)*; Josipa Josipović (University Hospital Centre Sestre Milosrdnice, Croatia)*; Farahnaz Joukar (Guilan University of Medical Sciences, Iran)*; Jacek Jóźwiak (University of Opole, Poland)*; Anthony Kafatos (University of Crete, Greece)*; Eero O Kajantie (Finnish Institute for Health and Welfare, Finland)*; Zhanna Kalmatayeva (Al-Farabi Kazakh National University, Kazakhstan)*; Ofra Kalter-Leibovici (The Gertner Institute for Epidemiology and Health Policy Research, Israel)*; Argyro Karakosta (National and Kapodistrian University of Athens, Greece)*; Khem B Karki (Maharajgunj Medical Campus, Nepal)*; Marzieh Katibeh (Aarhus University, Denmark)*; Prasad Katulanda (University of Colombo, Sri Lanka)*; Jussi Kauhanen (University of Eastern Finland, Finland)*; Gyulli M Kazakbaeva (Ufa Eye Research Institute, Russia)*; François F Kaze (University of Yaoundé 1, Cameroon)*; Calvin Ke (University of Toronto, Canada)*; Sirkka Keinänen-Kiukaanniemi (Oulu University Hospital, Finland)*; Roya Kelishadi (Isfahan University of Medical Sciences, Iran)*; Andre P Kengne (South African Medical Research Council, South Africa)*; Maryam Keramati (Mashhad University of Medical Sciences, Iran)*; Mathilde Kersting (Research Institute of Child Nutrition, Germany)*; Yousef Saleh Khader (Jordan University of Science and Technology, Jordan)*; Kazem Khalagi (Tehran University of Medical Sciences, Iran)*; Arsalan Khaledifar (Shahrekord University of Medical Sciences, Iran)*; Davood Khalili (Shahid Beheshti

University of Medical Sciences, Iran)*; Young-Ho Khang (Seoul National University College of Medicine, Republic of Korea)*; Bahareh Kheiri (Shahid Beheshti University of Medical Sciences, Iran)*; Motahareh Kheradmand (Mazandaran University of Medical Sciences, Iran)*; Alireza Khosravi Farsani (Hypertension Research Center, Iran)*; Ursula Kiechl-Kohlendorfer (Medical University of Innsbruck, Austria)*; Sophia J Kiechl (VASCage Research Centre on Vascular Ageing and Stroke, Austria)*; Stefan Kiechl (Medical University of Innsbruck, Austria; VASCage Research Centre on Vascular Ageing and Stroke, Austria)*; Hyeon Chang Kim (Yonsei University College of Medicine, Republic of Korea)*; Andrew Kingston (Newcastle University, UK)*; Heidi Klakk (University College South Denmark, Denmark)*; Jana Klanova (Masaryk University, Czechia)*; Michael Knoflach (Medical University of Innsbruck, Austria)*; Patrick Kolsteren (Ghent University, Belgium)*; Jürgen König (University of Vienna, Austria)*; Raija Korpelainen (University of Oulu, Finland)*; Paul Korrovits (Tartu University Clinics, Estonia)*; Jelena Kos (University Hospital Centre Zagreb, Croatia)*; Seppo Koskinen (Finnish Institute for Health and Welfare, Finland)*; Sudhir Kowlessur (Ministry of Health and Wellness, Mauritius)*; Slawomir Koziel (PASs Hirszfeld Institute of Immunology and Experimental Therapy, Poland)*; Wolfgang Kratzer (University Hospital Ulm, Germany)*; Susi Kriemler (University of Zurich, Switzerland)*; Peter Lund Kristensen (University of Southern Denmark, Denmark)*; Steinar Krokstad (Norwegian University of Science and Technology, Norway)*; Daan Kromhout (University of Groningen, The Netherlands)*; Ruzena Kubinova (National Institute of Public Health, Czechia)*; Urho M Kujala (University of Jyväskylä, Finland)*; Mukhtar Kulimbet (Al-Farabi Kazakh National University, Kazakhstan; Asfendiyarov Kazakh National Medical University, Kazakhstan)*; Vaitheeswaran Kulothungan (ICMR - National Centre for Disease Informatics and Research, India)*; Meena Kumari (University of Essex, UK)*; Vladimir Kutsenko (National Medical Research Centre for Therapy and Preventive Medicine, Russia)*; Catherine Kyobutungi (African Population and Health Research Center, Kenya)*; Quang Ngoc La (Hanoi University of Public Health, Vietnam)*; Tiina Laatikainen (University of Eastern Finland, Finland; Finnish Institute for Health and Welfare, Finland)*; Demetre Labadarios (Stellenbosch University, South Africa; University of Limpopo, South

Africa)*; Carl Lachat (Ghent University, Belgium)*; Youcef Laid (Ministry of Health, Algeria)*; Lachmie Lall (Ministry of Health, Guyana)*; Anne Langsted (Copenhagen University Hospital - Herlev and Gentofte, Denmark; University of Copenhagen, Denmark)*; Tiina Lankila (Oulu Deaconess Institute Foundation, Finland)*; Vera Lanska (Institute for Clinical and Experimental Medicine, Czechia)*; Georg Lappas (Sahlgrenska Academy, Sweden)*; Bagher Larijani (Endocrinology and Metabolism Research Center, Iran)*; Tint Swe Latt (University of Public Health, Myanmar)*; Martino Laurenzi (Centro Studi Epidemiologici di Gubbio, Italy)*; Avula Laxmaiah (ICMR - National Institute of Nutrition, India)*; Gwenaëlle Le Coroller (Luxembourg Institute of Health, Luxembourg)*; Jeannette Lee (National University of Singapore, Singapore; National University Health System, Singapore)*; Terho Lehtimäki (Tampere University Hospital, Finland; Tampere University, Finland)*; Daniel Lemogoum (University of Douala, Cameroon)*; Gabriel M Leung (The University of Hong Kong, China)*; Charlie Lim (National University of Singapore, Singapore; National University Health System, Singapore)*; Wei-Yen Lim (Tan Tock Seng Hospital, Singapore)*; M Fernanda Lima-Costa (Fundação Oswaldo Cruz, Brazil)*; Hsien-Ho Lin (National Taiwan University, Taiwan)*; Yi-Jing Lin (Ministry of Health and Welfare, Taiwan)*; Lars Lind (Uppsala University, Sweden)*; Lauren Lissner (University of Gothenburg, Sweden)*; Liping Liu (Beijing Center for Disease Prevention and Control, China)*; Xiaotian Liu (Zhengzhou University, China)*; Wei-Cheng Lo (Taipei Medical University, Taiwan)*; Helle-Mai Loit (National Institute for Health Development, Estonia)*; Esther Lopez-Garcia (CIBERESP, Spain; Universidad Autónoma de Madrid, Spain)*; Tania Lopez (Universidad San Martín de Porres, Peru)*; José Eugenio Lozano (Consejería de Sanidad Junta de Castilla y León, Spain)*; Dalia Luksiene (Lithuanian University of Health Sciences, Lithuania)*; Annamari Lundqvist (Finnish Institute for Health and Welfare, Finland)*; Nuno Lunet (University of Porto, Portugal)*; Thomas Lung (University of Sydney, Australia)*; Michala Lustigová (Charles University, Czechia; National Institute of Public Health, Czechia)*; Guansheng Ma (Peking University, China)*; George LL Machado-Coelho (Universidade Federal de Ouro Preto, Brazil)*; Aristides M Machado-Rodrigues (University of Coimbra, Portugal)*; Enguerran Macia (UMR 7268 ADES, France)*; Luisa M

Macieira (Coimbra University Hospital Center, Portugal)*; Ahmed A Madar (University of Oslo, Norway)*; Gladys E Maestre (University of Texas Rio Grande Valley, USA)*; Stefania Maggi (Institute of Neuroscience of the National Research Council, Italy)*; Dianna J Magliano (Baker Heart and Diabetes Institute, Australia)*; Emmanuella Magriplis (Agricultural University of Athens, Greece)*; Gowri Mahasampath (Christian Medical College Vellore, India)*; Bernard Maire (French National Research Institute for Sustainable Development, France)*; Marcia Makdisse (Academia VBHC, Brazil)*; Konstantinos Makrilakis (National and Kapodistrian University of Athens, Greece)*; Mohammad-Reza Malekpour (Non-Communicable Diseases Research Center, Iran)*; Fatemeh Malekzadeh (Tehran University of Medical Sciences, Iran)*; Reza Malekzadeh (Shiraz University of Medical Sciences, Iran; Tehran University of Medical Sciences, Iran)*; Sofia Malyutina (Institute of Internal and Preventive Medicine, Russia)*; Lynell V Maniego (Food and Nutrition Research Institute, Philippines)*; Yannis Manios (Harokopio University, Greece)*; Fariborz Mansour-Ghanaei (Guilan University of Medical Sciences, Iran)*; Enzo Manzato (University of Padua, Italy)*; Mala Ali Mapatano (University of Kinshasa, DR Congo)*; Anie Marcil (Statistics Canada, Canada)*; Francisco Mardones (Pontificia Universidad Católica de Chile, Chile)*; Paula Margozzini (Pontificia Universidad Católica de Chile, Chile)*; Pedro Marques-Vidal (Lausanne University Hospital, Switzerland; University of Lausanne, Switzerland)*; Larissa Pruner Marques (Fundação Oswaldo Cruz, Brazil)*; Reynaldo Martorell (Emory University, USA)*; Luis P Mascarenhas (Universidade Estadual do Centro-Oeste, Brazil)*; Mannix Masimango Imani (Université Catholique de Bukavu, DR Congo)*; Masoud Masinaei (Non-Communicable Diseases Research Center, Iran)*; Shariq R Masoodi (Sher-i-Kashmir Institute of Medical Sciences, India)*; Ellisiv B Mathiesen (UiT The Arctic University of Norway, Norway)*; Prashant Mathur (ICMR - National Centre for Disease Informatics and Research, India)*; Tandi E Matsha (Sefako Makgatho Health Sciences University, South Africa)*; Jean Claude N Mbanya (University of Yaoundé 1, Cameroon)*; Anselmo J Mc Donald Posso (Instituto Conmemorativo Gorgas de Estudios de la Salud, Panama)*; Shelly R McFarlane (The University of the West Indies, Jamaica)*; Stephen T McGarvey (Brown University, USA)*; Scott B McLean (Statistics Canada, Canada)*;

Breige A McNulty (University College Dublin, Ireland)*; Sounnia Mediene Benchekor (University Oran 1, Algeria)*; Kirsten Mehlig (University of Gothenburg, Sweden)*; Amir Houshang Mehrparvar (Shahid Sadoughi University of Medical Sciences, Iran)*; Jesus D Melgarejo (University of Texas Rio Grande Valley, USA)*; Fabián Méndez (Universidad del Valle, Colombia)*; Ana Maria B Menezes (Universidade Federal de Pelotas, Brazil)*; Alibek Mereke (Al-Farabi Kazakh National University, Kazakhstan)*; Indrapal I Meshram (ICMR - National Institute of Nutrition, India)*; Diane T Meto (University of Abidjan, Côte d'Ivoire)*; Nathalie Michels (Ghent University, Belgium)*; Cláudia S Minderico (Universidade de Lisboa, Portugal)*; GK Mini (Saveetha Dental College and Hospitals, India)*; Juan Francisco Miquel (Pontificia Universidad Católica de Chile, Chile)*; J Jaime Miranda (University of Sydney, Australia)*; Mohammad Reza Mirjalili (Shahid Sadoughi University of Medical Sciences, Iran)*; Daphne Mirkopoulou (Democritus University, Greece)*; Pietro A Modesti (Università degli Studi di Firenze, Italy)*; Sahar Saeedi Moghaddam (Non-Communicable Diseases Research Center, Iran)*; Kazem Mohammad (Tehran University of Medical Sciences, Iran)*; Mohammad Reza Mohammadi (Psychiatry and Psychology Research Center, Iran)*; Zahra Mohammadi (Tehran University of Medical Sciences, Iran)*; Noushin Mohammadifard (Isfahan Cardiovascular Research Center, Iran)*; Reza Mohammadpourhodki (Mashhad University of Medical Sciences, Iran)*; Viswanathan Mohan (Madras Diabetes Research Foundation, India)*; Muhammad Fadhli Mohd Yusoff (Ministry of Health, Malaysia)*; Iraj Mohebbi (Urmia University of Medical Sciences, Iran)*; Niels C Møller (University of Southern Denmark, Denmark)*; Dénes Molnár (University Medical School of Pécs, Hungary)*; Amirabbas Momenan (Shahid Beheshti University of Medical Sciences, Iran)*; Roger A Montenegro Mendoza (Gorgas Memorial Institute for Studies of Health, Panama)*; Mahmood Moosazadeh (Mazandaran University of Medical Sciences, Iran)*; Farhad Moradpour (Kurdistan University of Medical Sciences, Iran)*; Alain Morejon (University of Medical Sciences of Cienfuegos, Cuba)*; Luis A Moreno (CIBEROBN, Spain; Fundación Instituto de Investigación Sanitaria Aragón, Spain)*; Karen Morgan (Royal College of Surgeons in Ireland, Ireland)*; Suzanne N Morin (McGill University, Canada)*; George Moschonis (La Trobe University, Australia)*;

Alireza Moslem (Sabzevar University of Medical Sciences, Iran)*; Mildrey Mosquera (Universidad del Valle, Colombia)*; Malgorzata Mossakowska (International Institute of Molecular and Cell Biology, Poland)*; Aya Mostafa (Ain Shams University, Egypt)*; Seyed-Ali Mostafavi (Tehran University of Medical Sciences, Iran)*; Eugen Mota (University of Medicine and Pharmacy Craiova, Romania)*; Jorge Mota (University of Porto, Portugal)*; Maria Mota (University of Medicine and Pharmacy Craiova, Romania)*; Mohammad Esmaeel Motlagh (Ahvaz Jundishapur University of Medical Sciences, Iran)*; Jorge Motta (Instituto Conmemorativo Gorgas de Estudios de la Salud, Panama)*; Kelias P Msyamboza (World Health Organization Country Office, Malawi)*; Thet Thet Mu (Department of Public Health, Myanmar)*; Maria L Muiesan (University of Brescia, Italy)*; Patricia B Munroe (Queen Mary University, UK)*; Jaakko Mursu (University of Eastern Finland, Finland)*; Kamarul Imran Musa (Universiti Sains Malaysia, Malaysia)*; Norlaila Mustafa (Universiti Kebangsaan Malaysia, Malaysia)*; Muel Telo MC Muyer (University of Kinshasa, DR Congo)*; Iraj Nabipour (Bushehr University of Medical Sciences, Iran)*; Gabriele Nagel (Ulm University, Germany)*; Balkish M Naidu (Department of Statistics, Malaysia)*; Farid Najafi (Kermanshah University of Medical Sciences, Iran)*; Jana Námešná (Regional Authority of Public Health, Slovakia)*; Ei Ei K Nang (National University of Singapore, Singapore; National University Health System, Singapore)*; Vinay B Nangia (Suraj Eye Institute, India)*; KM Venkat Narayan (Emory University, USA)*; Take Naseri (Ministry of Health, Samoa)*; Ana J Navarro-Ramírez (Caja Costarricense de Seguro Social, Costa Rica)*; Nareemarn Neelapaichit (Mahidol University, Thailand)*; Azim Nejatizadeh (Hormozgan University of Medical Sciences, Iran)*; Ilona Nenko (Jagiellonian University Medical College, Poland)*; Flavio Nervi (Pontificia Universidad Católica de Chile, Chile)*; Tze Pin Ng (National University of Singapore, Singapore)*; Chung T Nguyen (National Institute of Hygiene and Epidemiology, Vietnam)*; Nguyen D Nguyen (University of Medicine and Pharmacy at Ho Chi Minh City, Vietnam)*; Quang Ngoc Nguyen (Hanoi Medical University, Vietnam)*; Michael Y Ni (The University of Hong Kong, China)*; Peng Nie (Xi'an Jiaotong University, China)*; Ramfis E Nieto-Martínez (Precision Care Clinic Corp, USA)*; Guang Ning (Shanghai Jiao-Tong University School of Medicine, China)*; Toshiharu Ninomiya (Kyushu

University, Japan)*; Nobuo Nishi (National Institutes of Biomedical Innovation, Health and Nutrition, Japan)*; Marianna Noale (Institute of Neuroscience of the National Research Council, Italy)*; Oscar A Noboa (Universidad de la República, Uruguay)*; Mitsuhiko Noda (International University of Health and Welfare, Japan)*; Børge G Nordestgaard (Copenhagen University Hospital - Herlev and Gentofte, Denmark; University of Copenhagen, Denmark)*; Davide Noto (University of Palermo, Italy)*; Mohannad Al Nsour (Eastern Mediterranean Public Health Network, Jordan)*; Irfan Nuhoğlu (Karadeniz Technical University, Türkiye)*; Moffat Nyirenda (London School of Hygiene & Tropical Medicine, UK)*; Terence W O'Neill (University of Manchester, UK)*; Kyungwon Oh (Korea Disease Control and Prevention Agency, Republic of Korea)*; Ryutaro Ohtsuka (Japan Wildlife Research Center, Japan; deceased)*; Mohd Azahadi Omar (Ministry of Health, Malaysia)*; Altan Onat (Istanbul University, Türkiye; deceased)*; Sok King Ong (Ministry of Health, Brunei)*; Obinna Onodugo (University of Nigeria, Nigeria)*; Pedro Ordunez (Pan American Health Organization, USA)*; Rui Ornelas (University of Madeira, Portugal)*; Pedro J Ortiz (Universidad Peruana Cayetano Heredia, Peru)*; Clive Osmond (University of Southampton, UK)*; Afshin Ostovar (Tehran University of Medical Sciences, Iran)*; Johanna A Otero (Universidad de Santander, Colombia)*; Charlotte B Ottendahl (University of Southern Denmark, Denmark)*; Akaninyene Otu (University of Calabar, Nigeria)*; Ellis Owusu-Dabo (Kwame Nkrumah University of Science and Technology, Ghana)*; Elena Pahomova (University of Latvia, Latvia)*; Luigi Palmieri (Istituto Superiore di Sanità, Italy)*; Wen-Harn Pan (Academia Sinica, Taiwan)*; Demosthenes Panagiotakos (Harokopio University in Athens, Greece)*; Songhomitra Panda-Jonas (University Hospital Heidelberg, Germany)*; Zengchang Pang (Qingdao Centers for Disease Control and Prevention, China)*; Francesco Panza (University of Bari Aldo Moro, Italy)*; Mariela Paoli (University of the Andes, Venezuela)*; Suyeon Park (Korea Disease Control and Prevention Agency, Republic of Korea)*; Mahboubeh Parsaeian (Tehran University of Medical Sciences, Iran)*; Chona F Patalen (Food and Nutrition Research Institute, Philippines)*; Nikhil D Patel (Jivandeep Hospital, India)*; Raimund Pechlaner (Medical University of Innsbruck, Austria)*; Ivan Pećin (University Hospital Centre Zagreb, Croatia)*;

João M Pedro (Centro de Investigação em Saúde de Angola, Angola)*; Sergio Viana Peixoto (Fundação Oswaldo Cruz, Brazil)*; Markku Peltonen (Finnish Institute for Health and Welfare, Finland)*; Alexandre C Pereira (University of São Paulo, Brazil)*; Thaliane Mayara Pessôa dos Prazeres (Universidade Federal de Pernambuco, Brazil)*; Niloofar Peykari (Ministry of Health and Medical Education, Iran)*; Modou Cheyassin Phall (Gambia National Nutrition Agency, The Gambia)*; Son Thai Pham (Vietnam National Heart Institute, Vietnam)*; Rafael N Pichardo (Clínica de Medicina Avanzada Dr. Abel González, Dominican Republic)*; Iris Pigeot (Leibniz Institute for Prevention Research and Epidemiology - BIPS, Germany)*; Hynek Pikhart (University College London, UK)*; Aida Pilav (University of Sarajevo, Bosnia and Herzegovina)*; Pavel Piler (Masaryk University, Czechia)*; Freda Pitakaka (Ministry of Health and Medical Services, Solomon Islands)*; Aleksandra Piwonska (National Institute of Cardiology, Poland)*; Andreia N Pizarro (University of Porto, Portugal)*; Pedro Plans-Rubió (College of Physicians of Barcelona, Spain)*; Silvia Plata (Observatorio de Salud Pública de Santander, Colombia)*; Barry M Popkin (University of North Carolina at Chapel Hill, USA)*; Miquel Porta (CIBERESP, Spain; Hospital del Mar Research Institute, Spain)*; Anil Poudyal (Nepal Health Research Council, Nepal)*; Farhad Pourfarzi (Ardabil University of Medical Sciences, Iran)*; Akram Pourshams (Tehran University of Medical Sciences, Iran)*; Hossein Poustchi (Tehran University of Medical Sciences, Iran)*; Dorairaj Prabhakaran (Public Health Foundation of India, India)*; Rajendra Pradeepa (Madras Diabetes Research Foundation, India)*; Alison J Price (London School of Hygiene & Tropical Medicine, UK)*; Jacqueline F Price (University of Edinburgh, UK)*; Rui Providencia (University College London, UK)*; Jardena J Puder (Lausanne University Hospital, Switzerland)*; Soile Puhakka (Oulu Deaconess Institute Foundation, Finland)*; Margus Punab (Tartu University Clinics, Estonia)*; Qing Qiao (Boehringer Ingelheim, Germany)*; Mostafa Qorbani (Alborz University of Medical Sciences, Iran)*; Hedley K Quintana (Gorgas Memorial Institute for Studies of Health, Panama)*; Tran Quoc Bao (Ministry of Health, Vietnam)*; Ricardas Radisauskas (Lithuanian University of Health Sciences, Lithuania)*; Salar Rahimikazerooni (Shiraz University of Medical Sciences, Iran)*; Olli Raitakari (University of Turku, Finland)*; Ambady

Ramachandran (India Diabetes Research Foundation, India)*; Manuel Ramirez-Zea (Institute of Nutrition of Central America and Panama, Guatemala)*; Jacqueline Ramke (University of Auckland, New Zealand)*; Elisabete Ramos (University of Porto, Portugal)*; Rafel Ramos (Institut Universitari d'Investigació en Atenció Primària Jordi Gol, Spain)*; Lekhraj Rampal (Universiti Putra Malaysia, Malaysia)*; Sanjay Rampal (University of Malaya, Malaysia)*; Sheena E Ramsay (Newcastle University, UK)*; Daniel A Rangel Reina (Instituto Conmemorativo Gorgas de Estudios de la Salud, Panama)*; Ravindra P Rannan-Eliya (Institute for Health Policy, Sri Lanka)*; Mohammad-Mahdi Rashidi (Non-Communicable Diseases Research Center, Iran)*; Josep Redon (University of Valencia, Spain)*; Jane DP Renner (University of Santa Cruz do Sul, Brazil)*; Cézane P Reuter (University of Santa Cruz do Sul, Brazil)*; Luis Revilla (Universidad San Martín de Porres, Peru)*; Negar Rezaei (Tehran University of Medical Sciences, Iran)*; Abbas Rezaianzadeh (Shiraz University of Medical Sciences, Iran)*; Yeunsook Rho (National Health Insurance Service, Republic of Korea)*; Fernando Rigo (CS S. Agustín Ibsalut, Spain)*; Leanne M Riley (World Health Organization, Switzerland)*; Ulf Risérus (Uppsala University, Sweden)*; Reina G Roa (Ministerio de Salud, Panama)*; Louise Robinson (Newcastle University, UK)*; Wendy E Rodríguez-Anderson (Caja Costarricense de Seguro Social, Costa Rica)*; Fernando Rodríguez-Artalejo (CIBERESP, Spain; Universidad Autónoma de Madrid, Spain)*; María del Cristo Rodriguez-Perez (Canarian Health Service, Spain)*; Laura A Rodríguez-Villamizar (Universidad Industrial de Santander, Colombia)*; Andrea Y Rodríguez (Ministry of Health and Social Protection, Colombia)*; Ulla Roggenbuck (University of Duisburg-Essen, Germany)*; Peter Rohloff (Wuqu' Kawoq, Guatemala)*; Rosalba Rojas-Martinez (National Institute of Public Health, Mexico)*; Elisabetta L Romeo (Associazione Calabrese di Epatologia, Italy)*; Annika Rosengren (University of Gothenburg, Sweden; Sahlgrenska University Hospital, Sweden)*; Joel GR Roy (Statistics Canada, Canada)*; Adolfo Rubinstein (Institute for Clinical Effectiveness and Health Policy, Argentina)*; Maria Ruiz-Castell (Luxembourg Institute of Health, Luxembourg)*; Paola Russo (National Research Council, Italy)*; Petra Rust (University of Vienna, Austria)*; Marcin Rutkowski (Medical University of Gdańsk, Poland)*;

Charumathi Sabanayagam (Singapore Eye Research Institute, Singapore)*; Hamideh Sabbaghi (Shahid Beheshti University of Medical Sciences, Iran)*; Harshpal S Sachdev (Sitaram Bhartia Institute of Science and Research, India)*; Alireza Sadjadi (Tehran University of Medical Sciences, Iran)*; Ali Reza Safarpour (Shiraz University of Medical Sciences, Iran)*; Sare Safi (Shahid Beheshti University of Medical Sciences, Iran)*; Saeid Safiri (Tabriz University of Medical Sciences, Iran)*; Mohammad Hossien Saghi (Sabzevar University of Medical Sciences, Iran)*; Olfa Saidi (University Tunis El Manar, Tunisia)*; Satoko Sakata (Kyushu University, Japan)*; Nader Saki (Ahvaz Jundishapur University of Medical Sciences, Iran)*; Sanja Šalaj (University of Zagreb, Croatia)*; Benoit Salanave (French Public Health Agency, France)*; Jukka T Salonen (University of Helsinki, Finland)*; Massimo Salvetti (University of Brescia, Italy)*; Jose Sánchez-Abanto (National Institute of Health, Peru)*; Diana A Santos (Universidade de Lisboa, Portugal)*; Lèlita C Santos (Coimbra University Hospital Center, Portugal)*; Maria Paula Santos (University of Porto, Portugal)*; Rute Santos (University of Porto, Portugal)*; Tamara R Santos (Universidade Federal de Alagoas, Brazil)*; Jouko L Saramies (Wellbeing Services County of South Karelia, Finland)*; Luis B Sardinha (Universidade de Lisboa, Portugal)*; Nizal Sarrafzadegan (Isfahan Cardiovascular Research Center, Iran)*; Yoko Sato (Shizuoka Graduate University of Public Health, Japan)*; Kai-Uwe Saum (German Cancer Research Center, Germany)*; Stefan Savin (World Health Organization, Switzerland)*; Norie Sawada (National Cancer Center, Japan)*; Mariana Sbaraini (Universidade Federal do Rio Grande do Sul, Brazil)*; Marcia Scazufca (University of São Paulo Clinics Hospital, Brazil)*; Beatriz D Schaan (Universidade Federal do Rio Grande do Sul, Brazil)*; Herman Schargrodsky (Hospital Italiano de Buenos Aires, Argentina; deceased)*; Christa Scheidt-Nave (Robert Koch Institute, Germany)*; Sabine Schipf (University Medicine Greifswald, Germany)*; Amand Floriaan Schmidt (University College London, UK; University of Amsterdam, The Netherlands)*; Börge Schmidt (University of Duisburg-Essen, Germany)*; Carsten O Schmidt (University Medicine Greifswald, Germany)*; Peter Schnohr (Copenhagen University Hospital - Bispebjerg and Frederiksberg, Denmark)*; Catherine Mary Schooling (The University of Hong Kong, China)*; Ben Schöttker (German

Cancer Research Center, Germany)*; Sara Schramm (University of Duisburg-Essen, Germany)*; Sylvain Sebert (University of Oulu, Finland)*; Moslem Sedaghattalab (Yasuj University of Medical Sciences, Iran)*; Aye Aye Sein (Ministry of Health and Sports, Myanmar)*; Abhijit Sen (Center for Oral Health Services and Research Mid-Norway, Norway)*; Sadaf G Sepanlou (Tehran University of Medical Sciences, Iran)*; Jennifer Servais (Statistics Canada, Canada)*; Ronel Sewpaul (Human Sciences Research Council, South Africa)*; Svetlana Shalnova (National Medical Research Centre for Therapy and Preventive Medicine, Russia)*; Seyed Morteza Shamshirgaran (Neyshabur University of Medical Sciences, Iran)*; Coimbatore Subramaniam Shanthirani (Madras Diabetes Research Foundation, India)*; Maryam Sharafkhah (Tehran University of Medical Sciences, Iran)*; Sanjib K Sharma (B P Koirala Institute of Health Sciences, Nepal)*; Almaz Sharman (Academy of Preventive Medicine, Kazakhstan)*; Jonathan E Shaw (Baker Heart and Diabetes Institute, Australia)*; Amaneh Shayanrad (Tehran University of Medical Sciences, Iran)*; Ali Akbar Shayesteh (Ahvaz Jundishapur University of Medical Sciences, Iran)*; Kenji Shibuya (King's College London, UK)*; Hana Shimizu-Furusawa (Teikyo University, Japan)*; Rahman Shiri (Finnish Institute of Occupational Health, Finland)*; Marat Shoranov (Kazakh National Medical University, Kazakhstan)*; Namuna Shrestha (Public Health Promotion and Development Organization, Nepal)*; Khairil Si-Ramlee (Ministry of Health, Brunei)*; Alfonso Siani (National Research Council, Italy)*; Mark J Siedner (Africa Health Research Institute, South Africa; University of KwaZulu-Natal, South Africa)*; Diego Augusto Santos Silva (Universidade Federal de Santa Catarina, Brazil)*; Xueling Sim (National University of Singapore, Singapore; National University Health System, Singapore)*; Mary Simon (India Diabetes Research Foundation, India)*; Judith Simons (St Vincent's Hospital, Australia)*; Leon A Simons (University of New South Wales, Australia)*; Michael Sjöström (Karolinska Institutet, Sweden; deceased)*; Jolanta Slowikowska-Hilczer (Medical University of Lodz, Poland)*; Przemysław Slusarczyk (International Institute of Molecular and Cell Biology, Poland)*; Liam Smeeth (London School of Hygiene & Tropical Medicine, UK)*; Eugène Sobngwi (University of Yaoundé 1, Cameroon)*; Stefan Söderberg (Umeå University, Sweden)*; Agustinus

Soemantri (Diponegoro University, Indonesia; deceased)*; Vincenzo Solfrizzi (University of Bari Aldo Moro, Italy)*; Mohammad Hossein Somi (Tabriz University of Medical Sciences, Iran)*; Aïcha Soumaré (University of Bordeaux, France)*; Alfonso Sousa-Poza (University of Hohenheim, Germany)*; Mafalda Sousa-Uva (National Institute of Health Doutor Ricardo Jorge, Portugal)*; Karen Sparrenberger (Universidade Federal do Rio Grande do Sul, Brazil)*; Jan A Staessen (KU Leuven, Belgium)*; Andreas Stang (University of Duisburg-Essen, Germany)*; Bill Stavreski (Heart Foundation, Australia)*; Jostein Steene-Johannessen (Norwegian School of Sport Sciences, Norway)*; Peter Stehle (Bonn University, Germany)*; Aryeh D Stein (Emory University, USA)*; Jochanan Stessman (Hadassah University Medical Center, Israel)*; Jakub Stokwiszewski (National Institute of Public Health NIH – National Research Institute, Poland)*; Karien Stronks (University of Amsterdam, The Netherlands)*; Milton F Suarez-Ortegón (Pontificia Universidad Javeriana Seccional Cali, Colombia)*; Phalakorn Suebsamran (Ubon Ratchathani University, Thailand)*; Machi Suka (The Jikei University School of Medicine, Japan)*; Chien-An Sun (Fu Jen Catholic University, Taiwan)*; Jianping Sun (Qingdao Municipal Health Admission, China)*; Johan Sundström (Uppsala University, Sweden)*; Paibul Suriyawongpaisal (Mahidol University, Thailand)*; René Charles Sylva (National Statistical Office, Cabo Verde)*; E Shyong Tai (National University of Singapore, Singapore; National University Health System, Singapore)*; Furusawa Takuro (Kyoto University, Japan)*; Abdonas Tamosiunas (Lithuanian University of Health Sciences, Lithuania)*; Eng Joo Tan (Monash University, Australia)*; Baimakhan Tanabayev (South Kazakhstan Medical Academy, Kazakhstan)*; Nikhil Tandon (All India Institute of Medical Sciences, India)*; Mohammed Rasoul Tarawneh (Ministry of Health, Jordan)*; Carolina B Tarqui-Mamani (National Institute of Health, Peru)*; Anne Taylor (University of Adelaide, Australia)*; Tania Tello (Universidad Peruana Cayetano Heredia, Peru)*; Yih Chung Tham (National University of Singapore, Singapore)*; KR Thankappan (Amrita Institute of Medical Sciences, India)*; Holger Theobald (Karolinska Institutet, Sweden)*; Xenophon Theodoridis (Aristotle University of Thessaloniki, Greece)*; Nihal Thomas (Christian Medical College Vellore, India)*; Amanda G Thrift (Monash University, Australia)*; Erik J Timmermans

(University Medical Center Utrecht, The Netherlands)*; Hanna K Tolonen (Finnish Institute for Health and Welfare, Finland)*; Janne S Tolstrup (University of Southern Denmark, Denmark)*; Maciej Tomaszewski (University of Manchester, UK)*; Murat Topbas (Karadeniz Technical University, Türkiye)*; Michael J Tornaritis (Research and Education Institute of Child Health, Cyprus)*; Maties Torrent (IB-SALUT Area de Salut de Menorca, Spain)*; Laura Torres-Collado (CIBERESP, Spain; Universidad Miguel Hernández de Elche, Spain)*; Giota Touloumi (National and Kapodistrian University of Athens, Greece)*; Pierre Traissac (French National Research Institute for Sustainable Development, France)*; Areti Triantafyllou (Aristotle University of Thessaloniki, Greece)*; Oanh TH Trinh (University of Medicine and Pharmacy at Ho Chi Minh City, Vietnam)*; Yu-Hsiang Tsao (Institute of Epidemiology and Preventive Medicine, Taiwan)*; Thomas Tsiampalis (Harokopio University in Athens, Greece)*; Shoichiro Tsugane (National Institutes of Biomedical Innovation, Health and Nutrition, Japan)*; John Tuitele (Tafuna Family Health Center, American Samoa; LBJ Hospital, American Samoa)*; Azaliia M Tuliakova (Ufa Eye Research Institute, Russia)*; Marshall K Tulloch-Reid (The University of the West Indies, Jamaica)*; Tomi-Pekka Tuomainen (University of Eastern Finland, Finland)*; Maria L Turley (Ministry of Health, New Zealand)*; Evangelia Tzala (Imperial College London, UK)*; Christophe Tzourio (University of Bordeaux, France)*; Peter Ueda (Karolinska Institutet, Sweden)*; Eunice Ugel (Universidad Centro-Occidental Lisandro Alvarado, Venezuela)*; Flora AM Ukoli (Meharry Medical College, USA)*; Hanno Ulmer (Medical University of Innsbruck, Austria)*; Hannu MT Uusitalo (University of Tampere Tays Eye Center, Finland)*; Gonzalo Valdivia (Pontificia Universidad Católica de Chile, Chile)*; Damaskini Valvi (Icahn School of Medicine at Mount Sinai, USA)*; Rob M van Dam (George Washington University, USA)*; Bert-Jan van den Born (University of Amsterdam, The Netherlands)*; Johan Van der Heyden (Sciensano, Belgium)*; Hoang Van Minh (Hanoi University of Public Health, Vietnam)*; Lenie van Rossem (University Medical Center Utrecht, The Netherlands)*; Natasja M Van Schoor (Vrije Universiteit Amsterdam, The Netherlands)*; Irene GM van Valkengoed (University of Amsterdam, The Netherlands)*; Dirk Vanderschueren (KU Leuven, Belgium)*; Diego Vanuzzo (MONICA FRIULI Study Group,

Italy)*; Anette Varbo (Copenhagen University Hospital - Herlev and Gentofte, Denmark; University of Copenhagen, Denmark)*; Senthil K Vasan (University of Southampton, UK)*; Tomas Vega (Consejería de Sanidad Junta de Castilla y León, Spain)*; Toomas Veidebaum (National Institute for Health Development, Estonia)*; Gustavo Velasquez-Melendez (Universidade Federal de Minas Gerais, Brazil)*; Charlotte Verdot (The National Public Health Agency, France)*; Giovanni Veronesi (University of Insubria, Italy)*; Roosmarijn Verstraeten (Institute of Tropical Medicine, Belgium)*; Cesar G Victora (Universidade Federal de Pelotas, Brazil)*; Lucie Viet (National Institute for Public Health and the Environment, The Netherlands)*; Luis Villarroel (Pontificia Universidad Católica de Chile, Chile)*; Jesus Vioque (CIBERESP, Spain; Universidad Miguel Hernández de Elche, Spain)*; Jyrki K Virtanen (University of Eastern Finland, Finland)*; Bharathi Viswanathan (Ministry of Health, Seychelles)*; Peter Vollenweider (Lausanne University Hospital, Switzerland; University of Lausanne, Switzerland)*; Ari Voutilainen (University of Eastern Finland, Finland)*; Martine Vrijheid (CIBERESP, Spain; Barcelona Institute for Global Health, Spain)*; Alisha N Wade (University of the Witwatersrand, South Africa)*; Janette Walton (Munster Technological University, Ireland)*; Wan Mohamad Wan Bebakar (Universiti Sains Malaysia, Malaysia)*; Wan Nazaimoon Wan Mohamud (Institute for Medical Research, Malaysia)*; Chongjian Wang (Zhengzhou University, China)*; Huijun Wang (Chinese Center for Disease Control and Prevention, China)*; Ningli Wang (Capital Medical University Beijing Tongren Hospital, China)*; Qian Wang (Xinjiang Medical University, China)*; Weiqing Wang (Shanghai Jiao-Tong University School of Medicine, China)*; Ya Xing Wang (Capital Medical University, China)*; Yi-Ren Wang (Ministry of Health and Welfare, Taiwan)*; Ying-Wei Wang (Ministry of Health and Welfare, Taiwan)*; S Goya Wannamethee (University College London, UK)*; Karen Webster-Kerr (The Ministry of Health and Wellness, Jamaica)*; Niels Wedderkopp (University of Southern Denmark, Denmark)*; Wenbin Wei (Capital Medical University, China)*; Leo D Westbury (University of Southampton, UK)*; Peter H Whincup (St George's, University of London, UK)*; Kurt Widhalm (Medical University of Vienna, Austria)*; Indah S Widyahening (Universitas Indonesia, Indonesia)*; Andrzej Więcek (Medical University of

Silesia, Poland)*; Nilmini Wijemunige (Institute for Health Policy, Sri Lanka)*; Rainford J Wilks (The University of the West Indies, Jamaica)*; Karin Willeit (Medical University of Innsbruck, Austria)*; Peter Willeit (Medical University of Innsbruck, Austria)*; Tom Wilsgaard (UiT The Arctic University of Norway, Norway)*; Bogdan Wojtyniak (National Institute of Public Health NIH – National Research Institute, Poland)*; Roy A Wong-McClure (Caja Costarricense de Seguro Social, Costa Rica)*; Andrew Wong (University College London, UK)*; Emily B Wong (Africa Health Research Institute, South Africa)*; Mark Woodward (University of New South Wales, Australia; Imperial College London, UK)*; Chao-Chun Wu (Ministry of Health and Welfare, Taiwan)*; Frederick C Wu (University of Manchester, UK)*; Haiquan Xu (Institute of Food and Nutrition Development of Ministry of Agriculture and Rural Affairs, China)*; Liang Xu (Beijing Institute of Ophthalmology, China)*; Yu Xu (Shanghai Jiao-Tong University School of Medicine, China)*; Nor Azwany Yaacob (Universiti Sains Malaysia, Malaysia)*; Li Yan (Imperial College London, UK)*; Weili Yan (Children's Hospital of Fudan University, China)*; Tabara Yasuharu (Shizuoka Graduate University of Public Health, Japan)*; Chao-Yu Yeh (Ministry of Health and Welfare, Taiwan)*; Moein Yoosefi (Non-Communicable Diseases Research Center, Iran)*; Akihiro Yoshihara (Niigata University, Japan)*; San-Lin You (Fu Jen Catholic University, Taiwan)*; Novie O Younger-Coleman (The University of the West Indies, Jamaica)*; Yu-Ling Yu (KU Leuven, Belgium)*; Ahmad Faudzi Yusoff (Ministry of Health, Malaysia)*; Ahmad A Zainuddin (Ministry of Health, Malaysia)*; Farhad Zamani (Iran University of Medical Sciences, Iran)*; Sabina Zambon (University of Padua, Italy)*; Antonis Zampelas (Agricultural University of Athens, Greece)*; Abdul Hamid Zargar (Center for Diabetes and Endocrine Care, India)*; Ko Ko Zaw (University of Public Health, Myanmar)*; Tomasz Zdrojewski (Medical University of Gdańsk, Poland)*; Tajana Zeljkovic Vrkic (University Hospital Centre Zagreb, Croatia)*; Yi Zeng (Peking University, China; Duke University, USA)*; Bing Zhang (Chinese Center for Disease Control and Prevention, China)*; Lei Zhang (Qingdao Endocrine and Diabetes Hospital, China)*; Luxia Zhang (Peking University First Hospital, China)*; Zhen-Yu Zhang (KU Leuven, Belgium)*; Ming-Hui Zhao (Peking University First Hospital, China)*; Wenhua Zhao (Chinese Center for Disease Control

and Prevention, China)*; Bekbolat Zholdin (West Kazakhstan Medical University, Kazakhstan)*; Paul Zimmet (Monash University, Australia)*; Marie Zins (Institut National de la Santé et de la Recherche Médicale, France; Paris Cité University, France)*; Emanuel Zitt (Agency for Preventive and Social Medicine, Austria)*; Nada Zoghlami (National Institute of Public Health, Tunisia)*; Julio Zuñiga Cisneros (Instituto Conmemorativo Gorgas de Estudios de la Salud, Panama)*

Appendix Text 2. Data sources

Data access

We used data from a population-based database on cardiometabolic risk factors collated by the Non-Communicable Disease Risk Factor Collaboration (NCD-RisC), as detailed previously.[1,2] Data were obtained from publicly available multi-country and national measurement surveys (e.g., WHO STEPwise approach to Surveillance (STEPS) surveys, and those identified via the Inter-University Consortium for Political and Social Research, UK Data Service, and European Health Interview & Health Examination Surveys Database). With the collaboration of the WHO and its regional and country offices, we identified and accessed population-based survey data from national health and statistical agencies. We searched and reviewed published studies as detailed previously,[1] and invited eligible studies to join NCD-RisC, as we did with data holders from earlier pooled analyses of cardiometabolic risk factors.[3-6] The NCD-RisC database is continuously updated through all the above routes as well as through periodic requests to NCD-RisC members to suggest additional sources in their countries.

Data inclusion criteria and characteristics of included studies

We carefully checked that each data source met our inclusion criteria, described below. All NCD-RisC members were also periodically asked to review the list of sources from their country, to verify that they met the inclusion criteria and were not duplicates. Potential duplicate data sources were first identified by comparing studies from the same country and year, followed by checking with NCD-RisC members who had provided data whether sources from the same country and year, and with similar sample sizes and age ranges, were the same or distinct. If two sources were confirmed as duplicates, one was discarded.

For each data source, we recorded the study population, the sampling approach, the years of measurement and the measurement methods. Only population-based data were included. All data were assessed and classified by whether they covered the whole country, one or more

subnational regions (i.e., one or more provinces or states, more than three cities, or more than five rural communities), or one or a small number of communities (limited geographical scope not meeting above national or subnational criteria). As stated in statistical methods, these study-level attributes were used in the Bayesian hierarchical meta-regression model so that all available data were used, while taking into account the aforementioned differences in the populations from which different studies had sampled. We recorded whether FPG and HbA1c were measured in a laboratory or using a point-of-care portable device. For studies with laboratory measurement, we recorded whether plasma or whole blood was used. For studies that used portable devices, we verified using the device manual, discussion the study investigators, and if relevant the manufacturers, whether the device reported glucose values in whole blood or whether it converted to plasma-equivalent internally, with those reporting glucose in whole blood converted to plasma equivalent as described below. This information was used so that the calculated prevalence for each survey was correctly and consistently calculated using plasma-equivalent glucose. All submitted data were checked by at least two persons independently. Questions and clarifications were discussed with NCD-RisC members and resolved before data were incorporated into the database.

Data were included if the following criteria were met: measured data on FPG and/or HbA1c were available; study participants were 18 years of age and older; data were from population samples at the national, subnational, or community level; and data were from the countries listed in Appendix Table 2.

We excluded all data sources that were solely based on self-reported diagnosis of diabetes or a registry of people with diagnosed diabetes because a substantial proportion of people with diabetes remain undiagnosed, especially in low- and middle-income countries.[7,8] We excluded data sources on population subgroups whose glycaemic levels may be systematically high or low, including studies that had included or excluded participants based on health status; studies whose participants were only from specific educational, occupational,

socioeconomic or ethnic subgroups. We also excluded studies that recruited through health facilities; the exceptions to this exclusion were studies whose sampling frame was health insurance schemes that are not segregated by occupation or socioeconomic status in countries where at least 80% of the population were insured, and studies based on primary care system in high-income and central European countries with universal insurance, as contact with the primary care systems in these countries tends to be as good as or better than response rates for population-based surveys.

We excluded FPG data from studies that had not instructed participants to fast for at least 6 hours before FPG measurement. We excluded FPG or HbA1c data in studies that measured these biomarkers only among participants with previously-diagnosed diabetes or those with high casual glucose levels. We excluded HbA1c data from studies whose decision to measure HbA1c depended on participants' FPG or casual glucose levels, and vice versa. We also excluded HbA1c data from studies whose mid-year was before 2000, before HbA1c assays were standardised.[9] When FPG and/or HbA1c data were missing for more than 10% of participants in a survey, we contacted the data providers and checked the study design documentation to verify that missingness was not based on pre-selected criteria and hence likely at random.

In addition to the studies in the NCD-RisC database, summary statistics such as diabetes prevalence, mean FPG and mean HbA1c for nationally representative studies that could not be accessed via the above routes were extracted from published reports. We also included such summaries from a previous global data pooling study,[4] when individual record data could not be accessed through the above routes, for example because the authors had retired or moved, data had been permanently archived, or data were stored using older storage technologies that could not be easily retrieved.

Data cleaning and management

In surveys with data on treatment, we determined whether a person was taking medication for diabetes using survey-specific questions worded as variations of "are you currently taking medication for diabetes or high blood sugar?"; or the combination of "do you currently inject/use insulin for diabetes?" and "are you currently taking any medicines, tablets, or pills for diabetes?"; or using information gathered on types of medicines used by the participant. Fifteen studies (1%) had only collected data on the use of oral hypoglycaemic drugs, but not insulin. For these studies, we used medication data for participants aged 40 years and older, but not for younger participants. We did this because data from studies that had information on both types of treatment showed that using insulin alone was more common for those in younger ages.

We excluded women who were pregnant at the time of examination. We excluded 32,498 participants (<0.1% of all participants) with FPG <2.0 mmol/L or >30.0 mmol/L, or HbA1c <3% or >18%, because these values are likely to reflect measurement or data recording errors. We excluded 254,954 participants with missing medication information (0.2% of all participants); those with and without medication information were similar in their age and BMI (differences of 0.6 years and 0.1 kg/m^2, respectively). In 74 studies (7%), fasting glucose was reported in capillary whole blood. We converted these measurements to plasma-equivalent values using a linear regression equation that quantified the relationship between the two in studies that had measured both whole-blood-based and plasma-based fasting glucose.[10]

Anonymised individual data from the studies in the NCD-RisC database were reanalysed according to a common protocol. We calculated diabetes prevalence and treatment, as defined in the primary outcomes, by sex and age group for each study. When applicable, we used survey sample weights and accounted for complex survey design in calculating summary statistics.

Some studies had asked questions about diabetes treatment from all participants but measured FPG and HbA1c in a subset of participants by design. The reasons for this practice include the different response rates for questionnaire and laboratory parts of a survey, cost of equipment or laboratory analysis, or the logistics of blood collection including fasting requirement for FPG. For example, the US National Health and Nutrition Examination Surveys (NHANES) divided participants into those who visited the data collection sites in the morning or afternoon; only the morning group was instructed to fast and had FPG data. The ICMR-INDIAB surveys in India measured HbA1c for every fifth of participants who did not have a prior diagnosis of diabetes. Both surveys collected data on diabetes medication from all participants.[11,12] In these studies, untreated diabetes was only calculated among those with biomarker measurement (see also Appendix Text 3).

Some studies in our analysis had data on both FPG and HbA1c; others had only one of these biomarkers (Appendix Table 1). In studies with data on both FPG and HbA1c, untreated diabetes was calculated using both biomarkers, i.e. participants with either FPG ≥7.0 mmol/L or HbA1c ≥6.5%. In studies with data on one biomarker only, participants whose measured biomarker was elevated (i.e., FPG ≥7.0 mmol/L in studies that had measured FPG; HbA1c ≥6.5% in studies that had measured HbA1c) were considered to have diabetes. For the remainder of the sample, who neither used treatment nor had elevated level of the measured biomarker, we used the coefficients of previously-validated regressions[8] to estimate the probability of having elevated level of the second (unmeasured) biomarker. These regressions were developed and validated using data from studies that had measured both FPG and HbA1c.[8] The model was specified as a logistic regression, and the predictors included the measured biomarker, age, sex, body-mass index, and region.[8] For example, in a study that measured only FPG, those who used treatment or had FPG ≥7.0 mmol/L were considered to have diabetes; for those who did not use treatment and whose FPG was <7.0 mmol/L, we estimated the probability of having HbA1c ≥6.5% using the coefficients of the aforementioned regression. The probabilities were then summed across individuals to obtain the equivalent

number of people with isolated elevated HbA1c, which was then added to the numerator of prevalence.

Some studies reported diabetes prevalence based on a different definition from the primary definition, e.g., one study used FPG ≥7.8 mmol/L and another used HbA1c ≥10%, or reported mean FPG. Many of these were from a previous global pooling study[4] or extracted from published reports and papers as stated earlier, hence reanalysis of their data was not feasible. This group also included studies with individual participant data that had not collected data on treatment and hence could be used to calculate mean FPG, mean HbA1c or prevalence of people with elevated levels of these markers, but not treated diabetes. We used regressions that converted data from these sources to our primary outcome for diabetes prevalence. The dependent variable in each of these regressions was the primary outcome for diabetes prevalence (prevalence of FPG ≥7.0 mmol/L, HbA1c ≥6.5%, or taking medication for diabetes), and the independent variable was a mean (e.g., mean FPG) or prevalence with a definition that differed from the primary outcome. The coefficients of these regressions were estimated from data sources which could be used to calculate both dependent and independent variables. Details of conversion regressions, and their specification, coefficients and performance, are reported at https://github.com/NCD-RisC/ncdrisc-methods/blob/main/NCD-RisC-conversion-model-for-diabetes-prevalence.pdf.

Appendix Text 3. Statistical methods

Overview

We used a Bayesian hierarchical meta-regression model, fitted using a Markov chain Monte Carlo (MCMC) sampler, with inference made using posterior MCMC samples, to estimate trends in diabetes prevalence and treatment, by sex, age, country, and year. As stated in the main paper, we report trends from 1990 to 2022, a period during which diabetes was recognised as an epidemic and the benefits of treatment were demonstrated in clinical trials.[13,14] Data from 1980 to 1989 were used in the model so that the estimates for 1990 and the subsequent years were informed by as many studies from the early period and from each country as possible. We modelled the two primary outcomes, diabetes prevalence and treatment, separately because their time trends and age associations may be different.

The statistical methods, including its implementation and computation, are detailed in a statistical paper[15] and related substantive papers.[2,16] Model specification is summarised below and further described using statistical notation. This is followed with details of model implementation and computation.

In summary, we organised countries into 20 regions, based on geography and other shared national characteristics, which were further grouped into eight super-regions (Appendix Table 2). The model had a hierarchical structure in which estimates for each country and year were informed by its own data, if available, and by data from other years in the same country and from other countries, especially those in the same region and super-region with data for similar time periods. The extent to which estimates for each country-year were influenced by data from other years and other countries depended on whether the country had data, the sample size of data, whether the available studies were at national, subnational or community level, and the within-country and within-region variability of the available data. Estimates for countries with more national studies, especially with data that were less variable, were informed to a greater degree by its own data than those with fewer studies, especially fewer

national studies, or those with data that varied extensively. At the extreme, for the 25 countries without data, the estimates were informed by data from other countries, especially those in the same region with data for similar time periods, and had larger uncertainty.

The model incorporated non-linear time trends as a combination of linear and second-order random walk terms. Both components were modelled hierarchically. The age association of diabetes was modelled using a cubic spline to allow non-linear age patterns, which might vary across countries. The coefficients of the splines were modelled hierarchically, and the coefficients are allowed to vary over time to reflect changing time trends in diabetes prevalence and treatment across ages, if supported by data.[2,16]

The model accounted for the possibility that diabetes prevalence and treatment in subnational and community studies might systematically differ from, and have larger variation than, nationally representative samples through the inclusion of fixed-effect and random-effect terms. The fixed effects allowed for systematic differences between subnational or community studies and national studies and allowed for these differences to vary over time. The random effects allowed national data to have a larger influence on the estimates than subnational or community data with similar sample sizes. The model also accounted for urban-rural differences in diabetes prevalence and treatment through fixed effect terms for urban-only and rural-only studies, so that our estimates were for the entire population of each country. These urban and rural effects were weighted by the difference between study-level and country-level urbanisation (i.e., proportion of population living in urban areas) in the year when the study was conducted and were also permitted to vary over time.

As stated in the Methods section of the main paper, we analysed diabetes prevalence for people aged 18+ years and treatment for people aged 30+ years. We performed all analyses separately by sex, because levels and trends in diabetes prevalence and treatment may be different by sex.[1]

Detailed model specification

As explained in *Overview*, we modelled the two primary outcomes separately. Here we describe the model in detail for diabetes prevalence. The model for diabetes treatment is described in the section *Model specification for treatment coverage*.

For diabetes prevalence, we used a latent variable $\alpha_{h,i} = \Phi^{-1}(prevalence_{h,i})$ which is the probit-transformed diabetes prevalence $(prevalence_{h,i})$ for age group h of study i. Φ is the cumulative distribution function of the standard normal distribution and referred to as probit transformation. This specification constrains the primary outcome to be between 0 and 1. We modelled $\alpha_{h,i}$ with a Gaussian distribution

$$\alpha_{h,i} \sim N\left(a_{j[i]} + b_{j[i]}t[i] + u_{j[i],t[i]} + \gamma_{j[i],t[i]}(z_h) + \boldsymbol{X_i\beta} + e_i, \tau^2 + \upsilon_{h,i}^2\right), \quad (1)$$

where j, the country in which a study was carried out, and t, the study year, are uniquely determined by the study index i; we denote this determination of j and t on i by $j[i]$ and $t[i]$ respectively. The country-specific intercept and linear time slope from country j are denoted a_j and b_j respectively, with $j \in \{1,..J\}$, where J = 200 is the number of countries in our analysis. We describe the hierarchical model used for the linear component of country time trends, a's and b's, in the section *Linear component of country time trends*. $u_{j,t}$ captures smooth non-linear change over time in country j, as described in the section *Nonlinear change*. $\gamma_{j,t}(z_h)$ is the age effect for age group h (with mid-age z_h) in year t in country j; it is described in detail in the section *Age model*. The matrix $\boldsymbol{X}$ contains terms describing whether studies were representative at the national, subnational or community level, and whether they were urban-only, rural-only, or covered both urban and rural populations, and $\boldsymbol{\beta}$ contains the associated fixed effects. In addition, a random effect e_i is estimated for each study i. These study-specific terms are described in the section *Study-level terms and study-specific random effects*. The variance term $\upsilon_{h,i}^2$ in the model accounts for the uncertainty arising from using regression models to estimate diabetes prevalence based on the primary definition. The variance term

τ^2 captures the variability not accounted for by the study-specific random effects. These are described in the section *Additional age-by-study variability*. Details on model fitting and convergence are given in the section *Model implementation.* Finally, details on how country-level inference was performed are given in the section *Model inference and post-processing.*

The studies used to fit the model provided data on the number (and hence prevalence) of people with diabetes in a sample. Each study contributed up to 13 data points for each sex, with the exact number depending on the age groups represented in the study. If a study collected data on glycaemic markers like FPG or HbA1c and diabetes medication information for all participants, then the number of people with diabetes $y_{h,i}$, from age group h of study i, is assumed to be an observation taken from a binomial distribution with sample size $n_{h,i}$ and prevalence $prevalence_{h,i}$,

$$y_{h,i} \sim Bin(n_{h,i},\ prevalence_{h,i}), \qquad (2)$$

with $prevalence_{h,i}$ linking to the latent variable $\alpha_{h,i}$ modelled in equation (1).

As stated in Appendix Text 2, some studies collected data on diabetes medication in all participants but measured glycaemic markers only in a subset of participants. This approach to data collection means that the prevalence of treated diabetes was based on the entire sample, whereas untreated diabetes (i.e., people who were not treated but had elevated levels of FPG or HbA1c) was based on the subset with biomarker data, with the two groups having different sample sizes.

With such survey design, the number of participants with diabetes is the sum of two separately observed quantities: treated diabetes ($y_{h,i}^{treated}$) and untreated diabetes ($y_{h,i}^{untreated}$), obtained from their own respective samples. Each is assumed to be an observation taken from a binomial distribution:

$$y_{h,i}^{treated} \sim Bin(n_{h,i}^{treated},\ prevalence_{h,i}^{treated}), \qquad (3)$$

$$y_{h,i}^{untreated} \sim Bin\left(n_{h,i}^{untreated},\ proportion_{h,i}^{untreated}\right), \quad (4)$$

where $prevalence_{h,i}^{treated}$ is the prevalence of treated diabetes, $proportion_{h,i}^{untreated}$ is the proportion of participants who do not use treatment with FPG ≥7.0 mmol/L or HbA1c ≥6.5%. Their respective sample sizes are $n_{h,i}^{treated}$, the number of participants with data on diabetes medication, and $n_{h,i}^{untreated}$, the number of untreated participants who had data on glycaemic markers. Prevalence of total diabetes can then be calculated as follows:

$$p_{h,i} = prevalence_{h,i}^{treated} + \left(1 - prevalence_{h,i}^{treated}\right) \times proportion_{h,i}^{untreated}. \quad (5)$$

833 (75%) studies (referred to as group A hereafter) had data available for treated and untreated diabetes. Data from all group A studies were used in equations (3) and (4) in the model, to allow for the aforementioned potentially different sample sizes for treated and untreated diabetes. The remaining 275 (25%) studies (group B) had data for prevalence of total diabetes, but not for treated versus untreated diabetes. Data from group B studies were used in equation (2) in the model.

In equation (3), we used the number of participants who answered the questionnaire about medication use as sample size for treated diabetes ($n_{h,i}^{treated}$), and in equation (4) we used those participants who had answered “no” to this question and had a biomarker measurement as sample size for untreated diabetes ($n_{h,i}^{untreated}$). In studies with complex survey design, effective sample size is smaller than actual sample size because sampling is done in clusters and/or with stratification. We used actual sample size and did not use effective sample size, which was used in previous analyses,[17] because there is no standard way to calculate effective sample size for the untreated group in equation (4), itself because the untreated group relies on data from a subset of participants (those without treatment and with biomarker measurement).

We used an additional latent variable in calculating $prevalence_{h,i}^{treated}$ and $proportion_{h,i}^{untreated}$, namely the probit-transformed treatment coverage $\zeta_{h,i} = \Phi^{-1}(treatment_{h,i})$ where $treatment_{h,i}$ is treatment coverage, our second primary outcome, for age group h of study i. This latent variable allows us to consistently model both of our primary outcomes (prevalence and treatment) as stated in the section *Model specification for treatment coverage*. $treatment_{h,i}$ has the following relationship with prevalence of diabetes and treated diabetes:

$$prevalence_{h,i}^{treated} = prevalence_{h,i} \times treatment_{h,i}. \quad (6)$$

All prevalence terms can be expressed using the two latent variables $\alpha_{h,i}$ and $\zeta_{h,i}$:

$$prevalence_{h,i} = \Phi(\alpha_{h,i}), \quad (7)$$

$$prevalence_{h,i}^{treated} = \Phi(\alpha_{h,i})\,\Phi(\zeta_{h,i}), \quad (8)$$

$$proportion_{h,i}^{untreated} = \frac{\Phi(\alpha_{h,i}) - \Phi(\alpha_{h,i})\,\Phi(\zeta_{h,i})}{1 - \Phi(\alpha_{h,i})\,\Phi(\zeta_{h,i})}. \quad (9)$$

In the model for diabetes prevalence, we modelled the latent variable $\alpha_{h,i}$ as described in equation (1) and detailed in the following sections, and we used a normal prior $\mathcal{N}(0,1)$ for the latent variable $\zeta_{h,i}$ which is equivalent to an uninformative uniform distribution on [0, 1] in the prevalence scale.

Linear component of country time trends

The model had a hierarchical structure, whereby studies were nested in countries, which were nested in regions (indexed by *l*), which were nested in super-regions (indexed by m), which were all nested in the globe (see Appendix Table 2 for a list of countries and territories in each region, and regions in each super-region). This structure allowed the model to share information across units to a greater degree when data were non-existent or weakly

informative (for example, had a small sample size or were not nationally representative) and, to a lesser extent, in data-rich countries and regions.[18]

The a and b terms are country-specific linear intercepts and time slopes with terms at each level of the hierarchy, denoted by the superscripts *c*, *r*, *s* and *g*, respectively:

$$a_j = a_j^c + a_{l[j]}^r + a_{m[j]}^s + a^g, \tag{10}$$

$$b_j = b_j^c + b_{l[j]}^r + b_{m[j]}^s + b^g, \tag{11}$$

$$a^x \sim N(0, \kappa_a^x), \tag{12}$$

$$b^x \sim N(0, \kappa_b^x), \tag{13}$$

where $x \in \{c, r, s\}$. The κ terms were each assigned a flat prior on the standard deviation scale.[18] We also assigned flat priors to a^g and b^g.

Nonlinear change

The prevalence of diabetes may change nonlinearly over time.[1] We captured smooth nonlinear change in time in country j using the vector u_j. Just as a_j and b_j are each defined as the sum of country, region, super-region and global components, we defined

$$u_j = u_j^c + u_{l[j]}^r + u_{m[j]}^s + u^g. \tag{14}$$

To allow the model to differentiate between the degrees of nonlinearity that exist at the country, region, super-region and global levels, we assigned the four components of each u a discrete second-order Gaussian autoregressive prior.[19] In particular, the vectors $u_j^c, j \in \{1, \dots, J\}$, $u_l^r, l \in \{1, \dots, L\}$, $u_m^s, m \in \{1, \dots, M\}$, and u^g, all of length T, are each given a Gaussian prior with mean zero and precision $\lambda_c P$, $\lambda_r P$, $\lambda_s P$ and $\lambda_g P$ respectively, where the scaled precision matrix *P* in the Gaussian autoregressive prior penalizes first and second differences as follows:

$$P = \begin{bmatrix} 1 & 0 & 0 & \cdots & 0 \\ -2 & 1 & 0 & \cdots & 0 \\ 1 & -2 & 1 & \cdots & 0 \\ 0 & 1 & -2 & \cdots & 0 \\ 0 & 0 & 1 & \cdots & 0 \\ \vdots & \vdots & \vdots & \ddots & \vdots \\ 0 & 0 & 0 & \cdots & 1 \end{bmatrix} \begin{bmatrix} 1 & -2 & 1 & 0 & 0 & \cdots & 0 \\ 0 & 1 & -2 & 1 & 0 & \cdots & 0 \\ 0 & 0 & 1 & -2 & 1 & \cdots & 0 \\ \vdots & \vdots & \vdots & \vdots & \vdots & \ddots & \vdots \\ 0 & 0 & 0 & 0 & 0 & \cdots & 1 \end{bmatrix}$$

$$= \begin{bmatrix} 1 & -2 & 1 & 0 & 0 & \cdots & 0 \\ -2 & 5 & -4 & 1 & 0 & \cdots & 0 \\ 1 & -4 & 6 & -4 & 1 & \cdots & 0 \\ 0 & 1 & -4 & 6 & -4 & \cdots & 0 \\ 0 & 0 & 1 & -4 & 6 & \cdots & 0 \\ \vdots & \vdots & \vdots & \vdots & \vdots & \ddots & \vdots \\ 0 & 0 & 0 & 0 & 0 & \cdots & 1 \end{bmatrix}. \qquad (15)$$

P is multiplied by the estimated precision parameters λ_c, λ_r, λ_s, and λ_g, thus upweighting or downweighting the strength of its penalties and ultimately determining the degree of smoothing at each level. For each of the four precision parameters, we used a truncated flat prior on the standard deviation scale ($1/\sqrt{\lambda}$).[18] We truncated these priors such that $\log\lambda \leq 20$ for each of the four $\lambda's$. This upper bound is enforced as a computational convenience, whereby models with $\log\lambda > 20$ are treated as equivalent to a model with $\log\lambda = 20$, as they essentially have no extra-linear variability in time. In practice, this upper bound had little effect on the parameter estimates. Furthermore, we ordered the $\lambda's$ a priori as follows: $\lambda_c < \lambda_r < \lambda_s < \lambda_g$. This prior constraint conveys the expectation that the global trend in the prevalence of diabetes has less extra-linear variability than the trend of any given super-region, which has less than those of constituent regions, which in turn has less variability than the trends of constituent countries.

The matrix P has rank $T-2$, corresponding to a flat, improper prior on the mean and the slope of the u_j^c's, the u_l^r's, the u_m^s's and u^g, and is not invertible.[20] Thus, we had a proper prior in a reduced-dimension space,[21] with the prior expressed as follows:

$$P(u_j^c|\lambda_c) \propto \lambda_c^{\frac{T-2}{2}} \exp\left\{-\frac{\lambda_c}{2} u_j^{c\prime} P u_j^c\right\}. \quad (16)$$

Note that if u_j^c had a non-zero mean, this would introduce non-identifiability with respect to a_j^c. By the same token, b_j^c would not be identifiable if u_j^c had a non-zero time slope, and similarly for the other means and slopes. Thus, to achieve identifiability of the a's, b's, and u's, we constrained the mean and slope of u^g and each of u^s, u^r, and u^c to be zero. Enforcing orthogonality between the linear and nonlinear portions of the time trends meant that each can be interpreted independently.

For the countries in which there are observations for at least two different time points, this improper prior will not lead to an improper posterior because the data provide information about the mean and slope. In order to enforce the desired orthogonality between the linear and nonlinear portions of the model, we used the Rue and Held correction.[21] For the countries without data (25 for women and 26 for men), we took the Moore-Penrose pseudoinverse of P,[22] setting to infinity those eigenvalues that correspond to the non-identifiability. This effectively constrained the non-identified portions of the model to zero, as the corresponding variances are set to zero;[19] in this case the Rue and Held correction[21] is not needed. An intermediate case occurs when data are observed for only one time point in a country. In this case, the full conditional precision has rank $T-1$ because the mean but not the linear trend of u_j^c is identified by the data. We therefore constrained the linear trend of u_j^c to zero in this case, by taking the generalized inverse of the full conditional precision. We then constrained the mean of u_j^c to zero using the one-dimensional version of the Rue and Held correction.[21] Computational details are given in the Appendix of a previous paper.[23]

Age model

We sought a smooth function that could characterise gradual changes in diabetes over age, as seen in the data, with parsimonious number of parameters to allow robust estimation. To

achieve this, we modelled age using cubic splines with two knots selected based on epidemiological knowledge about diabetes[24] and statistical considerations.

For age group h with mid-age z_h, in study i, conducted in year *t* and country *j*, the age term is given by

$$\gamma_{j[i],t[i]}(z_h) = \gamma_{1,i} z_h + \gamma_{2,i} z_h^2 + \gamma_{3,i} z_h^3 + \gamma_{4,i}(z_h - k_1)_+^3 + \gamma_{5,i}(z_h - k_2)_+^3, \qquad (17)$$

where the two knots were placed ages $(k_1, k_2) = (50, 65)$ years. To reduce dependence among model parameters, we centred the age variable at 55 years.

Each of the spline coefficients was allowed to vary across countries and was modelled hierarchically, and was further allowed to vary across time, in order to reflect different trends in prevalence across age groups. We modelled spline coefficients consistently with previous analysis,[2,16] with the *k*th age term coefficients for study *i* given as follows:

$$\gamma_{k,i} = \psi_k^g + \psi_{k,j[i]}^c + \psi_{k,l[i]}^r + \psi_{k,m[i]}^s + \left(\phi_k^g + \phi_{k,j[i]}^c + \phi_{k,l[i]}^r + \phi_{k,m[i]}^s\right) t[i], \qquad (18)$$

$$\psi_{k,j[i]}^c \sim N\left(0, \sigma_{\psi,k,c}^2\right), \qquad (19)$$

$$\psi_{k,l[i]}^r \sim N\left(0, \sigma_{\psi,k,r}^2\right), \qquad (20)$$

$$\psi_{k,m[i]}^s \sim N\left(0, \sigma_{\psi,k,s}^2\right), \qquad (21)$$

$$\phi_{k,j[i]}^c \sim N\left(0, \sigma_{\phi,k,c}^2\right), \qquad (22)$$

$$\phi_{k,l[i]}^r \sim N\left(0, \sigma_{\phi,k,r}^2\right), \qquad (23)$$

$$\phi_{k,m[i]}^s \sim N\left(0, \sigma_{\phi,k,s}^2\right). \qquad (24)$$

Here ψ^g, ψ^c, ψ^r, and ψ^s are global, country, region, and super-region intercepts, and ϕ^g, ϕ^c, ϕ^r, and ϕ^s are global, country, region and super-region time slope parameters. A flat improper prior was placed on each of the σ_ψ's and σ_ϕ's.

Study-level terms and study-specific random effects

The prevalence of diabetes as measured in individual studies may differ from the true unobserved country-year prevalence due to study implementation factors such as those associated with sampling, participation and response, and measurement. We included time-varying offsets (referred to above as fixed effects) to help account for potential systematic differences associated with data sources that are representative of subnational or community populations, and data sources that are representative of urban-only or rural-only populations, through the terms in $\boldsymbol{X_i\beta}$:

$$\begin{aligned}\boldsymbol{X_i\beta} = {} & \beta_1\mathbf{I}\{\boldsymbol{X}_i^{cvrg} = subnational\} + \beta_2\mathbf{I}\{\boldsymbol{X}_i^{cvrg} = subnational\}t[i] \\ & + \beta_3\mathbf{I}\{\boldsymbol{X}_i^{cvrg} = community\} + \beta_4\mathbf{I}\{\boldsymbol{X}_i^{cvrg} = community\}t[i] \\ & + \beta_5\boldsymbol{X}_{j[i],t[i]}^{c.urb}\mathbf{I}\{\boldsymbol{X}_i^{s.urb} = rural\} + \beta_6\mathrm{X}_{\mathrm{j[i]},t[i]}^{\mathrm{c.urb}}\mathbf{I}\{\boldsymbol{X}_i^{s.urb} = rural\}t[i] \\ & + \beta_7(1 - \boldsymbol{X}_{j[i],t[i]}^{c.urb})\mathbf{I}\{\boldsymbol{X}_i^{s.urb} = urban\} + \beta_8(1 - \boldsymbol{X}_{j[i],t[i]}^{c.urb})\mathbf{I}\{\boldsymbol{X}_i^{s.urb} = urban\}t[i],\end{aligned} \tag{25}$$

where $\boldsymbol{X}_i^{cvrg}$ is the indicator for whether the coverage of study $i,$ in country j and year $t,$ is subnational or community, $\boldsymbol{X}_i^{s.urb}$ is the indicator for whether the study i covered rural-only or urban-only populations, and $\boldsymbol{X}_{j[i],t[i]}^{c.urb}$ is the percentage of the national population of country j in year t living in urban areas, as obtained from the 2018 revision to the United Nation's World Urbanization Prospects.[25] We note that β_5 through β_8 are all multiplied by zero for studies which are urban-only in countries where all residents lived in urban areas (e.g., Singapore) and for studies which are rural-only in countries where all residents lived in rural areas (e.g., Tokelau), i.e., in such cases the model does not consider studies classified as urban (respectively rural) to have potential systematic differences from the true underlying prevalence in the country.

Even after accounting for sampling variability, national studies may still not reflect the true prevalence of diabetes in a country with perfect accuracy, due to factors related to response and measurement, and subnational and community studies have even larger variability. We include the study-specific random effect e_i to allow all age groups from the same study to have an unusually high or an unusually low prevalence, after conditioning on the other terms in the

model. Each e_i is assigned a Gaussian prior with variance dependent on whether study i is representative at the national, subnational or community level. Random effects from national studies were constrained to have smaller variance (ν_n) than random effects of subnational studies (ν_s), which were in turn constrained to have smaller variance than community studies (ν_c).

Additional age-by-study variability

The additional variance term $\upsilon_{h,i}^2$ in the model accounts for the additional variability arising from our use of regression models to estimate diabetes prevalence based on the primary definition for two types of studies described under *Data cleaning and management* section of Appendix Text 2. The first are the studies that only measured one glycaemic marker, for which we used previously-validated conversion regressions to obtain prevalence based on the primary definition.[8] The second are those that only had data for diabetes prevalence based on a different definition or for mean FPG or mean HbA1c, for which we used regression models to convert to diabetes prevalence based on the primary definition. This additional variance term is fixed for each data point and calculated using a simulation approach. Specifically, we sampled 2,000 draws from the joint distribution of the regression coefficients. We used each draw of regression coefficients to repeatedly calculate diabetes prevalence based on the primary definition. We then calculated $\upsilon_{h,i}^2$ term as the variance of the 2,000 probit-transformed diabetes prevalence, by study, sex and age group. The $\upsilon_{h,i}^2$ term is zero when the primary outcome was calculated directly from the data.

Finally, the age patterns across communities within a given country may differ from the overall age pattern of that country. This within-study variability cannot be captured by the e_i terms, which are equal across age-specific observations in each study, so we included an additional variance component for each study, τ^2.

Model specification for treatment coverage

As mentioned above, the parameters in the models for diabetes prevalence and diabetes treatment were estimated independently. In the model for treatment coverage, we modelled the latent variable $\zeta_{h,i}$, the probit-transformed treatment coverage, in the same way as we modelled the latent variable $\alpha_{h,i}$ for diabetes prevalence. We modelled $\zeta_{h,i}$ with a Gaussian distribution:

$$\zeta_{h,i} \sim N\left(a_{j[i]} + b_{j[i]}t[i] + u_{j[i],t[i]} + \gamma_i(z_h) + \boldsymbol{X_i\beta} + e_i, \tau^2 + \nu_{h,i}^2\right), \quad (26)$$

with the components having the same definitions as above. Similar to the model for diabetes prevalence, the additional variance term $\upsilon_{h,i}^2$ accounts for the uncertainty from our use of regression models to estimate diabetes prevalence based on the primary definition which appears in the denominator for treatment coverage. We applied the normal prior $\mathcal{N}(0,1)$ to the other latent variable $\alpha_{h,i}$, with this prior again being equivalent to an uninformative uniform distribution on [0, 1] in the prevalence scale. Only group A studies were used to fit this model because group B studies did not contain data on treatment.

Model implementation

The model was fitted through a bespoke MCMC sampler coded in R, which uses a combination of Metropolis-Hastings and Gibbs updates.[26] Details of the approach for generating starting values were given in a previous paper.[2]

We had a target of eight converged MCMC chains for generating our estimates, which is twice the recommended minimum number to assess convergence using the Rhat diagnostic.[27,28] We ran ten chains for each outcome (prevalence, treatment) and sex combination, with chains ordered by their seeds. The additional two chains per outcome and sex were run to allow for a small number of the first eight chains to be discarded if mixing was slow. In practice, only five of the 32 chains were slow to converge and were replaced. We did not run more chains because the computational and time cost outweighed the gains, if any, in results. We identified,

through visual inspection of hyperparameter trace plots, a burn-in period of 25,000 iterations for diabetes prevalence, and 100,000 for diabetes treatment because treatment models required more iterations for convergence. We took 50,000 post-burn-in iterations from each of the eight target chains, and combined and thinned to obtain a final sample of 5,000 posterior draws for each outcome. Convergence was confirmed through visual inspection as well as through calculated split-Rhat diagnostic for country-year-age outcomes as implemented in the R package 'rstan' v2.26.15.[27,29] The 97.5th quantile of split-Rhat for the two primary outcomes and two sexes ranged from 1.005 to 1.066. 99% of country-year-age combinations for the two primary outcomes and two sexes had split-Rhat <1.05.

Model inference and post-processing

All inference was done for country-year-age combinations, through combining the a, b, u, and γ terms, and setting $\beta = e_i = 0$. We set $\beta = 0$ as fixed effects associated with study design are not relevant for country-level inference. We set $e_i = 0$ as random effects arising from imperfections and variations in study design and implementation, and from within-country variability of the primary outcomes, are also not relevant for country-level inference.

Posterior estimates were made in five-year age groups. For presentation, we summarised the age-specific results as age-standardised results. Age-standardisation puts the population for each country-year on the same (standard) age distribution, and hence enables comparisons to be made over time and across countries. Age-standardisation was performed by taking the weighted means of age-sex-specific estimates, using age weights from the WHO standard population.[30] Estimates for regions and the world were calculated as population-weighted averages of the constituent country estimates by sex and age group, using population data obtained from the United Nations' World Population Prospects (2024 revision).[31] Consistent with analysis of hypertension treatment,[16] when calculating age-standardised treatment we also accounted for the age pattern of diabetes prevalence, because the denominator of treatment is only people with diabetes, by multiplying the WHO standard population weights

with age-specific diabetes prevalence in each country and year. The number of adults who had diabetes or untreated diabetes were calculated by multiplying the corresponding age-specific prevalence by the age-specific population by sex, country, and year.

The uncertainties of our estimates, represented by their posterior distributions, include the following sources: uncertainty due to sampling in each data source; uncertainty associated with the variability of national data beyond what is accounted for by sampling; uncertainty associated with subnational and community data, which are more variable than national data; uncertainty associated with using regression equations to estimate the primary outcomes; and uncertainty due to making estimates by country, year, and age when data were missing, scarce or weakly informative. The reported credible intervals (CrI) represent the 2.5th to 97.5th percentiles of the posterior distributions, which contain the true estimates with 95% probability. We obtained the posterior probability (PP) that an estimated change in diabetes prevalence or treatment coverage represented a true increase as the proportion of draws from the posterior distribution that indicated an increase, i.e., a positive change.

Appendix Text 4. Decomposition of change in the number of people with untreated diabetes

The number of people with untreated diabetes in 1990 can be written as:

$$N_{1990} = P_{1990} \times DP_{1990} \times (1 - TC_{1990}),$$

where N_{1990} is the number of people with untreated diabetes in 1990, P_{1990} is the population in 1990, DP_{1990} is diabetes prevalence in 1990, and TC_{1990} is treatment coverage in 1990. Similarly for a later year *year2*:

$$N_{year2} = P_{year2} \times DP_{year2} \times (1 - TC_{year2}).$$

Total change in N from 1990 to year2 is the subtraction of the above two numbers, as below:

$$\textit{Total change in N} = N_{year2} - N_{1990}$$

$$= P_{year2} \times DP_{year2} \times (1 - TC_{year2}) - P_{1990} \times DP_{1990} \times (1 - TC_{1990}).$$

Algebraically, *Total change in N* can be written as the sum of the *Contribution attributed to change in P*, the *Contribution attributed to change in DP*, the *Contribution attributed to change in TC*, and a fourth term that represents the residual change after accounting for the three named contributions. The first three terms are defined as below, with the residual being the difference between the total change and the sum of the first three:

Contribution attributed to change in P

$$= (P_{year2} - P_{1990}) \times DP_{1990} \times (1 - TC_{1990}),$$

Contribution attributed to change in DP

$$= P_{1990} \times (DP_{year2} - DP_{1990}) \times (1 - TC_{1990}),$$

Contribution attributed to change in TC

$$= P_{1990} \times DP_{1990} \times [(1 - TC_{year2}) - (1 - TC_{1990})],$$

Residual term

$$= P_{year2} \times DP_{year2} \times (1 - TC_{year2}) - P_{year2} \times DP_{1990} \times (1 - TC_{1990})$$

$$- P_{1990} \times DP_{year2} \times (1 - TC_{1990}) - P_{1990} \times DP_{1990} \times (1 - TC_{year2})$$

$$+ 2 \times P_{1990} \times DP_{1990} \times (1 - TC_{1990}).$$

Appendix Table 1. Data sources used in the analysis.

	Country	Data years	Survey/Study name/Citation	Level of representative-ness	Rural, urban, or both	Age range as used for global analysis		Sample size used for global analysis		Glycaemic markers available	Information available on diabetes treatment	Whether measured in a laboratory or by a point-of-care portable device		Notes
						Female	Male	Female	Male			Glucose	HbA1c	
1	Afghanistan	2018	STEPS	National	both	18-69	18-69	1,727	1,993	Fasting glucose	Yes	Portable		
2	Algeria	2001	Temmar et al., J Hpertens 25:2218-26, 2007	Community	rural	35+	35+	655	561	Fasting glucose	No	Unknown		
3	Algeria	2003	STEPS	Subnational	both	25-64	25-64	2,451	1,613	Fasting glucose	Yes	Unknown		
4	Algeria	2005	Transition and Health Impact in North Africa	National	both	35-70	35-70	2,770	2,002	Fasting glucose	Yes	Portable		
5	Algeria	2007-2009	The ISOR (InSulino-resistance in ORan) Study	Community	urban	30-64	30-64	408	375	Fasting glucose	Yes	Lab		
6	Algeria	2016-2017	STEPS	National	both	18-69	18-69	3,690	3,022	Fasting glucose	Yes	Portable		
7	American Samoa	1994	McGarvey, Pac Health Dialog 8(1):157-62, 2001	National	both	29+	29+	247	165	Fasting glucose	Yes	Lab		
8	American Samoa	2004	STEPS	National	both	25-64	25-64	1,061	945	Fasting glucose	Yes	Portable		
9	Angola	2013-2014	CardioBengo - Population based cardiovascular longitudinal study in Bengo Province, Angola	Community	both	18-65	18-65	1,348	768	Fasting glucose	Yes	Portable		
10	Argentina	2004-2005	CArdiovascular Risk factors Multiple Evaluation in Latin America (CARMELA)	Community	urban	25-64	25-64	748	734	Fasting glucose	Yes	Lab		
11	Argentina	2011-2012	CESCAS Study	Community	urban	35-74	35-74	2,362	1,576	Fasting glucose	Yes	Lab		
12	Argentina	2018	Encuesta Nacional de Factores de Riesgo 2018	National	both	18+	18+	15,406	11,925	Fasting glucose	Yes	Unknown		
13	Armenia	2016	STEPS	National	both	18-69	18-69	1,582	736	Fasting glucose	Yes	Portable		
14	Australia	1981	APCSC-Busselton	Community	urban	25+	25+	701	608	Fasting glucose	No	Unknown		
15	Australia	1981	Glatthaar et al., Med J Aust 143:436-40, 1985	Community	both	25+	25+	1,739	1,457	Fasting glucose	No	Unknown		
16	Australia	1983	Risk Factor Prevalence Study	National	urban	25-64	25-64	3,811	3,731	Fasting glucose	Yes	Unknown		
17	Australia	1988-1989	Dubbo Study of Australian Elderly	Community	urban	59+	59+	1,222	878	Fasting glucose	Yes	Lab		
18	Australia	1999-2000	The Australian Diabetes, Obesity and Lifestyle Study 1999-2000	National	both	25+	25+	6,138	5,043	Fasting glucose, HbA1c	Yes	Lab	Lab	
19	Australia	1999-2003	North West Adelaide Health Study	Community	urban	18+	18+	2,089	1,891	Fasting glucose, HbA1c	No	Lab	Lab	
20	Australia	2004-2005	The Australian Diabetes, Obesity and Lifestyle Study 2004-2005	National	both	30+	30+	3,438	2,890	Fasting glucose, HbA1c	Yes	Lab	Lab	
21	Australia	2004-2006	North West Adelaide Health Study	Community	urban	20+	20+	1,665	1,498	Fasting glucose, HbA1c	No	Lab	Lab	
22	Australia	2008-2010	North West Adelaide Health Study	Community	urban	24+	24+	1,277	1,142	Fasting glucose, HbA1c	No	Lab	Lab	
23	Australia	2012	The Australian Diabetes, Obesity and Lifestyle Study 2012	National	both	37+	37+	2,480	2,029	Fasting glucose, HbA1c	Yes	Lab	Lab	
24	Australia	2011-2012	National Health Measure Survey	National	both	18+	18+	4,153	3,320	Fasting glucose, HbA1c	No	Lab	Lab	
25	Austria	1985	VHM&PP; Ulmer et al., J Intern Med 261:566-76, 2007	Subnational	both	20+	20+	42,176	32,600	Fasting glucose	No	Unknown		
26	Austria	1991	CINDI survey Vorarlberg/Austria	Subnational	both	25-64	25-64	736	695	Fasting glucose	Yes	Lab		
27	Austria	1992	Vorarlberg Health Monitoring and Promotion Programme (VHM&PP)	Subnational	both	18+	18+	18,769	14,104	Fasting glucose	No	Lab		
28	Austria	1998	Vorarlberg Health Monitoring and Promotion Programme (VHM&PP)	Subnational	both	18+	18+	20,902	16,140	Fasting glucose	No	Lab		
29	Austria	1998-1999	CINDI survey Vorarlberg/Austria	Subnational	both	25-64	25-64	88	86	Fasting glucose	Yes	Lab		
30	Austria	2004	Vorarlberg Health Monitoring and Promotion Programme (VHM&PP)	Subnational	both	18+	18+	23,890	20,159	Fasting glucose	No	Lab		
31	Austria	2010-2012	Austrian Study on Nutritional Status 2012	National	both	18-80	18-80	278	176	Fasting glucose, HbA1c	No	Lab	Lab	
32	Azerbaijan	2017	STEPS	National	both	18-69	18-69	1,642	1,136	Fasting glucose	Yes	Portable		
33	Bahamas	2019	STEPS	National	both	18-69	18-69	1,403	932	Fasting glucose	Yes	Portable		
34	Bahrain	2007	STEPS	National	both	20-64	20-64	906	863	Fasting glucose	No	Lab		
35	Bangladesh	2002	Hussain et al., Diabet Med 22:931-6, 2005	Community	rural	20+	20+	2,720	2,037	Fasting glucose	No	Unknown		
36	Bangladesh	2002	Hussain et al., Diabet Med 22:931-6, 2005	Community	urban	20+	20+	824	731	Fasting glucose	No	Unknown		
37	Bangladesh	2006	Urban Health Survey	Subnational	urban	35-59	35-59	1,272	1,520	Fasting glucose	Yes	Portable		
38	Bangladesh	2011	Demographic and Health Survey Bangladesh 2011	National	both	35+	35+	3,572	3,753	Fasting glucose	Yes	Portable		
39	Bangladesh	2011-2012	Chronic Disease Risk Factor Study	Community	rural	18+	18+	427	292	Fasting glucose	Yes	Lab		
40	Bangladesh	2016	Diabetes Mellitus: Action through community Groups or Health Information for better Control of population blood glucose, risk factors, knowledge and care seeking (DMagic)	Subnational	rural	30+	30+	6,414	5,630	Fasting glucose	Yes	Portable		
41	Bangladesh	2017-2018	Demographic and Health Survey Bangladesh 2017-2018	National	both	18-49		4,858		Fasting glucose	Yes	Portable		
42	Bangladesh	2018	STEPS	National	both	18-69	18-69	3,702	3,247	Fasting glucose	No	Lab		
43	Barbados	1992	Foster et al., Ethn Dis 3:404-12, 1993	Community	both	40+	40+	272	188	Fasting glucose	No	Unknown		
44	Barbados	1997-2002	The Barbados Incidence Studies of Eye Diseases II	National	both	40-59	40-59	840	606	HbA1c	No		Unknown	
45	Barbados	2011-2013	Health of the Nation (HotN)	National	both	25+	25+	741	469	Fasting glucose, HbA1c	Yes	Lab	Portable	
46	Belarus	2016-2017	STEPS	National	both	18-69	18-69	2,897	2,089	Fasting glucose	Yes	Portable		
47	Belarus	2020	STEPS	National	both	18-69	18-69	2,990	2,280	Fasting glucose	Yes	Portable		
48	Belgium	1991-1994	Flemish Study on Environment, Genes and Health Outcomes	Community	rural	26+	26+	412	397	Fasting glucose	Yes	Lab		
49	Belgium	2003	The European Male Ageing Study	Community	both		40+		447	Fasting glucose	Yes	Lab		
50	Belgium	2005-2008	Flemish Study on Environment, Genes and Health Outcomes	Community	rural	18+	18+	357	346	Fasting glucose	Yes	Lab		
51	Belgium	2006-2008	Flemish Study on Environment, Genes and Health Outcomes	Community	rural	18+	18+	83	97	Fasting glucose	Yes	Lab		
52	Belgium	2008	The European Male Ageing Study	Community	both		45+		372	Fasting glucose	Yes	Lab		
53	Belgium	2009-2013	Flemish Study on Environment, Genes and Health Outcomes	Community	rural	20+	20+	335	330	Fasting glucose	Yes	Lab		
54	Belgium	2010-2015	Flemish Study on Environment, Genes and Health Outcomes	Community	rural	18+	18+	410	389	Fasting glucose	Yes	Lab		
55	Belgium	2018-2019	Belgian Health Examination Survey	National	both	18+	18+	614	557	Fasting glucose, HbA1c	Yes	Lab	Lab	1
56	Belize	2005-2006	CAMDI	National	both	20+	20+	1,021	600	Fasting glucose	Yes	Lab		
57	Benin	2007	STEPS	Community	urban	25-64	25-64	1,508	955	Fasting glucose	Yes	Portable		
58	Benin	2008	STEPS	National	both	25-64	25-64	3,391	3,442	Fasting glucose	Yes	Portable		
59	Benin	2015	STEPS	National	both	18-69	18-69	2,547	2,307	Fasting glucose	Yes	Portable		
60	Bhutan	2007	STEPS	Community	urban	25-74	25-74	1,330	1,132	Fasting glucose	Yes	Lab		
61	Bhutan	2014	STEPS	National	both	18-69	18-69	1,682	1,072	Fasting glucose	Yes	Portable		
62	Bhutan	2019	STEPS	National	both	18-69	18-69	3,280	2,099	Fasting glucose	Yes	Portable		
63	Bolivia	2005-2007	Cardiovascular and metabolic syndrome risk assessment of Bolivian school children and adolescents - Relationships to obesity, diabetes, income, food intake and physical activity	National	both	18	18	139	144	Fasting glucose	Yes	Lab		
64	Bosnia and Herzegovina	2012	Non-communicable disease risk factor survey, Federation of B&H	Subnational	rural	18+	18+	1,288	1,201	Fasting glucose	Yes	Portable		

	Country	Data years	Survey/Study name/Citation	Level of representative-ness	Rural, urban, or both	Age range as used for global analysis		Sample size used for global analysis		Glycaemic markers available	Information available on diabetes treatment	Whether measured in a laboratory or by a point-of-care portable device		Notes
						Female	Male	Female	Male			Glucose	HbA1c	
65	Bosnia and Herzegovina	2012	Non-communicable disease risk factor survey, Federation of B&H	Subnational	urban	18+	18+	716	594	Fasting glucose	Yes	Portable		
66	Botswana	2014	STEPS	National	both	18-69	18-69	2,546	1,252	Fasting glucose	Yes	Portable		
67	Brazil	1991	Fornes et al., Rev Saude Publica 36:12-8, 2002	Community	urban	25+	25+	547	386	Fasting glucose	No	Unknown		
68	Brazil	1992	EPIDOSO; Ramos et al., Rev Saude Publica 32:397-407, 1998	Community	urban	65+	65+	293	171	Fasting glucose	No	Unknown		
69	Brazil	1996-1997	The Bambui Cohort Study of Ageing	Community	urban	18+	18+	1,389	974	Fasting glucose	Yes	Lab		
70	Brazil	1999-2000	Prevalence of Risk Factors for Coronary Artery Disease in the State of Rio Grande do Sul	Subnational	urban	20+	20+	467	430	Fasting glucose	Yes	Lab		
71	Brazil	2003	Marquezine et al., Int J Cardiol 129:259-65, 2004	Community	urban	25-64	25-64	1,787	1,042	Fasting glucose	No	Unknown		
72	Brazil	2004	Caju & Virgen das Gracas	Community	rural	18+	18+	288	291	Fasting glucose	Yes	Lab		
73	Brazil	2002-2004	1978-1979 Ribeira Preto Birth Cohort	Community	urban	22-25	22-25	1,086	1,015	Fasting glucose	Yes	Lab		
74	Brazil	2003-2005	Sao Paulo Health and Ageing Study	Community	urban	65+	65+	1,255	817	Fasting glucose	Yes	Lab		
75	Brazil	2004-2006	Hearts of Brazil	National	urban	18+	18+	655	577	Fasting glucose	Yes	Portable		
76	Brazil	2008	The Bambui Cohort Study of Ageing	Community	urban	71+	71+	518	266	Fasting glucose	Yes	Lab		
77	Brazil	2008	Caju & Virgen das Gracas	Community	rural	18+	18+	289	273	Fasting glucose	Yes	Lab		
78	Brazil	2010	San Pedro	Community	rural	18+	18+	208	146	Fasting glucose	Yes	Lab		
79	Brazil	2011-2012	The 1993 Pelotas (Brazil) Birth Cohort: 18 years follow-up	Community	urban	18-19	18-19	1,963	1,883	HbA1c	Yes		Lab	
80	Brazil	2010-2015	Baependi Heart Study	Community	rural	18+	18+	1,407	1,057	Fasting glucose, HbA1c	Yes	Lab	Lab	
81	Brazil	2012-2013	The 1982 Pelotas (Brazil) Birth Cohort: 30 years follow-up	Community	urban	30	30	1,737	1,684	HbA1c	No		Lab	
82	Brazil	2013	Pesquisas Nacional de Saude	National	both	18+	18+	32,671	25,141	HbA1c	Yes		Unknown	
83	Brazil	2011-2014	Profile of Risk Factors for Coronary Arterial Disease in Rio Grade do Sul - Revaluation After 10 Years	Subnational	urban	20+	20+	465	362	Fasting glucose	Yes	Lab		
84	Brazil	2014-2015	II Diagnóstico de Saúde da População Materno-Infantil do Estado de Alagoas	Subnational	both	19-49		3,146		Fasting glucose	Yes	Portable		
85	Brazil	2014-2015	EpiFloripa Cohort Study of Ageing - Wave 2, Clinical and Laboratory Exams	Community	urban	63+	63+	386	209	HbA1c	No		Lab	
86	Brazil	2014-2015	EpiFloripa Adults Cohort Study	Community	urban	25-65	25-65	410	298	Fasting glucose, HbA1c	No	Lab	Lab	
87	Brazil	2015-2016	The Ouro Preto Study	Community	rural	18+	18+	330	186	Fasting glucose	Yes	Lab		
88	Brunei Darussalam	2010-2011	National Health And Nutritional Status Survey (NHANSS)	National	both	20-75	20-75	807	675	Fasting glucose, HbA1c	Yes	Lab	Lab	
89	Brunei Darussalam	2015-2016	National Non-Communicable Diseases Survey (NNCDS)	National	both	18-69	18-69	2,114	1,677	Fasting glucose	Yes	Lab		
90	Burkina Faso	2013	STEPS	National	both	25-64	25-64	2,289	2,254	Fasting glucose	Yes	Portable		
91	Burkina Faso	2021	STEPS	National	both	18-69	18-69	2,026	1,491	Fasting glucose	Yes	Portable		
92	Cabo Verde	2007	STEPS	National	both	25-64	25-64	1,076	664	Fasting glucose	Yes	Portable		
93	Cabo Verde	2020	STEPS	National	both	18-69	18-69	1,994	1,318	Fasting glucose	Yes	Portable		
94	Cambodia	2010	STEPS	National	both	25-64	25-64	3,410	1,937	Fasting glucose	Yes	Portable		
95	Cambodia	2023	STEPS	National	both	18-69	18-69	2,677	1,513	Fasting glucose	Yes	Portable		2
96	Cameroon	1998-1999	Essential Non-communicable disease Health Intervention Project (ENHIP)	Community	rural	18+	18+	698	485	Fasting glucose	Yes	Portable		
97	Cameroon	1998-1999	Essential Non-communicable disease Health Intervention Project (ENHIP)	Community	urban	18+	18+	538	430	Fasting glucose	Yes	Portable		
98	Cameroon	2000	Defining the relationship between poverty and non-communicable disease burden in Cameroon: Preliminary report; Infobase 101051a1	Subnational	urban	18+	18+	2,028	1,641	Fasting glucose	No	Unknown		
99	Cameroon	2007	Cameroon Burden of Diabetes - Second Survey	Subnational	urban	18+	18+	4,581	3,305	Fasting glucose	Yes	Portable		
100	Cameroon	2014	Prevalence and determinants of chronic kidney disease in rural and urban Cameroonians: A cross-sectional study	Community	both	20+	20+	253	183	Fasting glucose	Yes	Unknown		
101	Cameroon	2014-2015	Cardiovascular risk factors screening in urban and rural areas in the Far-North Region Cameroon	Subnational	both	20+	20+	369	520	Fasting glucose	Yes	Portable		
102	Cameroon	2018	Prevalence and determinants of chronic kidney disease in urban adults' populations of northern Cameroon	Community	urban	20+	20+	220	209	Fasting glucose	No	Unknown		
103	Canada	1993-1995	Kriska et al., Diabetes Care 24:1787-92, 2001	Community	rural	18-35	18-35	180	136	Fasting glucose	No	Lab		
104	Canada	1995-1997	Canadian Multicentre Osteoporosis Study (CaMos) - Adult Baseline	Community	both	25+	25+	6,539	2,884	Fasting glucose	Yes	Lab		
105	Canada	2005-2008	Canadian Multicentre Osteoporosis Study (CaMos) - Adult Year 10 follow-up	Subnational	both	35+	35+	3,993	1,570	Fasting glucose	Yes	Lab		
106	Canada	2007-2009	Canadian Health Measures Survey, Cycle 1	National	both	18-79	18-79	859	789	Fasting glucose, HbA1c	Yes	Lab	Lab	
107	Canada	2009-2011	Canadian Health Measures Survey, Cycle 2	National	both	18-79	18-79	955	825	Fasting glucose, HbA1c	Yes	Lab	Lab	
108	Canada	2012-2013	Canadian Health Measures Survey, Cycle 3	National	both	18-79	18-79	763	798	Fasting glucose, HbA1c	Yes	Lab	Lab	
109	Canada	2014-2015	Canadian Health Measures Survey, Cycle 4	National	both	18-79	18-79	764	736	Fasting glucose, HbA1c	Yes	Lab	Lab	
110	Canada	2016-2017	Canadian Health Measures Survey, Cycle 5	National	both	18-79	18-79	759	787	Fasting glucose, HbA1c	Yes	Lab	Lab	
111	Canada	2018-2019	Canadian Health Measures Survey, Cycle 6	National	both	18-79	18-79	750	809	Fasting glucose, HbA1c	Yes	Lab	Lab	
112	Central African Republic	2010	STEPS	Subnational	both	25-64	25-64	1,998	1,882	Fasting glucose	Yes	Portable		
113	Chile	1988	Chilean Health Study	Subnational	urban	18+	18+	688	415	Fasting glucose	Yes	Lab		
114	Chile	1992-1993	Miquel et al., Gastroenterology 115(4):937-46, 1998	Community	urban	18+	18+	1,032	657	Fasting glucose	No	Lab		
115	Chile	2000	Nervi et al., J Hepatol 45(2):299-305, 2006	Community	urban	18+	18+	625	335	Fasting glucose	No	Lab		
116	Chile	2003	Encuesta Nacional de Salud	National	both	18+	18+	1,951	1,632	Fasting glucose	Yes	Lab		
117	Chile	2004-2005	CArdiovascular Risk factors Multiple Evaluation in Latin America (CARMELA)	Community	urban	25-64	25-64	872	783	Fasting glucose	Yes	Lab		
118	Chile	2009-2010	Encuesta Nacional de Salud	National	both	18+	18+	2,791	1,841	Fasting glucose	Yes	Lab		
119	Chile	2011-2012	CESCAS Study	Community	urban	35-74	35-74	999	916	Fasting glucose	Yes	Lab		
120	Chile	2016-2017	Encuesta Nacional de Salud	National	both	18+	18+	3,796	2,199	Fasting glucose	Yes	Lab		
121	China	1992-1993	Anzhen 02 Cohort Study	Community	urban	34-65	34-65	2,112	2,030	Fasting glucose	No	Unknown		
122	China	1991-1992	Fangshan Cohort Study	Community	urban	34-86	34-86	555	266	Fasting glucose	No	Unknown		
123	China	1995-1996	Hong Kong Cardiovascular Risk Factor Prevalence Study 1995-1996	Community	urban	25-74	25-74	1,483	1,412	Fasting glucose	Yes	Lab		
124	China	1997	DECODA; DECODA Study Group, Diabetes Care 26:1770-80, 2003	Community	urban	30-89	30-89	2,571	1,577	Fasting glucose	No	Unknown		
125	China	2000-2001	The International Collaborative Study of Cardiovascular Disease in Asia	National	both	35-74	35-74	7,828	7,327	Fasting glucose	No	Lab		
126	China	2003	Fan et al., World J Gastroenterol 14:2418-24, 2008	Community	both	25+	25+	7,770	5,529	Fasting glucose	No	Unknown		

	Country	Data years	Survey/Study name/Citation	Level of representative-ness	Rural, urban, or both	Age range as used for global analysis		Sample size used for global analysis		Glycaemic markers available	Information available on diabetes treatment	Whether measured in a laboratory or by a point-of-care portable device		Notes
						Female	Male	Female	Male			Glucose	HbA1c	
127	China	2003	Wu, et al., Prev Med 51:412-5, 2010	Community	both	15+	15+	8,260	6,123	Fasting glucose	No	Unknown		3
128	China	2004	Tian et al., Diabets Res Clin Pract 84:273-8, 2009	Community	rural	35+	35+	405,011	364,781	Fasting glucose	No	Unknown		
129	China	2005	Zhi et al., Chin Med Sci J 23:249-52, 2008	Community	both	18-69	18-69	10,716	9,943	Fasting glucose	No	Unknown		
130	China	2004-2005	Xinjiang Children and Adolescent Survey	Community	urban	18	18	68	55	Fasting glucose	No	Portable		
131	China	2006	Beijing Eye Study	Community	both	45+	45+	1,827	1,393	Fasting glucose	Yes	Lab		
132	China	2006	Qingdao Diabetes Cohort Study	Community	both	35-74	35-74	2,310	1,536	Fasting glucose, HbA1c	Yes	Lab	Lab	
133	China	2006-2007	Fu et al., BMC Public Health 11:862, 2011	Community	rural	18-64	18-64	2,582	1,815	Fasting glucose	No	Lab		
134	China	2006-2007	Handan Eye Study	Community	rural	30+	30+	3,430	2,998	Fasting glucose	Yes	Lab		
135	China	2008-2009	Chinese Longitudinal Healthy Longevity Survey	Subnational	both	65+	65+	753	477	Fasting glucose	No	Lab		4
136	China	2009	China Health and Nutrition Study	National	both	18+	18+	5,163	4,811	Fasting glucose, HbA1c	Yes	Lab	Lab	5
137	China	2009	Qingdao Diabetes Cohort Study	Community	both	37-78	37-78	1,528	979	Fasting glucose, HbA1c	Yes	Lab	Lab	
138	China	2009-2010	China National Survey of Chronic Kidney Disease	National	both	18+	18+	23,341	16,611	Fasting glucose	Yes	Lab		
139	China	2010	China Noncommunicable Disease Surveillance	National	rural	18+	18+	31,297	27,369	Fasting glucose, HbA1c	Yes	Lab	Lab	
140	China	2010	China Noncommunicable Disease Surveillance	National	urban	18+	18+	21,279	16,973	Fasting glucose, HbA1c	Yes	Lab	Lab	
141	China	2011	Beijing Eye Study	Community	both	50+	50+	1,963	1,505	Fasting glucose	Yes	Lab		
142	China	2011-2012	China Health and Retirement Longitudinal Study (CHARLS), baseline survey	National	both	45+	45+	7,083	6,425	Fasting glucose, HbA1c	Yes	Lab	Lab	
143	China	2011-2012	Chinese Longitudinal Healthy Longevity Survey	Subnational	both	65+	65+	1,123	944	Fasting glucose	No	Lab		4
144	China	2010-2013	China National Nutrition and Health Survey	National	both	18+	18+	65,491	50,394	Fasting glucose	No	Lab		
145	China	2014	Chinese Longitudinal Healthy Longevity Survey	Subnational	both	65+	65+	1,219	1,022	Fasting glucose	No	Lab		4
146	China	2015	China Adult Chronic Disease and Nutrition Surveillance	National	both	18+	18+	64,059	56,354	Fasting glucose, HbA1c	No	Lab	Lab	
147	China	2013-2017	Children of 1997 Birth Cohort- Biobank Clinical Follow-up	Community	both	18-20	18-20	250	255	Fasting glucose, HbA1c	No	Lab	Lab	
148	China	2015-2017	Henan Rural Cohort	Subnational	rural	18-79	18-79	23,721	15,488	Fasting glucose	Yes	Lab		
149	China	2015-2016	INTERMAP China Prospective (ICP) Study	Subnational	rural	40-79	40-79	433	348	Fasting glucose	Yes	Unknown		
150	China	2016-2018	The FAMILY Cohort	Community	urban	18+	18+	1,118	842	HbA1c	Yes		Lab	
151	China	2018	Chinese Longitudinal Healthy Longevity Survey	Subnational	both	65+	65+	7,771	6,191	Fasting glucose	Yes	Lab		6
152	Colombia	2001	CINDI/CARMEN-Bucaramaga; Bautista et al., Eur J Cardiovasc Prev Rehabil 13:769-75, 2006	Community	urban	25-64	25-64	1,217	622	Fasting glucose	No	Unknown		
153	Colombia	2004-2005	CArdiovascular Risk factors Multiple Evaluation in Latin America (CARMELA)	Community	urban	25-64	25-64	815	738	Fasting glucose	Yes	Lab		
154	Colombia	2007	Encuesta Nacional de Salud	National	both	18-69	18-69	7,637	5,348	Fasting glucose	Yes	Portable		
155	Colombia	2010	STEPS	Subnational	urban	18-64	18-64	1,239	924	Fasting glucose	Yes	Portable		
156	Colombia	2015	STEPS	Subnational	both	18-64	18-64	1,142	868	Fasting glucose	Yes	Portable		
157	Colombia	2016	The Survey on Health, Well-Being, and Aging in Latin America and the Caribbean (SABE)	National	both	60+	60+	13,582	10,112	Fasting glucose	Yes	Lab		
158	Comoros	2011	STEPS	National	both	25-64	25-64	3,639	1,584	Fasting glucose	Yes	Unknown		
159	Congo	2019	Diabetes prevalence and risk factors	Community	rural	19+	19+	797	709	Fasting glucose	Yes	Portable		
160	Cook Islands	2013-2015	STEPS	National	both	18-64	18-64	630	622	Fasting glucose	Yes	Portable		
161	Cook Islands	2022	STEPS	National	both	18-69	18-69	718	692	Fasting glucose	Yes	Portable		
162	Costa Rica	1988	Campos et al., Circulation 85:648-58, 1992	Community	rural	20-65	20-65	123	111	Fasting glucose	No	Unknown		
163	Costa Rica	1988	Campos et al., Circulation 85:648-58, 1992	Community	urban	20-65	20-65	120	111	Fasting glucose	No	Unknown		
164	Costa Rica	2000	Ministerio de Salud, 2003	Community	urban	25-64	25-64	636	330	Fasting glucose	No	Unknown		
165	Costa Rica	2004	CAMDI	Community	urban	20+	20+	756	390	Fasting glucose	Yes	Lab		
166	Costa Rica	2004-2006	Costa Rican Longevity and Healthy Aging Study Pre-1945 Cohort Wave 1	National	both	60+	60+	1,534	1,293	Fasting glucose, HbA1c	Yes	Lab	Lab	
167	Costa Rica	2006-2008	Costa Rican Longevity and Healthy Aging Study Pre-1945 Cohort Wave 2	National	both	62+	62+	1,286	1,075	Fasting glucose, HbA1c	Yes	Lab	Lab	
168	Costa Rica	2010	Costa Rican National Cardiovascular Risk Factors Survey, 2010	National	both	20+	20+	2,571	992	Fasting glucose	Yes	Lab		
169	Costa Rica	2010-2011	Costa Rican Longevity and Healthy Aging Study 1945-1955 Cohort Wave 1	National	both	54-66	54-66	1,688	1,077	HbA1c	Yes		Lab	
170	Costa Rica	2014	Costa Rican National Cardiovascular Risk Factors Survey, 2014	National	both	20+	20+	2,126	954	Fasting glucose	Yes	Lab		
171	Costa Rica	2018	Costa Rican National Cardiovascular Risk Factors Survey, 2018	National	both	20+	20+	2,291	1,286	Fasting glucose	Yes	Lab		
172	Croatia	2008	Endemic Nephropathy and Arterial hypertension (ENAH)	Subnational	rural	18+	18+	649	502	Fasting glucose	Yes	Lab		
173	Croatia	2010	Endemic Nephropathy and Arterial hypertension (ENAH)	Subnational	rural	18+	18+	401	299	Fasting glucose	Yes	Lab		
174	Croatia	2015	Endemic Nephropathy and Arterial hypertension (ENAH) Follow-up Study	Subnational	rural	18+	18+	464	225	Fasting glucose	Yes	Lab		
175	Croatia	2018-2021	Epidemiology of arterial hypertension in Croatia (EH-UH)	National	both	18+	18+	738	474	Fasting glucose	Yes	Lab		
176	Cuba	2010	National Survey on Risk Factors and Chronic Diseases (NSRFCD)	National	both	18+	18+	4,036	3,558	Fasting glucose	Yes	Lab		
177	Cuba	2010-2011	Noncommunicable disease risk factors in Cienfuegos	Community	urban	18-74	18-74	841	587	Fasting glucose	Yes	Lab		
178	Cuba	2018-2020	Encuesta nacional de salud Cuba 2018-2022 (ENS)	National	both	18+	18+	3,219	2,057	Fasting glucose	Yes	Lab		
179	Czechia	1981	Machova et al., Cas Lek Cesk 143:90-3, 2004; Site 1	Subnational	rural	25+	25+	11,004	9,189	Fasting glucose	No	Unknown		
180	Czechia	1981	Machova et al., Cas Lek Cesk 143:90-3, 2004; Site 2	Subnational	rural	25+	25+	11,004	9,189	Fasting glucose	No	Unknown		
181	Czechia	1997-1998	Czech post-MONICA	National	both	25-64	25-64	1,664	1,529	Fasting glucose	Yes	Lab		
182	Czechia	2000-2001	Czech post-MONICA	National	both	25-64	25-64	1,755	1,686	Fasting glucose	Yes	Lab		
183	Czechia	2002-2005	Health, Alcohol and Psychosocial Factors In Eastern Europe	Subnational	urban	45-70	45-70	4,665	4,060	Fasting glucose, HbA1c	Yes	Lab	Lab	
184	Czechia	2006-2009	Czech post-MONICA	National	both	25-64	25-64	1,840	1,679	Fasting glucose	Yes	Lab		
185	Czechia	2014-2015	European Heath Examination Survey	National	both	25-64	25-64	691	473	HbA1c	Yes		Lab	
186	Czechia	2015-2018	Czech post-MONICA	National	both	25-64	25-64	1,338	1,215	Fasting glucose, HbA1c	Yes	Lab	Lab	
187	Czechia	2019-2020	European Heath Examination Survey	National	both	25-64	25-64	628	426	HbA1c	Yes		Lab	
188	Czechia	2019-2022	CELSPAC: YA (The Central European Longitudinal Studies of Parents and Children: Young Adults)	Community	both	27-30	27-30	151	138	Fasting glucose	No	Lab		
189	Denmark	2007-2008	The Danish Health Examination Survey 2007-2008	National	both	18+	18+	10,334	7,089	HbA1c	Yes		Lab	
190	Denmark	2009-2010	European Youth Heart Study	Community	both	18-28	18-28	333	305	Fasting glucose	Yes	Lab		
191	Denmark	2016	Copenhagen General Population Study	Subnational	urban	20-90	20-90	5,031	4,148	HbA1c	Yes		Lab	
192	Denmark	2017	Copenhagen General Population Study	Subnational	urban	20-90	20-90	4,519	3,282	HbA1c	Yes		Lab	

	Country	Data years	Survey/Study name/Citation	Level of representative-ness	Rural, urban, or both	Age range as used for global analysis		Sample size used for global analysis		Glycaemic markers available	Information available on diabetes treatment	Whether measured in a laboratory or by a point-of-care portable device		Notes
						Female	Male	Female	Male			Glucose	HbA1c	
193	Denmark	2018	Copenhagen General Population Study	Subnational	urban	20-90	20-90	4,018	3,042	HbA1c	Yes		Lab	
194	Denmark	2019	Copenhagen General Population Study	Subnational	urban	20-90	20-90	1,635	1,324	HbA1c	Yes		Lab	
195	Denmark	2020-2021	Copenhagen General Population Study	Subnational	urban	20-90	20-90	1,205	1,066	HbA1c	Yes		Lab	
196	Denmark	2022-2023	Copenhagen General Population Study	Subnational	urban	20-90	20-90	4,047	3,551	HbA1c	Yes		Lab	
197	Dominica	2007-2008	STEPS	National	both	18-64	18-64	531	430	Fasting glucose	Yes	Unknown		
198	Dominican Republic	1996-1998	Estudio factores de riesgo cardiovascular y sindrome metabolico en la Republica Dominicana I (EFRICARD I)	National	both	18-75	18-75	4,097	2,087	Fasting glucose	Yes	Lab		
199	Dominican Republic	2010-2012	Estudio factores de riesgo cardiovascular y sindrome metabolico en la Republica Dominicana II (EFRICARD II)	National	both	18-75	18-75	3,318	1,658	Fasting glucose	Yes	Lab		
200	DR Congo	2007	Diabetes and intermediate hyperglycaemia in Kisantu, DR Congo: a cross-sectional prevalence study	Community	urban	20+	20+	1,197	656	Fasting glucose	Yes	Portable		
201	DR Congo	2016-2017	Prevalence and Risk Factors of CKD in South Kivu, Democratic Republic of Congo: A Large-Scale Population Study	Subnational	both	18+	18+	802	515	Fasting glucose	Yes	Portable		
202	Ecuador	2004-2005	CArdiovascular Risk factors Multiple Evaluation in Latin America (CARMELA)	Community	urban	25-64	25-64	825	813	Fasting glucose	Yes	Lab		
203	Ecuador	2009-2010	The Survey on Health, Well-Being, and Aging in Latin America and the Caribbean (SABE)	National	both	60+	60+	2,706	2,426	Fasting glucose	Yes	Unknown		
204	Ecuador	2011-2013	Encuesta Nacional de Salud y Nutrición (ENSANUT)	National	both	18-59	18-59	8,068	3,690	Fasting glucose	No	Lab		
205	Ecuador	2018	STEPS	National	both	18-69	18-69	2,632	1,944	Fasting glucose	Yes	Portable		
206	Egypt	1995	Herman et al., Diabet Med 12:1126-31, 1995	Community	urban	20-79	20-79	425	604	Fasting glucose	No	Unknown		
207	Egypt	2003-2004	Marzouk et al., Gut 56(8):1105-10, 2007	Community	rural	25+	25+	455	321	Fasting glucose	Yes	Lab		
208	Egypt	2005	STEPS	National	both	18-65	18-65	3,923	4,164	Fasting glucose	Yes	Lab		
209	Egypt	2007-2009	Mostafa et al., Gut 59(8):1135-40, 2010	Community	rural	35+	35+	846	642	Fasting glucose	Yes	Lab		
210	Egypt	2011	STEPS	National	both	18-65	18-65	2,959	1,740	Fasting glucose	Yes	Unknown		
211	Egypt	2017	STEPS	National	both	18-69	18-69	3,669	2,094	Fasting glucose	Yes	Portable		
212	El Salvador	2004	CAMDI	Community	urban	20+	20+	822	405	Fasting glucose	Yes	Lab		
213	El Salvador	2014-2015	Encuesta Nacional de Enfermedades Crónicas (ENECA-ELS)	National	both	20+	20+	2,964	1,703	Fasting glucose	Yes	Lab		
214	Eritrea	2010	STEPS	National	both	25-74	25-74	4,309	1,725	Fasting glucose	Yes	Portable		
215	Estonia	1997	SWESTONIA; Johansson et al., J Intern Med 252:551-60, 2002	Community	urban	35-55	35-55	133	144	Fasting glucose	No	Unknown		
216	Estonia	2003	The European Male Ageing Study	Community	both		40+		428	Fasting glucose	Yes	Lab		
217	Estonia	2008	The European Male Ageing Study	Community	both		45+		327	Fasting glucose	Yes	Lab		
218	Eswatini	2014	STEPS	National	both	18-69	18-69	1,925	1,016	Fasting glucose	Yes	Portable		
219	Ethiopia	2015	STEPS	National	both	18-69	18-69	5,127	3,752	Fasting glucose	Yes	Portable		
220	Fiji	1980	National Cardiovascular and Diabetes Survey (NCVDS)	Subnational	both	20+	20+	1,523	1,449	Fasting glucose	Yes	Lab		
221	Fiji	2002	STEPS	National	both	25-64	25-64	2,985	2,055	Fasting glucose	Yes	Portable		
222	Fiji	2009	Fiji Eye Health Survey 2009	National	both	40+	40+	787	590	HbA1c	Yes		Portable	
223	Fiji	2011	STEPS	National	both	25-64	25-64	1,394	1,096	Fasting glucose	Yes	Portable		
224	Finland	1984	Tuomilehto et al., Diabetologia 29:611-5, 1986; Site 1	Subnational	both		65-84		296	Fasting glucose	No	Unknown		
225	Finland	1984	Tuomilehto et al., Diabetologia 29:611-5, 1986; Site 2	Subnational	both		65-84		367	Fasting glucose	No	Unknown		
226	Finland	1984	Finland, Italy, Netherlands, Elderly (Fine-Finland)	Community	rural		65-84		715	Fasting glucose	Yes	Lab		
227	Finland	1986	Young Finns Study 1986	National	rural	18-24	18-24	230	200	Fasting glucose	No	Lab		
228	Finland	1986	Young Finns Study 1986	National	urban	18-24	18-24	326	253	Fasting glucose	No	Lab		
229	Finland	1984-1989	Kuopio Ischaemic Heart Disease Risk Factor Study	Subnational	both		42-61		2,682	Fasting glucose	Yes	Lab		
230	Finland	1989	Finland, Italy, Netherlands, Elderly (Fine-Finland)	Community	rural		70-89		450	Fasting glucose	No	Lab		
231	Finland	1990-1992	Oulu 35 Study	Community	urban	56-57	56-57	327	231	Fasting glucose	Yes	Lab		
232	Finland	1991-1993	Kuopio Ischaemic Heart Disease Risk Factor Study	Subnational	both		46-65		1,038	Fasting glucose	Yes	Lab		
233	Finland	1997	Northern Finland Birth Cohort 1966	Community	both	30-31	30-31	256	2,631	Fasting glucose	Yes	Lab		
234	Finland	1996-1998	Oulu 35 Study	Community	urban	60-63	60-63	346	244	Fasting glucose	Yes	Portable + Lab		
235	Finland	1998-2001	Kuopio Ischaemic Heart Disease Risk Factor Study	Subnational	both	53-73	53-73	919	834	Fasting glucose	Yes	Lab		
236	Finland	2000-2001	Health 2000 Survey	National	both	30+	30+	3,889	3,159	Fasting glucose, HbA1c	Yes	Lab	Lab	
237	Finland	2001	Young Finns Study 2001	National	rural	24-39	24-39	395	344	Fasting glucose	No	Lab		
238	Finland	2001	Young Finns Study 2001	National	urban	24-39	24-39	770	660	Fasting glucose	No	Lab		
239	Finland	2001-2003	Oulu 45 Study	Community	urban	55-58	55-58	550	428	Fasting glucose	Yes	Lab		
240	Finland	2001-2004	Helsinki Birth Cohort Study	Community	urban	56-69	56-69	1,075	928	Fasting glucose	Yes	Lab		
241	Finland	2005	Mantyselka et al., Rheumatology (Oxford) 47:1235-8, 2008	Community	rural	30-65	30-65	250	229	Fasting glucose	No	Unknown		
242	Finland	2005-2008	Kuopio Ischaemic Heart Disease Risk Factor Study	Subnational	both	60-81	60-81	634	1,241	Fasting glucose	Yes	Lab		
243	Finland	2007	Oulu 35 Study	Community	urban	71-73	71-73	272	184	Fasting glucose	Yes	Lab		
244	Finland	2007	Young Finns Study 2007	National	rural	30-45	30-45	448	384	Fasting glucose	Yes	Lab		
245	Finland	2007	Young Finns Study 2007	National	urban	30-45	30-45	728	603	Fasting glucose	Yes	Lab		
246	Finland	2008	Control group for Finnish male former elite athletes	National	both		61+		207	Fasting glucose	Yes	Lab		
247	Finland	2007-2008	Savitaipale Study, 10-year Follow-up	Community	rural	51-75	51-75	358	259	Fasting glucose	Yes	Portable		
248	Finland	2011	Young Finns Study 2011	National	rural	34-49	34-49	436	368	Fasting glucose, HbA1c	Yes	Lab	Lab	
249	Finland	2011	Young Finns Study 2011	National	urban	34-49	34-49	650	513	Fasting glucose, HbA1c	Yes	Lab	Lab	
250	Finland	2011-2012	Health 2011 Survey	National	both	30+	30+	2,819	2,288	Fasting glucose, HbA1c	Yes	Lab	Lab	
251	Finland	2012	Northern Finland Birth Cohort 1966	Community	both	45-47	45-47	2,843	2,206	Fasting glucose, HbA1c	Yes	Lab	Lab	
252	Finland	2017	The FinHealth Survey	National	both	18+	18+	3,627	3,209	Fasting glucose, HbA1c	Yes	Lab	Lab	
253	Finland	2018-2019	Savitaipale Study, 22-year Follow-up	Community	rural	62-86	62-86	320	243	Fasting glucose, HbA1c	Yes	Lab	Lab	
254	Finland	2018-2020	Young Finns Study: Follow-up	National	both	18+	18+	2,953	2,138	Fasting glucose, HbA1c	Yes	Lab	Lab	
255	Finland	2019-2020	Northern Finland Birth Cohort 1986	Community	both	33-35	33-35	918	581	Fasting glucose, HbA1c	Yes	Lab	Lab	
256	France	1996	POLA Study; Defay et al., Int J Obes Relat Metab Disord 25:512-8, 2001	Community	both	60+	60+	1,419	1,113	Fasting glucose	No	Unknown		
257	France	1996	Asmar et al., J Hypertens 19:1727-32, 2001	Subnational	both	18+	18+	31,416	29,692	Fasting glucose	No	Unknown		

	Country	Data years	Survey/Study name/Citation	Level of representative-ness	Rural, urban, or both	Age range as used for global analysis		Sample size used for global analysis		Glycaemic markers available	Information available on diabetes treatment	Whether measured in a laboratory or by a point-of-care portable device		Notes
						Female	Male	Female	Male			Glucose	HbA1c	
258	France	1999-2001	The Three City Study	Community	urban	65+	65+	5,644	3,650	Fasting glucose	Yes	Lab		
259	France	2003-2005	The Three City Study	Community	urban	68+	68+	4,435	2,712	Fasting glucose	Yes	Lab		
260	France	2006-2007	Etude Nationale Nutrition Santé	National	both	18-74	18-74	1,379	824	Fasting glucose	Yes	Lab		7
261	France	2008-2010	The Three City Study	Community	urban	73+	73+	3,024	1,666	Fasting glucose, HbA1c	Yes	Lab	Lab	
262	France	2011-2013	Enquête LIttorale Souffle Air Biologie EnvironnemenT (ELISABET) Dunkerque	Community	urban	40-64	40-64	779	751	Fasting glucose, HbA1c	Yes	Lab	Lab	
263	France	2011-2013	Enquête LIttorale Souffle Air Biologie EnvironnemenT (ELISABET) Lille	Community	urban	40-64	40-64	838	754	Fasting glucose, HbA1c	Yes	Lab	Lab	
264	France	2012-2014	Cohorte des consultants des Centres d'examens de santé (CONSTANCES)	National	urban	18-69	18-69	25,538	22,367	Fasting glucose	Yes	Lab		
265	France	2014-2016	L'Etude de Sante sur l'Environnement, la Biosurveillance, l'Activite physique et la Nutrition (Etude Esteban)	National	both	18-74	18-74	1,268	992	Fasting glucose	Yes	Lab		8
266	France	2015-2017	Cohorte des consultants des Centres d'examens de santé (CONSTANCES)	National	urban	18-69	18-69	50,369	44,390	Fasting glucose	Yes	Lab		
267	France	2017-2019	Cohorte des consultants des Centres d'examens de santé (CONSTANCES)	Subnational	urban	22-76	22-76	8,552	7,703	Fasting glucose	Yes	Lab		
268	France	2018-2019	Cohorte des consultants des Centres d'examens de santé (CONSTANCES)	National	urban	18-69	18-69	26,699	23,288	Fasting glucose	Yes	Lab		
269	France	2020-2021	Cohorte des consultants des Centres d'examens de santé (CONSTANCES)	Subnational	urban	18-69	18-69	1,635	1,344	Fasting glucose	Yes	Lab		
270	France	2020-2022	Cohorte des consultants des Centres d'examens de santé (CONSTANCES)	Subnational	urban	22-80	22-80	19,004	17,393	Fasting glucose	Yes	Lab		
271	French Polynesia	2010	STEPS	National	both	18-64	18-64	1,267	950	Fasting glucose	No	Portable		
272	Gambia	2018	The Gambia Micronutrient Survey (GMNS)	National	both	18-49		1,192		HbA1c	No		Portable	
273	Georgia	2010	STEPS	National	both	18-64	18-64	4,499	1,870	Fasting glucose	Yes	Portable		
274	Georgia	2016	STEPS	National	both	18-69	18-69	2,887	1,271	Fasting glucose	Yes	Portable		
275	Germany	2000-2002	Epidemiological study of the chances of prevention, early recognition and optimal treatment of chronic diseases in an elderly population (ESTHER)	Subnational	both	50-75	50-75	5,418	4,436	Fasting glucose, HbA1c	Yes	Lab	Lab	
276	Germany	2002	Echinoccoccus Multilocularis and Internal Diseases in Leutkirch	Community	urban	18-65	18-65	964	875	HbA1c	Yes		Lab	
277	Germany	2000-2003	Heinz Nixdorf Recall Study	Subnational	urban	45-75	45-75	2,273	2,223	Fasting glucose, HbA1c	Yes	Lab	Lab	9
278	Germany	2002-2006	Study of Health in Pomerania (SHIP-START-1) 5-year follow-up	Subnational	both	25-85	25-85	1,674	1,568	HbA1c	No		Lab	10
279	Germany	2005-2008	Heinz Nixdorf Recall Study	Subnational	both	50-80	50-80	2,103	2,054	Fasting glucose, HbA1c	Yes	Lab	Lab	9
280	Germany	2008-2011	Epidemiological study of the chances of prevention, early recognition and optimal treatment of chronic diseases in an elderly population (ESTHER)	Subnational	both	58-84	58-84	3,267	2,655	HbA1c	Yes		Lab	
281	Germany	2008-2011	German Health Interview and Examination Survey for Adults 2008-11 (DEGS1)	National	both	18-79	18-79	3,549	3,280	HbA1c	Yes		Lab	
282	Germany	2008-2012	Study of Health in Pomerania (SHIP-START-2) 11-year follow-up	Subnational	both	31-81	31-81	1,198	1,052	HbA1c	No		Lab	10
283	Germany	2008-2012	Study of Health in Pomerania, second cohort (SHIP-TREND-0)	Subnational	both	20-79	20-79	2,229	2,096	Fasting glucose, HbA1c	Yes	Lab	Lab	10
284	Germany	2011-2014	Heinz Nixdorf Recall Study	Subnational	both	56-85	56-85	1,573	1,504	Fasting glucose, HbA1c	Yes	Lab	Lab	9
285	Germany	2014-2016	Study of Health in Pomerania (SHIP-START-3) 16-year follow-up	Subnational	both	37-87	37-87	908	776	HbA1c	Yes		Lab	10
286	Germany	2016-2019	Study of Health in Pomerania, second cohort (SHIP-TREND-1) 8-year follow-up	Subnational	both	28-90	28-90	1,276	1,202	Fasting glucose, HbA1c	Yes	Lab	Lab	10
287	Ghana	2003	Women's Health Study of Accra (WHSA-I)	Community	urban	18+		3,004		Fasting glucose	Yes	Lab		
288	Ghana	2006	STEPS	Community	urban	25+	25+	1,706	887	Fasting glucose	Yes	Portable		
289	Ghana	2012-2014	Research on Obesity and Diabetes among African Migrants (RODAM), control group	Subnational	rural	25+	25+	679	432	Fasting glucose, HbA1c	Yes	Lab	Lab	
290	Ghana	2012-2014	Research on Obesity and Diabetes among African Migrants (RODAM), control group	Subnational	urban	25+	25+	1,033	419	Fasting glucose, HbA1c	Yes	Lab	Lab	
291	Ghana	2023	STEPS	National	both	18-69	18-69	3,251	2,022	Fasting glucose	Yes	Portable		2
292	Greece	2001	Karalis et al., BMC Public Health 25:1330-6, 2007	Community	rural	25+	25+	103	91	Fasting glucose	No	Unknown		
293	Greece	2001-2002	The ATTICA study	Community	urban	18+	18+	1,525	1,505	Fasting glucose	Yes	Lab		
294	Greece	2006	Paliouri Study	Community	rural	65-94	65-94	71	95	Fasting glucose	Yes	Lab		
295	Greece	2013-2015	Hellenic National Nutrition and Health Survey (HNNHS)	Subnational	urban	18+	18+	2,265	1,559	Fasting glucose	Yes	Lab		
296	Greece	2013-2016	National Survey of Morbidity and Risk Factors (EMENO)	National	both	18+	18+	3,400	2,519	Fasting glucose, HbA1c	Yes	Lab	Lab	
297	Greenland	2005-2010	Population Health Survey in Greenland	National	both	18+	18+	1,727	1,356	Fasting glucose, HbA1c	Yes	Lab	Lab	
298	Greenland	2016-2019	Population Health Survey in Greenland	National	both	18+	18+	1,053	841	Fasting glucose, HbA1c	Yes	Lab	Lab	
299	Guatemala	2001-2002	CAMDI	Community	urban	20+	20+	683	349	Fasting glucose	No	Lab		
300	Guatemala	2003-2005	The Institute of Nutrition of Central America and Panama Nutrition Supplementation Trial Cohort	Community	both	25-41	25-41	293	242	Fasting glucose	Yes	Portable		
301	Guatemala	2015	STEPS	Subnational	urban	18+	18+	1,551	458	Fasting glucose	Yes	Portable		
302	Guatemala	2015-2017	Nutrition on early childhood and metabolomic and cardiometabolic profile on adulthood (META)	Community	both	37-55	37-55	302	207	Fasting glucose, HbA1c	No	Lab	Lab	
303	Guatemala	2016	Sistema de vigilancia Epidemiológica de Salud y Nutrición (SIVESNU)	National	both	18-49		1,341		HbA1c	No		Portable	
304	Guatemala	2017-2018	Sistema de vigilancia Epidemiológica de Salud y Nutrición (SIVESNU)	National	both	18-49		1,344		HbA1c	No		Lab	
305	Guatemala	2018-2019	Population-Based Survey of Chronic Kidney Disease in Guatemala	Community	rural	18+	18+	508	263	HbA1c	No		Lab	
306	Guatemala	2018-2019	Sistema de vigilancia Epidemiológica de Salud y Nutrición (SIVESNU)	National	both	18-49		1,489		HbA1c	Yes		Lab	
307	Guinea	2009	STEPS	Subnational	both	18-64	18-64	1,131	1,056	Fasting glucose	Yes	Portable		
308	Guyana	2016	STEPS	National	both	18-69	18-69	1,585	1,068	Fasting glucose, HbA1c	Yes	Portable	Lab	
309	Haiti	2015-2016	Haiti Health Study (Carrefour)	Community	urban	25-65	25-65	685	474	HbA1c	No		Portable	
310	Haiti	2015-2016	Haiti Health Study (Thomonde)	Community	rural	25-65	25-65	423	258	HbA1c	Yes		Portable	
311	Honduras	2003-2004	CAMDI	Community	urban	20+	20+	786	435	Fasting glucose	Yes	Lab		
312	Hungary	1990-1998	Simay et al., Public Health 119:437-41, 2005	Community	both	18+	18+	13,647	10,651	Fasting glucose	No	Lab		
313	Hungary	2003	The European Male Ageing Study	Community	both		40+		421	Fasting glucose	Yes	Lab		
314	Hungary	2008	The European Male Ageing Study	Community	both		45+		343	Fasting glucose	Yes	Lab		
315	Iceland	2002-2006	AGES	Subnational	urban	66-96	66-96	3,324	2,437	Fasting glucose	Yes	Lab		
316	Iceland	2007-2011	AGESII	Subnational	urban	71-98	71-98	1,934	1,382	Fasting glucose	Yes	Lab		
317	India	1988-1989	Ramachandran et al., Diabetes Res Clin Pract 58(1):55-60, 2002	Community	urban	20-74	20-74	408	438	Fasting glucose	No	Portable		
318	India	1995	Shobana et al., Diabetes Res Clin Pract 42(3):18186, 1998	Community	urban	20-74	20-74	710	741	Fasting glucose	No	Portable		
319	India	1996-1999	Chennai Urban Population Study	Community	urban	20+	20+	698	542	Fasting glucose	Yes	Lab		
320	India	1999	DECODA; DECODA Study Group, Diabetes Care 26:1770-80, 2003	Community	urban	30-79	30-79	1,322	1,297	Fasting glucose	No	Unknown		
321	India	2000	Ramachandran et al., Diabet Med 20(3):220-24, 2003	Subnational	urban	20-75	20-75	5,267	4,644	Fasting glucose	No	Portable		
322	India	1999-2001	Jaipur Heart Watch 2	Community	urban	20-75	20-75	562	485	Fasting glucose	No	Lab		

	Country	Data years	Survey/Study name/Citation	Level of representative-ness	Rural, urban, or both	Age range as used for global analysis		Sample size used for global analysis		Glycaemic markers available	Information available on diabetes treatment	Whether measured in a laboratory or by a point-of-care portable device		Notes
						Female	Male	Female	Male			Glucose	HbA1c	
323	India	1998-2002	Vellore Birth Cohort	Subnational	both	25-31	25-31	1,055	1,163	Fasting glucose	Yes	Lab		
324	India	1999-2002	New Delhi Birth Cohort	Community	urban	26-33	26-33	636	881	Fasting glucose	Yes	Lab		
325	India	2003	Study in Chennai	Community	rural	20-79	20-79	575	346	Fasting glucose	No	Unknown		
326	India	2001-2004	Chennai Urban Rural Epidemiology Study	Community	urban	20+	20+	1,254	1,096	Fasting glucose, HbA1c	Yes	Lab	Lab	
327	India	2003-2004	Jaipur Heart Watch 3	Community	urban	20-75	20-75	228	212	Fasting glucose	Yes	Lab		
328	India	2006	Ramachandran et al., Diabetes Care 31(5):893-98, 2008	Community	both	20+	20+	3,745	3,321	Fasting glucose	Yes	Lab		
329	India	2005-2006	Risk factor profile for chronic non-communicable diseases: Results of a community-based study in Kerala, India	Community	both	18-64	18-64	2,810	2,615	Fasting glucose	Yes	Unknown		
330	India	2005-2006	National Nutrition Monitoring Bureau rural survey	Subnational	rural	20+	20+	13,671	11,901	Fasting glucose	Yes	Portable		
331	India	2006-2008	Central India Eye and Medical Study	Community	rural	30+	30+	2,518	2,191	HbA1c	Yes		Lab	
332	India	2006-2007	Jaipur Heart Watch 4	Community	urban	20-75	20-75	536	502	Fasting glucose	Yes	Lab		
333	India	2006-2008	Kashmiri Young Adults	Subnational	both	20-40	20-40	912	2,120	Fasting glucose	No	Portable		
334	India	2007-2008	Urban population in Hyderabad	Community	urban	20-60	20-60	1,552	1,511	Fasting glucose, HbA1c	Yes	Portable	Portable	
335	India	2006-2009	New Delhi Birth Cohort	Community	urban	33-38	33-38	448	652	Fasting glucose	Yes	Lab		
336	India	2008-2010	ICMR-India Diabetes (INDIAB) Study, Phase I	National	both	20+	20+	6,944	7,110	Fasting glucose, HbA1c	Yes	Portable	Lab	
337	India	2009-2010	Jaipur Heart Watch 5	Community	urban	20-75	20-75	274	429	Fasting glucose	Yes	Lab		
338	India	2010-2012	Centre for cArdiometabolic Risk Reduction in South-Asia (CARRS) - Surveillance Study	Community	urban	20+	20+	6,402	5,867	Fasting glucose, HbA1c	Yes	Lab	Lab	11
339	India	2011-2012	National Nutrition Monitoring Bureau rural survey	National	rural	18+	18+	27,080	21,937	Fasting glucose	Yes	Lab		
340	India	2012-2013	ICMR-India Diabetes (INDIAB) Study, Phase II	Subnational	both	20+	20+	10,866	8,252	Fasting glucose, HbA1c	Yes	Portable	Lab	
341	India	2012-2014	Jaipur Heart Watch 6	Community	urban	20-75	20-75	353	516	Fasting glucose	No	Lab		
342	India	2012-2013	Processed and non-processed foods - Rural sample	National	rural	18+	18+	2,093	1,855	Fasting glucose	No	Portable		
343	India	2014	Annual Health Survey: Clinical, Anthropometric and Bio-chemical	National	both	18+	18+	449,906	407,330	Fasting glucose	No	Lab		
344	India	2012-2015	ICMR-India Diabetes (INDIAB) Study, North East Phase	Subnational	both	20+	20+	16,682	14,260	Fasting glucose, HbA1c	Yes	Portable	Lab	
345	India	2013-2014	Vellore Birth Cohort	Subnational	both	39-44	39-44	499	581	Fasting glucose, HbA1c	Yes	Lab	Lab	
346	India	2014-2015	Control of Hypertension In Rural India (CHIRI) - Rishi Valley	Community	rural	18+	18+	3,551	2,616	Fasting glucose, HbA1c	Yes	Portable	Portable	
347	India	2015-2016	Diet and nutritional status of urban population and prevalence of hypertension	National	urban	18+	18+	53,527	39,397	Fasting glucose	Yes	Portable		
348	India	2017-2018	National Noncommunicable Disease Monitoring Survey (NNMS)	National	both	18-69	18-69	4,666	5,011	Fasting glucose	Yes	Portable		
349	India	2016-2017	Secular TRends in DiabEtes in India (STRiDE-I) -Change in Prevalence in Ten Years among Urban and Rural Populations in Tamil Nadu	Community	both	20+	20+	5,319	4,527	Fasting glucose	Yes	Portable		
350	India	2017-2018	ICMR-India Diabetes (INDIAB) Study, Phase III	Subnational	both	20+	20+	7,693	7,282	Fasting glucose, HbA1c	Yes	Portable	Lab	
351	India	2016-2019	Vellore Birth Cohort	Subnational	both	43-48	43-48	758	843	Fasting glucose, HbA1c	Yes	Lab	Lab	
352	India	2018-2019	ICMR-India Diabetes (INDIAB) Study, Phase IV	Subnational	both	20+	20+	10,320	9,026	Fasting glucose, HbA1c	Yes	Portable	Lab	
353	India	2019-2020	ICMR-India Diabetes (INDIAB) Study, Phase V	Subnational	both	20+	20+	6,701	6,670	Fasting glucose, HbA1c	Yes	Portable	Lab	
354	India	2021	STEPS, Mumbai	Community	urban	18-69	18-69	2,575	2,601	Fasting glucose	Yes	Portable		
355	Indonesia	2001	STEPS/SURKESNAS	Subnational	both	25+	25+	2,186	1,895	Fasting glucose	No	Unknown		
356	Indonesia	2003	A genetic-ecological study of the risk factors for lifestyle-related diseases in Oceanian populations, Study A	Community	rural	18-79	18-79	103	100	Fasting glucose	Yes	Lab		
357	Indonesia	2003	A genetic-ecological study of the risk factors for lifestyle-related diseases in Oceanian populations, Study B	Community	rural	18-79	18-79	140	100	Fasting glucose	Yes	Lab		
358	Indonesia	2006	NCD RFS; Soebardi et al., Acta Med Indones 41:186-90, 2009	Community	urban	25-64	25-64	950	641	Fasting glucose	No	Unknown		
359	Indonesia	2018	Indonesian Basic Health Survey (RISKESDAS) 2018	National	both	18+	18+	20,614	14,493	Fasting glucose	Yes	Portable		12
360	Iran	1994	Sarraf-Zadegan et al., Acta Cardiol 54:257-63, 1999	Community	urban	20-69	20-69	1,069	1,000	Fasting glucose	No	Unknown		
361	Iran	1999-2000	National Health Survey II	Subnational	both	18+	18+	598	498	Fasting glucose	No	Lab		
362	Iran	2001	Report on the pilot project for community-based primary prevention of the major noncommunicable diseases in Qazvin & Abhar cities 2001; 1; Infobase 101013a2	Community	urban	25+	25+	496	471	Fasting glucose	No	Unknown		
363	Iran	2001	Report on the pilot project for community-based primary prevention of the major noncommunicable diseases in Qazvin & Abhar cities 2001; Infobase 101013a1	Community	urban	25+	25+	495	489	Fasting glucose	No	Unknown		
364	Iran	2001	Isfahan Healthy Heart Programme (IHHP), Arak	Community	rural	19+	19+	1,091	1,028	Fasting glucose	Yes	Lab		
365	Iran	2001	Isfahan Healthy Heart Programme (IHHP), Arak	Community	urban	19+	19+	2,131	2,089	Fasting glucose	Yes	Lab		
366	Iran	2001	Isfahan Healthy Heart Programme (IHHP), Isfahan	Community	rural	19+	19+	238	234	Fasting glucose	Yes	Lab		
367	Iran	2001	Isfahan Healthy Heart Programme (IHHP), Isfahan	Community	urban	19+	19+	1,932	1,782	Fasting glucose	Yes	Lab		
368	Iran	2001	Isfahan Healthy Heart Programme (IHHP), Najaf Abad	Community	rural	19+	19+	419	409	Fasting glucose	Yes	Lab		
369	Iran	2001	Isfahan Healthy Heart Programme (IHHP), Najaf Abad	Community	urban	19+	19+	578	581	Fasting glucose	Yes	Lab		
370	Iran	2004	Azimi-Nezhad et al., Singapore Med J 49:571-6, 2008	Community	both	15+	15+	1,675	1,585	Fasting glucose	No	Lab		3
371	Iran	2003-2004	Childhood and Adolescence Surveillance and Prevention of Adult Noncommunicable Disease (CASPIAN)	National	both	18	18	368	373	Fasting glucose	Yes	Lab		
372	Iran	2003-2004	The Persian Gulf Healthy Heart Study	Subnational	urban	25-75	25-75	1,974	1,741	Fasting glucose	Yes	Lab		
373	Iran	2005	STEPS	National	both	25-64	25-64	31,782	32,723	Fasting glucose	Yes	Lab		
374	Iran	2007	Isfahan Healthy Heart Programme (IHHP), Arak	Community	rural	19+	19+	1,028	1,030	Fasting glucose	Yes	Lab		
375	Iran	2007	Isfahan Healthy Heart Programme (IHHP), Arak	Community	urban	19+	19+	1,366	1,429	Fasting glucose	Yes	Lab		
376	Iran	2007	Isfahan Healthy Heart Programme (IHHP), Isfahan	Community	rural	19+	19+	153	158	Fasting glucose	Yes	Lab		
377	Iran	2007	Isfahan Healthy Heart Programme (IHHP), Isfahan	Community	urban	19+	19+	1,435	1,415	Fasting glucose	Yes	Lab		
378	Iran	2007	Isfahan Healthy Heart Programme (IHHP), Najaf Abad	Community	rural	19+	19+	254	254	Fasting glucose	Yes	Lab		
379	Iran	2007	Isfahan Healthy Heart Programme (IHHP), Najaf Abad	Community	urban	19+	19+	544	498	Fasting glucose	Yes	Lab		
380	Iran	2007	Isfahan Healthy Heart Programme (IHHP) Students, Arak	Community	rural	18	18	4	8	Fasting glucose	No	Lab		
381	Iran	2007	Isfahan Healthy Heart Programme (IHHP) Students, Arak	Community	urban	18	18	4	17	Fasting glucose	No	Lab		
382	Iran	2007	Isfahan Healthy Heart Programme (IHHP) Students, Isfahan	Community	urban	18	18	6	9	Fasting glucose	No	Lab		
383	Iran	2007	Isfahan Healthy Heart Programme (IHHP) Students, Najaf Abad	Community	rural		18		3	Fasting glucose	No	Lab		
384	Iran	2007	STEPS - National	National	both	25-64	25-64	1,886	1,912	Fasting glucose	Yes	Lab		
385	Iran	2007	STEPS - Provincial	National	both	25-64	25-64	11,705	11,919	Fasting glucose	Yes	Lab		
386	Iran	2009-2010	Childhood and Adolescence Surveillance and Prevention of Adult Noncommunicable Disease (CASPIAN)	National	both	18	18	532	497	Fasting glucose	Yes	Lab		

	Country	Data years	Survey/Study name/Citation	Level of representative-ness	Rural, urban, or both	Age range as used for global analysis		Sample size used for global analysis		Glycaemic markers available	Information available on diabetes treatment	Whether measured in a laboratory or by a point-of-care portable device		Notes
						Female	Male	Female	Male			Glucose	HbA1c	
387	Iran	2009-2010	The Persian Gulf Healthy Heart Study	Subnational	urban	31-79	31-79	1,014	833	Fasting glucose	Yes	Lab		
388	Iran	2008-2011	Tehran Lipid and Glucose Study	Community	urban	20+	20+	6,000	4,704	Fasting glucose	Yes	Lab		
389	Iran	2010-2012	Golestan Cohort Study Second Phase	Subnational	rural	43-82	43-82	4,921	4,325	Fasting glucose	Yes	Lab		
390	Iran	2010-2012	Golestan Cohort Study Second Phase	Community	urban	43-82	43-82	1,061	1,091	Fasting glucose	Yes	Lab		
391	Iran	2011	STEPS	National	both	25-69	25-69	4,854	3,313	Fasting glucose	Yes	Lab		
392	Iran	2011-2012	Amol county study	Community	rural	18+	18+	1,024	1,685	Fasting glucose	Yes	Lab		
393	Iran	2011-2012	Amol county study	Community	urban	18+	18+	1,476	1,522	Fasting glucose	Yes	Lab		
394	Iran	2012-2013	Tehran City	Community	urban	18-90	18-90	519	395	Fasting glucose	Yes	Lab		
395	Iran	2012-2014	Pars Cohort Study	Community	rural	40-90	40-90	4,988	4,276	Fasting glucose	Yes	Lab		
396	Iran	2012-2013	Zahedan City	Community	urban	18-90	18-90	1,072	1,133	Fasting glucose	Yes	Lab		
397	Iran	2013-2014	Bushehr Elderly Health Program (BEH)	Community	urban	60+	60+	1,545	1,455	Fasting glucose	Yes	Lab		
398	Iran	2013-2014	Gilan Eye Study	Subnational	both	50+	50+	1,159	839	Fasting glucose, HbA1c	No	Lab	Lab	
399	Iran	2014-2015	Childhood and Adolescence Surveillance and Prevention of Adult Noncommunicable Disease (CASPIAN)	National	both	18	18	388	330	Fasting glucose	Yes	Lab		
400	Iran	2014-2016	The PERSIAN Fasa Cohort Study	Community	rural	35-70	35-70	4,584	3,752	Fasting glucose	Yes	Lab		
401	Iran	2014-2016	The PERSIAN Fasa Cohort Study	Community	urban	35-70	35-70	812	722	Fasting glucose	Yes	Lab		
402	Iran	2014-2016	The PERSIAN Guilan Cohort Study	Community	rural	35-70	35-70	3,261	2,646	Fasting glucose	Yes	Lab		
403	Iran	2014-2016	The PERSIAN Guilan Cohort Study	Community	urban	35-70	35-70	2,350	2,236	Fasting glucose	Yes	Lab		
404	Iran	2014-2016	The PERSIAN Kermanshah Cohort Study	Community	rural	35-70	35-70	2,198	1,809	Fasting glucose	Yes	Lab		
405	Iran	2014-2016	The PERSIAN Kermanshah Cohort Study	Community	urban	35-70	35-70	2,976	2,948	Fasting glucose	Yes	Lab		
406	Iran	2014-2016	The PERSIAN Kharameh Cohort Study	Community	rural	35-70	35-70	3,861	2,914	Fasting glucose	Yes	Lab		
407	Iran	2014-2016	The PERSIAN Kharameh Cohort Study	Community	urban	35-70	35-70	2,001	1,795	Fasting glucose	Yes	Lab		
408	Iran	2014-2016	The PERSIAN Tabriz Cohort Study	Community	rural	35-70	35-70	2,549	1,974	Fasting glucose	Yes	Lab		
409	Iran	2014-2016	The PERSIAN Tabriz Cohort Study	Community	urban	35-70	35-70	5,595	4,671	Fasting glucose	Yes	Lab		
410	Iran	2015-2017	The PERSIAN Mazandaran Cohort Study	Community	rural	35-70	35-70	1,608	936	Fasting glucose	Yes	Lab		
411	Iran	2015-2017	The PERSIAN Mazandaran Cohort Study	Community	urban	35-70	35-70	4,421	3,179	Fasting glucose	Yes	Lab		
412	Iran	2015-2017	The PERSIAN Rafsanjan Cohort Study	Community	rural	35-70	35-70	1,030	1,578	Fasting glucose	Yes	Lab		
413	Iran	2015-2017	The PERSIAN Rafsanjan Cohort Study	Community	urban	35-70	35-70	4,280	3,593	Fasting glucose	Yes	Lab		
414	Iran	2016	Iran STEPS 2016	National	both	25+	25+	10,015	8,704	Fasting glucose, HbA1c	No	Lab	Lab	
415	Iran	2015-2017	The PERSIAN Yazd Cohort Study	Community	urban	30-70	30-70	4,861	5,002	Fasting glucose	Yes	Lab		
416	Iran	2016-2018	The PERSIAN Ahvaz Cohort Study	Community	rural	35-70	35-70	2,282	1,479	Fasting glucose	Yes	Lab		
417	Iran	2016-2018	The PERSIAN Ahvaz Cohort Study	Community	urban	35-70	35-70	3,554	2,512	Fasting glucose	Yes	Lab		
418	Iran	2016-2018	The PERSIAN BandarKong Cohort Study	Community	rural	35-70	35-70	366	234	Fasting glucose	Yes	Lab		
419	Iran	2016-2018	The PERSIAN BandarKong Cohort Study	Community	urban	35-70	35-70	1,902	1,485	Fasting glucose	Yes	Lab		
420	Iran	2017	Northwest Iran - population based blood sample programme	Community	urban	21+	21+	317	176	Fasting glucose, HbA1c	No	Lab	Lab	
421	Iran	2016-2017	IraPEN Study	Community	rural	30+	30+	3,173	2,919	Fasting glucose	No	Portable		
422	Iran	2016-2017	Iranian Children and Adolescents Psychiatric Disorders (IRCAP) Survey	Subnational	both	18	18	16	15	Fasting glucose	No	Lab		
423	Iran	2016-2018	The PERSIAN Urmia Cohort Study	Community	rural	35-70	35-70	2,379	1,821	Fasting glucose	Yes	Lab		
424	Iran	2016-2018	The PERSIAN Urmia Cohort Study	Community	urban	35-70	35-70	484	423	Fasting glucose	Yes	Lab		
425	Iran	2015-2018	The PERSIAN Zahedan Cohort Study	Community	urban	35-70	35-70	6,024	3,909	Fasting glucose	Yes	Lab		
426	Iran	2016-2020	The PERSIAN Ardabil Cohort Study	Community	urban	35-70	35-70	11,250	9,552	Fasting glucose	Yes	Lab		
427	Iran	2017-2019	The PERSIAN Dena (Yasouj) Cohort Study	Community	rural	35-70	35-70	920	610	Fasting glucose	Yes	Lab		
428	Iran	2017-2019	The PERSIAN Dena (Yasouj) Cohort Study	Community	urban	35-70	35-70	1,126	960	Fasting glucose	Yes	Lab		
429	Iran	2018-2019	Prevalence of risk factors for cardiovascular disease among a rural population in eastern Iran	Community	rural	18-69	18-69	146	152	Fasting glucose	Yes	Lab		
430	Iran	2017-2018	The PERSIAN Kavar Cohort Study	Community	urban	35-70	35-70	2,540	2,418	Fasting glucose	Yes	Lab		
431	Iran	2016-2019	The Khuzestan comprehensive health study: A platform for NCDs, blood borne and mental diseases research	Subnational	both	20-65	20-65	18,413	10,216	Fasting glucose	No	Lab		
432	Iran	2017-2018	PERSIAN Elderly Component-Iranian Longitudinal Study on Ageing	Subnational	urban	50-95	50-95	3,943	3,497	Fasting glucose	Yes	Lab		
433	Iran	2017-2019	The PERSIAN Sabzevar Cohort Study	Community	urban	35-70	35-70	2,341	1,898	Fasting glucose	Yes	Lab		
434	Iran	2016-2019	The PERSIAN Shahrekord Cohort Study	Community	rural	35-70	35-70	1,791	1,233	Fasting glucose	Yes	Lab		
435	Iran	2016-2019	The PERSIAN Shahrekord Cohort Study	Community	urban	35-70	35-70	3,495	3,499	Fasting glucose	Yes	Lab		
436	Iran	2018-2020	Bushehr Elderly Health program Phase II	Community	urban	50-95	50-95	1,120	835	Fasting glucose, HbA1c	Yes	Lab	Lab	
437	Iran	2018-2019	The PERSIAN Dehgolan (Kordistan) Cohort Study	Community	urban	35-70	35-70	2,206	1,748	Fasting glucose	Yes	Lab		
438	Iran	2020-2021	STEPS	National	both	25+	25+	13,979	11,202	Fasting glucose, HbA1c	Yes	Lab	Lab	
439	Iraq	2006	STEPS	National	both	25-65	25-65	2,379	1,817	Fasting glucose	No	Lab		
440	Iraq	2015	STEPS	National	both	18+	18+	1,530	959	Fasting glucose	Yes	Lab		
441	Ireland	2006-2007	Survey of Lifestyle, Attitudes and Nutritional in Ireland 2006-2007	National	both	45+	45+	680	526	HbA1c	Yes		Lab	
442	Ireland	2008-2010	National Adult Nutrition Survey	National	both	18+	18+	440	445	Fasting glucose	No	Lab		
443	Israel	1990-1991	The Jerusalem Longitudinal Cohort Study	Community	urban	69-70	69-70	207	249	Fasting glucose	Yes	Lab		
444	Israel	1997-1998	The Jerusalem Longitudinal Cohort Study	Community	urban	76-77	76-77	454	446	Fasting glucose	Yes	Lab		
445	Israel	1999-2005	The Israel Glucose Intolerance, Obesity and Hypertension Study (GOH)	National	urban	58-93	58-93	607	536	Fasting glucose	Yes	Lab		
446	Israel	2002-2008	The Hadera District Study (HDS)	Subnational	urban	25-78	25-78	551	550	Fasting glucose	Yes	Lab		
447	Israel	2005-2006	The Jerusalem Longitudinal Cohort Study	Community	urban	83-85	83-85	635	522	Fasting glucose	Yes	Lab		
448	Israel	2010-2011	The Jerusalem Longitudinal Cohort Study	Community	urban	89-92	89-92	347	259	Fasting glucose	Yes	Lab		
449	Italy	1982	Verrillo et al, Diabetes Res 2:301-6, 1985	Community	rural	18+	18+	476	410	Fasting glucose	No	Unknown		
450	Italy	1983-1985	Gubbio Study	Community	both	18+	18+	2,512	2,040	Fasting glucose	Yes	Lab		
451	Italy	1983-1984	Malattie cardiovascolari ATerosclerotiche Istituto Superiore di Sanità (MATISS)	Community	rural	19-69	19-69	1,903	1,700	Fasting glucose	Yes	Lab		
452	Italy	1986-1987	Malattie cardiovascolari ATerosclerotiche Istituto Superiore di Sanità (MATISS)	Community	rural	19-72	19-72	1,479	1,209	Fasting glucose	Yes	Lab		

	Country	Data years	Survey/Study name/Citation	Level of representative-ness	Rural, urban, or both	Age range as used for global analysis		Sample size used for global analysis		Glycaemic markers available	Information available on diabetes treatment	Whether measured in a laboratory or by a point-of-care portable device		Notes
						Female	Male	Female	Male			Glucose	HbA1c	
453	Italy	1986-1987	MONICA, Brianza	Subnational	both	25-64	25-64	803	787	Fasting glucose	No	Lab		
454	Italy	1989	Ventimiglia Heart Study	Community	rural	18+	18+	599	500	Fasting glucose	Yes	Lab		
455	Italy	1990	Bruneck Study	Community	rural	40-79	40-79	450	469	Fasting glucose	Yes	Lab		
456	Italy	1989-1990	MONICA, Brianza	Subnational	both	25-64	25-64	769	785	Fasting glucose	No	Lab		
457	Italy	1991	DECODE; DECODE Study Group, Diabetes Care 26:61-9, 2003	Community	urban	40-89	40-89	1,013	804	Fasting glucose	No	Unknown		
458	Italy	1991	Finland, Italy, Netherlands, Elderly (Fine-Italy)	Community	rural		70-90		423	Fasting glucose	Yes	Lab		
459	Italy	1989-1992	Gubbio Study	Community	both	18+	18+	1,886	1,549	Fasting glucose	Yes	Lab		
460	Italy	1992-1993	Italian Longitudinal Study on Aging	National	both	65-84	65-84	2,146	2,271	Fasting glucose	Yes	Lab		
461	Italy	1993-1996	Malattie cardiovascolari ATerosclerotiche Istituto Superiore di Sanità (MATISS)	Community	rural	20-77	20-77	991	963	Fasting glucose	Yes	Lab		
462	Italy	1993-1994	MONICA, Brianza	Subnational	urban	25-64	25-64	774	740	Fasting glucose	Yes	Lab		
463	Italy	1995	Vobarno Study; Muiesan et al, Blood Press 15:14-9, 2006	Community	both	35-64	35-64	309	265	Fasting glucose	No	Unknown		
464	Italy	1995	Bruneck Study	Community	rural	45-84	45-84	410	411	Fasting glucose	Yes	Lab		
465	Italy	1995-1996	Italian Longitudinal Study on Aging	National	both	68-90	68-90	1,413	1,541	Fasting glucose	Yes	Lab		
466	Italy	1995-1999	PROgetto Veneto Anziani (PROVA)	Subnational	both	65+	65+	1,853	1,243	Fasting glucose	Yes	Lab		
467	Italy	1999	InCHIANTI Study; Ferrrucci et al., J Am Geriatr Soc 48:1618-25, 2000	Community	both	25+	25+	725	582	Fasting glucose	No	Unknown		
468	Italy	1998-1999	Progetto VIP	Community	both	25-74	25-74	601	599	Fasting glucose	Yes	Lab		
469	Italy	2000	Bruneck Study	Community	rural	50-89	50-89	361	331	Fasting glucose, HbA1c	Yes	Lab	Lab	
470	Italy	2000	Finland, Italy, Netherlands, Elderly (Fine-Italy)	Community	rural		80-100		225	Fasting glucose	Yes	Lab		
471	Italy	1998-2002	Osservatorio Epidemiologico Cardiovascolare (OEC)	National	both	35-74	35-74	4,771	4,878	Fasting glucose	Yes	Lab		
472	Italy	2000-2001	Italian Longitudinal Study on Aging	National	both	73-93	73-93	987	941	Fasting glucose	Yes	Lab		
473	Italy	2002	Vobarno Study; Muiesan et al, Blood Press 15:14-9, 2006	Community	both	25-64	25-64	216	169	Fasting glucose	No	Unknown		
474	Italy	2000-2003	PROgetto Veneto Anziani (PROVA)	Subnational	both	67+	67+	1,377	813	Fasting glucose	Yes	Lab		
475	Italy	2001-2004	Scuteri et al., Nutr Metab Cardiovasc Dis 19:532-41, 2009	Community	urban	18+	18+	2,265	1,697	Fasting glucose	No	Lab		
476	Italy	2003	The European Male Ageing Study	Community	both		40+		428	Fasting glucose	Yes	Lab		
477	Italy	2001-2007	Gubbio Study	Community	both	26+	26+	1,462	1,191	Fasting glucose	Yes	Lab		
478	Italy	2002-2005	PROgetto Veneto Anziani (PROVA)	Subnational	both	68+	68+	1,147	629	Fasting glucose	Yes	Lab		
479	Italy	2005	Bruneck Study	Community	rural	55-93	55-93	307	263	Fasting glucose, HbA1c	Yes	Lab	Lab	
480	Italy	2004-2005	Italian Project on the Epidemiology of Alzheimer's Disease	National	both	65-84	65-84	1,444	1,589	Fasting glucose	Yes	Lab		
481	Italy	2004-2005	Vobarno study	Community	rural	55-74	55-74	113	99	Fasting glucose	Yes	Lab		
482	Italy	2005-2007	Moli-family Study	Subnational	both	18+	18+	270	216	Fasting glucose	Yes	Lab		
483	Italy	2008	The European Male Ageing Study	Community	both		45+		344	Fasting glucose	Yes	Lab		
484	Italy	2005-2010	Moli-sani Study	Subnational	both	35+	35+	12,366	11,495	Fasting glucose	Yes	Lab		
485	Italy	2008-2009	Progetto VIP	Community	both	25-74	25-74	600	600	Fasting glucose	Yes	Lab		
486	Italy	2010	Bruneck Study	Community	rural	60+	60+	259	225	Fasting glucose, HbA1c	Yes	Lab	Lab	
487	Italy	2009-2010	Factors associated with metabolic syndrome in a mediterranean population: role of caffeinated beverages	Community	both	19-88	19-88	1,119	752	Fasting glucose	No	Unknown		
488	Italy	2008-2012	Osservatorio Epidemiologico Cardiovascolare/Health Examination Survey (OEC/HES)	National	both	35-80	35-80	4,330	4,369	Fasting glucose	Yes	Lab		
489	Italy	2010-2012	CArdiovascular risk MEtabolic syndrome LIver and Autoimmunity diseases (CA.ME.LI.A)	Community	both	18-75	18-75	506	466	Fasting glucose	Yes	Lab		
490	Italy	2011-2012	Vobarno study	Community	rural	49-62	49-62	143	107	Fasting glucose	Yes	Lab		
491	Italy	2015	Bruneck Study	Community	rural	65+	65+	169	171	Fasting glucose, HbA1c	Yes	Lab	Lab	
492	Italy	2016	The Tyrolean Early Vascular Ageing-study (EVA-Tyrol) - South-Tyrol	Subnational	both		18		2	Fasting glucose, HbA1c	Yes	Lab	Lab	
493	Italy	2018-2019	Progetto VIP	Community	both	25-74	25-74	598	600	Fasting glucose	Yes	Lab		
494	Italy	2017-2020	Moli-sani Study	Subnational	both	47-94	47-94	1,309	1,081	Fasting glucose	Yes	Lab		
495	Jamaica	1991	Eldemire et al, West Indian Med J 45:82-4, 1996	National	both	60+	60+	669	649	Fasting glucose	No	Unknown		
496	Jamaica	1995	MacFarlane-Anderson et al., Metabolism 47:617-21, 1998	Community	urban	25+	25+	329	233	Fasting glucose	No	Unknown		
497	Jamaica	2000-2001	Jamaica Health and Lifestyle Survey	National	both	18-74	18-74	1,249	622	Fasting glucose	Yes	Portable		
498	Jamaica	2006-2007	Jamaica Youth Risk and Resiliency Behaviour Survey 2006	National	both	18-19	18-19	192	152	Fasting glucose	Yes	Portable		
499	Jamaica	2007-2008	Jamaica Health and Lifestyle Survey	National	both	18-74	18-74	1,869	842	Fasting glucose	Yes	Portable		
500	Jamaica	2012	Older Persons in Jamaica 2012	National	both	60+	60+	208	156	HbA1c	Yes		Lab	13
501	Jamaica	2016-2017	Jamaica Health and Lifestyle Survey	National	both	18+	18+	1,633	1,026	Fasting glucose, HbA1c	Yes	Portable	Portable	
502	Japan	1985-1986	Akabane Study	Community	urban	40-69	40-69	593	471	Fasting glucose	No	Unknown		
503	Japan	1987	Konan Town Study	Community	rural	20-79	20-79	87	69	Fasting glucose	No	Unknown		
504	Japan	1988	Konan Town Study	Community	rural	20-79	20-79	85	76	Fasting glucose	No	Unknown		
505	Japan	1988	The Hisayama Study	Community	rural	40+	40+	1,574	1,162	Fasting glucose	Yes	Lab		
506	Japan	1989	Konan Town Study	Community	rural	20-79	20-79	63	59	Fasting glucose	No	Unknown		
507	Japan	1989	National Nutrition Survey	National	both	30+	30+	1,613	1,377	Fasting glucose	No	Lab		
508	Japan	1990	Konan Town Study	Community	rural	20-79	20-79	58	30	Fasting glucose	No	Unknown		
509	Japan	1990	National Nutrition Survey and National Cadiovascular Survey	National	both	30+	30+	1,615	1,517	Fasting glucose	No	Lab		
510	Japan	1991	DECODA; DECODA Study Group, Diabetes Care 26:1770-80, 2003	Community	rural	30-89	30-89	5,182	3,896	Fasting glucose	No	Unknown		
511	Japan	1991	Konan Town Study	Community	rural	20-79	20-79	117	93	Fasting glucose	No	Unknown		
512	Japan	1991	National Nutrition Survey	National	both	30+	30+	1,523	1,444	Fasting glucose	No	Lab		
513	Japan	1992	Konan Town Study	Community	rural	20-79	20-79	52	55	Fasting glucose	No	Unknown		
514	Japan	1992	National Nutrition Survey	National	both	30+	30+	1,445	1,332	Fasting glucose	No	Lab		
515	Japan	1993	Konan Town Study	Community	rural	20-79	20-79	65	54	Fasting glucose	No	Unknown		
516	Japan	1993	National Nutrition Survey	National	both	30+	30+	1,360	1,251	Fasting glucose	No	Lab		
517	Japan	1994	Konan Town Study	Community	rural	20-79	20-79	59	42	Fasting glucose	No	Unknown		
518	Japan	1994	National Nutrition Survey	National	both	20-59	20-59	1,112	970	Fasting glucose	No	Lab		

	Country	Data years	Survey/Study name/Citation	Level of representative-ness	Rural, urban, or both	Age range as used for global analysis		Sample size used for global analysis		Glycaemic markers available	Information available on diabetes treatment	Whether measured in a laboratory or by a point-of-care portable device		Notes
						Female	Male	Female	Male			Glucose	HbA1c	
519	Japan	1995	Konan Town Study	Community	rural	20-79	20-79	60	45	Fasting glucose	No	Unknown		
520	Japan	1995	National Nutrition Survey	National	both	20-59	20-59	1,076	948	Fasting glucose	No	Lab		
521	Japan	1996	National Nutrition Survey	National	both	30+	30+	1,060	953	Fasting glucose	No	Lab		
522	Japan	1997	National Nutrition Survey	National	both	20+	20+	1,293	1,196	Fasting glucose	No	Lab		
523	Japan	1998	APCSC-Akabane	Community	urban	35-74	35-74	593	471	Fasting glucose	No	Unknown		
524	Japan	1998	National Nutrition Survey	National	both	20+	20+	1,337	1,135	Fasting glucose	No	Lab		
525	Japan	1998-2000	The Japan Public Health Center-based Prospective Diabetes study: JPHC Diabetes study	Subnational	both	46-75	46-75	7,332	4,493	Fasting glucose	Yes	Lab		
526	Japan	1999	National Nutrition Survey	National	both	20+	20+	1,013	920	Fasting glucose	No	Lab		
527	Japan	2000	Niigata Study	Community	urban	72	72	171	212	HbA1c	No		Lab	
528	Japan	2000	National Nutrition Survey and National Cadiovascular Survey	National	both	20+	20+	1,219	1,073	Fasting glucose	No	Lab		
529	Japan	2001	Nakagami et al., Diabetologia 46:1063-70, 2003	Community	rural	35+	35+	1,016	783	Fasting glucose	No	Unknown		
530	Japan	2001	The Japan Association of Health Service Database	Subnational	both	20+	20+	298,123	323,553	Fasting glucose, HbA1c	No	Lab	Lab	
531	Japan	2001	Niigata Study	Community	urban	73	73	196	234	HbA1c	No		Lab	
532	Japan	2001	National Nutrition Survey	National	both	20+	20+	1,088	968	Fasting glucose	No	Lab		
533	Japan	2002	Niigata Study	Community	urban	74	74	185	211	HbA1c	No		Lab	
534	Japan	2002	National Nutrition Survey	National	both	20+	20+	1,072	952	Fasting glucose	No	Lab		
535	Japan	2002-2003	The Hisayama Study	Community	rural	40+	40+	1,837	1,391	Fasting glucose, HbA1c	Yes	Lab	Lab	
536	Japan	2003	National Health and Nutrition Survey	National	both	20+	20+	3,534	2,475	Fasting glucose, HbA1c	Yes	Lab	Lab	
537	Japan	2003	Niigata Study	Community	urban	75	75	191	215	HbA1c	No		Lab	
538	Japan	2004	National Health and Nutrition Survey	National	both	20+	20+	2,700	1,879	Fasting glucose, HbA1c	Yes	Lab	Lab	
539	Japan	2004	Niigata Study	Community	urban	76	76	184	212	HbA1c	No		Lab	
540	Japan	2003-2006	The Japan Public Health Center-based Prospective Diabetes study: JPHC Diabetes study	Subnational	both	51-80	51-81	4,731	3,068	Fasting glucose, HbA1c	Yes	Unknown	Lab	
541	Japan	2005	National Health and Nutrition Survey	National	both	20+	20+	2,540	1,824	Fasting glucose, HbA1c	Yes	Lab	Lab	
542	Japan	2005	Niigata Study	Community	urban	77	77	189	202	HbA1c	No		Lab	
543	Japan	2006	National Health and Nutrition Survey	National	both	20+	20+	2,862	2,055	Fasting glucose, HbA1c	Yes	Lab	Lab	
544	Japan	2006	Niigata Study	Community	urban	78	78	193	190	HbA1c	No		Lab	
545	Japan	2007	National Health and Nutrition Survey	National	both	20+	20+	2,734	1,976	Fasting glucose, HbA1c	Yes	Lab	Lab	
546	Japan	2007	Niigata Study	Community	urban	79	79	192	181	HbA1c	No		Lab	
547	Japan	2008	Study on residents in Kanazawa City aged over 40	Community	urban	40+	40+	11,944	6,562	Fasting glucose	No	Unknown		
548	Japan	2008	National Health and Nutrition Survey	National	both	20+	20+	2,939	2,149	Fasting glucose, HbA1c	Yes	Lab	Lab	
549	Japan	2008	Niigata Study	Community	urban	80	80	162	160	HbA1c	No		Lab	
550	Japan	2009	National Health and Nutrition Survey	National	both	20+	20+	2,851	2,018	Fasting glucose, HbA1c	Yes	Lab	Lab	
551	Japan	2010	National Health and Nutrition Survey	National	both	20+	20+	2,649	1,977	Fasting glucose, HbA1c	Yes	Lab	Lab	
552	Japan	2011	National Health and Nutrition Survey	National	both	20+	20+	2,411	1,776	Fasting glucose, HbA1c	Yes	Lab	Lab	
553	Japan	2011	The Tokyo Health Service Association Database	Community	urban	20+	20+	5,232	6,426	Fasting glucose, HbA1c	No	Lab	Lab	
554	Japan	2012	National Health and Nutrition Survey	National	both	20+	20+	9,670	7,007	HbA1c	Yes		Lab	
555	Japan	2013	National Health and Nutrition Survey	National	both	20+	20+	2,310	1,765	Fasting glucose, HbA1c	Yes	Lab	Lab	
556	Japan	2011-2016	Japan Public Health Center-based Prospective Study for the Next Generation (JPHC-NEXT Study)	Subnational	both	40-73	40-73	5,629	5,003	Fasting glucose, HbA1c	Yes	Unknown	Lab	
557	Japan	2012-2016	The Nagahama Study	Community	rural	35-80	35-80	6,107	2,915	Fasting glucose, HbA1c	Yes	Lab	Lab	
558	Japan	2014	National Health and Nutrition Survey	National	both	20+	20+	2,396	1,843	Fasting glucose, HbA1c	Yes	Lab	Lab	
559	Japan	2015	National Health and Nutrition Survey	National	both	20+	20+	2,297	1,662	Fasting glucose, HbA1c	Yes	Lab	Lab	
560	Japan	2016	National Health and Nutrition Survey	National	both	20+	20+	7,918	5,819	HbA1c	Yes		Lab	
561	Japan	2017	National Health and Nutrition Survey	National	both	20+	20+	2,037	1,556	Fasting glucose, HbA1c	Yes	Lab	Lab	
562	Japan	2015-2019	The Shizuoka KDB study	Subnational	both	75-90	75-90	4,337	2,109	Fasting glucose, HbA1c	Yes	Lab	Lab	
563	Japan	2017	The Tokyo Health Service Association Database	Community	urban	20+	20+	14,915	27,734	Fasting glucose, HbA1c	Yes	Unknown	Unknown	
564	Japan	2018	National Health and Nutrition Survey	National	both	20+	20+	2,041	1,528	Fasting glucose, HbA1c	Yes	Lab	Lab	
565	Japan	2019	National Health and Nutrition Survey	National	both	20+	20+	1,645	1,260	Fasting glucose, HbA1c	Yes	Lab	Lab	
566	Japan	2022-2023	The Tokyo Health Service Association Database	Community	urban	20-79	20-79	28,652	43,488	Fasting glucose, HbA1c	Yes	Lab	Lab	
567	Jordan	2004	Behavioural Risk Factor Surveillence Survey	National	rural	18+	18+	1,974	1,351	Fasting glucose	Yes	Lab		
568	Jordan	2007	Behavioural Risk Factor Surveillence Survey	National	both	18+	18+	1,705	1,949	Fasting glucose	Yes	Lab		
569	Jordan	2009	Metablic abnomalities and vitamin D study	National	both	18+	18+	3,355	1,144	Fasting glucose	Yes	Lab		
570	Jordan	2016-2017	National Cardiovascular Diseases and Diabetes Study (NCDDS)	National	both	18+	18+	2,763	1,182	Fasting glucose, HbA1c	Yes	Portable	Lab	
571	Jordan	2019	STEPS	National	both	18-69	18-69	3,324	2,202	Fasting glucose	Yes	Portable		
572	Kazakhstan	2011-2012	Household Health Survey	National	both	18+	18+	539	399	Fasting glucose	No	Portable		
573	Kazakhstan	2015	Almaty STEPS	Subnational	both	18-69	18-69	1,138	377	Fasting glucose	Yes	Lab		
574	Kazakhstan	2015	Shymkent STEPS	Subnational	both	18-69	18-69	925	428	Fasting glucose	Yes	Lab		
575	Kazakhstan	2015-2016	Aktobe STEPS	Subnational	both	18-69	18-69	1,138	347	Fasting glucose	Yes	Lab		
576	Kazakhstan	2019	A health status assessment of a population of Karaganda urban region	Community	urban	18+	18+	664	315	Fasting glucose, HbA1c	No	Lab	Lab	
577	Kazakhstan	2021	Prevalence of NCD Risk Factors in Kazakhstan	Subnational	both	18-69	18-69	813	801	Fasting glucose, HbA1c	Yes	Lab	Lab	14
578	Kazakhstan	2021-2022	Prevalence of risk factors for NCD in Kazakhstan	National	both	18-69	18-69	1,997	1,811	Fasting glucose, HbA1c	Yes	Lab	Lab	
579	Kenya	2015	STEPS	National	both	18-69	18-69	2,527	1,763	Fasting glucose	Yes	Unknown		
580	Kiribati	1981	Epidemiological survey of Kiribati	Subnational	rural	20+	20+	533	474	Fasting glucose	Yes	Lab		
581	Kiribati	1981	Epidemiological survey of Kiribati	Subnational	urban	20+	20+	879	917	Fasting glucose	Yes	Lab		
582	Kiribati	2004	STEPS	National	both	18-64	18-64	897	727	Fasting glucose	Yes	Portable		
583	Kiribati	2015-2016	STEPS	National	both	18-69	18-69	1,141	963	Fasting glucose	Yes	Portable		
584	Kuwait	2006	STEPS	National	both	20-64	20-64	1,298	918	Fasting glucose	Yes	Lab		

	Country	Data years	Survey/Study name/Citation	Level of representative-ness	Rural, urban, or both	Age range as used for global analysis		Sample size used for global analysis		Glycaemic markers available	Information available on diabetes treatment	Whether measured in a laboratory or by a point-of-care portable device		Notes
						Female	Male	Female	Male			Glucose	HbA1c	
585	Kuwait	2008-2009	National Nutrition Program for the State of Kuwait	National	both	18-86	18-86	530	459	Fasting glucose, HbA1c	Yes	Lab	Lab	15
586	Kuwait	2011-2014	Kuwait Diabetes Epidemiology Program	National	both	18-82	18-82	2,144	2,781	Fasting glucose, HbA1c	Yes	Lab	Lab	
587	Kuwait	2014	STEPS	National	both	18-69	18-69	2,406	1,458	Fasting glucose, HbA1c	Yes	Lab	Lab	
588	Kyrgyzstan	2013	STEPS	National	both	25-64	25-64	1,605	946	Fasting glucose	Yes	Portable		
589	Lao PDR	2013	STEPS	National	both	18-64	18-64	1,471	989	Fasting glucose	Yes	Portable		
590	Latvia	2008-2009	Cardiovascular risk factor study	National	both	25-74	25-74	2,394	1,359	Fasting glucose	No	Lab		
591	Lebanon	2017	STEPS	National	both	18-69	18-69	1,086	790	Fasting glucose	Yes	Lab		
592	Lebanon	2023-2024	STEPS	National	both	18-69	18-69	1,820	1,053	Fasting glucose, HbA1c	Yes	Portable	Unknown	2
593	Lesotho	2012	STEPS	National	both	25-64	25-64	1,492	774	Fasting glucose	Yes	Portable		
594	Liberia	2011	STEPS	Subnational	both	25-64	25-64	1,316	1,060	Fasting glucose	Yes	Portable		
595	Liberia	2022	STEPS	National	both	18-69	18-69	2,367	1,532	Fasting glucose	Yes	Portable		
596	Libya	1999	Kadiki et al., Diabetes Metab 27:647-54, 2001	Community	both	25-84	25-84	388	211	Fasting glucose	No	Unknown		
597	Libya	2009	STEPS	National	both	25-64	25-64	1,622	1,712	Fasting glucose	Yes	Portable		
598	Libya	2022-2023	STEPS	National	both	18-69	18-69	2,599	2,271	Fasting glucose, HbA1c	Yes	Portable	Lab	2
599	Lithuania	2001-2002	MONICA4	Community	urban	35-64	35-64	760	609	Fasting glucose	Yes	Portable		
600	Lithuania	2006-2008	Health, Alcohol and Psychosocial Factors In Eastern Europe	Community	urban	45-70	45-70	3,497	2,906	Fasting glucose	Yes	Portable		
601	Luxembourg	2007-2009	Observation of cardiovascular risk factors in Luxembourg (ORISCAV-LUX)	National	both	18-69	18-69	735	697	Fasting glucose, HbA1c	Yes	Lab	Lab	
602	Luxembourg	2013-2015	European Health Examination Survey in Luxembourg	National	both	25-64	25-64	781	721	Fasting glucose, HbA1c	Yes	Lab	Lab	
603	Luxembourg	2016-2018	Observation of cardiovascular risk factors in Luxembourg (ORISCAV-LUX2)	Community	both	25-79	25-79	825	731	Fasting glucose, HbA1c	Yes	Lab	Lab	
604	Malawi	2009	STEPS	National	both	25-64	25-64	3,252	1,690	Fasting glucose	Yes	Portable		
605	Malawi	2013-2017	NCD Survey Malawi Epidemiology and Intervention Research Unit	Community	rural	18+	18+	7,497	5,830	Fasting glucose	Yes	Lab		
606	Malawi	2013-2017	NCD Survey Malawi Epidemiology and Intervention Research Unit	Community	urban	18+	18+	10,291	5,799	Fasting glucose	Yes	Lab		
607	Malawi	2017	STEPS	National	both	18-69	18-69	2,560	1,485	Fasting glucose	Yes	Portable		
608	Malaysia	2004	Rampal et al., Public Health 122(1):11-8, 2008	National	both	18+	18+	9,840	7,362	Fasting glucose	Yes	Lab		
609	Malaysia	2006	National Health and Morbidity Survey (NHMS)	National	both	18+	18+	18,176	15,274	Fasting glucose	Yes	Portable		
610	Malaysia	2008	Metabolic Syndrome Study in Malaysia	National	rural	18+	18+	1,360	749	Fasting glucose, HbA1c	Yes	Lab	Lab	
611	Malaysia	2008	Metabolic Syndrome Study in Malaysia	National	urban	18+	18+	1,435	765	Fasting glucose, HbA1c	Yes	Lab	Lab	
612	Malaysia	2011	National Health and Morbidity Survey (NHMS)	National	both	18+	18+	8,844	8,090	Fasting glucose	Yes	Portable		
613	Malaysia	2015	National Health and Morbidity Survey (NHMS)	National	both	18+	18+	10,411	9,439	Fasting glucose	Yes	Portable		
614	Malaysia	2019	National Health and Morbidity Survey (NHMS)	National	both	18+	18+	5,555	4,783	Fasting glucose	Yes	Portable		
615	Maldives	2004	STEPS	Subnational	urban	25-64	25-64	839	716	Fasting glucose	No	Lab		
616	Maldives	2020-2021	STEPS	National	both	18-69	18-69	1,994	886	Fasting glucose	Yes	Portable		
617	Mali	2013	STEPS	Subnational	both	18-65	18-65	805	446	Fasting glucose	Yes	Portable		
618	Malta	1981	DECODE; DECODE Study Group, Diabetes Care 26:61-9, 2003	Community	both	30-89	30-89	1,173	870	Fasting glucose	No	Unknown		
619	Malta	2014-2016	SAHHTEK - The University of Malta Health and Wellbeing Study	National	both	18-70	18-70	1,022	834	Fasting glucose	Yes	Lab		
620	Marshall Islands	2002	STEPS	National	both	18-64	18-64	1,532	1,081	Fasting glucose	Yes	Portable		
621	Marshall Islands	2017-2018	STEPS	National	both	18+	18+	1,556	1,406	Fasting glucose	Yes	Unknown		
622	Mauritania	2006	STEPS	Community	urban	18-64	18-64	1,126	1,023	Fasting glucose	Yes	Lab		
623	Mauritius	1987	Mauritius Noncommunicable Disease Survey	National	both	25-74	25-74	2,662	2,355	Fasting glucose	Yes	Lab		
624	Mauritius	1992	Mauritius Noncommunicable Disease Survey	National	both	25-74	25-74	3,481	2,994	Fasting glucose	Yes	Lab		
625	Mauritius	1992	Rodrigues, Mauritius (1992)	Community	rural	25-64	25-64	774	737	Fasting glucose	Yes	Lab		
626	Mauritius	1998	Mauritius Noncommunicable Disease Survey	National	both	25-74	25-74	3,248	2,567	Fasting glucose	Yes	Lab		
627	Mauritius	1999	Rodrigues, Mauritius (1999)	Community	rural	20+	20+	1,295	977	Fasting glucose	Yes	Lab		
628	Mauritius	2009	Mauritius Noncommunicable Disease Survey	National	both	19-74	19-74	3,432	2,903	Fasting glucose	Yes	Lab		
629	Mauritius	2015	Mauritius Noncommunicable Disease Survey	National	both	20-74	20-74	1,948	1,626	Fasting glucose, HbA1c	Yes	Lab	Lab	
630	Mauritius	2015	Mauritius Noncommunicable Disease Survey 1998 Follow Up	National	both	20+	20+	1,171	886	Fasting glucose, HbA1c	Yes	Lab	Lab	
631	Mexico	2004-2005	CArdiovascular Risk factors Multiple Evaluation in Latin America (CARMELA)	Community	urban	25-64	25-64	889	833	Fasting glucose	Yes	Lab		
632	Mexico	2006	Encuesta Nacional de Salud y Nutrición	National	both	20+	20+	24,881	19,673	Fasting glucose	Yes	Unknown		
633	Mexico	2009-2012	Encuesta Nacional Sobre Niveles de vida de los Hogares	National	both	18+	18+	10,495	8,215	HbA1c	Yes		Portable	
634	Mexico	2012	The Mexican Health and Aging Study	National	both	50+	50+	8,399	6,441	HbA1c	Yes		Portable	
635	Mexico	2016	Encuesta Nacional de Salud y Nutrición	National	both	20+	20+	5,477	2,865	Fasting glucose, HbA1c	Yes	Lab	Lab	
636	Mexico	2016	Cognitive Aging Linked to MHAS (Mex-Cog)	National	both	55+	55+	1,191	833	HbA1c	Yes		Portable	16
637	Mexico	2018-2019	Encuesta Nacional de Salud y Nutrición	National	both	20+	20+	9,798	7,584	Fasting glucose, HbA1c	Yes	Lab	Lab	
638	Mexico	2020	Encuesta Nacional de Salud y Nutrición	National	both	20+	20+	1,359	932	Fasting glucose, HbA1c	No	Lab	Lab	
639	Mexico	2021	Encuesta Nacional de Salud y Nutrición	National	both	20+	20+	5,243	2,947	Fasting glucose, HbA1c	Yes	Lab	Lab	
640	Mexico	2022	Encuesta Nacional de Salud y Nutrición	National	both	20+	20+	6,627	4,338	Fasting glucose, HbA1c	Yes	Lab	Lab	
641	Micronesia	2002	STEPS	Subnational	both	25-64	25-64	866	580	Fasting glucose	Yes	Lab		
642	Micronesia	2006	STEPS	Subnational	both	25-64	25-64	1,246	702	Fasting glucose	Yes	Unknown		
643	Micronesia	2008	STEPS	Subnational	both	25-64	25-64	1,255	857	Fasting glucose	Yes	Unknown		
644	Micronesia	2009	STEPS, Kosrae	Subnational	both	18-64	18-64	446	246	Fasting glucose	Yes	Portable		
645	Micronesia	2009	STEPS, Yap	Subnational	both	18-64	18-64	562	435	Fasting glucose	Yes	Unknown		
646	Moldova	2013	STEPS	National	both	18-69	18-69	2,893	1,806	Fasting glucose	Yes	Portable		
647	Moldova	2021	STEPS	National	both	18-69	18-69	2,289	1,774	Fasting glucose	Yes	Portable		
648	Mongolia	2005	STEPS	National	both	25-64	25-64	1,374	1,323	Fasting glucose	Yes	Portable		
649	Mongolia	2009	STEPS	National	both	25-64	25-64	2,658	1,814	Fasting glucose	Yes	Unknown		
650	Mongolia	2013	STEPS	National	both	25-64	25-64	2,366	1,921	Fasting glucose	Yes	Portable		

	Country	Data years	Survey/Study name/Citation	Level of representative-ness	Rural, urban, or both	Age range as used for global analysis		Sample size used for global analysis		Glycaemic markers available	Information available on diabetes treatment	Whether measured in a laboratory or by a point-of-care portable device		Notes
						Female	Male	Female	Male			Glucose	HbA1c	
651	Mongolia	2019	STEPS	National	both	18-69	18-69	3,481	2,885	Fasting glucose	Yes	Portable		
652	Morocco	2000	National Survey 2000	National	both	20-89	20-89	973	689	Fasting glucose	No	Lab		
653	Morocco	2017	STEPS	National	both	18+	18+	3,417	1,891	Fasting glucose	Yes	Portable		
654	Mozambique	2014-2015	STEPS	National	both	18-64	18-64	1,644	1,111	Fasting glucose	Yes	Portable		
655	Myanmar	2003-2004	STEPS	Subnational	both	25-74	25-74	2,453	1,991	Fasting glucose	Yes	Lab		
656	Myanmar	2014	National survey of Diabetes Mellitus and risk factors for Non-communicable diseases in Myanmar	National	both	25-64	25-64	5,592	3,079	Fasting glucose	Yes	Portable		
657	Myanmar	2013-2014	STEPS, Yangon	Subnational	both	25-74	25-74	740	745	Fasting glucose	Yes	Lab		
658	Namibia	2013	Demographic and Health Survey Namibia 2013	National	both	35-64	35-64	2,054	1,551	Fasting glucose	Yes	Portable		
659	Nauru	1982	Trends in the prevalence and incidence of non-insulin-dependent diabetes mellitus and impaired glucose tolerance	National	both	20+	20+	775	706	Fasting glucose	Yes	Unknown		
660	Nauru	1987	Trends in the prevalence and incidence of non-insulin-dependent diabetes mellitus and impaired glucose tolerance	National	both	20+	20+	662	554	Fasting glucose	Yes	Lab		
661	Nauru	1994	Trends in the prevalence and incidence of non-insulin-dependent diabetes mellitus and impaired glucose tolerance	National	both	25+	25+	735	652	Fasting glucose	Yes	Lab		
662	Nauru	2006	STEPS	National	both	18-65	18-65	231	245	Fasting glucose	Yes	Lab		
663	Nauru	2015	STEPS	National	both	18-69	18-69	704	649	Fasting glucose	Yes	Portable		
664	Nepal	1990	Sasaki et al., Diabetes Res Clin Pract 67:167-74, 2005	Community	rural		20+		85	Fasting glucose	No	Unknown		
665	Nepal	2000	Singh et al., Diabet Med 20:170-1, 2003	Subnational	rural	20+	20+	235	105	Fasting glucose	No	Unknown		
666	Nepal	2000	Singh et al., Diabet Med 20:170-1, 2003	Subnational	urban	20+	20+	456	442	Fasting glucose	No	Unknown		
667	Nepal	2006-2011	Early detection and management of Kidney disease, Hypertension, Diabetes and Cardiovascular disease (KHDC Nepal), Tarahara	Community	rural	18+	18+	2,351	1,176	Fasting glucose	Yes	Lab		
668	Nepal	2006-2011	Early detection and management of Kidney disease, Hypertension, Diabetes and Cardiovascular disease (KHDC Nepal), Damak	Community	urban	18+	18+	1,577	1,095	Fasting glucose	Yes	Lab		
669	Nepal	2006-2011	Early detection and management of Kidney disease, Hypertension, Diabetes and Cardiovascular disease (KHDC Nepal), Dharan	Community	urban	18+	18+	6,130	4,130	Fasting glucose	Yes	Lab		
670	Nepal	2012-2013	STEPS	National	both	18-69	18-69	2,702	1,276	Fasting glucose	Yes	Lab		
671	Nepal	2015	Community based intervention for prevention and control of non-communicable diseases risk factors (CIPCON) baseline survey, Dhankuta	Subnational	rural	18-69	18-69	779	555	Fasting glucose	Yes	Lab		
672	Nepal	2015	Community based intervention for prevention and control of non-communicable diseases risk factors (CIPCON) baseline survey, Ilam	Subnational	rural	18-69	18-69	717	553	Fasting glucose	Yes	Lab		
673	Nepal	2016-2018	The Population Based Prevalence of Selected Non-Communicable Diseases In Nepal	National	both	20+	20+	7,423	4,834	Fasting glucose	Yes	Lab		
674	Nepal	2019	STEPS	National	both	18-69	18-69	3,400	1,910	Fasting glucose	Yes	Portable		
675	Netherlands	1990	DECODE; DECODE Study Group, Diabetes Care 26:61-9, 2003	Community	urban	50-79	50-79	1,322	1,131	Fasting glucose	No	Unknown		
676	Netherlands	1990	Zutphen Elderly Study	Community	urban		69-90		555	Fasting glucose	Yes	Lab		
677	Netherlands	1995-1996	The Longitudinal Aging Study Amsterdam (LASA)	Subnational	both	65-88	65-88	780	725	Fasting glucose	Yes	Unknown		17
678	Netherlands	1998-2001	Regenboog Project	National	both	18-89	18-89	2,305	2,323	Fasting glucose	Yes	Lab		
679	Netherlands	2001-2003	Surinamese in the Netherlands: Study on Ethnicity and Health (SUNSET)	Community	urban	35-60	35-60	257	251	Fasting glucose	Yes	Lab		
680	Netherlands	2008-2009	The Longitudinal Aging Study Amsterdam (LASA)	Subnational	both	60-100	60-100	827	665	Fasting glucose	Yes	Unknown		17
681	Netherlands	2012-2013	The Longitudinal Aging Study Amsterdam (LASA)	Subnational	both	55-65	55-65	454	433	Fasting glucose	Yes	Unknown		17
682	New Zealand	2008-2009	New Zealand Adult Nutrition Survey	National	both	18+	18+	2,284	1,806	HbA1c	Yes		Lab	
683	Nicaragua	2003-2004	CAMDI	Community	urban	20+	20+	919	781	Fasting glucose	Yes	Lab		
684	Niger	2021	STEPS	National	both	18-69	18-69	3,076	2,283	Fasting glucose	Yes	Portable		
685	Nigeria	1998	Okesina et al., East Afr Med J 76:212-6, 1999	Community	rural	21+	21+	120	222	Fasting glucose	No	Unknown		
686	Nigeria	1999-2009	Prostate cancer dietary risk factors study	Subnational	both		35+		447	Fasting glucose	No	Portable		
687	Nigeria	2006	Clustering of cardiovascular disease risk-factors in semiurban population in Northern Nigeria	Community	urban	18+	18+	106	87	Fasting glucose	No	Lab		
688	Nigeria	2007	Southeast Nigeria kidney disease study	Community	rural	25-64	25-64	442	169	Fasting glucose	Yes	Portable		
689	Nigeria	2009-2011	Anthropometric indices in Calabar	Community	urban	18-79	18-79	331	381	Fasting glucose	No	Lab		
690	Norway	2001-2002	The Tromsø Study: Tromsø 5, Tromsø Study Panel	Community	both	30-89	30-89	3,576	2,536	HbA1c	Yes		Lab	
691	Norway	2007-2008	The Tromsø Study: Tromsø 6	Community	both	30-87	30-87	6,709	5,927	HbA1c	Yes		Lab	
692	Norway	2017-2019	HUNT4 study	Community	rural	19+	19+	29,556	24,585	HbA1c	Yes		Lab	
693	Oman	1991	The 1991 National Diabetes Survey of Oman	National	both	20+	20+	2,809	1,989	Fasting glucose	Yes	Lab		
694	Oman	2000	Oman National Health Survey	National	both	20+	20+	2,933	2,905	Fasting glucose	No	Lab		
695	Oman	2001	Nizwa Healthy Lifestyle Project	Community	urban	20+	20+	692	600	Fasting glucose	Yes	Lab		
696	Oman	2008	Gulf Cooperation Council World Health Survey	National	both	18+	18+	2,264	2,446	Fasting glucose	Yes	Unknown		
697	Oman	2017	STEPS	National	both	18+	18+	2,997	3,365	Fasting glucose	Yes	Portable		
698	Pakistan	1994	Basit et al., J Pak Med Assoc 52:357-60, 2002	Subnational	rural	25+	25+	1,362	761	Fasting glucose	No	Unknown		
699	Pakistan	2002	Basit et al., Diabetes Res Clin Pract 94:456-62, 2011; Study 1	Subnational	rural	25+	25+	1,362	670	Fasting glucose	No	Lab		
700	Pakistan	2005	COBRA-1	Community	urban	40+	40+	1,506	1,376	Fasting glucose	No	Unknown		
701	Pakistan	2009-2010	Basit et al., Diabetes Res Clin Pract 94:456-62, 2011; Study 2	Subnational	rural	25+	25+	840	272	Fasting glucose	No	Lab		
702	Pakistan	2016-2017	National Diabetes Survey of Pakistan	National	both	20+	20+	4,261	3,273	Fasting glucose, HbA1c	No	Lab	Lab	18
703	Palau	2011-2013	STEPS	National	both	25-64	25-64	1,124	1,038	Fasting glucose	Yes	Portable		
704	Palau	2016	STEPS	National	both	18+	18+	854	872	Fasting glucose	Yes	Portable		
705	Panama	2010-2011	Prevalencia de factores de riesgo asociados a enfermedad cardiovascular 2010-2011	Subnational	both	18+	18+	2,472	1,072	Fasting glucose, HbA1c	Yes	Lab	Lab	
706	Panama	2019	Encuesta Nacional de Salud de Panama (ENSPA)	National	both	18+	18+	12,013	4,846	Fasting glucose, HbA1c	Yes	Lab	Lab	
707	Papua New Guinea	1991	Dowse et al., Med J Aust 160:767-74, 1994	Subnational	both	25-88	25-88	1,009	837	Fasting glucose	Yes	Lab		
708	Peru	2004	Factores de Riesgo de Enfermedades No Transmisibles	Community	urban	18+	18+	430	209	Fasting glucose	Yes	Lab		
709	Peru	2005	PREVENCION Study; Medina-Lezama et al., J Am Soc Hypertens 1:216-25, 2007	Community	urban	20-80	20-80	1,011	867	Fasting glucose	No	Unknown		
710	Peru	2004-2005	CArdiovascular Risk factors Multiple Evaluation in Latin America (CARMELA)	Community	urban	25-64	25-64	883	769	Fasting glucose	Yes	Lab		

	Country	Data years	Survey/Study name/Citation	Level of representative-ness	Rural, urban, or both	Age range as used for global analysis		Sample size used for global analysis		Glycaemic markers available	Information available on diabetes treatment	Whether measured in a laboratory or by a point-of-care portable device		Notes
						Female	Male	Female	Male			Glucose	HbA1c	
711	Peru	2004-2005	Encuesta Nacional de Indicadores Nutricionales, Bioquímicos, Socioeconómicos y Culturales Relacionados con las Enfermedades Crónicas Degenerativas	National	both	20+	20+	2,095	2,095	Fasting glucose	Yes	Lab		
712	Peru	2005	Factores de Riesgo de Enfermedades No Transmisibles	Community	urban	18+	18+	532	199	Fasting glucose	Yes	Lab		
713	Peru	2006	Factores de Riesgo de Enfermedades No Transmisibles	Community	urban	18+	18+	1,030	608	Fasting glucose	Yes	Lab		
714	Peru	2007-2008	PERU MIGRANT Study	Community	both	30+	30+	521	466	Fasting glucose, HbA1c	Yes	Lab	Lab	
715	Peru	2009-2012	CRONICAS Cohort Study	Subnational	both	35+	35+	1,849	1,737	Fasting glucose, HbA1c	Yes	Lab	Lab	
716	Peru	2013	Clinical functional and sociofamilial profiles of the elderly from a community in a district of Lima, Peru	Community	urban	60+	60+	200	114	Fasting glucose	No	Lab		
717	Peru	2013-2014	CRONICAS Cohort Study	Subnational	both	36+	36+	1,387	1,309	Fasting glucose, HbA1c	Yes	Lab	Lab	
718	Peru	2015-2016	PERU MIGRANT Study	Community	both	38+	38+	437	344	Fasting glucose	Yes	Lab		
719	Peru	2016-2017	Screening of T2DM	Community	urban	30-70	30-70	804	792	Fasting glucose, HbA1c	Yes	Lab	Lab	
720	Peru	2017-2018	Vigilancia Alimentario Nutricional por Etapas de Vida (VIANEV) 2017-2018	National	both	18-59	18-59	618	463	Fasting glucose	Yes	Portable		
721	Philippines	1998	National Nutrition Survey; Tanchoco et al., Asia Pac J Clin Nutr 12:271-6, 2003	National	both	20+	20+	927	1,030	Fasting glucose	No	Unknown		
722	Philippines	2003	6th National Nutrition Survey	National	both	20+	20+	2,497	2,255	Fasting glucose	Yes	Lab		
723	Philippines	2005	Cebu Longitudinal Health and Nutrition Survey 2005 Child Follow-up	Community	both	20-22	20-22	764	927	Fasting glucose	No	Unknown		
724	Philippines	2005	Cebu Longitudinal Health and Nutrition Survey 2005 Mother Follow-up	Community	both	35-69		1,872		Fasting glucose	No	Unknown		
725	Philippines	2008	7th National Nutrition Survey	National	both	20+	20+	3,719	3,318	Fasting glucose	Yes	Lab		
726	Philippines	2008	Philippines LIFECARE Cohort	National	both	20-50	20-50	1,743	1,329	Fasting glucose	No	Lab		
727	Philippines	2013-2014	8th National Nutrition Survey	National	both	18+	18+	10,427	9,797	Fasting glucose	Yes	Lab		
728	Philippines	2018-2021	Philippine Expanded National Nutrition Survey	National	both	18+	18+	42,650	34,265	Fasting glucose	No	Lab		
729	Poland	1989-1990	Polish Program CINDI (CINDI Lodz 1989-1990)	Community	urban	25-64	25-64	945	812	Fasting glucose	Yes	Lab		
730	Poland	1993	DECODE; DECODE Study Group, Diabetes Care 26:61-9, 2003	Community	urban	40-79	40-79	192	172	Fasting glucose	No	Unknown		
731	Poland	2000	The health status, risk factors of chronic diseases and health behaviors of residents of Torun (CINDI Torun 2000)	Community	urban	18-83	18-83	1,010	929	Fasting glucose	Yes	Lab		
732	Poland	2001-2002	The health status, risk factors of chronic diseases and health behaviors of residents of Lodz (CINDI Lodz 2001)	Community	urban	18-64	18-64	841	1,003	Fasting glucose	Yes	Lab		
733	Poland	2002	The health status, risk factors of chronic diseases and health behaviors of residents of Lodz - seniors (CINDI Lodz 2002)	Community	urban	65+	65+	539	291	Fasting glucose	Yes	Lab		
734	Poland	2002	NATPOL	National	both	18+	18+	1,294	1,014	Fasting glucose	Yes	Lab		
735	Poland	2001-2002	Young Men Cardiovascular Association Study	Community	urban		18+		944	Fasting glucose	Yes	Lab		19
736	Poland	2003	The European Male Ageing Study	Community	both		40+		394	Fasting glucose	Yes	Lab		
737	Poland	2002-2005	Health, Alcohol and Psychosocial Factors In Eastern Europe	Community	urban	45-70	45-70	5,484	5,214	Fasting glucose, HbA1c	Yes	Lab	Lab	
738	Poland	2003-2005	National Multicenter Health Survey in Poland. Project WOBASZ	National	both	20-74	20-74	6,960	6,310	Fasting glucose	Yes	Lab		
739	Poland	2006	The health, risk factors for chronic diseases, attitudes and behaviors of health residents of Torun (CINDI Torun 2006)	Community	urban	18-65	18-65	1,115	750	Fasting glucose	Yes	Lab		
740	Poland	2008	The European Male Ageing Study	Community	both		45+		308	Fasting glucose	Yes	Lab		
741	Poland	2007-2011	Medical, psychological and socioeconomic aspects of aging in Poland	National	both	55+	55+	2,671	2,775	Fasting glucose	Yes	Lab		
742	Poland	2010	Zatonska et al., Ann Agric Environ Med 18:265-9, 2011	Community	both	45-64	45-64	2,570	1,289	Fasting glucose	No	Lab		
743	Poland	2011	NATPOL	National	both	18-79	18-79	1,210	1,145	Fasting glucose, HbA1c	Yes	Lab	Lab	
744	Poland	2011-2014	Mogielica Human Ecology Study Site	Community	rural	45+	45+	405	142	Fasting glucose	Yes	Lab		
745	Poland	2013-2014	National Multicenter Health Survey in Poland. Project WOBASZ II	National	both	20+	20+	3,360	2,747	Fasting glucose	Yes	Lab		
746	Poland	2015-2016	LIPIDOGRAM2015 & LIPIDOGEN2015 Study - National epidemiological study of lipid disorders and selected risk factors of cardiovascular disease in primary health care in Poland	National	both	18+	18+	8,690	5,034	Fasting glucose, HbA1c	Yes	Portable	Lab	
747	Portugal	1999-2003	EPIPorto study	Community	urban	18+	18+	1,539	946	Fasting glucose	Yes	Lab		
748	Portugal	2011-2013	EPITeen - Epidemiological Health Investigation of Teenagers in Porto	Community	urban	20-23	20-23	868	810	Fasting glucose, HbA1c	No	Lab	Unknown	
749	Portugal	2015	Inquérito Nacional de Saúde com Exame Físico (INSEF)	National	both	25-74	25-74	2,615	2,265	HbA1c	Yes		Lab	
750	Puerto Rico	2006	Pérez et al., Ethn Dis 18:434-41, 2008	Community	urban	25-84	25-84	532	274	Fasting glucose	No	Unknown		
751	Qatar	2012	STEPS	National	both	18-64	18-64	1,379	1,053	Fasting glucose	Yes	Portable		
752	Romania	1997	Valorile medii si limitele normalitatii unor constante biologice	National	both	30-85	30-85	5,050	3,964	Fasting glucose	No	Unknown		
753	Romania	2011-2012	SEPHAR II (Study for the Evaluation of Prevalence of Hypertension and Cardiovascular Risk in Romania - 2nd edition)	National	both	18-80	18-80	1,038	936	Fasting glucose, HbA1c	Yes	Lab	Lab	
754	Romania	2012-2014	PREDATORR	National	both	20-79	20-79	1,431	1,285	Fasting glucose, HbA1c	Yes	Lab	Lab	
755	Romania	2021	SEPHAR IV (Study for the Evaluation of Prevalence of Hypertension and Cardiovascular Risk in Romania - 4th edition)	National	both	18-80	18-80	884	588	Fasting glucose, HbA1c	Yes	Lab	Lab	
756	Russian Federation	2002-2005	Health, Alcohol and Psychosocial Factors In Eastern Europe	Community	urban	45-70	45-70	5,079	4,244	Fasting glucose, HbA1c	Yes	Lab	Lab	
757	Russian Federation	2012-2014	Epidemiology of Cardiovascular dieseases in different regions of Russia (ESSE-RF)	National	both	25-64	25-64	11,639	6,979	Fasting glucose	Yes	Lab		
758	Russian Federation	2015-2017	Ural Eye and Medical Study (UEMS)	Subnational	rural	40+	40+	1,869	1,524	Fasting glucose	Yes	Lab		
759	Russian Federation	2015-2017	Ural Eye and Medical Study (UEMS)	Community	urban	40+	40+	1,437	1,035	Fasting glucose	Yes	Lab		
760	Russian Federation	2017	Epidemiology of Cardiovascular Diseases in Different Regions of Russia - 2 (ESSE-RF-2)	Subnational	both	25-64	25-64	3,699	2,980	Fasting glucose	Yes	Lab		
761	Russian Federation	2017-2020	Ural Very Old Study	Community	both	85+	85+	635	231	Fasting glucose	Yes	Lab		
762	Rwanda	2012	STEPS	National	both	18-64	18-64	4,066	2,524	Fasting glucose	Yes	Portable		
763	Rwanda	2021-2022	STEPS	National	both	18-69	18-69	3,389	2,130	Fasting glucose	Yes	Portable		
764	Saint Lucia	2012	STEPS	National	both	25-64	25-64	1,126	693	Fasting glucose	Yes	Portable		
765	Saint Lucia	2019-2020	STEPS	National	both	18-69	18-69	1,651	1,296	Fasting glucose	Yes	Portable		
766	Saint Vincent and the Grenadines	2013-2014	STEPS	National	both	18-69	18-69	1,923	1,544	Fasting glucose	Yes	Portable		
767	Samoa	1991	Non-Communicable Disease Risk Factor (NCDRF)	Subnational	rural	25+	25+	495	466	Fasting glucose	Yes	Lab		
768	Samoa	1991	Non-Communicable Disease Risk Factor (NCDRF)	Subnational	urban	25+	25+	443	328	Fasting glucose	Yes	Lab		

	Country	Data years	Survey/Study name/Citation	Level of representative-ness	Rural, urban, or both	Age range as used for global analysis		Sample size used for global analysis		Glycaemic markers available	Information available on diabetes treatment	Whether measured in a laboratory or by a point-of-care portable device		Notes
						Female	Male	Female	Male			Glucose	HbA1c	
769	Samoa	1995	McGarvey, Pac Health Dialog 8(1):157-62, 2001	National	both	29+	29+	153	145	Fasting glucose	Yes	Lab		
770	Samoa	2002	STEPS	National	both	25-64	25-64	1,339	1,188	Fasting glucose	Yes	Portable		
771	Samoa	2010	Samoan Genome-Wide Association Study	National	both	24-65	24-65	2,056	1,404	Fasting glucose	Yes	Lab		20
772	Sao Tome and Principe	2019	STEPS	National	both	18-69	18-69	1,367	979	Fasting glucose	Yes	Portable		
773	Saudi Arabia	1998	National Epidemiological Health Survey; Al-Nozha et al., Saudi Med J 25:1603-10, 2004	National	both	30-70	30-70	8,804	8,002	Fasting glucose	No	Unknown		
774	Saudi Arabia	2004-2005	STEPS	National	both	15-64	18-64	2,231	2,155	Fasting glucose	No	Lab		3
775	Saudi Arabia	2009	RIYADH Cohort 2; Al-Daghri et al., BMC Med 9:76, 2011	Community	urban	18-80	18-80	1,267	1,409	Fasting glucose	No	Lab		
776	Saudi Arabia	2011-2012	Jeeluna Study- National Assessment of the Health Needs of Adolescents in Saudi Arabia	National	both	18-19	18-19	256	430	Fasting glucose	No	Lab		
777	Senegal	2010-2012	Biocultural determinants of overweight and obesity in the context of nutrition transition in Senegal: a holistic anthropological approach	Subnational	both	18+	18+	242	216	Fasting glucose	Yes	Portable		
778	Senegal	2015	Les maladies chroniques au Sénégal: Une écologie de la santé comparative entre Dakar et Widou Thiengoly	Community	both	20+	20+	765	732	Fasting glucose	Yes	Portable		
779	Senegal	2015	Enquête Nationale sur les Facteurs de Risque des Maladies Non Transmissibles (STEPS)	National	both	18-70	18-70	3,336	1,923	Fasting glucose	Yes	Portable		
780	Seychelles	1989	Seychelles Heart Survey I	National	both	25-64	25-64	568	513	Fasting glucose	Yes	Portable		
781	Seychelles	2004	Seychelles Heart Survey III	National	both	25-64	25-64	687	568	Fasting glucose	Yes	Portable		
782	Seychelles	2013-2014	Seychelles Heart Survey IV	National	both	25-64	25-64	699	531	Fasting glucose, HbA1c	Yes	Portable	Lab	
783	Seychelles	2023	Seychelles Heart Survey V	National	both	18-74	18-74	654	531	Fasting glucose, HbA1c	Yes	Lab	Lab	2
784	Singapore	1982-1985	Thyroid Heart Study	National	both	18+	18+	900	959	Fasting glucose	Yes	Lab		
785	Singapore	1992	National Health Survey	National	both	18-64	18-64	1,703	1,744	Fasting glucose	No	Lab		
786	Singapore	1993-1995	NUH Heart Study	National	both	26+	26+	484	495	Fasting glucose	Yes	Lab		
787	Singapore	1998	National Health Survey	National	both	18-69	18-69	2,262	2,280	Fasting glucose	No	Lab		
788	Singapore	2004	National Health Survey	National	both	18-74	18-74	1,842	1,796	Fasting glucose	No	Unknown		
789	Singapore	2003-2005	Singapore Longitudinal Ageing Study - Cohort 1; SLAS-1	Community	both	55+	55+	1,766	1,031	Fasting glucose	Yes	Lab		
790	Singapore	2004-2007	Singapore Cardiovascular Cohort Study and Singapore Prospective Study Program	National	both	21+	21+	4,489	3,868	Fasting glucose, HbA1c	Yes	Lab	Lab	21
791	Singapore	2009-2011	The Singapore Chinese Eye Study	Community	both	40-80	40-80	1,597	1,590	HbA1c	Yes		Lab	
792	Singapore	2008-2013	Singapore Longitudinal Ageing Study - Cohort 2; SLAS-2	Community	both	55+	55+	2,000	1,216	Fasting glucose	Yes	Lab		
793	Singapore	2012-2013	Singapore Health Study	National	both	18-79	18-79	1,192	1,128	Fasting glucose, HbA1c	Yes	Lab	Lab	22
794	Singapore	2014-2015	Singapore Health 2	National	both	18-79	18-79	1,476	1,203	Fasting glucose, HbA1c	Yes	Lab	Lab	22
795	Singapore	2015-2017	The Singapore Chinese Eye Study	Community	both	50+	50+	1,193	1,169	HbA1c	Yes		Lab	
796	Slovakia	1993	Countrywide Integrated Noncommunicable Diseases Intervention Programme	National	both	18-64	18-64	1,177	719	Fasting glucose	No	Lab		
797	Slovakia	1998	Countrywide Integrated Noncommunicable Diseases Intervention Programme	National	both	18-64	18-64	1,045	855	Fasting glucose	No	Lab		
798	Slovakia	2003	Countrywide Integrated Noncommunicable Diseases Intervention Programme	National	both	18-64	18-64	866	620	Fasting glucose	No	Lab		
799	Slovakia	2008	Countrywide Integrated Noncommunicable Diseases Intervention Programme	National	both	18-64	18-64	561	390	Fasting glucose	No	Lab		
800	Slovakia	2011-2012	European Health Examination Survey	National	both	18-64	18-64	1,091	893	Fasting glucose	Yes	Lab		
801	Solomon Islands	2004	A genetic-ecological study of the risk factors for lifestyle-related diseases in Oceanian populations	Community	rural	18+	18+	107	106	Fasting glucose	Yes	Lab		
802	Solomon Islands	2004	A genetic-ecological study of the risk factors for lifestyle-related diseases in Oceanian populations	Community	urban	18+	18+	95	91	Fasting glucose	Yes	Lab		
803	Solomon Islands	2006	STEPS	Subnational	both	18-64	18-64	1,368	1,159	Fasting glucose	Yes	Portable		
804	Solomon Islands	2009-2010	Furusawa et al., N Z Med J 124(1333):17-28, 2011	Community	rural	18+	18+	215	153	HbA1c	Yes		Portable	
805	Solomon Islands	2009-2010	Furusawa et al., N Z Med J 124(1333):17-28, 2011	Community	urban	18-70	18-70	70	38	HbA1c	Yes		Portable	
806	Solomon Islands	2015	STEPS	National	both	18-69	18-69	1,318	1,087	Fasting glucose	Yes	Portable		
807	Solomon Islands	2017-2018	Impact of sea-level rise and relocation projects on health, ecology, and society in Oceania	Community	both	18+	18+	261	117	HbA1c	Yes		Portable	
808	Somalia	2016	The prevalence of selected risk factors for non-communicable diseases in Hargeisa, Somaliland: a cross-sectional study	Community	urban	20-69	20-69	955	145	Fasting glucose, HbA1c	Yes	Lab	Lab	
809	South Africa	2008-2009	Cape Town Bellville South Cohort Study - Baseline evaluation I	Community	urban	35-65	35-65	494	140	Fasting glucose, HbA1c	Yes	Lab	Lab	
810	South Africa	2011-2012	South Africa National Health and Nutrition Examination Survey	National	both	18+	18+	8,081	5,576	HbA1c	Yes		Lab	
811	South Africa	2018-2020	Vukuzazi Study	Community	both	18+	18+	10,976	4,874	HbA1c	Yes		Lab	
812	South Korea	2001	Kim et al., Br J Psychiatry 185:102-7, 2004	Community	both	65+	65+	432	300	Fasting glucose	No	Unknown		
813	South Korea	2001	Korea National Health and Nutrition Examination Survey	National	both	18+	18+	3,366	2,647	Fasting glucose	Yes	Lab		
814	South Korea	2005	Korea National Health and Nutrition Examination Survey	National	both	18+	18+	3,101	2,313	Fasting glucose	Yes	Lab		
815	South Korea	2007	Korea National Health and Nutrition Examination Survey	National	both	18+	18+	1,734	1,257	Fasting glucose	Yes	Lab		
816	South Korea	2008	Korea National Health and Nutrition Examination Survey	National	both	18+	18+	3,967	2,895	Fasting glucose	Yes	Lab		
817	South Korea	2009	Korea National Health and Nutrition Examination Survey	National	both	18+	18+	4,264	3,314	Fasting glucose	Yes	Lab		
818	South Korea	2008-2009	Korean National Health Insurance	National	both	40+	40+	6,087,857	5,763,109	Fasting glucose	Yes	Lab		
819	South Korea	2010	Korea National Health and Nutrition Examination Survey	National	both	18+	18+	3,543	2,740	Fasting glucose	Yes	Lab		
820	South Korea	2011	Korea National Health and Nutrition Examination Survey	National	both	18+	18+	3,479	2,598	Fasting glucose, HbA1c	Yes	Lab	Lab	
821	South Korea	2010-2011	Korean National Health Insurance	National	both	40+	40+	7,126,632	6,670,305	Fasting glucose	Yes	Lab		
822	South Korea	2012	Korea National Health and Nutrition Examination Survey	National	both	18+	18+	3,300	2,344	Fasting glucose, HbA1c	Yes	Lab	Lab	
823	South Korea	2013	Korea National Health and Nutrition Examination Survey	National	both	18+	18+	3,115	2,303	Fasting glucose, HbA1c	Yes	Lab	Lab	
824	South Korea	2012-2013	Korean National Health Insurance	National	both	40+	40+	7,782,567	7,256,160	Fasting glucose	Yes	Lab		
825	South Korea	2014	Korea National Health and Nutrition Examination Survey	National	both	18+	18+	2,943	2,115	Fasting glucose, HbA1c	Yes	Lab	Lab	
826	South Korea	2015	Korea National Health and Nutrition Examination Survey	National	both	18+	18+	2,963	2,308	Fasting glucose, HbA1c	Yes	Lab	Lab	
827	South Korea	2014-2015	Korean National Health Insurance	National	both	40+	40+	8,356,468	7,869,529	Fasting glucose	Yes	Lab		
828	South Korea	2016	Korea National Health and Nutrition Examination Survey	National	both	18+	18+	3,474	2,682	Fasting glucose, HbA1c	Yes	Lab	Lab	
829	South Korea	2017	Korea National Health and Nutrition Examination Survey	National	both	18+	18+	3,438	2,780	Fasting glucose, HbA1c	Yes	Lab	Lab	
830	South Korea	2017	Korean National Health Insurance	National	both	40+	40+	9,074,778	8,535,547	Fasting glucose	Yes	Lab		
831	South Korea	2018	Korea National Health and Nutrition Examination Survey	National	both	18+	18+	3,522	2,763	Fasting glucose, HbA1c	Yes	Lab	Lab	
832	South Korea	2019	Korea National Health and Nutrition Examination Survey	National	both	18+	18+	3,504	2,835	Fasting glucose, HbA1c	Yes	Lab	Lab	
833	South Korea	2019	Korean National Health Insurance	National	both	40+	40+	9,809,734	9,238,805	Fasting glucose	Yes	Lab		

	Country	Data years	Survey/Study name/Citation	Level of representative-ness	Rural, urban, or both	Age range as used for global analysis		Sample size used for global analysis		Glycaemic markers available	Information available on diabetes treatment	Whether measured in a laboratory or by a point-of-care portable device		Notes
						Female	Male	Female	Male			Glucose	HbA1c	
834	South Korea	2020	Korea National Health and Nutrition Examination Survey	National	both	18+	18+	3,283	2,685	Fasting glucose, HbA1c	Yes	Lab	Lab	
835	South Korea	2021	Korea National Health and Nutrition Examination Survey	National	both	18+	18+	3,203	2,510	Fasting glucose, HbA1c	Yes	Lab	Lab	
836	South Korea	2021	Korean National Health Insurance	National	both	40+	40+	9,735,163	9,234,806	Fasting glucose	Yes	Lab		
837	South Korea	2023	Korean National Health Insurance	National	both	40+	40+	10,590,978	10,102,790	Fasting glucose	Yes	Lab		2
838	Spain	1989	Cardiovascular Risk Factors Study in Catalonia	Subnational	both	18+	18+	115	109	Fasting glucose	No	Lab		
839	Spain	1994	DECODE; DECODE Study Group, Diabetes Care 26:61-9, 2003	Community	urban	30-89	30-89	2,699	2,108	Fasting glucose	No	Unknown		
840	Spain	1996	REGICOR Study; Masia et al., Rev Esp Cardiol 57:261-4, 2004	Subnational	both	25-74	25-74	874	874	Fasting glucose	No	Unknown		
841	Spain	1999	The Asturias Study; Botas, et al., Diabet Med 20:904-8, 2003	Subnational	both	30-79	30-79	542	445	Fasting glucose	No	Unknown		
842	Spain	1999-2000	Factores de riesgo en las islas Baleares: Estudio CORSAIB	Subnational	both	35-74	35-74	865	804	Fasting glucose	Yes	Lab		
843	Spain	2001-2002	Catalan Health Interview Survey	Subnational	both	18-74	18-74	755	599	Fasting glucose	Yes	Portable		
844	Spain	2001-2003	DIabetes, Nutrición y Obesidad en la población adulta de la Región de Murcia (DINO)	Subnational	both	20+	20+	837	719	Fasting glucose, HbA1c	Yes	Lab	Lab	
845	Spain	2000-2005	CDC of the Canary Islands	Subnational	both	18-75	18-75	3,763	2,908	Fasting glucose	Yes	Lab		
846	Spain	2003	The European Male Ageing Study	Community	both		40+		402	Fasting glucose	Yes	Lab		
847	Spain	2004	Vioque J et al., Obesity 16(3):664-70, 2008	Community	urban	24+	24+	117	87	Fasting glucose, HbA1c	Yes	Portable	Portable	
848	Spain	2004	Cardiovascular Risk Study in Castilla y León (RECCyL)	Subnational	both	18+	18+	2,003	1,816	Fasting glucose	Yes	Lab		
849	Spain	2003-2005	Registre Gironi del Cor (REGICOR)	Subnational	both	35-79	35-79	3,200	2,883	Fasting glucose	Yes	Lab		
850	Spain	2004-2006	PREVICTUS	National	both	60+	60+	3,905	3,405	Fasting glucose	Yes	Lab		
851	Spain	2006	Lopez Suarez et al., Rev Esp Cardiol 61:1150-8, 2008	Community	urban	50-75	50-75	460	398	Fasting glucose	No	Unknown		
852	Spain	2008	The European Male Ageing Study	Community	both		45+		289	Fasting glucose	Yes	Lab		
853	Spain	2007-2009	Harmonizing Equation of Risk in Mediterraneon countries EXtremadura (HERMEX)	Subnational	both	25-79	25-79	1,498	1,298	Fasting glucose, HbA1c	Yes	Lab	Lab	
854	Spain	2008-2010	Study on Nutrition and Cardiovascular Risk in Spain	National	both	18+	18+	6,803	6,123	Fasting glucose, HbA1c	Yes	Lab	Lab	
855	Spain	2009	Cardiovascular Risk Study in Castilla y León (RECCyL)	Subnational	both	20+	20+	1,579	1,299	Fasting glucose	Yes	Lab		
856	Spain	2014	Cardiovascular Risk Study in Castilla y León (RECCyL)	Subnational	both	20+	20+	1,495	1,226	Fasting glucose	Yes	Lab		
857	Spain	2015	Study on Nutrition and Cardiovascular Risk in Spain (ENRICA)	National	both	65+	65+	952	871	Fasting glucose	Yes	Lab		
858	Spain	2016-2017	Estudio de Nutrición y Riesgo Cardiovascular en España (ENRICA)-Seniors cohort	Subnational	urban	65-94	65-94	1,739	1,532	Fasting glucose, HbA1c	Yes	Lab	Lab	
859	Spain	2019	Estudio de Nutrición y Riesgo Cardiovascular en España (ENRICA)-Seniors cohort	Subnational	urban	65-95	65-95	968	926	Fasting glucose, HbA1c	Yes	Lab	Lab	
860	Sri Lanka	2000	Malavige et al., Diabetes Res Clin Pract 57:143-5, 2002	Community	urban	30-64	30-64	621	421	Fasting glucose	No	Unknown		
861	Sri Lanka	2005-2006	Sri Lanka Diabetes, Cardiovascular study (SLDCS)	National	both	18+	18+	2,717	1,777	Fasting glucose	Yes	Lab		
862	Sri Lanka	2007	Pinidiyapathirage et al., Diabet Med 30:326-32, 2013	Community	urban	35-64	35-64	1,636	1,349	Fasting glucose, HbA1c	No	Lab	Lab	
863	Sri Lanka	2010	Pubudu De Silva et al., Int J Equity Health 11:76, 2012	Subnational	both	35-64	35-64	606	628	Fasting glucose	No	Lab		
864	Sri Lanka	2014	STEPS	National	both	18-69	18-69	3,082	2,017	Fasting glucose	Yes	Portable		
865	Sri Lanka	2018-2019	The Sri Lanka Health and Ageing Study (SLHAS)	National	both	18+	18+	3,239	3,109	Fasting glucose, HbA1c	Yes	Lab	Lab	
866	Sri Lanka	2021	STEPS	National	both	18-69	18-69	3,767	2,447	Fasting glucose	Yes	Portable		
867	State of Palestine	1996-1998	Ramallah study	Community	rural	18-64	18-64	443	206	Fasting glucose	Yes	Portable		
868	State of Palestine	1996-1998	Ramallah study	Community	urban	18-64	18-64	458	182	Fasting glucose	Yes	Portable		
869	State of Palestine	2010	STEPS	National	both	18-64	18-64	3,834	2,330	Fasting glucose	Yes	Unknown		
870	State of Palestine	2022	STEPS	National	both	18-69	18-69	3,661	1,701	Fasting glucose	Yes	Portable		
871	Sudan	2005-2006	STEPS	Subnational	both	25-64	25-64	321	145	Fasting glucose	No	Unknown		
872	Sudan	2016	STEPS	National	both	18-69	18-69	4,594	2,707	Fasting glucose	Yes	Portable		
873	Suriname	2013-2015	The Healthy Life in Suriname Study (HELISUR)	Subnational	urban	18-70	18-70	722	422	Fasting glucose, HbA1c	Yes	Lab	Lab	
874	Sweden	1980-1981	Population Study of Women in Gothenburg	Community	urban	50-72		1,153		Fasting glucose	Yes	Lab		
875	Sweden	1980-1985	Uppsala Longitudinal Study of Adult Men	Community	both		55-64		1,814	Fasting glucose	No	Lab		23
876	Sweden	1991	Asplund-Carlson et al., J Intern Me 236:57-64, 1994	Subnational	both		40-50		1,564	Fasting glucose	No	Unknown		
877	Sweden	1992-1993	Population Study of Women in Gothenburg	Community	urban	62-84		834		Fasting glucose	Yes	Lab		
878	Sweden	1991-1995	Uppsala Longitudinal Study of Adult Men	Community	both		69-74		1,151	Fasting glucose	Yes	Lab		23
879	Sweden	1994	DECODE; DECODE Study Group, Diabetes Care 26:61-9, 2003	Community	urban	30-79	30-79	1,120	1,058	Fasting glucose	No	Unknown		
880	Sweden	1994	Nilson et al., Scand J Prim Health Care 18(2):111-112, 2000	Community	urban	56-65	56-65	217	170	Fasting glucose	No	Lab		
881	Sweden	1995	MONICA Gothenburg	Community	urban	25-64	25-64	865	745	Fasting glucose	Yes	Unknown		
882	Sweden	1997	SWESTONIA; Johansson et al., J Intern Med 252:551-60, 2002	Community	urban	35-55	35-55	135	137	Fasting glucose	No	Unknown		
883	Sweden	1997-2001	Uppsala Longitudinal Study of Adult Men	Community	both		73-80		781	Fasting glucose	Yes	Lab		23
884	Sweden	2003	The European Male Ageing Study	Community	both		40+		404	Fasting glucose	Yes	Lab		
885	Sweden	2001-2004	Swedish INTERGENE Cohort Study	Subnational	both	24-76	24-76	1,907	1,694	Fasting glucose	Yes	Lab		
886	Sweden	2001-2004	PIVUS Study	Community	both	70	70	1,527	1,512	Fasting glucose	Yes	Portable		
887	Sweden	2004	Welin et al., BMC Public Health 8:403, 2008	Community	urban	50-61	50-61	667	1,250	Fasting glucose	No	Unknown		
888	Sweden	2002-2006	Malmö Preventive Project Re-examination Study Cohort; Leosdottir et al., Cardiovasc Diabetol 10:118, 2011	Community	both	57-86	57-86	6,680	11,546	Fasting glucose	No	Lab		
889	Sweden	2003-2005	Uppsala Longitudinal Study of Adult Men	Community	both		80-83		476	Fasting glucose, HbA1c	Yes	Lab	Lab	
890	Sweden	2004-2005	European Youth Heart Study (EYHS) II	Subnational	urban	18-21	18-21	109	68	Fasting glucose	No	Lab		
891	Sweden	2004-2005	Population Study of Women in Gothenburg	Community	urban	38-50		500		Fasting glucose	Yes	Lab		
892	Sweden	2008	The European Male Ageing Study	Community	both		45+		382	Fasting glucose	Yes	Lab		
893	Sweden	2007-2009	PIVUS Study	Community	both	75	75	1,440	1,389	Fasting glucose	Yes	Portable		
894	Sweden	2008-2009	Uppsala Longitudinal Study of Adult Men	Community	both		84-88		275	Fasting glucose, HbA1c	Yes	Lab	Unknown	
895	Sweden	2011-2014	PIVUS Study	Community	both	80	80	1,275	1,161	Fasting glucose	Yes	Portable		
896	Sweden	2014-2016	Swedish INTERGENE Cohort Study	Subnational	urban	37-88	37-88	659	606	Fasting glucose	Yes	Lab		
897	Sweden	2016-2017	Population Study of Women in Gothenburg	Community	urban	38-50		573		Fasting glucose	Yes	Lab		
898	Switzerland	2005	Bus Santé Study	Subnational	urban	20-80	20-80	74	53	Fasting glucose	Yes	Lab		
899	Switzerland	2003-2006	Cohorte Lausannoise	Community	urban	35-75	35-75	3,454	3,104	Fasting glucose	Yes	Lab		

	Country	Data years	Survey/Study name/Citation	Level of representative-ness	Rural, urban, or both	Age range as used for global analysis		Sample size used for global analysis		Glycaemic markers available	Information available on diabetes treatment	Whether measured in a laboratory or by a point-of-care portable device		Notes
						Female	Male	Female	Male			Glucose	HbA1c	
900	Switzerland	2006	Bus Santé Study	Subnational	urban	20-80	20-80	53	67	Fasting glucose	Yes	Lab		
901	Switzerland	2007	Bus Santé Study	Subnational	urban	20-80	20-80	46	48	Fasting glucose	Yes	Lab		
902	Switzerland	2008	Bus Santé Study	Subnational	urban	20-80	20-80	209	215	Fasting glucose	Yes	Lab		
903	Switzerland	2009	Bus Santé Study	Subnational	urban	20-80	20-80	505	455	Fasting glucose	Yes	Lab		
904	Switzerland	2010	Bus Santé Study	Subnational	urban	20-80	20-80	483	467	Fasting glucose	Yes	Lab		
905	Switzerland	2011	Bus Santé Study	Subnational	urban	20-80	20-80	457	434	Fasting glucose	Yes	Lab		
906	Switzerland	2009-2012	Cohorte Lausannoise	Community	urban	40-75	40-75	29	2,337	Fasting glucose	Yes	Lab		
907	Switzerland	2012	Bus Santé Study	Subnational	urban	20-80	20-80	451	473	Fasting glucose	Yes	Lab		
908	Switzerland	2013	Bus Santé Study	Subnational	urban	20-80	20-80	514	461	Fasting glucose	Yes	Lab		
909	Switzerland	2014	Bus Santé Study	Subnational	urban	20-80	20-80	512	464	Fasting glucose	Yes	Lab		
910	Switzerland	2015	Bus Santé Study	Subnational	urban	20-80	20-80	499	501	Fasting glucose	Yes	Lab		
911	Switzerland	2016	Bus Santé Study	Subnational	urban	20-80	20-80	496	460	Fasting glucose	Yes	Lab		
912	Switzerland	2014-2017	Cohorte Lausannoise	Community	urban	45-87	45-87	2,398	1,938	Fasting glucose, HbA1c	Yes	Lab	Lab	
913	Switzerland	2017	Bus Santé Study	Subnational	urban	20-80	20-80	577	543	Fasting glucose	Yes	Lab		
914	Switzerland	2018	Bus Santé Study	Subnational	urban	20-80	20-80	545	513	Fasting glucose	Yes	Lab		
915	Switzerland	2019	Bus Santé Study	Subnational	urban	20-80	20-80	418	350	Fasting glucose	Yes	Lab		
916	Switzerland	2018-2021	Cohorte Lausannoise	Community	urban	49-90	49-90	1,866	1,510	Fasting glucose, HbA1c	Yes	Lab	Lab	
917	Syrian Arab Republic	2002	National Survey on non-communicable diseases and factors affecting their development	National	both	15-65	15-65	2,958	1,784	Fasting glucose	No	Unknown		3
918	Taiwan	1993-1996	Nutrition and Health Survey in Taiwan	National	both	18+	18+	2,608	2,598	Fasting glucose	Yes	Lab		
919	Taiwan	1996	Chen et al., Diabetes Res Clin Pract 44:59-69, 1999	Community	both	40-79	40-79	822	779	Fasting glucose	No	Unknown		
920	Taiwan	1997	Chen et al., Diabetes Res Clin Pract 51:59-66, 2001	Community	both	50-79	50-79	1,053	540	Fasting glucose	No	Unknown		
921	Taiwan	1998	Lai et al., J Gerontol A Biol Sci Med Sci 55:M257-9, 2000	Community	both	65-80	65-80	198	387	Fasting glucose	No	Unknown		
922	Taiwan	1999-2000	Nutrition and Health Survey in Taiwan	National	both	65+	65+	754	796	Fasting glucose	Yes	Lab		
923	Taiwan	2000	Social Environment and Biomarkers of Aging Study	National	both	50+	50+	432	590	Fasting glucose, HbA1c	Yes	Lab	Lab	
924	Taiwan	2002	Taiwanese Survey on Hypertension, Hyperglycemia and Hyperlipidemia	National	both	18+	18+	3,215	2,944	Fasting glucose, HbA1c	No	Lab	Lab	
925	Taiwan	2005	TCHS; Lin et al., Eur J Clin Invest 37:783-90, 2007	Community	urban	40+	40+	1,212	1,147	Fasting glucose	No	Unknown		
926	Taiwan	2006	Social Environment and Biomarkers of Aging Study	National	both	53+	53+	604	679	Fasting glucose, HbA1c	Yes	Lab	Lab	
927	Taiwan	2005-2008	Nutrition and Health Survey in Taiwan	National	both	19+	19+	1,363	1,327	Fasting glucose	Yes	Lab		
928	Taiwan	2007	Taiwanese Survey on Hypertension, Hyperglycemia and Hyperlipidemia	National	both	20+	20+	2,508	2,174	Fasting glucose, HbA1c	Yes	Lab	Lab	
929	Taiwan	2013-2016	Nutrition and Health Survey in Taiwan	National	both	18+	18+	2,861	2,863	Fasting glucose, HbA1c	Yes	Lab	Lab	
930	Taiwan	2017-2020	Nutrition and Health Survey in Taiwan	National	both	18+	18+	3,399	3,379	Fasting glucose, HbA1c	Yes	Lab	Lab	
931	Tajikistan	2016	STEPS	National	both	18-69	18-69	1,567	1,098	Fasting glucose	Yes	Portable		
932	Tanzania	1987	Swai et al., BMJ 305:1057-62, 1992	Community	rural	15+	18+	4,283	3,301	Fasting glucose	No	Lab		3
933	Tanzania	1997	Aspray et al., Trans R Soc Trop Med Hyg 94:637-44, 2000	Community	rural	18-64	18-64	527	401	Fasting glucose	No	Unknown		
934	Tanzania	1997	Aspray et al., Trans R Soc Trop Med Hyg 94:637-44, 2000	Community	urban	18-64	18-64	438	332	Fasting glucose	No	Unknown		
935	Tanzania	2011	STEPS	Subnational	both	25-64	25-64	1,524	1,011	Fasting glucose	Yes	Portable		
936	Tanzania	2012	STEPS	National	both	25-64	25-64	2,849	2,601	Fasting glucose	Yes	Portable		
937	Tanzania	2023	STEPS	National	both	18-69	18-69	1,945	1,459	Fasting glucose	Yes	Portable		2
938	Thailand	1983	Vannasaeng et al., J Med Assoc Thai 70 Suppl 2:126-30, 1987	Subnational	urban	20-79	20-79	681	442	Fasting glucose	No	Unknown		
939	Thailand	1991	Thailand National Health Examination Survey I	National	both	18+	18+	7,255	5,363	Fasting glucose	Yes	Lab		
940	Thailand	1997	Thailand National Health Examination Survey II	National	both	18-59	18-59	1,682	1,022	Fasting glucose	No	Lab		
941	Thailand	2000	InterASIA	National	both	35+	35+	3,212	2,093	Fasting glucose	Yes	Lab		
942	Thailand	2004	Thailand National Health Examination Survey III	National	both	18+	18+	19,942	18,500	Fasting glucose	Yes	Lab		
943	Thailand	2009	Thailand National Health Examination Survey IV	National	both	18+	18+	10,225	9,271	Fasting glucose	Yes	Lab		
944	Thailand	2014	Thailand National Health Examination Survey V	National	both	18+	18+	10,566	7,714	Fasting glucose	Yes	Lab		
945	Thailand	2019-2020	Thailand National Health Examination Survey VI	National	both	18+	18+	12,463	8,976	Fasting glucose, HbA1c	Yes	Lab	Lab	
946	Timor-Leste	2009-2010	Timor-Leste Eye Health Survey	Subnational	both	40+	40+	248	246	HbA1c	Yes		Lab	
947	Timor-Leste	2014	STEPS	National	both	18-69	18-69	1,465	1,080	Fasting glucose	Yes	Portable		
948	Togo	2010	STEPS	National	both	18-64	18-64	1,967	1,897	Fasting glucose	Yes	Portable		
949	Togo	2021-2022	STEPS	National	both	18-69	18-69	2,172	1,586	Fasting glucose	Yes	Portable		
950	Tokelau	2005	STEPS	National	both	18-64	18-64	267	241	Fasting glucose	Yes	Portable		
951	Tokelau	2014	STEPS	National	both	18-64	18-64	282	262	Fasting glucose	Yes	Portable		
952	Tonga	2004	STEPS	National	both	18-64	18-64	301	237	Fasting glucose	No	Portable		
953	Tonga	2011	STEPS	National	both	18-64	18-64	1,464	928	Fasting glucose	Yes	Portable		
954	Trinidad and Tobago	2011	STEPS	National	both	18-64	18-64	1,538	1,050	Fasting glucose	Yes	Portable		
955	Tunisia	1981	Papoz et al., Int J Epidemiol 17:419-22, 1988	Community	rural	20+	20+	893	618	Fasting glucose	No	Unknown		
956	Tunisia	1990	Gharbi et al., Rev Epidemiol Sante Publique 50:349-55, 2002	Community	both	35-50	35-50	345	345	Fasting glucose	No	Unknown		
957	Tunisia	1996-1997	Ariana Healthy Project 1997	Community	both	35-65	35-65	2,724	2,650	Fasting glucose	Yes	Lab		
958	Tunisia	1996-1997	Tunisian National Nutrition Survey 1996-1997	National	both	18+	18+	2,674	1,397	Fasting glucose	Yes	Lab		
959	Tunisia	2001	Romdhane et al., Tunis Med 83 Suppl 5:41-6, 2005	Community	both	40-69	40-69	1,092	744	Fasting glucose	No	Unknown		
960	Tunisia	2005	Tunisian National Survey 2005 (TAHINA)	National	both	35-71	35-71	4,436	3,314	Fasting glucose	Yes	Lab		
961	Tunisia	2009-2010	ObeMaghreb	Subnational	urban	18-49	18-49	696	998	Fasting glucose	Yes	Lab		
962	Tunisia	2016	Tunisian Health Examination Survey	Community	both	18+	18+	4,702	4,196	HbA1c	Yes		Portable	
963	Türkiye	1990	Turkish Adult Risk Factor Study	National	both	20+	20+	1,130	1,028	Fasting glucose	No	Lab		
964	Türkiye	1995	Turkish Adult Risk Factor Study	National	both	25+	25+	606	584	Fasting glucose	No	Lab		

	Country	Data years	Survey/Study name/Citation	Level of representative-ness	Rural, urban, or both	Age range as used for global analysis		Sample size used for global analysis		Glycaemic markers available	Information available on diabetes treatment	Whether measured in a laboratory or by a point-of-care portable device		Notes
						Female	Male	Female	Male			Glucose	HbA1c	
965	Türkiye	1998	Turkish Adult Risk Factor Study	National	both	28+	28+	781	718	Fasting glucose	Yes	Lab		
966	Türkiye	2000	Turkish Adult Risk Factor Study	National	both	30+	30+	952	904	Fasting glucose	Yes	Lab		
967	Türkiye	2001	Yumuk et al., Diabetes Res Clin Pract 70:151-8, 2005	Community	urban	20+	20+	1,789	688	Fasting glucose	No	Unknown		
968	Türkiye	2001	Sekuri et al., Jpn Heart J 45:119-31, 2004	Community	rural	45+		205		Fasting glucose	No	Unknown		
969	Türkiye	2000-2002	The Healthy Nutrition for Healthy Heart Study	National	both	30+	30+	10,657	4,778	Fasting glucose	No	Lab		
970	Türkiye	2002	Gokcel et al., Diabetes Care 26:3031-4, 2003	Subnational	both	20-79	20-79	1,030	607	Fasting glucose	No	Unknown		
971	Türkiye	2002	Soysal et al., Anadolu Kardiyol Derg 5:196-201, 2005	Subnational	urban	25-39	25-39	469	289	Fasting glucose	No	Unknown		
972	Türkiye	2001-2002	Turkish Adult Risk Factor Study	National	both	32+	32+	1,247	1,137	Fasting glucose	Yes	Lab		
973	Türkiye	2003-2005	Prevalence of prehypertension and associated risk factors among Turkish adults: Trabzon Hypertension Study	Subnational	both	20+	20+	2,601	2,208	Fasting glucose	No	Lab		
974	Türkiye	2005-2006	Turkish Adult Risk Factor Study	National	both	35+	35+	1,473	1,401	Fasting glucose	Yes	Lab		
975	Türkiye	2007-2009	Balcova Heart Study	Community	urban	30+	30+	8,441	4,187	Fasting glucose	Yes	Lab		
976	Türkiye	2007-2008	Turkish Adult Risk Factor Study	National	both	37+	37+	1,473	1,401	Fasting glucose	Yes	Lab		
977	Türkiye	2009-2010	Turkish Adult Risk Factor Study	National	both	39+	39+	1,474	1,403	Fasting glucose	Yes	Lab		
978	Türkiye	2010	TURDEP-II; Satman et al., Eur J Epidemiol 28:169-80, 2013	National	urban	20+	20+	9,943	5,840	Fasting glucose	No	Lab		
979	Türkiye	2010	TURDEP-II; Satman et al., Eur J Epidemiol 28:169-80, 2013	National	rural	20+	20+	6,578	3,863	Fasting glucose	No	Lab		
980	Türkiye	2009-2012	Prevalence of diabetes and associated risk factors among adult population in Trabzon city	Subnational	both	20+	20+	2,125	1,574	Fasting glucose	No	Lab		
981	Türkiye	2012-2013	Turkish Adult Risk Factor Study	National	both	37+	37+	1,115	1,028	Fasting glucose, HbA1c	Yes	Lab	Lab	
982	Türkiye	2014-2015	Turkish Adult Risk Factor Study	National	both	44+	44+	756	712	Fasting glucose	No	Lab		
983	Türkiye	2017	STEPS	National	both	18+	18+	3,493	2,369	Fasting glucose	Yes	Portable		
984	Turkmenistan	2013	STEPS	National	both	18-64	18-64	2,874	2,033	Fasting glucose	Yes	Portable		
985	Turkmenistan	2018	STEPS	National	both	18-69	18-69	2,244	1,716	Fasting glucose	Yes	Portable		
986	Tuvalu	2015	STEPS	National	both	18-69	18-69	616	525	Fasting glucose	Yes	Portable		
987	Uganda	2014	STEPS	National	both	18-69	18-69	2,132	1,570	Fasting glucose	Yes	Portable		
988	Uganda	2023	STEPS	National	both	18-69	18-69	2,069	1,426	Fasting glucose	Yes	Portable		2
989	Ukraine	2019	STEPS	National	both	18-69	18-69	2,662	1,610	Fasting glucose	Yes	Portable		
990	United Arab Emirates	1999-2000	Emirates National Diabetes and Coronary Artery Disease Risk Factor Study	National	both	20-80	20-80	3,773	2,839	Fasting glucose	No	Lab		
991	United Arab Emirates	2017-2018	STEPS	National	both	18+	18+	2,356	2,172	Fasting glucose	Yes	Portable		
992	United Kingdom	1984-1986	Scottish Heart Health Survey	Subnational	both	40-59	40-59	4,447	4,381	Fasting glucose	Yes	Lab		
993	United Kingdom	1987-1988	Edinburgh Artery Study	Community	urban	54-75	54-75	769	797	Fasting glucose	No	Lab		
994	United Kingdom	1993	DECODE; DECODE Study Group, Diabetes Care 26:61-9, 2003	Community	urban	30-79	30-79	384	415	Fasting glucose	No	Unknown		
995	United Kingdom	1993	Whickham Survey; Vanderpump et al., Diabet Med 13:741-7, 1996	Community	urban	35+	35+	938	761	Fasting glucose	No	Unknown		
996	United Kingdom	1998-2000	The British Regional Heart Study	National	urban		60-79		4,105	Fasting glucose	Yes	Lab		
997	United Kingdom	1999-2001	British Women's Heart and Health Study	National	both	60-79		3,909		Fasting glucose	Yes	Lab		24
998	United Kingdom	1999-2004	Hertfordshire Cohort Study	Subnational	both	59-73	59-73	1,418	1,579	Fasting glucose	Yes	Lab		
999	United Kingdom	2003	The European Male Ageing Study	Community	both		40+		389	Fasting glucose	Yes	Lab		
1000	United Kingdom	2003	Health Survey for England	National	both	18+	18+	7,823	6,408	Fasting glucose, HbA1c	Yes	Lab	Lab	
1001	United Kingdom	2003	Scottish Health Survey (SHeS)	Subnational	both	18+	18+	4,370	3,497	HbA1c	Yes		Lab	
1002	United Kingdom	2003-2005	Hertfordshire Ageing Study	Subnational	both	72-82	72-82	151	208	Fasting glucose, HbA1c	Yes	Lab	Lab	
1003	United Kingdom	2004-2005	English Longitudinal Study of Ageing Wave 2	National	both	52-80	52-80	3,715	3,153	Fasting glucose, HbA1c	Yes	Lab	Lab	
1004	United Kingdom	2005	Health Survey for England	National	both	65+	65+	2,372	1,897	HbA1c	Yes		Lab	
1005	United Kingdom	2006	Health Survey for England	National	both	18+	18+	6,561	5,444	HbA1c	Yes		Lab	
1006	United Kingdom	2006-2007	Newcastle 85+ Study	Community	urban	84+	84+	431	283	Fasting glucose, HbA1c	No	Lab	Lab	25
1007	United Kingdom	2008	The European Male Ageing Study	Community	both		45+		335	Fasting glucose	Yes	Lab		
1008	United Kingdom	2008	Health Survey for England	National	both	18+	18+	7,960	6,550	HbA1c	Yes		Lab	
1009	United Kingdom	2007-2009	Newcastle 85+ Study	Community	urban	85+	85+	311	187	HbA1c	No		Lab	25
1010	United Kingdom	2008	Scottish Health Survey (SHeS)	Subnational	both	18+	18+	3,475	2,772	HbA1c	Yes		Lab	
1011	United Kingdom	2008-2009	English Longitudinal Study of Ageing Wave 4	National	both	50+	50+	4,499	3,674	Fasting glucose, HbA1c	Yes	Lab	Lab	
1012	United Kingdom	2009	Health Survey for England	National	both	18+	18+	2,420	2,050	HbA1c	Yes		Lab	
1013	United Kingdom	2006-2010	MRC National Survey of Health and Development	National	both	60-65	60-65	1,068	969	Fasting glucose, HbA1c	Yes	Lab	Lab	
1014	United Kingdom	2009	Scottish Health Survey (SHeS)	Subnational	both	18+	18+	4,089	3,226	HbA1c	Yes		Lab	
1015	United Kingdom	2010	Health Survey for England	National	both	18+	18+	4,486	3,598	HbA1c	Yes		Lab	
1016	United Kingdom	2008-2012	National Diet and Nutrition Survey (NDNS)	National	both	18+	18+	914	674	Fasting glucose, HbA1c	No	Lab	Lab	
1017	United Kingdom	2009-2010	Newcastle 85+ Study	Community	urban	85+	85+	258	160	Fasting glucose, HbA1c	No	Lab	Lab	25
1018	United Kingdom	2010	Scottish Health Survey (SHeS)	Subnational	both	18+	18+	3,981	3,047	HbA1c	Yes		Lab	
1019	United Kingdom	2010	Understanding Society: the UK Household Longitudinal Study	National	both	18+	18+	4,709	3,716	HbA1c	No		Lab	26
1020	United Kingdom	2011	British Household Panel Survey	National	both	18+	18+	1,385	1,157	HbA1c	No		Lab	26
1021	United Kingdom	2010-2012	The British Regional Heart Study	National	urban		72-91		1,671	Fasting glucose, HbA1c	Yes	Lab	Unknown	
1022	United Kingdom	2011	Health Survey for England	National	both	18+	18+	4,584	3,725	HbA1c	Yes		Lab	
1023	United Kingdom	2011	Scottish Health Survey (SHeS)	Subnational	both	18+	18+	4,093	3,219	HbA1c	Yes		Lab	
1024	United Kingdom	2012	Health Survey for England	National	both	18+	18+	4,447	3,580	HbA1c	Yes		Lab	
1025	United Kingdom	2012-2013	English Longitudinal Study of Ageing Wave 6	National	both	50+	50+	4,874	3,945	Fasting glucose, HbA1c	Yes	Lab	Lab	
1026	United Kingdom	2013	Health Survey for England	National	both	18+	18+	4,653	3,815	HbA1c	Yes		Lab	
1027	United Kingdom	2014	Health Survey for England	National	both	18+	18+	4,308	3,485	HbA1c	Yes		Lab	
1028	United Kingdom	2013-2014	National Diet and Nutrition Survey (NDNS)	National	both	18+	18+	407	281	Fasting glucose, HbA1c	No	Lab	Lab	
1029	United Kingdom	2015	Health Survey for England	National	both	18+	18+	4,290	3,503	HbA1c	Yes		Lab	

	Country	Data years	Survey/Study name/Citation	Level of representative-ness	Rural, urban, or both	Age range as used for global analysis		Sample size used for global analysis		Glycaemic markers available	Information available on diabetes treatment	Whether measured in a laboratory or by a point-of-care portable device		Notes
						Female	Male	Female	Male			Glucose	HbA1c	
1030	United Kingdom	2015	MRC National Survey of Health and Development	National	both	69-70	69-70	1,016	949	HbA1c	Yes		Lab	
1031	United Kingdom	2016	Health Survey for England	National	both	18+	18+	4,274	3,461	HbA1c	Yes		Lab	
1032	United Kingdom	2015-2016	National Diet and Nutrition Survey (NDNS)	National	both	18+	18+	370	273	Fasting glucose, HbA1c	No	Lab	Lab	
1033	United Kingdom	2015-2018	British Cohort Study 1970	National	both	45-48	45-48	4,426	4,154	HbA1c	Yes		Lab	
1034	United Kingdom	2016-2017	English Longitudinal Study of Ageing Wave 8	National	both	50+	50+	3,650	2,891	Fasting glucose, HbA1c	Yes	Lab	Lab	
1035	United Kingdom	2017	Health Survey for England	National	both	18+	18+	4,318	3,454	HbA1c	Yes		Lab	
1036	United Kingdom	2018	Health Survey for England	National	both	18+	18+	4,347	3,576	HbA1c	Yes		Lab	
1037	United Kingdom	2016-2019	National Diet and Nutrition Survey (NDNS)	National	both	18+	18+	318	234	Fasting glucose, HbA1c	No	Lab	Lab	
1038	United Kingdom	2018-2019	English Longitudinal Study of Ageing Wave 9	National	both	50+	50+	3,981	3,053	Fasting glucose, HbA1c	Yes	Lab	Lab	
1039	United Kingdom	2019	Health Survey for England	National	both	18+	18+	4,363	3,588	HbA1c	Yes		Lab	
1040	United Kingdom	2019	Understanding Society: Innovation Panel	National	both	18+	18+	214	181	HbA1c	No		Lab	
1041	United States of America	1976-1980	US NHANES II	National	both	18-74	18-74	6,236	5,594	Fasting glucose	Yes	Lab		27
1042	United States of America	1985-1986	Coronary Artery Risk Development in Young Adults (CARDIA)	Subnational	urban	18-30	18-30	2,633	2,198	Fasting glucose	No	Lab		
1043	United States of America	1987-1989	Atherosclerosis Risk in Communities Study	Subnational	both	44-66	44-66	6,222	5,051	Fasting glucose	Yes	Lab		
1044	United States of America	1989-1990	Cardiovascular Health Study	Subnational	both	65+	65+	3,321	2,462	Fasting glucose	Yes	Lab		
1045	United States of America	1990-1992	Atherosclerosis Risk in Communities Study	Subnational	both	46-70	46-70	5,631	4,546	Fasting glucose	Yes	Lab		
1046	United States of America	1988-1994	US NHANES III	National	both	20+	20+	9,702	8,806	Fasting glucose	Yes	Lab		
1047	United States of America	1992-1993	Coronary Artery Risk Development in Young Adults (CARDIA)	Subnational	urban	25-37	25-37	1,979	1,670	Fasting glucose	No	Lab		
1048	United States of America	1992-1993	Cardiovascular Health Study	Subnational	both	65+	65+	2,971	2,105	Fasting glucose	Yes	Lab		
1049	United States of America	1993-1995	Atherosclerosis Risk in Communities Study	Subnational	both	48-73	48-73	5,019	4,004	Fasting glucose	Yes	Lab		
1050	United States of America	1995-1996	The Bogalusa Heart Study	Community	rural	20-37	20-37	790	515	Fasting glucose	No	Lab		
1051	United States of America	1995-1996	Coronary Artery Risk Development in Young Adults (CARDIA)	Subnational	urban	28-40	28-40	1,923	1,595	Fasting glucose	No	Lab		
1052	United States of America	1996-1998	Atherosclerosis Risk in Communities Study	Subnational	both	50-75	50-75	4,497	3,561	Fasting glucose	Yes	Lab		
1053	United States of America	1996-1997	Cardiovascular Health Study	Subnational	both	67+	67+	2,504	1,567	Fasting glucose	Yes	Lab		
1054	United States of America	1996-1997	Study of Women's Health Across the Nation	Subnational	both	40-55		3,240		Fasting glucose	Yes	Lab		28
1055	United States of America	1997-1999	Study of Women's Health Across the Nation	Subnational	both	40-55		2,862		Fasting glucose	Yes	Lab		28
1056	United States of America	1999-2000	US NHANES 1999-2000	National	both	18+	18+	2,586	2,546	Fasting glucose, HbA1c	Yes	Lab	Lab	
1057	United States of America	1999-2001	Study of Women's Health Across the Nation	Subnational	both	40-56		2,694		Fasting glucose	Yes	Lab		28
1058	United States of America	2000-2001	Coronary Artery Risk Development in Young Adults (CARDIA)	Subnational	urban	33-45	33-45	1,823	1,474	Fasting glucose	No	Lab		
1059	United States of America	2000-2002	Study of Women's Health Across the Nation	Subnational	both	40-57		2,658		Fasting glucose	Yes	Lab		28
1060	United States of America	2001-2002	US NHANES 2001-2002	National	both	18+	18+	2,800	2,844	Fasting glucose, HbA1c	Yes	Lab	Lab	
1061	United States of America	2003-2004	US NHANES 2003-2004	National	both	18+	18+	2,654	2,695	Fasting glucose, HbA1c	Yes	Lab	Lab	
1062	United States of America	2004	2004 New York City HANES	Community	urban	20+	20+	1,129	824	Fasting glucose, HbA1c	Yes	Lab	Lab	
1063	United States of America	2005-2006	Coronary Artery Risk Development in Young Adults (CARDIA)	Subnational	urban	38-50	38-50	2,010	1,531	Fasting glucose	Yes	Lab		
1064	United States of America	2005-2006	Cardiovascular Health Study	Subnational	both	70+	70+	1,018	536	Fasting glucose	Yes	Lab		
1065	United States of America	2005-2006	US NHANES 2005-2006	National	both	18+	18+	2,519	2,671	Fasting glucose, HbA1c	Yes	Lab	Lab	
1066	United States of America	2007-2008	US NHANES 2007-2008	National	both	18+	18+	3,100	3,063	Fasting glucose, HbA1c	Yes	Lab	Lab	
1067	United States of America	2008-2009	National Longitudinal Study of Adolescent Health Wave IV	National	both	24-34	24-34	2,761	2,353	HbA1c	Yes		Lab	29
1068	United States of America	2009-2010	US NHANES 2009-2010	National	both	18+	18+	3,281	3,174	Fasting glucose, HbA1c	Yes	Lab	Lab	
1069	United States of America	2010-2011	Coronary Artery Risk Development in Young Adults (CARDIA)	Subnational	urban	43-55	43-55	1,971	1,510	Fasting glucose	Yes	Lab		

	Country	Data years	Survey/Study name/Citation	Level of representative-ness	Rural, urban, or both	Age range as used for global analysis		Sample size used for global analysis		Glycaemic markers available	Information available on diabetes treatment	Whether measured in a laboratory or by a point-of-care portable device		Notes
						Female	Male	Female	Male			Glucose	HbA1c	
1070	United States of America	2011-2013	Atherosclerosis Risk in Communities Study	Subnational	both	67-90	67-90	2,210	1,605	Fasting glucose, HbA1c	Yes	Lab	Lab	
1071	United States of America	2011-2012	US NHANES 2011-2012	National	both	18+	18+	2,907	2,894	Fasting glucose, HbA1c	Yes	Lab	Lab	
1072	United States of America	2013-2014	US NHANES 2013-2014	National	both	18+	18+	3,128	2,914	Fasting glucose, HbA1c	Yes	Lab	Lab	
1073	United States of America	2015-2016	US NHANES 2015-2016	National	both	18+	18+	3,030	2,886	Fasting glucose, HbA1c	Yes	Lab	Lab	
1074	United States of America	2017-2018	US NHANES 2017-2018	National	both	18+	18+	2,959	2,839	Fasting glucose, HbA1c	Yes	Lab	Lab	
1075	United States of America	2019-2020	US NHANES 2019-2020	Subnational	both	18+	18+	1,923	1,878	Fasting glucose, HbA1c	Yes	Lab	Lab	30
1076	Uruguay	1992	Enfermedades Cardiovasculares	National	both	19+	19+	574	449	Fasting glucose	No	Unknown		
1077	Uruguay	2011-2012	CESCAS Study	Community	urban	35-74	35-74	903	639	Fasting glucose	Yes	Lab		
1078	Uruguay	2013	STEPS	National	urban	18-64	18-64	1,468	872	Fasting glucose	Yes	Lab		
1079	Uruguay	2012-2016	Genotype, Phenotype and Environment of Hypertension in Uruguay (GEFA-HT-UY)	Community	urban	19+	19+	193	130	Fasting glucose	Yes	Lab		
1080	Uzbekistan	2014	STEPS	National	both	18-64	18-64	2,176	1,543	Fasting glucose	Yes	Portable		
1081	Uzbekistan	2015-2016	Epidemiology of Diabetes and Prediabetes in Uzbekistan Screening Results	Subnational	both	35+	35+	1,511	714	Fasting glucose, HbA1c	Yes	Portable	Lab	
1082	Uzbekistan	2019	STEPS	National	both	18-69	18-69	2,260	1,488	Fasting glucose	Yes	Portable		
1083	Vanuatu	1998	Vanuatu Non-communicable Disease Survey	National	both	20-59	20-59	179	185	Fasting glucose	No	Unknown		
1084	Vanuatu	2011	STEPS	National	both	25-64	25-64	2,236	2,312	Fasting glucose	Yes	Portable		
1085	Venezuela	2000	Zulia Coronary Heart Disease Risk Factor Study; Florez et al., Diabetes Res Clin Pract 69:63-77, 2005	Subnational	both	25+	25+	2,552	1,120	Fasting glucose	No	Unknown		
1086	Venezuela	1998-2001	Maracaibo aging study Santa lucia cohort	Community	urban	55+	55+	1,609	787	Fasting glucose	Yes	Lab		
1087	Venezuela	2004-2005	CArdiovascular Risk factors Multiple Evaluation in Latin America (CARMELA)	Community	urban	25-64	25-64	1,135	713	Fasting glucose	Yes	Lab		
1088	Venezuela	2005-2006	Brajkovich et al., Rev Ven Endoc Metab 4(3):31-32, 2006	Community	urban	20-65	20-65	331	114	Fasting glucose	No	Lab		
1089	Venezuela	2007-2008	Venezuelan Study of Metabolic Syndrome, Obesity and Lifestyle (VEMSOLS)	Community	urban	20+	20+	229	107	Fasting glucose	Yes	Lab		
1090	Venezuela	2008-2009	Venezuelan Study of Metabolic Syndrome, Obesity and Lifestyle (VEMSOLS)	Community	rural	20+	20+	89	51	Fasting glucose	Yes	Lab		
1091	Venezuela	2010-2011	Cardiometabolic risk factors in schoolchildren and adolescents of Mérida, Venezuela (CREDEFAR)	Community	urban	18	18	13	12	Fasting glucose	Yes	Lab		
1092	Venezuela	2010-2011	Venezuelan Study of Metabolic Syndrome, Obesity and Lifestyle (VEMSOLS)	Community	urban	20+	20+	154	51	Fasting glucose	No	Lab		
1093	Venezuela	2015-2017	Cardio-Metabolic Health Venezuelan Study (EVESCAM)	National	both	20+	20+	2,347	1,059	Fasting glucose	Yes	Lab		
1094	Venezuela	2014-2017	Maracaibo aging study Santa Rosa cohort	Community	urban	37+	37+	292	116	Fasting glucose, HbA1c	Yes	Lab	Lab	
1095	Venezuela	2018-2020	Cardio-metabolic Health Venezuelan Study (EVESCAM) follow-up	National	both	22+	22+	890	354	Fasting glucose	Yes	Lab		
1096	Viet Nam	1990	Quoc et al., Am J Epidemiol 139:713-22, 1994	Community	both	25-69	25-69	2,061	1,712	Fasting glucose	No	Unknown		
1097	Viet Nam	2001	Duc Son et al., Diabet Med 21:371-6, 2004	Community	both	25+	25+	2,001	654	Fasting glucose	No	Unknown		
1098	Viet Nam	2005	Non-communicable disease risk factors in Ho Chi Minh City	Community	urban	25-64	25-64	1,029	887	Fasting glucose	No	Portable		
1099	Viet Nam	2008-2009	The Survey on Diabetes and Its Risk Factors	Subnational	both	25+	25+	1,357	795	Fasting glucose, HbA1c	Yes	Lab	Lab	
1100	Viet Nam	2009	STEPS	National	both	25-64	25-64	7,754	6,677	Fasting glucose	Yes	Portable		
1101	Viet Nam	2012	National Survey of Diabetes in Vietnam	National	both	30-69	30-69	3,869	3,495	Fasting glucose	No	Portable		
1102	Viet Nam	2015	STEPS	National	both	18-69	18-69	2,043	1,669	Fasting glucose	Yes	Portable		
1103	Viet Nam	2021	STEPS	National	both	18+	18+	2,172	2,200	Fasting glucose	Yes	Portable		
1104	Yemen	2007-2009	Hypertension and Diabetes in Yemen (HYDY)	National	rural	18-70	18-70	2,408	2,358	Fasting glucose	Yes	Portable		
1105	Yemen	2007-2009	Hypertension and Diabetes in Yemen (HYDY)	National	urban	18-70	18-70	2,432	2,351	Fasting glucose	Yes	Portable		
1106	Zambia	2008	STEPS	Subnational	urban	25+	25+	1,219	631	Fasting glucose	Yes	Unknown		
1107	Zambia	2017	STEPS	National	both	18-69	18-69	2,513	1,614	Fasting glucose	Yes	Portable		
1108	Zimbabwe	2005	Zimbabwe Non-Communicable Disease Risk Factors (ZiNCoDs/STEPS)	National	both	25+	25+	1,393	444	Fasting glucose	No	Lab		

1. Sciensano, OD Public health and surveillance (2020). Health Interview Survey 2018 [Data file and code book]. Conditionally obtainable from the Sciensano website: https://www.sciensano.be/en/node/55737/health-interview-survey-microdata-request-procedure.

2. National studies from 2023 were assigned to 2022 so that they inform the estimates in countries with slightly later national data.

3. The first age group started from 15 years old, but had mean age ≥18 years.

4. The bibliographic citation for this data source is: Zeng, Yi, and Vaupel, James W. Chinese Longitudinal Healthy Longevity Survey (CLHLS), Biomarkers Datasets, 2009, 2012, 2014. Inter-university Consortium for Political and Social Research [distributor], 2019-01-15. https://doi.org/10.3886/ICPSR37226.v1.

5. This research uses data from China Health and Nutrition Survey (CHNS). We thank the National Institute for Health (NIH), the Eunice Kennedy Shriver National Institute of Child Health and Human Development (NICHD) for R01 HD30880, National Institute on Aging (NIA) for R01 AG065357, National Institute of Diabetes and Digestive and Kidney Diseases (NIDDK) for R01 DK104371 and P30 DK056350, National Heart, Lung, and Blood Institute (NHLBI) for R01 HL108427, the NIH Fogarty grant D43 TW009077, the Carolina Population Center for P2C HD050924 and P30 AG066615. We also thank the National Institute for Nutrition and Health, China Center for Disease Control and Prevention; Beijing Municipal Center for Disease Control and Prevention; the Chinese National Human Genome Center at Shanghai; and the China-Japan Friendship Hospital, National Health Commission of China.

6. The bibliographic citation for this data source is: Center for Healthy Aging and Development Studies, 2020, "The Chinese Longitudinal Healthy Longevity Survey (CLHLS)-Longitudinal Data（1998-2018）", https://doi.org/10.18170/DVN/WBO7LK, Peking University Open Research Data Platform, V2.

7. Santé publique France, en tant qu'investigateur principal, promoteur et financeur de l'étude ENNS.; Aux Centres d'examen de santé de la Caisse nationale d'assurance maladie des travailleurs salariés (CnamTS) et leurs laboratoires.

8. Santé publique France, en tant qu'investigateur principal, promoteur et financeur de l'étude Esteban.; Aux Centres d'examen de santé de la Caisse nationale d'assurance maladie des travailleurs salariés (CnamTS) et leurs laboratoires.

9. The authors thank the Heinz Nixdorf Foundation [Chairman: Martin Nixdorf; Past Chairman: Dr jur. Gerhard Schmidt], for their generous support of this study. Parts of the study were also supported by the German Research Council (DFG) [DFG project: EI 969/2-3, ER 155/6-1;6-2, HO 3314/2-1;2-2;3-4;3, INST 58219/32-1, JO 170/8-1, KN 885/3-1, PE 2309/2-1, SI 236/8-1;9-1;10-1,], the German Ministry of Education and Science [BMBF project: 01EG0401, 01GI0856, 01GI0860, 01GS0820_WB2-C, 01ER1001D, 01GI0205], the Ministry of Innovation, Science, Research and Technology, North Rhine-Westphalia (MIWFT-NRW), the Else Kröner-Fresenius-Stiftung [project: 2015_A119] and the German Social Accident Insurance [DGUV project: FF-FP295]. Furthermore the study was supported by the Competence Network for HIV/AIDS, the deanship of the University Hospital and IFORES of the University Duisburg-Essen, the European Union, the German Competence Network Heart Failure, Kulturstiftung Essen, the Protein Research Unit within Europe (PURE), the Dr. Werner-Jackstädt Stiftung and the following companies: Celgene GmbH München, Imatron/GE-Imatron, Janssen, Merck KG, Philips, ResMed Foundation, Roche Diagnostics, Sarstedt AG&Co, Siemens HealthCare Diagnostics, Volkswagen Foundation. The authors express their gratitude to all study participants of the Heinz Nixdorf Recall (HNR) Study, the personnel of the HNR study center and the EBT-scanner facilities, the investigative group and all former employees of the HNR study. The authors also thank the Advisory Board of the HNR Study: T. Meinertz, Hamburg, Germany (Chair); C. Bode, Freiburg, Germany; P.J. de Feyter, Rotterdam, Netherlands; B. Güntert, Hall i.T., Austria; F. Gutzwiller, Bern, Switzerland; H. Heinen, Bonn, Germany; O. Hess (†), Bern, Switzerland; B. Klein (†), Essen, Germany; H. Löwel, Neuherberg, Germany; M. Reiser, Munich, Germany; G. Schmidt (†), Essen, Germany; M. Schwaiger, Munich, Germany; C. Steinmüller, Bonn, Germany; T. Theorell, Stockholm, Sweden; and S.N Willich, Berlin, Germany.

10. Data have been provided by the Study of Health in Pomerania (SHIP) from the University Medicine Greifswald.

11. The CARRS Study was funded in part by the National Heart, Lung, and Blood Institute (NHLBI), National Institutes of Health (NIH), Department of Health and Human Services, under Contract No. HHSN268200900026C, the United Health Group, Minneapolis, MN, USA, and by the by the National Heart, Lung, And Blood Institute of the National Institutes of Health under Award Number P01HL154996.

12. Data on diabetes treatment were collected in the questionnaire from all participants, and at the time of examination which was done in randomly selected subset of 2,500 of the 30,000 PSUs. At the time of this analysis, only the data on diabetes treatment collected at the time of physical examination was available to us and was used in the analysis.

13. The Older Persons in Jamaica Study was funded by the National Health Fund, Jamaica.

14. The study was supported by the grant of the Ministry of Healthcare of the Republic of Kazakhstan "National Programme for the Introduction of Personalized and Preventive Medicine in The Republic of Kazakhstan (2021–2023)" (Grant number OR12165486).

15. The National Nutrition Survey of Kuwait (2008-2009) which was supported by The Kuwait Foundation for Advancement Sciences (KFAS) Grant # 2003-1202-02.

16. The MHAS Cognitive Aging Ancillary Study (Mex-Cog) is sponsored by the National Institutes of Health/National Institute on Aging (NIH R01AG051158). Data files and documentation are public use and available at www.MHASweb.org.

17. The Longitudinal Aging Study Amsterdam is supported by a grant from the Netherlands Ministry of Health Welfare and Sports, Directorate of Long-Term Care.

18. Data on 1,216 participants from one specific area in this study, which had high prevalence of thalassemia, were excluded. Glycaemic measurements in these participants were systematically different from the rest from the same study, possibly because of the high thalassemia prevalence.[41]

19. The bibliographic citation for this data source is: Am J Hypertens 2009 Jan;22(1):100-5 and Atherosclerosis. 2009 Mar;203(1):257-62.

20. Dr Take Naseri (Ministry of Health, Samoa), and Muagututia Sefuiva Reupena (Lutia I Puava Ae Mapu I Fagalele) contributed to the GWAS studies in Samoa.

21. The SP2 and SCCS2 studies are supported by individual research and clinical scientist award schemes from National Medical Research Council (NMRC) and the Biomedical Research Council (BMRC) of Singapore, the Singapore Ministry of Health, National University of Singapore and University Health System, Singapore.

22. The SH2012 and SH2 studies are supported by infrastructure funding from the Singapore Ministry of Health (Population Health Metrics Population Health Metrics and Analytics PHMA), National University of Singapore and National University Health System, Singapore.

23. The ULSAM study was supported by Uppsala University and Uppsala University Hospital.

24. The British Women's Heart and Health Study is supported by the British Heart Foundation (PG/13/66/30442). British Women's Heart and Health Study data are available to bona fide researchers for research purposes. Please refer to the BWHHS data sharing policy at http://www.ucl.ac.uk/british-womens-heart-health-study.

25. The Newcastle 85+ Study has been funded by the Medical Research Council, Biotechnology and Biological Sciences Research Council, the Dunhill Medical Trust and the National Institute for Health Research School for Primary Care. Parts of the work have also been funded by the British Heart Foundation, Unilever Corporate Research, Newcastle University, NHS North of Tyne (Newcastle Primary Care Trust). Mortality data was obtained from NHS Digital. We acknowledge the operational support of the North of England Commissioning Support Unit, the National Institute for Health Research Clinical Research Network North East and North Cumbria, local general practitioners and their staff. We thank the research nurses, laboratory technicians, data management and clerical team for their work throughout, as well as many colleagues for their expert advice. Thanks are due especially to the study participants and, where appropriate, their families and carers.

26. University of Essex. Institute for Social and Economic Research and National Centre for Social Research, Understanding Society: Waves 2 and 3 Nurse Health Assessment, 2010- 2012 [data collection]. 5th Edition. UK Data Service. SN:7251. http://doi.org/10.5255/UKDA-SN-7251-5.

27. National studies for the 3 years prior to 1980 were assigned to 1980 so that they inform the estimates in countries with slightly earlier national data.

28. The bibliographic citation for this data source is: Sutton-Tyrrell, Kim, Faith Selzer, MaryFran Sowers, Robert Neer, Lynda Powell, Ellen Gold, Gail Greendale, Gerson Weiss, Karen Matthews, and Sonja McKinlay. Study of Women's Health Across the Nation (SWAN), 1996-1997: Baseline Dataset. ICPSR28762-v2. Ann Arbor, MI: Inter-university Consortium for Political and Social Research[distributor], 2014-02-04. http://doi.org/10.3886/ICPSR28762.v2.

29. This research uses data from Add Health, a program project designed by J. Richard Udry, Peter S. Bearman, and Kathleen Mullan Harris, and funded by a grant P01-HD31921 from the Eunice Kennedy Shriver National Institute of Child Health and Human Development, with cooperative funding from 17 other agencies. Special acknowledgment is due Ronald R. Rindfuss and Barbara Entwisle for assistance in the original design. Persons interested in obtaining data files from Add Health should contact Add Health, Carolina Population Center, 123 W. Franklin Street, Chapel Hill, NC 27516-2524 (addhealth@unc.edu). No direct support was received from grant P01-HD31921 for this analysis.

30. Due to the COVID-19 pandemic the NHANES 2019-2020 cycle was not completed. As a result the data are not nationally representative and were considered subnational.

Appendix Table 2. List of analysis regions and super-regions, and countries in each region.

Super-region	Region
**Central and eastern Europe**	**Central Europe**: Albania, Bosnia and Herzegovina, Bulgaria, Croatia, Czechia, Hungary, Montenegro, North Macedonia, Poland, Romania, Serbia, Slovakia, Slovenia
	**Eastern Europe**: Belarus, Estonia, Latvia, Lithuania, Moldova, Russian Federation, Ukraine
**Central Asia, Middle East and north Africa**	**Central Asia**: Armenia, Azerbaijan, Georgia, Kazakhstan, Kyrgyzstan, Mongolia, Tajikistan, Turkmenistan, Uzbekistan
	**Middle East and north Africa**: Algeria, Bahrain, Egypt, Iran, Iraq, Jordan, Kuwait, Lebanon, Libya, Morocco, Oman, Qatar, Saudi Arabia, State of Palestine, Syrian Arab Republic, Tunisia, Türkiye, United Arab Emirates, Yemen
**High-income western**	**High-income English-speaking countries***: Australia, Canada, Ireland, New Zealand, United Kingdom, United States of America
	**Northwestern Europe**: Austria, Belgium, Denmark, Finland, Germany, Greenland, Iceland, Luxembourg, Netherlands, Norway, Sweden, Switzerland
	**Southwestern Europe**: Andorra, Cyprus, France, Greece, Israel, Italy, Malta, Portugal, Spain
**Latin America and the Caribbean**	**Andean Latin America**: Bolivia, Ecuador, Peru
	**The Caribbean**: Antigua and Barbuda, Bahamas, Barbados, Belize, Bermuda, Cuba, Dominica, Dominican Republic, Grenada, Guyana, Haiti, Jamaica, Puerto Rico, Saint Kitts and Nevis, Saint Lucia, Saint Vincent and the Grenadines, Suriname, Trinidad and Tobago
	**Central Latin America**: Colombia, Costa Rica, El Salvador, Guatemala, Honduras, Mexico, Nicaragua, Panama, Venezuela
	**Southern Latin America**: Argentina, Brazil, Chile, Paraguay, Uruguay
**Pacific island nations**	**Melanesia**: Fiji, Papua New Guinea, Solomon Islands, Vanuatu
	**Polynesia and Micronesia**: American Samoa, Cook Islands, French Polynesia, Kiribati, Marshall Islands, Federated States of Micronesia, Nauru, Niue, Palau, Samoa, Tokelau, Tonga, Tuvalu
**South Asia**	**South Asia**: Afghanistan, Bangladesh, Bhutan, India, Nepal, Pakistan, Sri Lanka
**East and southeast Asia and the Pacific**	**East Asia and the Pacific**: China, Japan, Singapore, South Korea, Taiwan
	**Southeast Asia**: Brunei Darussalam, Cambodia, Indonesia, Lao PDR, Malaysia, Maldives, Myanmar, North Korea, Philippines, Thailand, Timor-Leste, Viet Nam
**Sub-Saharan Africa**	**Central and southern Africa**: Angola, Botswana, Central African Republic, Congo, DR Congo, Equatorial Guinea, Gabon, Namibia
	**East Africa**: Burundi, Comoros, Djibouti, Eritrea, Eswatini, Ethiopia, Kenya, Lesotho, Madagascar, Malawi, Mozambique, Rwanda, Somalia, South Sudan, Sudan, Tanzania, Uganda, Zambia, Zimbabwe
	**West Africa**: Benin, Burkina Faso, Cabo Verde, Cameroon, Chad, Côte d'Ivoire, Gambia, Ghana, Guinea, Guinea Bissau, Liberia, Mali, Mauritania, Niger, Nigeria, Sao Tome and Principe, Senegal, Sierra Leone, Togo
	**Other sub-Saharan Africa**: Mauritius, Seychelles, South Africa

* Although high-income English-speaking countries are geographically separated, they experienced similar trends in cardiometabolic risk factors and outcomes.[1,2,16,32-38] They were therefore grouped together so that the statistical model shares information amongst them more than it does with other countries that are geographically closer but epidemiologically more distinct.

Appendix Table 3. Global age-standardised diabetes prevalence and number of people with diabetes in 1990 and 2022 for women and men aged 18+ years and 30+ years. Numbers in brackets are 95% credible intervals.

			18+ years	30+ years
Prevalence (%)	Women	1990	6.9 (5.7-8.1)	9.1 (7.6-10.7)
		2022	13.9 (12.3-15.8)	17.3 (15.4-19.5)
	Men	1990	6.8 (5.7-8.0)	8.9 (7.6-10.4)
		2022	14.3 (12.5-16.4)	17.9 (15.7-20.4)
Number (millions)	Women	1990	103.7 (86.1-123.0)	97.0 (81.3-114.3)
		2022	419.8 (372.2-473.5)	381.3 (340.0-428.4)
	Men	1990	93.8 (78.4-110.9)	86.1 (72.5-100.8)
		2022	408.0 (356.2-466.8)	367.4 (322.2-418.1)

Appendix Table 4. Comparison of age-standardised diabetes prevalence estimates in this work with recent global studies.

		Ong et al., 2023 [39]	Sun et al., 2022 [40]	NCD-RisC (this study)
**Women**	0+ years	5.8 (5.4-6.1)	n.a.	n.a.
	20-79 years	n.a.	10.2	14.1 (12.4-16.0)
	18+ years	n.a.	n.a.	13.9 (12.3-15.8)
**Men**	0+ years	6.5 (6.2-7.0)	n.a.	n.a.
	20-79 years	n.a.	10.8	14.6 (12.7-16.7)
	18+ years	n.a.	n.a.	14.3 (12.5-16.4)

Estimates from Ong et al. (2023) and Sun et al. (2022) are for 2021. Estimates from this study are for 2022.
n.a. = not available.

Appendix Table 5. Comparison of data sources and estimates of diabetes prevalence in this work with recent global studies, for the 30 countries with the largest adult population in 2022. For each country, the table shows the year of the most recent data source used and diabetes prevalence estimates.

Country	Years of the most recent data sources †			Estimates of age-standardised diabetes prevalence (%) ‡			
	Ong et al., 2023 [39]	Sun et al., 2022 [40]	NCD-RisC (this study)	Ong et al., 2023 [39] (0+ years)	Sun et al., 2022 [40] (20-79 years)	NCD-RisC (18+ years)	
						Women	Men
**China**	2016 (2013)	2017 (2017)	2018 (2015)	6.2 (5.7-6.6)	10.6	9.4 (5.4-14.7)	12.9 (7.4-19.7)
**India**	2017 (2014)	2015 (2015)	2021 (2018)	5.8 (5.4-6.4)	9.6	23.7 (18.0-29.9)	21.4 (16.4-27.1)
**USA**	2018 (2018)	2016 (2016)	2020 (2018)	9.0 (8.6-9.5)	10.7	11.4 (7.5-16.3)	13.6 (9.4-18.6)
**Indonesia**	2008 (2008)	2018 (2018)	2018 (2018)	4.6 (4.2-5.0)	10.6	14.5 (8.4-22.7)	10.9 (5.9-17.6)
**Brazil**	2008 (n.a.)	2019* (2019*)	2016 (2013)	5.4 (5.0-5.9)	8.8	14.0 (7.4-23.2)	11.7 (5.7-20.4)
**Pakistan**	2011 (n.a.)	2017 (2017)	2017 (2017)	7.1 (6.6-7.8)	30.8	30.9 (21.0-42.1)	30.8 (21.3-42.1)
**Bangladesh**	2018 (2018)	2018 (2018)	2018 (2018)	7.1 (6.7-7.6)	14.2	19.7 (12.9-27.7)	16.1 (9.9-23.7)
**Russian Federation**	2020 (n.a.)	2020 (2020)	2020 (2014)	4.0 (3.7-4.3)	5.6	8.3 (4.5-13.6)	7.6 (4.0-12.7)
**Nigeria**	2005 (n.a.)	2007 (2007)	2011 (n.a.)	3.7 (3.4-4.0)	3.6	11.2 (3.2-24.2)	10.7 (3.4-22.6)
**Japan**	2017 (2017)	2011* (2011*)	2023 (2019)	5.9 (5.4-6.4)	6.6	4.3 (2.8-6.2)	8.5 (6.2-11.4)
**Mexico**	2019 (2019)	2012 (2012)	2022 (2022)	9.1 (8.4-9.8)	16.9	15.2 (12.4-18.2)	13.2 (10.5-16.1)
**Philippines**	2014 (2014)	2008 (2008)	2021 (2021)	4.4 (4.1-4.8)	7.1	13.8 (8.6-19.9)	12.5 (7.8-18.8)
**Viet Nam**	2015 (2015)	2013 (2009)	2021 (2021)	4.5 (4.3-4.8)	6.1	11.1 (7.0-16.5)	10.4 (6.4-15.7)
**Germany**	2008 (n.a.)	2015* (2015*)	2019 (2012)	4.8 (4.4-5.2)	6.9	5.2 (2.5-9.2)	8.0 (4.4-12.8)
**Egypt**	2012 (2012)	2017 (2017)	2017 (2017)	8.4 (7.7-9.2)	20.9	28.8 (18.7-40.2)	26.3 (16.4-37.6)
**Ethiopia**	2015 (2015)	2015 (2015)	2015 (2015)	3.4 (3.1-3.6)	5.0	7.4 (2.6-14.7)	6.2 (2.3-12.6)
**Iran**	2011 (2011)	2007 (2007)	2021 (2021)	6.9 (6.3-7.5)	9.1	15.2 (12.3-18.6)	14.2 (11.2-17.5)
**Türkiye**	2011 (2011)	2010 (2010)	2017 (2017)	6.6 (6.1-7.2)	14.5	17.1 (10.6-24.7)	16.0 (10.0-23.3)
**Thailand**	2009 (2009)	2014 (2014)	2020 (2020)	5.4 (4.9-5.9)	9.7	12.8 (8.6-18.0)	11.7 (7.7-16.6)
**UK**	2013 (2013)	2021* (2021*)	2019 (2019)	7.8 (7.2-8.3)	6.3	7.9 (5.5-10.8)	9.7 (7.0-12.7)

**France**	2007 (2007)	2016 (2016)	2023 (2019)	3.6 (3.4-4.0)	5.3	1.8 (1.1-2.7)	3.7 (2.5-5.2)
**Italy**	2012 (2012)	2005* (2005*)	2020 (2012)	4.7 (4.3-5.2)	6.4	5.1 (2.7-8.4)	9.5 (5.7-14.1)
**DR Congo**	2007 (2005)	n.a. (n.a.)	2017 (n.a.)	4.8 (4.3-5.1)	5.8	10.0 (3.8-19.5)	8.7 (3.4-17.1)
**South Korea**	2019 (2019)	2015 (2015)	2023 (2023)	10.3 (9.8-10.8)	6.8	8.1 (6.7-9.6)	12.6 (10.6-14.7)
**South Africa**	2005 (n.a.)	2012* (2012*)	2020 (2012)	5.9 (5.5-6.3)	10.8	15.5 (7.8-25.6)	11.9 (5.8-20.1)
**Spain**	2011 (2010)	2010 (2010)	2019 (2015)	6.9 (6.4-7.3)	10.3	2.6 (1.3-4.4)	4.6 (2.6-7.2)
**Colombia**	2015 (2015)	2010 (n.a.)	2016 (2016)	6.1 (5.7-6.5)	8.3	12.2 (6.0-20.6)	12.3 (6.0-21.6)
**Myanmar**	2014 (2014)	2014 (2014)	2014 (2014)	8.0 (7.5-8.5)	7.1	14.9 (7.2-25.1)	12.5 (5.9-21.6)
**Argentina**	1995 (n.a.)	1995 (n.a.)	2018 (2018)	5.5 (5.0-6.0)	5.4	10.4 (5.4-17.0)	10.7 (5.8-17.1)
**Tanzania**	2012 (2012)	2012 (2012)	2023 (2023)	2.6 (2.4-2.8)	12.3	7.3 (4.1-11.6)	5.8 (3.3-9.3)

† For each country, the first number shows the year of the most recent data source, and the number in brackets shows the year of the most recent national data source. Data sources that only reported type 1 diabetes are not included in this table because type 1 diabetes is an outcome only in Ong et al. (2023) but not in Sun et al. (2022) or this study.
‡ Estimates from Ong et al. (2023) and Sun et al. (2022) are for both sexes in 2021. Estimates from this study are for 2022. Numbers in brackets are 95% uncertainty intervals, when reported.
* Data were based on self-reported diabetes diagnosis, medical records, or a registry of people with diagnosed diabetes, handled as described in Research in Context panel.
n.a. = not available.

Appendix Figure 1. Number of data sources used in this analysis, by country.

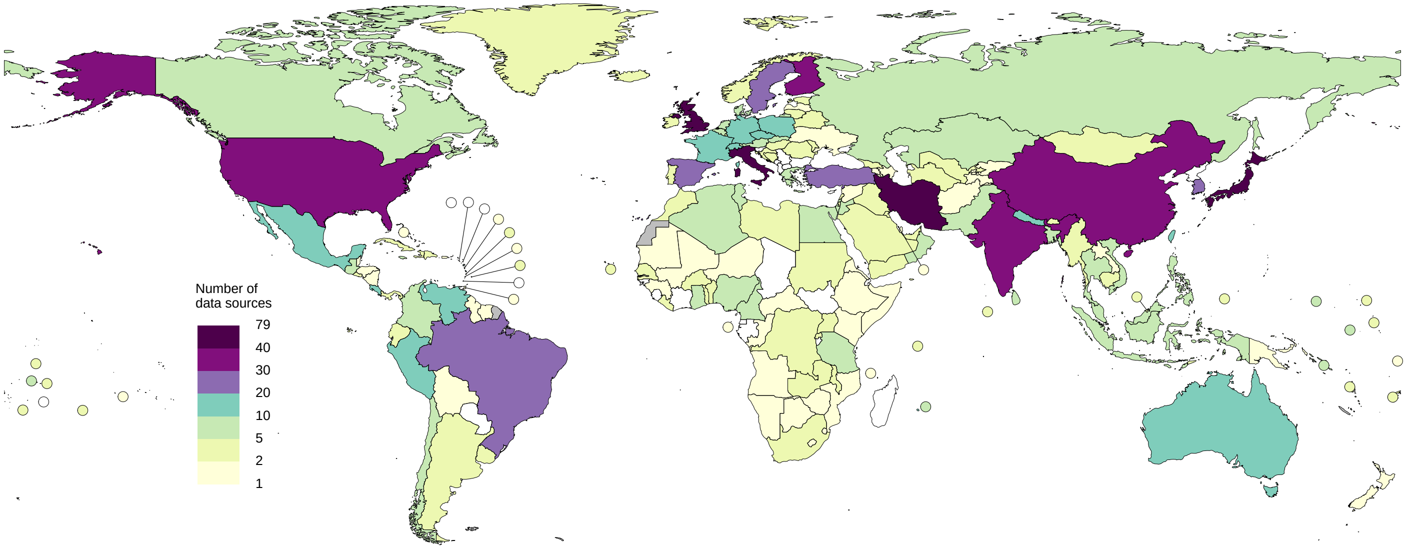
Number of data sources
79
40
30
20
10
5
2
1

Appendix Figure 2. Number of data sources used in this paper, by region and year. The size of each circle shows the number of data sources for each region and year, and the colours indicate the relative count of national, subnational and community data sources.

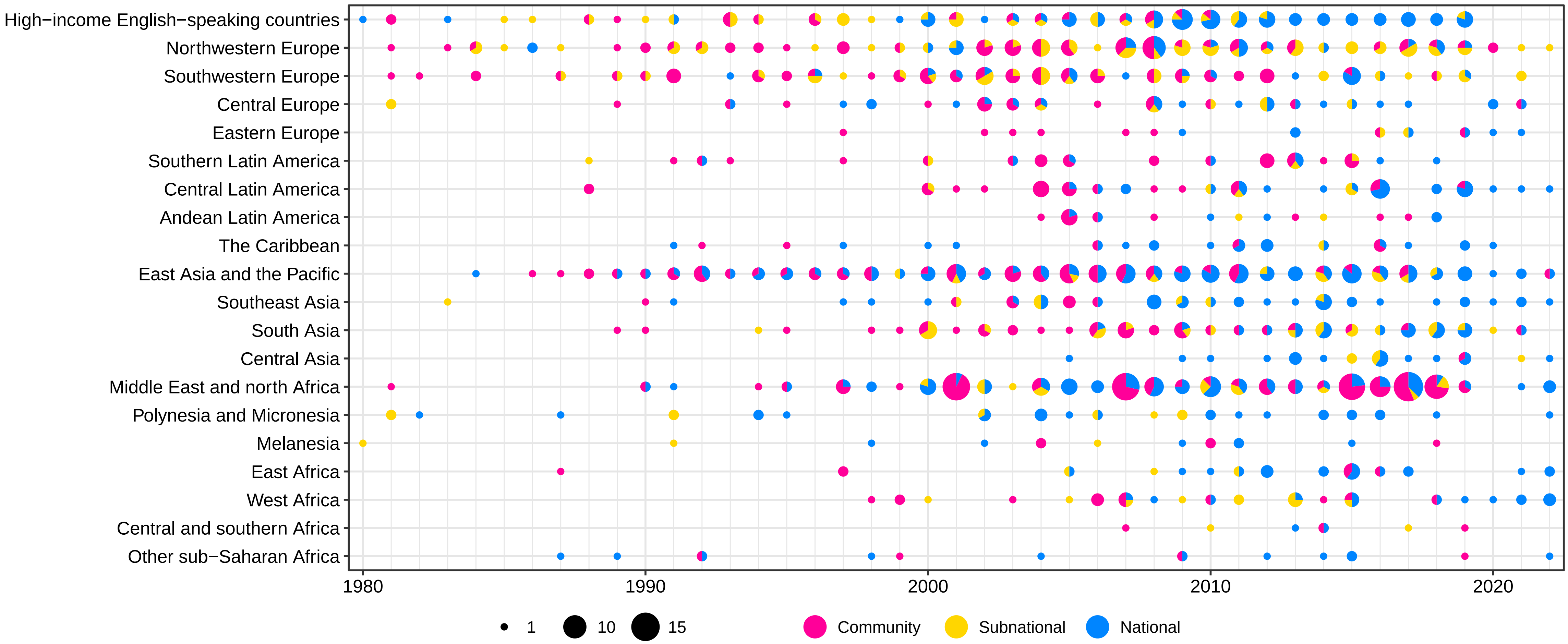

High−income English−speaking countries
Northwestern Europe
Southwestern Europe
Central Europe
Eastern Europe
Southern Latin America
Central Latin America
Andean Latin America
The Caribbean
East Asia and the Pacific
Southeast Asia
South Asia
Central Asia
Middle East and north Africa
Polynesia and Micronesia
Melanesia
East Africa
West Africa
Central and southern Africa
Other sub−Saharan Africa
1980
1990
2000
2010
2020
1
10
15
Community
Subnational
National

Appendix Figure 3. Posterior probability that age-standardised diabetes prevalence for people aged 18+ years increased from 1990 to 2022.

The maps show the PP of an increase from 1990 to 2022. The PP of a decrease is one minus that of an increase. The density plot alongside each map shows the smoothed distribution of estimates across countries.

Women

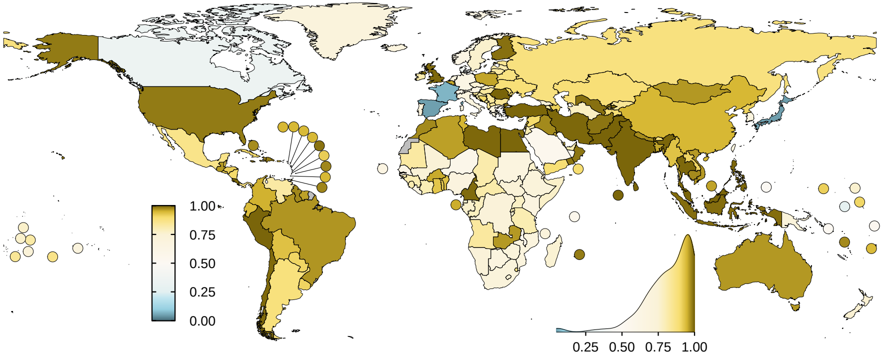

1.00
0.75
0.50
0.25
0.00
0.25
0.50
0.75
1.00

Men

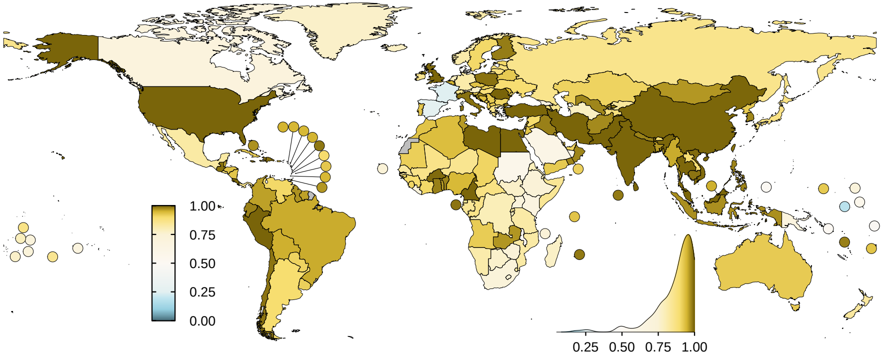

1.00
0.75
0.50
0.25
0.00
0.25
0.50
0.75
1.00

Appendix Figure 4. Posterior probability that age-standardised diabetes treatment coverage for people aged 30+ years increased from 1990 to 2022.

The maps show the PP of an increase from 1990 to 2022. The PP of a decrease is one minus that of an increase. The density plot alongside each map shows the smoothed distribution of estimates across countries.

Women

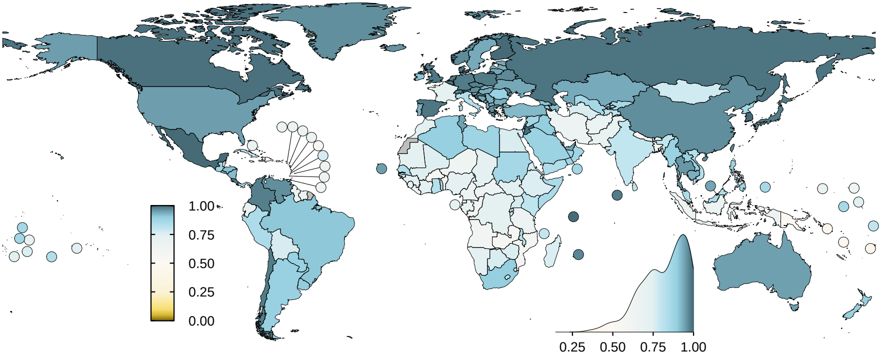

1.00
0.75
0.50
0.25
0.00
0.25 0.50 0.75 1.00

Men

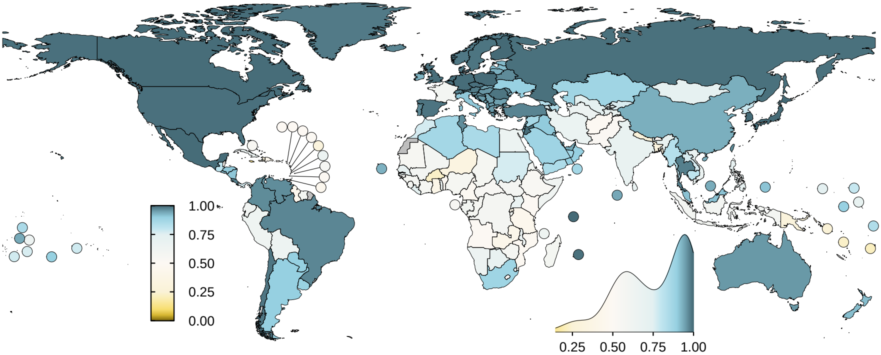

1.00
0.75
0.50
0.25
0.00
0.25 0.50 0.75 1.00

Appendix Figure 5. Relationship between levels in 1990 and 2022 for age-standardised diabetes prevalence for people aged 18+ years and age-standardised treatment coverage for people aged 30+ years.

Each point shows one country. Points that are filled have a posterior probability (PP) >0.80 of the observed change being a true decrease or increase. If neither an increase nor a decrease was detected at PP = 0.80 the point is hollow.

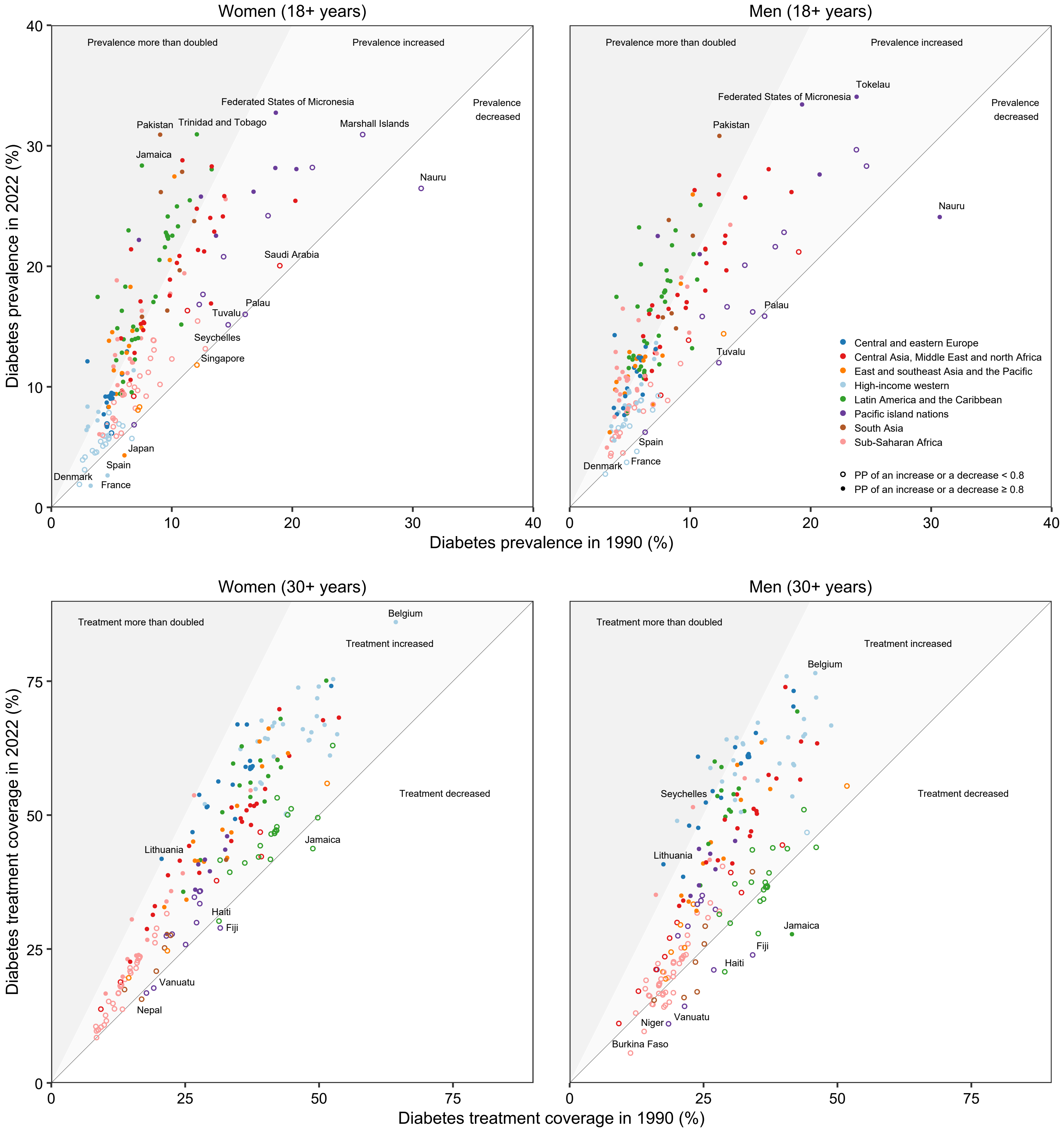

Women (18+ years)
Men (18+ years)
Prevalence more than doubled
Prevalence increased
Prevalence decreased
Federated States of Micronesia
Pakistan
Trinidad and Tobago
Marshall Islands
Jamaica
Nauru
Saudi Arabia
Palau
Tuvalu
Seychelles
Singapore
Japan
Spain
Denmark
France
Tokelau
Diabetes prevalence in 2022 (%)
Diabetes prevalence in 1990 (%)
Central and eastern Europe
Central Asia, Middle East and north Africa
East and southeast Asia and the Pacific
High-income western
Latin America and the Caribbean
Pacific island nations
South Asia
Sub-Saharan Africa
PP of an increase or a decrease < 0.8
PP of an increase or a decrease ≥ 0.8
Women (30+ years)
Men (30+ years)
Treatment more than doubled
Treatment increased
Treatment decreased
Belgium
Lithuania
Haiti
Fiji
Vanuatu
Nepal
Niger
Burkina Faso
Diabetes treatment coverage in 2022 (%)
Diabetes treatment coverage in 1990 (%)

Appendix Figure 6. Relationship of age-standardised diabetes prevalence and treatment coverage between women and men in 2022. Each point shows one country.

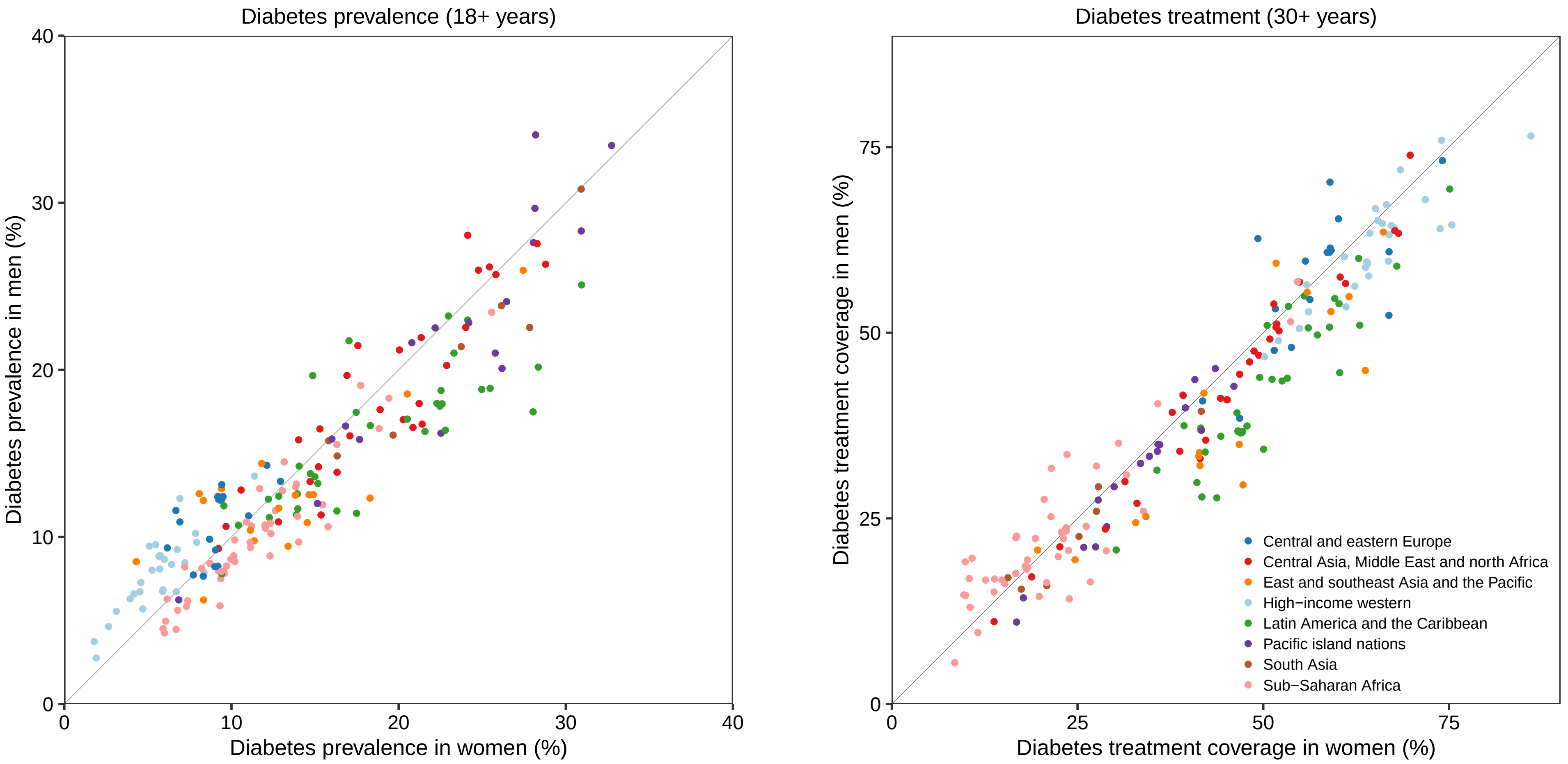
Diabetes prevalence (18+ years)
Diabetes prevalence in men (%)
Diabetes prevalence in women (%)
0
10
20
30
40
Diabetes treatment (30+ years)
Diabetes treatment coverage in men (%)
Diabetes treatment coverage in women (%)
0
25
50
75
Central and eastern Europe
Central Asia, Middle East and north Africa
East and southeast Asia and the Pacific
High−income western
Latin America and the Caribbean
Pacific island nations
South Asia
Sub−Saharan Africa

Appendix Figure 7. Relationship between age-standardised and crude prevalence of diabetes among women and men aged 18+ years in 2022. Each point shows one country.

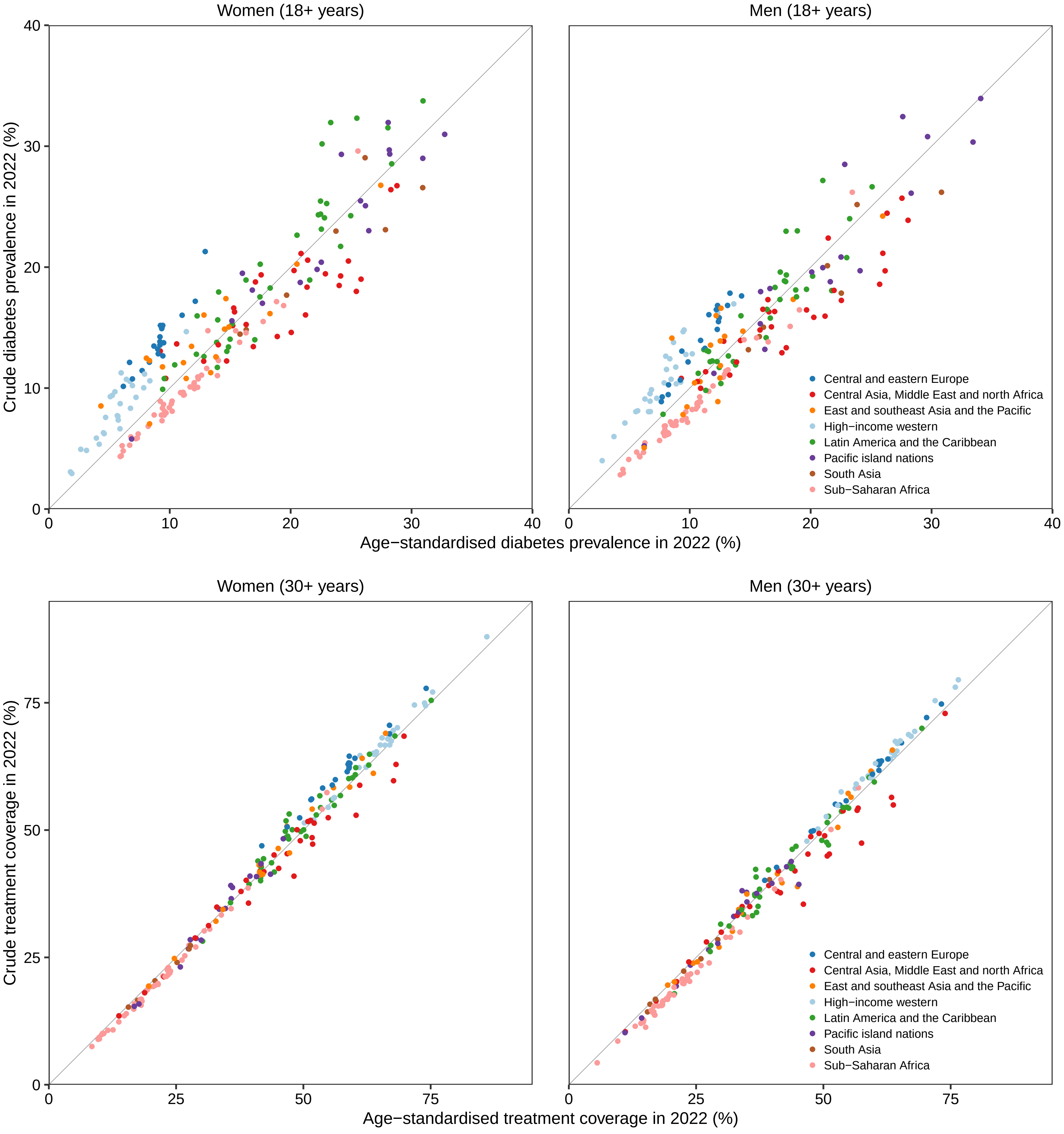

Women (18+ years)
Men (18+ years)
Crude diabetes prevalence in 2022 (%)
Age−standardised diabetes prevalence in 2022 (%)
Women (30+ years)
Men (30+ years)
Crude treatment coverage in 2022 (%)
Age−standardised treatment coverage in 2022 (%)
Central and eastern Europe
Central Asia, Middle East and north Africa
East and southeast Asia and the Pacific
High−income western
Latin America and the Caribbean
Pacific island nations
South Asia
Sub−Saharan Africa

Appendix Figure 8. Median age of women and men with diabetes in 2022 by country.

Women

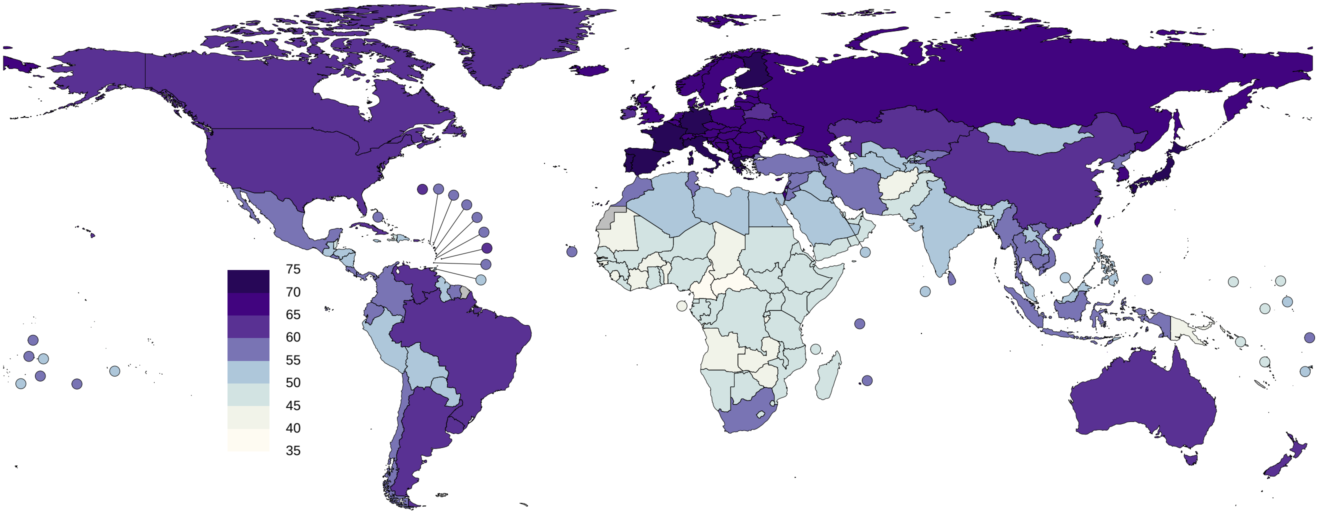

Men

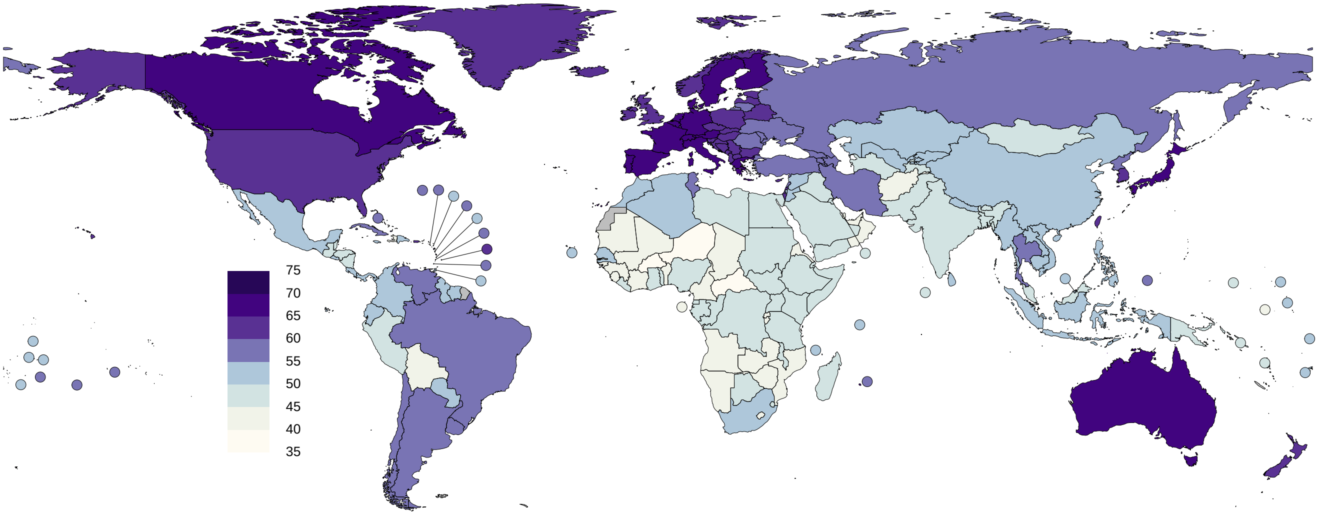

Appendix Figure 9. Age-standardised diabetes prevalence for people aged 18+ years from 1990 to 2022 by country, for both sexes combined.

See main paper Figure 1 caption for descriptions of the contents of the figure and for definitions.

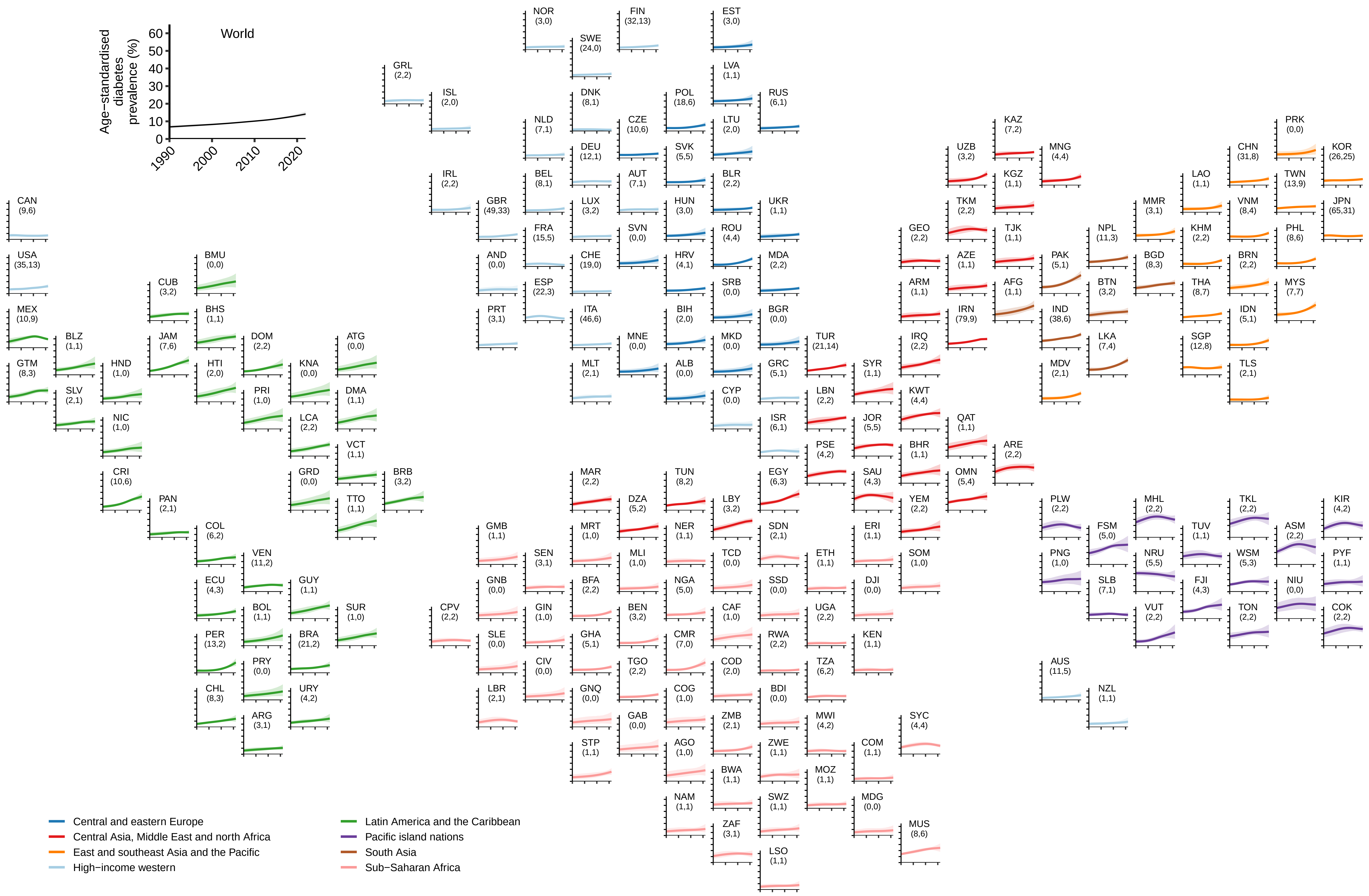

Age−standardised diabetes prevalence (%)
World
60
50
40
30
20
10
0
1990
2000
2010
2020
NOR (3,0)
FIN (32,13)
EST (3,0)
SWE (24,0)
GRL (2,2)
LVA (1,1)
ISL (2,0)
DNK (8,1)
POL (18,6)
RUS (6,1)
NLD (7,1)
CZE (10,6)
LTU (2,0)
KAZ (7,2)
PRK (0,0)
DEU (12,1)
SVK (5,5)
UZB (3,2)
MNG (4,4)
CHN (31,8)
KOR (26,25)
IRL (2,2)
BEL (8,1)
AUT (7,1)
BLR (2,2)
KGZ (1,1)
LAO (1,1)
TWN (13,9)
CAN (9,6)
GBR (49,33)
LUX (3,2)
HUN (3,0)
UKR (1,1)
TKM (2,2)
MMR (3,1)
VNM (8,4)
JPN (65,31)
FRA (15,5)
SVN (0,0)
ROU (4,4)
GEO (2,2)
TJK (1,1)
NPL (11,3)
KHM (2,2)
PHL (8,6)
USA (35,13)
BMU (0,0)
AND (0,0)
CHE (19,0)
HRV (4,1)
MDA (2,2)
AZE (1,1)
PAK (5,1)
BGD (8,3)
BRN (2,2)
CUB (3,2)
ESP (22,3)
SRB (0,0)
ARM (1,1)
AFG (1,1)
BTN (3,2)
THA (8,7)
MYS (7,7)
MEX (10,9)
BHS (1,1)
PRT (3,1)
ITA (46,6)
BIH (2,0)
BGR (0,0)
IRN (79,9)
IND (38,6)
IDN (5,1)
BLZ (1,1)
JAM (7,6)
DOM (2,2)
ATG (0,0)
MNE (0,0)
MKD (0,0)
TUR (21,14)
IRQ (2,2)
LKA (7,4)
SGP (12,8)
GTM (8,3)
HND (1,0)
HTI (2,0)
KNA (0,0)
MLT (2,1)
ALB (0,0)
GRC (5,1)
SYR (1,1)
MDV (2,1)
TLS (2,1)
SLV (2,1)
PRI (1,0)
DMA (1,1)
CYP (0,0)
LBN (2,2)
KWT (4,4)
NIC (1,0)
LCA (2,2)
ISR (6,1)
JOR (5,5)
QAT (1,1)
VCT (1,1)
PSE (4,2)
BHR (1,1)
ARE (2,2)
CRI (10,6)
GRD (0,0)
BRB (3,2)
MAR (2,2)
TUN (8,2)
EGY (6,3)
SAU (4,3)
OMN (5,4)
PAN (2,1)
TTO (1,1)
DZA (5,2)
LBY (3,2)
YEM (2,2)
PLW (2,2)
MHL (2,2)
TKL (2,2)
KIR (4,2)
COL (6,2)
GMB (1,1)
MRT (1,0)
NER (1,1)
SDN (2,1)
ERI (1,1)
FSM (5,0)
TUV (1,1)
ASM (2,2)
VEN (11,2)
SEN (3,1)
MLI (1,0)
TCD (0,0)
ETH (1,1)
SOM (1,0)
PNG (1,0)
NRU (5,5)
WSM (5,3)
PYF (1,1)
ECU (4,3)
GUY (1,1)
GNB (0,0)
BFA (2,2)
NGA (5,0)
SSD (0,0)
DJI (0,0)
SLB (7,1)
FJI (4,3)
NIU (0,0)
BOL (1,1)
SUR (1,0)
CPV (2,2)
GIN (1,0)
BEN (3,2)
CAF (1,0)
UGA (2,2)
VUT (2,2)
TON (2,2)
COK (2,2)
PER (13,2)
BRA (21,2)
SLE (0,0)
GHA (5,1)
CMR (7,0)
RWA (2,2)
KEN (1,1)
PRY (0,0)
CIV (0,0)
TGO (2,2)
COD (2,0)
TZA (6,2)
AUS (11,5)
CHL (8,3)
URY (4,2)
LBR (2,1)
GNQ (0,0)
COG (1,0)
BDI (0,0)
NZL (1,1)
ARG (3,1)
GAB (0,0)
ZMB (2,1)
MWI (4,2)
SYC (4,4)
STP (1,1)
AGO (1,0)
ZWE (1,1)
COM (1,1)
BWA (1,1)
MOZ (1,1)
NAM (1,1)
SWZ (1,1)
MDG (0,0)
ZAF (3,1)
MUS (8,6)
LSO (1,1)
Central and eastern Europe
Central Asia, Middle East and north Africa
East and southeast Asia and the Pacific
High−income western
Latin America and the Caribbean
Pacific island nations
South Asia
Sub−Saharan Africa

Appendix Figure 10. Levels in 2022 and change from 1990 to 2022 by country of (A) age-standardised diabetes prevalence for people aged 18+ years, and (B) age-standardised treatment coverage for people aged 30+ years, for both sexes combined.

The density plot alongside each map shows the smoothed distribution of estimates across countries.

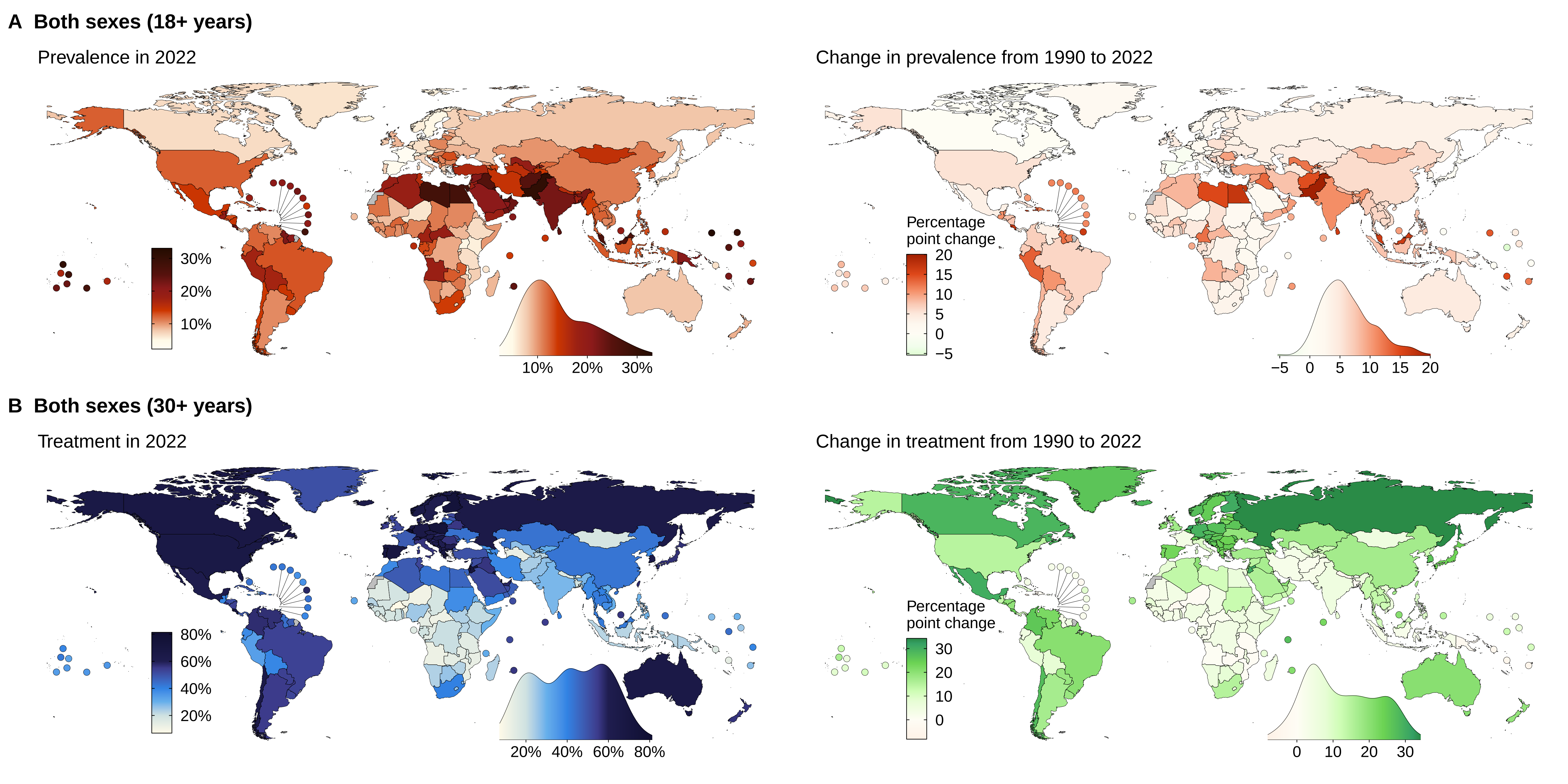
A Both sexes (18+ years)
Prevalence in 2022
30%
20%
10%
10% 20% 30%
Change in prevalence from 1990 to 2022
Percentage point change
20
15
10
5
0
−5
−5 0 5 10 15 20
B Both sexes (30+ years)
Treatment in 2022
80%
60%
40%
20%
20% 40% 60% 80%
Change in treatment from 1990 to 2022
Percentage point change
30
20
10
0
0 10 20 30

Appendix Figure 11. Age-standardised treatment coverage for people aged 30+ years in 1990 and 2022, for both sexes combined.

Each line represents a country, with countries coloured by the super-region in which they fall. The black triangle shows the age-standardised treatment coverage for the world.

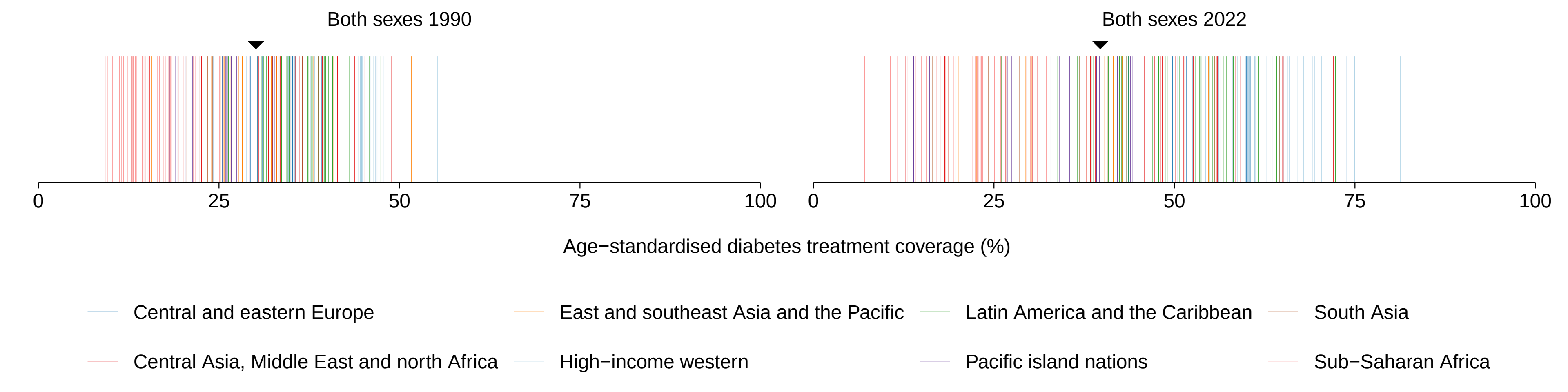

Both sexes 1990
Both sexes 2022
0
25
50
75
100
Age−standardised diabetes treatment coverage (%)
Central and eastern Europe
East and southeast Asia and the Pacific
Latin America and the Caribbean
South Asia
Central Asia, Middle East and north Africa
High−income western
Pacific island nations
Sub−Saharan Africa

References

1. NCD Risk Factor Collaboration (NCD-RisC). Worldwide trends in diabetes since 1980: a pooled analysis of 751 population-based studies with 4.4 million participants. *Lancet* 2016; **387**(10027): 1513-30.
2. NCD Risk Factor Collaboration (NCD-RisC). Worldwide trends in underweight and obesity from 1990 to 2022: a pooled analysis of 3663 population-representative studies with 222 million children, adolescents, and adults. *Lancet* 2024; **403**(10431): 1027-50.
3. Danaei G, Finucane MM, Lin JK, et al. National, regional, and global trends in systolic blood pressure since 1980: systematic analysis of health examination surveys and epidemiological studies with 786 country-years and 5·4 million participants. *Lancet* 2011; **377**(9765): 568-77.
4. Danaei G, Finucane MM, Lu Y, et al. National, regional, and global trends in fasting plasma glucose and diabetes prevalence since 1980: systematic analysis of health examination surveys and epidemiological studies with 370 country-years and 2·7 million participants. *Lancet* 2011; **378**(9785): 31-40.
5. Farzadfar F, Finucane MM, Danaei G, et al. National, regional, and global trends in serum total cholesterol since 1980: systematic analysis of health examination surveys and epidemiological studies with 321 country-years and 3·0 million participants. *Lancet* 2011; **377**(9765): 578-86.
6. Finucane MM, Stevens GA, Cowan MJ, et al. National, regional, and global trends in body-mass index since 1980: systematic analysis of health examination surveys and epidemiological studies with 960 country-years and 9·1 million participants. *Lancet* 2011; **377**(9765): 557-67.
7. Ogurtsova K, Guariguata L, Barengo NC, et al. IDF Diabetes Atlas: global estimates of undiagnosed diabetes in adults for 2021. *Diabetes Res Clin Pract* 2022; **183**: 109118.
8. NCD Risk Factor Collaboration (NCD-RisC). Global variation in diabetes diagnosis and prevalence based on fasting glucose and hemoglobin A1c. *Nat Med* 2023; **29**(11): 2885-901.
9. John WG, Mosca A, Weykamp C, Goodall I. HbA1c standardisation: history, science and politics. *Clin Biochem Rev* 2007; **28**(4): 163-8.
10. Carstensen B, Lindstrom J, Sundvall J, Borch-Johnsen K, Tuomilehto J, DPS Study Group. Measurement of blood glucose: comparison between different types of specimens. *Ann Clin Biochem* 2008; **45**(Pt 2): 140-8.
11. Cowie CC, Rust KF, Ford ES, et al. Full accounting of diabetes and pre-diabetes in the U.S. population in 1988-1994 and 2005-2006. *Diabetes Care* 2009; **32**(2): 287-94.
12. Anjana RM, Unnikrishnan R, Deepa M, et al. Metabolic non-communicable disease health report of India: the ICMR-INDIAB national cross-sectional study (ICMR-INDIAB-17). *Lancet Diabetes Endocrinol* 2023; **11**(7): 474-89.
13. Diabetes Control Complications Trial Research Group, Nathan DM, Genuth S, et al. The effect of intensive treatment of diabetes on the development and progression of long-term complications in insulin-dependent diabetes mellitus. *N Engl J Med* 1993; **329**(14): 977-86.
14. UK Prospective Diabetes Study (UKPDS) Group. Intensive blood-glucose control with sulphonylureas or insulin compared with conventional treatment and risk of complications in patients with type 2 diabetes (UKPDS 33). *Lancet* 1998; **352**(9131): 837-53.
15. Finucane MM, Paciorek CJ, Danaei G, Ezzati M. Bayesian estimation of population-level trends in measures of health status. *Stat Sci* 2014; **29**(1): 18-25.
16. NCD Risk Factor Collaboration (NCD-RisC). Worldwide trends in hypertension prevalence and progress in treatment and control from 1990 to 2019: a pooled analysis of 1201 population-representative studies with 104 million participants. *Lancet* 2021; **398**(10304): 957-80.
17. Finucane MM, Paciorek CJ, Stevens GA, Ezzati M. Semiparametric Bayesian density estimation with disparate data sources: a meta-analysis of global childhood undernutrition. *J Am Stat Assoc* 2015; **110**(511): 889-901.
18. Gelman A. Prior distributions for variance parameters in hierarchical models (comment on article by Browne and Draper). *Bayesian Anal* 2006; **1**(3): 515-34.

19. Breslow NE, Clayton DG. Approximate inference in generalized linear mixed models. *J Am Stat Assoc* 1993; **88**(421): 9-25.
20. Wood SN. Generalized Additive Models: An Introduction with R. Boca Raton, FL: Chapman & Hall/CRC; 2006.
21. Rue H, Held L. Gaussian Markov Random Fields: Theory and Applications. New York: CRC Press; 2005.
22. Harville DA. Matrix Algebra from a Statistician's Perspective: Springer-Verlag; 2008.
23. Danaei G, Finucane MM, Lin JK, et al. National, regional, and global trends in systolic blood pressure since 1980: systematic analysis of health examination surveys and epidemiological studies with 786 country-years and 5.4 million participants. *Lancet* 2011; **377**(9765): 568-77.
24. Singh GM, Danaei G, Pelizzari PM, et al. The age associations of blood pressure, cholesterol, and glucose: analysis of health examination surveys from international populations. *Circulation* 2012; **125**(18): 2204-11.
25. United Nations Department of Economic and Social Affairs, Population Division. World Urbanization Prospects: The 2018 Revision. New York: United Nations, 2019.
26. Smith AF, Roberts GO. Bayesian computation via the Gibbs sampler and related Markov chain Monte Carlo methods. *J R Stat Soc Series B Stat Methodol* 1993; **55**(1): 3-23.
27. Vehtari A, Gelman A, Simpson D, Carpenter B, Bürkner P-C. Rank-normalization, folding, and localization: an improved R for assessing convergence of MCMC (with discussion). *Bayesian Anal* 2021; **16**(2): 667-718.
28. Gelman A, Hwang J, Vehtari A. Understanding predictive information criteria for Bayesian models. *Stat Comput* 2014; **24**(6): 997-1016.
29. Stan Development Team. RStan: the R interface to Stan. 2022. https://mc-stan.org/. .
30. Ahmad OB, Boschi-Pinto C, Lopez AD, Murray CJ, Lozano R, Inoue M. Age standardization of rates: a new WHO standard. GPE Discussion Paper Series 31. Geneva: World Health Organization, 2001.
31. United Nations, Department of Economic and Social Affairs, Population Division. World Population Prospects 2024, Online Edition., 2024.
32. NCD Risk Factor Collaboration (NCD-RisC). Repositioning of the global epicentre of non-optimal cholesterol. *Nature* 2020; **582**(7810): 73-7.
33. NCD Risk Factor Collaboration (NCD-RisC). Trends in adult body-mass index in 200 countries from 1975 to 2014: a pooled analysis of 1698 population-based measurement studies with 19.2 million participants. *Lancet* 2016; **387**(10026): 1377-96.
34. NCD Risk Factor Collaboration (NCD-RisC). Rising rural body-mass index is the main driver of the global obesity epidemic in adults. *Nature* 2019; **569**(7755): 260-4.
35. NCD Risk Factor Collaboration (NCD-RisC). Height and body-mass index trajectories of school-aged children and adolescents from 1985 to 2019 in 200 countries and territories: a pooled analysis of 2181 population-based studies with 65 million participants. *Lancet* 2020; **396**(10261): 1511-24.
36. NCD Risk Factor Collaboration (NCD-RisC). Diminishing benefits of urban living for children and adolescents' growth and development. *Nature* 2023; **615**(7954): 874-83.
37. NCD Risk Factor Collaboration (NCD-RisC). Worldwide trends in blood pressure from 1975 to 2015: a pooled analysis of 1479 population-based measurement studies with 19.1 million participants. *Lancet* 2017; **389**(10064): 37-55.
38. NCD Risk Factor Collaboration (NCD-RisC). Worldwide trends in body-mass index, underweight, overweight, and obesity from 1975 to 2016: a pooled analysis of 2416 population-based measurement studies in 128.9 million children, adolescents, and adults. *Lancet* 2017; **390**(10113): 2627-42.
39. GBD Diabetes Collaborators. Global, regional, and national burden of diabetes from 1990 to 2021, with projections of prevalence to 2050: a systematic analysis for the Global Burden of Disease Study 2021. *Lancet* 2023; **402**(10397): 203-34.
40. Sun H, Saeedi P, Karuranga S, et al. IDF Diabetes Atlas: global, regional and country-level diabetes prevalence estimates for 2021 and projections for 2045. *Diabetes Res Clin Pract* 2022; **183**: 109119.

41. Shabbir S, Nadeem M, Sattar A, et al. Type and frequency of hemoglobinopathies, diagnosed in the area of Karachi, in Pakistan. *Cogent Med* 2016; **3**: 1188875.
